# A Facile Route to Flavone-3-Carboxamides and Flavone-3-Carboxylates via Palladium-Catalyzed Amino- and Aryloxy-Carbonylation Reactions

**DOI:** 10.3390/ijms251810128

**Published:** 2024-09-20

**Authors:** Sami Chniti, László Kollár, Attila Bényei, Ágnes Dörnyei, Attila Takács

**Affiliations:** 1 Department of General and Inorganic Chemistry, University of Pécs, Ifjúság útja 6., H-7624 Pécs, Hungary; samichniti@yahoo.fr (S.C.); kollar@gamma.ttk.pte.hu (L.K.); 2 HUN-REN-PTE Research Group for Selective Chemical Syntheses, Ifjúság útja 6., H-7624 Pécs, Hungary; 3 János Szentágothai Research Centre, University of Pécs, Ifjúság útja 20., H-7624 Pécs, Hungary; dornyei@gamma.ttk.pte.hu; 4 Department of Physical Chemistry, University of Debrecen, Egyetem tér 1., H-4032 Debrecen, Hungary; benyei.attila@science.unideb.hu; 5 Department of Analytical and Environmental Chemistry, University of Pécs, Ifjúság útja 6., H-7624 Pécs, Hungary

**Keywords:** carbon monoxide, aminocarbonylation, aryloxycarbonylation, flavon-3-carboxamides, flavone-3-carboxylates

## Abstract

A library of C-3 functionalized flavones was successfully provided via palladium-catalyzed amino- and aryloxycarbonylation reactions of 3-iodoflavone (**1**), under mild conditions. This methodology showed good functional group tolerance using a variety of amines and phenols, under an atmospheric pressure of carbon monoxide as a carbonyl source. While the flavone-3-carboxamides (**2a-t**) were produced in 22–79%, the flavone-3-carboxylates (**4a′-l′**) were obtained in excellent yields (up to 88%), under identical reaction conditions, just by switching *N*-nucleophiles to *O*-nucleophiles. The convenient availability of the involved starting materials confers simplicity to this approach to design new C-3-substituted flavones of biological relevance. The solid-state structures of flavone-3-carboxamide (**2r**) and flavone-3-ester (**4f′**) were further studied by single-crystal XRD analysis.

## 1. Introduction

Flavones represent an important subgroup of flavonoids possessing 2-phenyl-4H-chromen-4-one as the basic skeleton [1]. The outstanding pharmacological profile of remarkable naturally occurring and structurally modulated flavone derivatives has gained a great deal of attention among chemists and biologists [2,3,4]. As a privileged scaffold, the flavone backbone has been embedded in many marketed drugs such as *Flavoxate*, *Efloxate*, and *Vitexin* [5]. Therefore, further designing new hybrids to generate potentially important candidates is still a valid task. To date, the C-3 functionalization of flavones has been reported as a captivating tool to access different building blocks of considerable interest [6,7,8,9,10,11].

**Figure 1:**
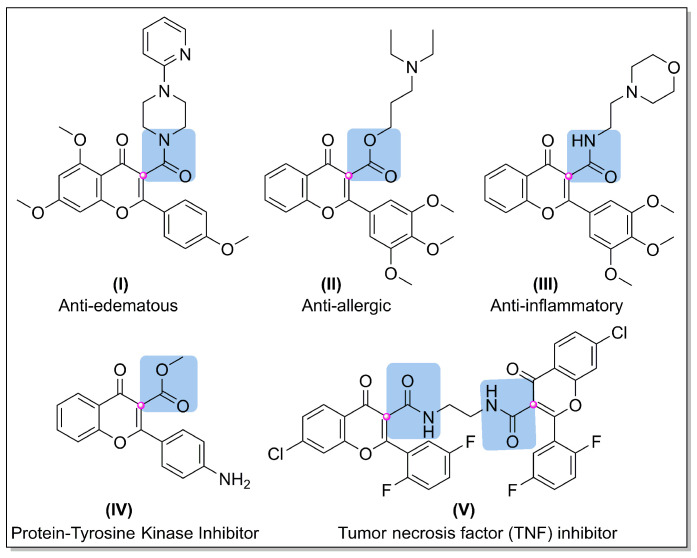
Examples of biologically active flavone-based carboxamides and carboxylates.

More importantly, a literature survey disclosed that biologically active flavone-3-carboxylates and flavone-3-carboxamides have barely been documented. As far as we could ascertain, only a few reports mentioned these fascinating molecular architectures (Figure 1), which could exhibit either anti-edematous (**I**), anti-allergic (**II**), and anti-inflammatory activities (**III**) [12] and showed the ability to act as selective inhibitors of the protein-tyrosine kinase enzyme (**IV**) [13] and the tumor necrosis factor (**V**) [14].

**Scheme 1:**
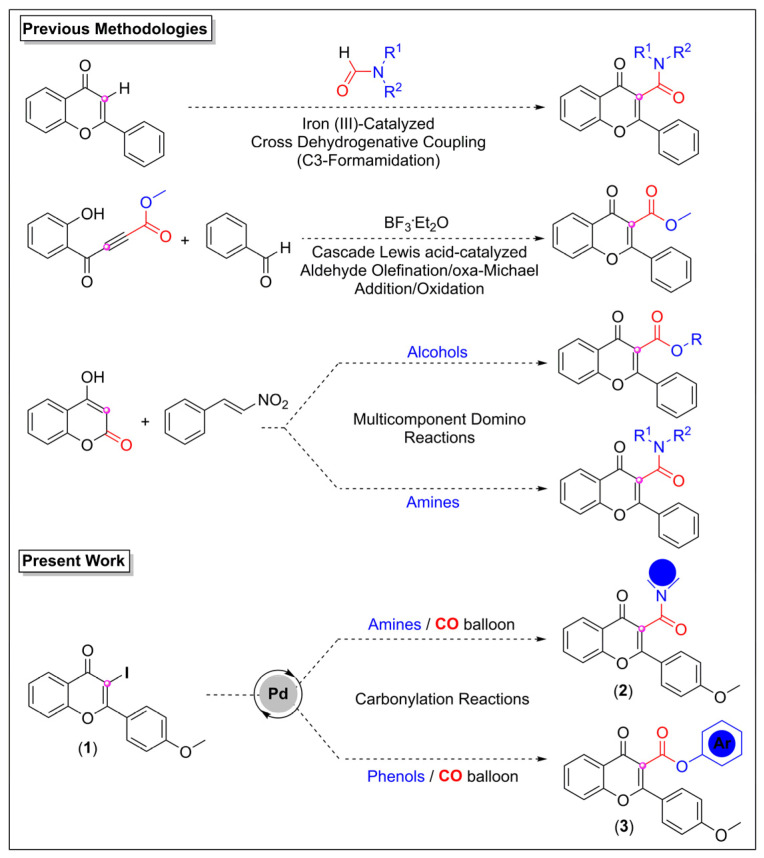
Some representative synthetic strategies and the hereby proposed routes for flavone-3-carboxamides and flavone-3-carboxylates.

On the other hand, known synthetic routes to flavone-3-carboxylates and flavone-3-carboxamides have been described as classical esterification [12], amidation [15], and direct C-3 formamidation reactions [16] that require harsh conditions and tedious work-ups. Seemingly, cascade [17] and tandem [18] transformations as well as formal [3 + 3]-cycloadditions [19] and multi-component domino reactions [20,21] could be alternative procedures but involve the use of complex mixtures of reagents, suffer from long reaction times, and mostly provide low yields (Scheme 1). Consequently, looking for more practical and simple protocols for producing the target flavone-3-carboxamides and flavone-3-carboxylates seems essential to overcome these limitations.

Transition metal-catalyzed carbonylation reactions have represented, up until now, an efficient method to install a set of functionalities such as amides, carboxylic acids, esters, ketoamides, carbamates, ketones, aldehydes, and azides into a large variety of activated substrates (aryl-, heteroaryl-, alkenyl halides, etc.) [22]. The high chemoselectivity of such cross-coupling transformation and its tolerance to a wide range of partners are some of the best features of this methodology, making carbonylation reactions an intriguing candidate for analyses using novel strategies to quantify the influence of substitutions on the reactivity and specificity of the carbonyl group [23].

Interestingly, the introduction of an iodine atom at the C-3 position of a flavone skeleton allows for a plethora of further late-stage functionalization through C-C cross-coupling reactions [24,25,26,27]. In continuation of our constant efforts in exploring new halo(hetero)arenes models for palladium-catalyzed carbonylation reactions [28,29,30,31,32,33], we planned to use a 3-iodoflavone derivative as a substrate for building the target flavone-3-carboxamides and flavone-3-carboxylates via amino- and aryloxycarbonylation reactions, respectively (Scheme 1). To check the feasibility of this peculiar approach, we studied the behavior of an easily accessible 3-iodoflavone derivative **1** (2-(4′-methoxyphenyl)-3-iodo-4*H*-chromen-4-one) under palladium-catalyzed carbonylation conditions in the presence of different amines and phenols.

## 2. Results and Discussions

### 2.1. Synthesis of Flavone-3-Carboxamides via Aminocarbonylation of 3-Iodoflavone 1

In the first part of this study, we were interested in the design of flavone-3-carboxamide type **2** compounds. For this, a set of experiments were carried out with the starting 3-iodoflavone (**1**) and L-alanine methyl ester (**a**), and the reactions were monitored by GC-MS and ^1^H NMR measurements (Table 1).

A preliminary reaction was performed in DMF at 50 °C in the presence of Et_3_N as a base when the Pd(OAc)_2_/PPh_3_ in situ catalyst was introduced in a 1:2 ratio [34,35,36]. The reaction was very slow, and the desired flavone-3-carboxamide **2a** was provided only with 50% conversion after 72 h (Table 1, entry 1). When the reaction was carried out at 80 °C under standard reaction conditions, complete conversion of the starting material was accomplished after 72 h (Table 1, entry 2).

A careful checking of the crude reaction subjected to column chromatography unveiled the presence of flavone-3-carboxamide **2a** isolated as a major compound in a 42% yield (Table 1, entry 2) and of *N*-substituted β-enaminone **3a** as a side-product, albeit in a small amount (10% yield). Both compounds were fully characterized using NMR and GC-MS measurements. It is worth noting that coumarin-enaminone **3a** (*N*-substituted-β-enaminone) formation stemmed from an interesting flavone–coumarin rearrangement under palladium-catalyzed carbonylation conditions.

**Scheme 2:**
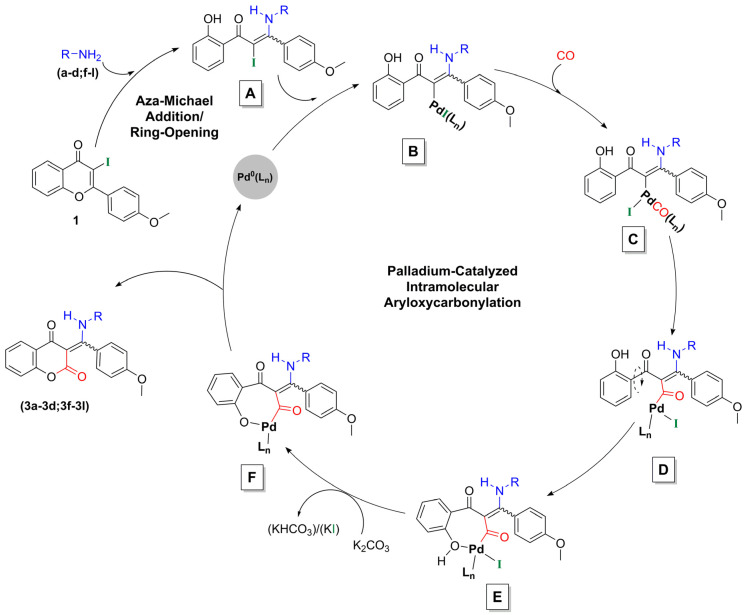
Plausible mechanism for the formation of compound **3**.

Given the tendency of 3-iodoflavone **1** (Michael acceptor) to undergo a ring-opening/ring-closing process in the presence of amines [37], the compound of type **3** formation could simply be described as a domino aza-Michael addition/ring-opening/palladium-catalyzed intramolecular aryloxycarbonylation sequence (Scheme 2, steps A–F). This rearrangement had been recently studied by our research group in the chromone series (flavone-like derivative), and a similar mechanism was proposed [28].

Afterward, bearing in mind our main goal to produce target carboxamides efficiently and selectively, the optimization of model reaction parameters was necessary to find suitable conditions, with respect to ligand type, solvent, base, temperature, and carbon monoxide pressure.

Notwithstanding the high-pressure carbon monoxide (40 bar) applied at 50 °C and 80 °C, under the same initial experimental conditions, the reaction provided only the expected flavone-3-carboxamide **2a,** and no double-carbonylated derivative (flavone-3-glyoxylamide) was detected. Based on the GC-MS analysis, only 20% and 85% conversions were reached at 50 °C and 80 °C, respectively (Table 1, entries 3 and 4). Interestingly, a fine-tuning of the ligand structure showed a significant effect on the reaction course. When the reaction was carried out at 80 °C, dppf could reduce the reaction time to 24 h, with a small increase in the isolated yield (49%) compared to the reaction with triphenylphosphine (Table 1, entry 5). On the other hand, incomplete conversion was observed after 72 h with more flexible bidentate ligands such as dppp (Table 1, entry 6). Furthermore, based on the previously established effectivity of XantPhos for palladium-catalyzed aminocarbonylation reactions [38,39], we examined the reaction model in the presence of this bidentate ligand. Expectedly, the reaction in DMF in the presence Et_3_N at 80 °C led to the target carboxamide **2a** with 78% selectivity within 12 h. The main compound was isolated in 56% of yield (Table 1, entry 7), and only 16% of *N*-substituted-β-enaminone **3a** was obtained.

Our attempts to identify the most suitable solvent, keeping XantPhos as the appropriate ligand, revealed that different polar and nonpolar solvents such as acetonitrile and toluene gave moderate yields (40% and 55%, respectively) and longer reaction times (Table 1, entries 8 and 9), whereas conversion was incomplete when 1,4-dioxane was used (Table 1, entry 10).

When a high temperature (80 °C) was applied in DMF, the use of an inorganic base (K_2_CO_3_) instead of Et_3_N could significantly decrease the reaction time to 12 h with an excellent selectivity toward **2a** (96%) and an improvement in yield (72%) (Table 1, entry 11).

Gratifyingly, the reaction carried out in DMF at 50 °C in the presence of K_2_CO_3_ proceeded to completion over 24 h and also showed a very high selectivity and a comparable yield (79%) to the reaction performed at 80 °C (Table 1, compare entries 11 and 12). A longer reaction time (24 h, at 50 °C) was identified as a suitable compromise because a higher temperature (80 °C) could remarkably speed up the reaction (12 h), but it gave an inferior yield (72%). It should be emphasized that increasing the temperature could favor the hydrodeiodination process that 3-iodoflavone **1** could undergo, leading to the corresponding flavone [40].

After the optimization of the reaction parameters, we decided to explore the applicability of this palladium-catalyzed aminocarbonylation protocol on 3-iodoflavone **1**, under optimal conditions (Table 1, entry 12), using a broad range of primary and secondary amines. 

The screening of enantiopure α-amino acid esters (**a**-**e**) provided the target carboxamides (**2a**-**2e**) within 24 h, with very high selectivity (Table 2, entries 1–6). The corresponding carboxamides **2a-e** were isolated in good yields, ranging from 46% to 79%, whereas *N*-substituted β-enaminones **3a-b** were obtained in very low yields. Moreover, when L-serine methyl ester (**e**) was used as an *N*-nucleophile, the hydroxyl group remained unreacted, and no ester-type compound was formed (Table 2, entry 5).

The use of an aromatic amine such as aniline was able to shift selectivity toward *N*-substituted-β-enaminone **3f**. A mixture of **2f/3f** was obtained in a 1:3 ratio, and carboxamide form **2f** was isolated, albeit in a 22% yield (Table 2, entry 7). Furthermore, aliphatic primary amines such as 4-picolylamine (**g**) and piperonylamine (**j)** exhibited good selectivity, giving rise to corresponding carboxamides (**2g**, and **2j**) in moderate yields (36–44%), while benzylamine (**h**) and phenethylamine (**i**) provided carboxamides (**2h**-**2i**) in moderate yields (41–44%), alongside *N*-substituted β-enaminone (**3h**-**j**) formation, (Table 2, entries 7–10).

Outstandingly, the reaction proceeded well for a range of secondary amines (**m**-**q**, **s**-**t**) and revealed excellent selectivity toward the corresponding flavone-3-carboxamides (**2m**-**q** and **2s**-**t**). The target compounds were isolated in moderate-to-good yields (41–77%) (Table 2, entries 13–17 and 19–20).

Unlike the secondary amines used, both *N*,*O*-dimethylhydroxylamine (**k**) and diethylamine (**l**) afforded a mixture of the corresponding flavone-3-carboxamides and *N*-substituted-β-enaminone counterparts in an 18:1 ratio. The target carboxamides **2k** and **2l** were isolated in good yields (71% and 57%) (Table 2, entries 11–12).

Finally, we could prepare a promising adamantyl-based flavone-3-carboxamide **2r** in a satisfactory yield (46%), using bulky and sterically hindered 1-adamantylamine (**r**), a subunit of multifaceted value in drug design [41,42].

The inspection of the results gathered in Table 2 reveals that this synthetic approach exhibits notable chemoselectivity to flavone-3-carboxamides **2**, as *N*-substituted β-enaminones **3** could be supplied in low yields only with some involved amines. Mechanistically, this interesting feature could be explained by the structural and electronic properties of the starting iodoheteroarene **1**. Hence, the presence of a bulkier aryl group at the C-2 position of the 3-iodoflavone **1** core can relatively stabilize the pyrone ring (Michael acceptor) once prone to *N*-nucleophile attack, like amines. Consequently, the ring-opening/ring-closing process, promoting side-product **3** formation, could be notoriously less favored. Instead, aminocarbonylation might easily occur at the C-3 site of the flavone moiety.

**Figure 2:**
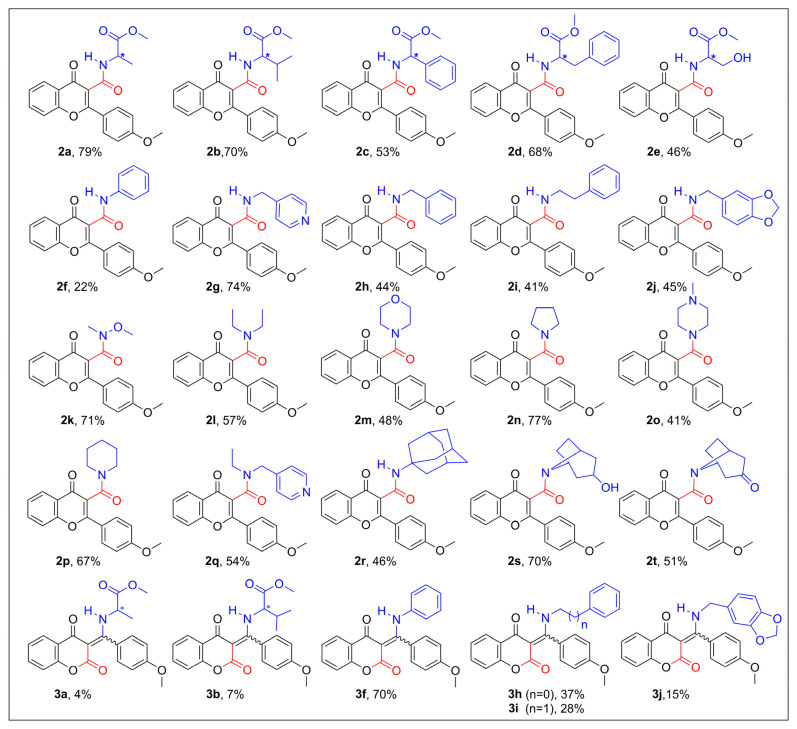
Sketch of prepared flavone-3-carboxamides (**2**) and coumarin-based *N*-substituted β-enaminones (**3**), isolated and fully characterized.

Figure 2 summarizes a set of twenty new flavone-3-carboxamides (**2a**-**2t**) prepared in moderate-to-good yields. Additionally, we were able to further isolate, in small amounts, some coumarin-based *N*-substituted β-enaminones **3** (Figure 2). This might deserve more interest in terms of increased scaffold diversification, as both carbonylated products (**2** and **3**) belong to privileged classes with wide spectra of biological activities and relevant photophysical properties [43]. Further investigations aiming to selectively obtain the coumarin-based *N*-substituted β-enaminones **3** under palladium-catalyzed carbonylation conditions could be the aim of future projects.

### 2.2. Synthesis of Flavone-3-Esters (4) via Aryloxycarbonylation of 3-Iodoflavone (1)

From the same perspective, to check the performance of the previously optimized protocol, we turned our attention to the use of phenol derivatives such as *O*-nucleophiles to access new flavone-3-esters **4** starting from 3-iodoflavone **1** under palladium-catalyzed aryloxycarbonylation conditions.

**Figure 3:**
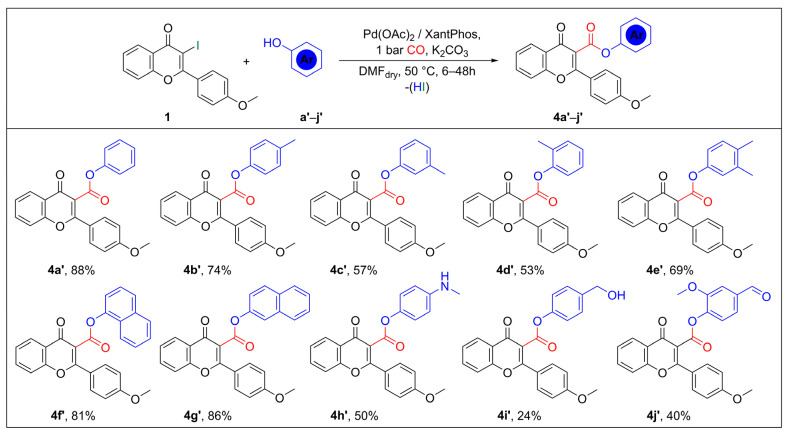
Palladium-catalyzed aryloxycarbonylation of 3-iodoflavone **1** ^(a)^. (a) Experimental protocol: 0.5 mmol of 3-iodoflavone **1**, 0.55 mmol of solid phenols (0.75 mmol of liquid phenols), 0.0125 mmol of Pd(OAc)_2_, 0.0125 mmol of XantPhos, 0.75 mmol of K_2_CO_3_, and 5 mL of dry DMF, at 50 °C, under 1 bar of carbon monoxide, for 6–48 h.

Initially, substrate **1** was reacted with phenol (**a′**) in the presence of the Pd(OAc)_2_/XantPhos catalyst in DMF at 50 °C under atmospheric carbon monoxide pressure using K_2_CO_3_. The reaction successfully provided the expected flavone-3-ester **4a′** in an excellent yield (88%), within 6 h (Figure 3). Encouraged by the excellent result above, we examined various phenols (**b′-j′**) under the same experimental conditions. To our delight, this efficient catalytic system showed compatibility for most of the *O*-nucleophiles evaluated, giving the corresponding flavone-3-esters in moderate-to-excellent yields.

Phenols bearing electron-releasing (4-CH_3_, 3-CH_3_, 2-CH_3_, and 3,4-CH_3_) substituents at the aryl ring afforded the corresponding 3-substituted flavones in good yields (53–74%, **4b′**-**4e′**). Furthermore, 1-naphthol (**f′**) and 2-naphthol (**g′**) exhibited excellent reactivity, giving the expected product in excellent yields (81–86%, **4f′**-**4g′**). Moreover, phenols bearing different functionalities, such as secondary amine, alcohol, and aldehyde, are also tolerated for the aryloxycarbonylation reaction of substrate **1**, providing the corresponding esters (**3h′-j′**) in moderate yields (24–50%).

## 3. X-ray Crystallographic Study

The unambiguous molecular structures of **2r** and **4f′** were established by X-ray diffraction analysis. Details of the crystal parameters, data collection, and structure refinement are given in Table 3.

The geometric parameters for both structures are given in Supporting Information File (Pages S1–S23, Figures S1 and S2). The supplementary crystallographic data for each compound can be obtained free of charge from the Cambridge Crystallographic Data Centre via http://www.ccdc.cam.ac.uk/data_request/cif accessed on 16 September 2024, using the following reference deposition numbers: CCDC2369639 for **2r**, and CCDC2369640 for **4f′**.

### 3.1. Molecular Structure Analysis

It is worth mentioning that the listed geometric data and the present preliminary structural analysis can serve as a starting point for computational studies to explore the quantitative energy and electron density landscape of the intermolecular interactions in a solid state for these compounds.

The crystal structure of **2r** is solved and refined in the orthorhombic space group P2_1_2_1_2_1_ with four symmetry-independent molecules.

**Figure 4:**
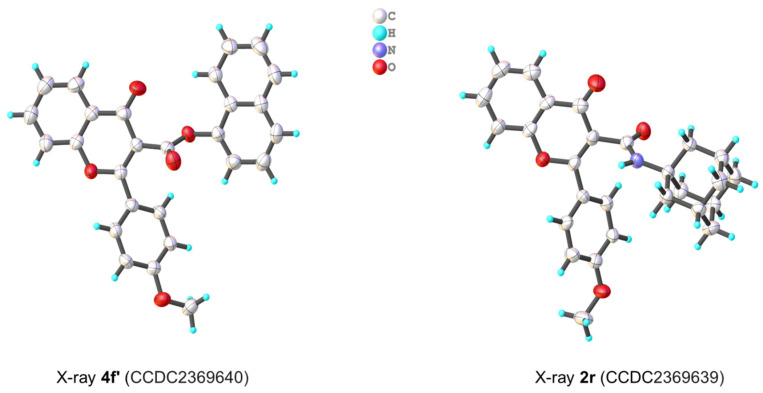
ORTEP diagram of solid-state structures showing thermal displacement ellipsoids, drawn at the 50% probability level (graphic designed using the Olex2-1.5 program).

The inspection of the crystal lattice content showed that all molecules exhibited an intriguing twist, and no rotation around the amide moiety was detected in a solid state (Figure 4).

The resulting *trans*-conformation of the amide bond (**∠**O = C−N−H ≈ 177°) was thermodynamically more favorable due to the mutual repulsion of the bulkier adamantyl group, which conferred an interesting conformational rigidity to the flavone derivative.

On the other hand, the title compound naphthalen-1-yl-2-(4-methoxyphenyl)-4-oxo-4H-chromene-3-carboxylate **4f′** was crystallized in the monoclinic space group P2_1_/c with four non-planar molecules in the asymmetric unit cell.

The crystal lattice clearly showed *syn*-oriented pairs of **4f′**, which were *anti*-oriented relative to one another (Figure 4).

### 3.2. Molecular Packing Analysis

**Figure 5:**
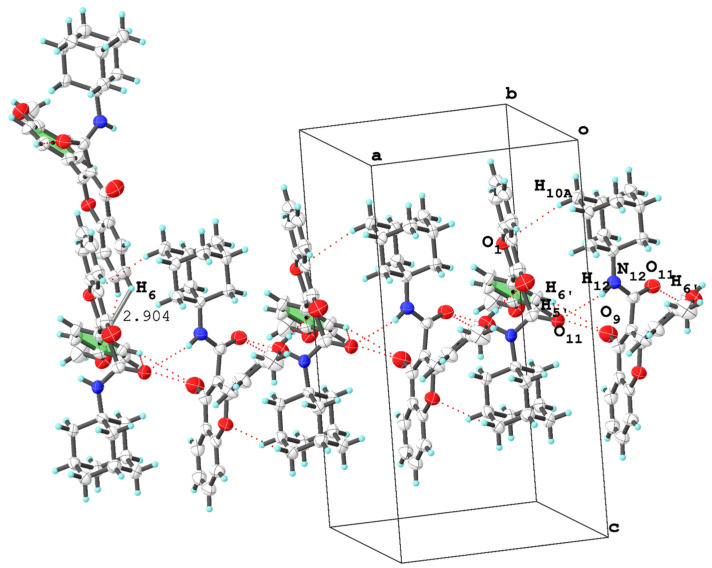
ORTEP plot of fragments of crystal packings [view normal to (010)] of *N*-(adamantan-1-yl)-2-(4-methoxyphenyl)-4-oxo-4*H*-chromene-3-carboxamide **2r**, showing thermal displacement ellipsoids, drawn at the 50% probability level (graphic designed using the Olex2 program). The red dashed lines highlight the inter/intramolecular hydrogen bonds and weak interactions. The gray solid lines indicate possible *edge-to-face* (CH···π) aromatic stackings. The indicated values show the interplanar distances (Å).

The crystal packing of compound **2r** was probably stabilized by a network of inter- and intramolecular N–H···O; C;–H;···O; and aromatic C;–H;···π contacts (Figure 5). The front view normal to (010) revealed that the molecules of *N*-(adamantane-1-yl)-carboxamide **2r** were arranged side-by-side and aligned in chains via N–H···O hydrogen bonds (d_(C=O⋯H-N)_ ≈ 2.42 Å, Table 4) along the *a*-axis.

Interestingly, it was found that the amide oxygen atom was involved in the following weak polar interaction: C;_6′_–H;_6′_···O_11_ (d_(C6′—H6′···O11)_ ≈ 2.71 Å), which was probably caused by the geometric criteria of the E-conformation of the amide bond. The structure was additionally stabilized by possible C–H;···π contacts in the crystal (Table 4), which set out “*edge-to-face̕*” stackings between the *p*-methoxyphenyl π system and the H6 hydrogen atom of the flavone ring (d_(C6—H6···π)_ ≈ 2.71 Å), but there was no evidence of “*face-to-face*” π···π stacking interactions.

**Figure 6:**
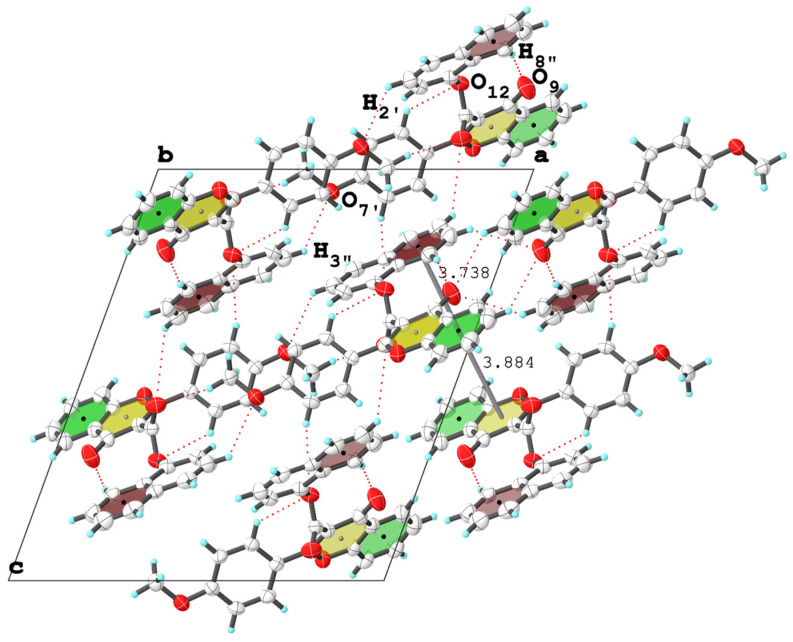
ORTEP plot of fragments of crystal packings [view normal to (010)] of naphthalen-1-yl 2-(4-methoxyphenyl)-4-oxo-4*H*-chromene-3-carboxylate **4f′**, showing thermal displacement ellipsoids, drawn at the 50% probability level (graphic designed using the Olex2 program). The red dashed lines highlight the inter/intramolecular hydrogen bonds and weak polar interactions. The gray solid lines indicate the probable *face-to-face* (π···π) aromatic stackings. The indicated values show the interplanar distances (Å).

Conversely, the top view normal to (010) of supramolecular structure **4f′** unveiled perfect tapes of flavones, and inversion-related molecules were connected primarily via π···π interactions with an interplanar distance (*centroid-to-centroid*) of 3.89 Å which probably enabled “*face-to-face*” π···π stackings between the aromatic platforms. Moreover, we found that naphthyl rings were arranged parallel relative to the flavone moiety and distanced by 3.74 Å, which was ideal for parallel-displaced π···π interactions (Figure 6, Table 4). Furthermore, the non-planar molecules of flavone-3-esters **4f′** were possibly involved in a set of intramolecular hydrogen bonds and weak interactions that stabilized the final crystal packing of **4f′** (Figure 6). Nevertheless, these structural features are not yet confirmed.

The analysis of the supramolecular structure revealed that the molecules **4f′** were linked by bifurcated interactions forming continued chains along the *a-, b-,* and *c*-axes (Figure 6). It is noteworthy that each molecule was involved in C2′–H2′···O12 and C8”–H8”···O9 intramolecular interactions (d_(C2′—H2′···O12)_ ≈ 2.86 Å, and d_(C8”—H8”···O9)_ ≈ 2.65 Å), which could explain the non-planar geometry of the flavone-3-ester.

## 4. Materials and Methods

### 4.1. General Procedures

The Pd(OAc)_2_, the ligands—PPh_3_ (triphenylphosphine), dppf (1,1′-bis(diphenylphosphino)ferrocene), dppp (1,3-bis(diphenylphosphino)propane), and XantPhos (4,5-bis(diphenylphosphino)-9,9-dimethylxanthene)—the bases (KOH, Et_3_N, and K_2_CO_3_), 2-hydroxyacetophenone, 4-anisaldehyde, ceric ammonium nitrate (CAN), iodine (I_2_), all the solvents, amines (**a-t**), and phenols (**a′-j′**) were purchased from Sigma-Aldrich (St. Louis, MO, USA) and used without any further purification. Precoated silica gel 60F_254_ plates were used for thin-layer chromatography (TLC) and also purchased from Sigma-Aldrich. Column chromatography was performed with 0.063–0.200 mm mesh silica gels. The ^1^H and ^13^C NMR spectra were recorded in CDCl_3_ or DMSO-d_6_ on a Bruker Avance III 500 spectrometer (Bruker BioSpin Corp., Karlsruhe, Germany) at 500 and 125 MHz, respectively. Chemical shifts δ were reported in ppm relative to CDCl_3_ (7.26 and 77.00 ppm for ^1^H and ^13^C, respectively) or DMSO-d_6_ (2.50 and 39.50 ppm for ^1^H and ^13^C, respectively). The copies of NMR spectra are given in the Supplementary file (Figures S3–S74). A Nicolet IMPACT 400 spectrometer (Thermo Fisher Scientific, Waltham, MA, USA) was applied to record the FT-IR spectra in KBr pellets using a DTGS detector in the region of 400–4000 cm^−1^, with a resolution of 4 cm^−1^.

The samples were analyzed with a Shimadzu GC-2030 gas-chromatograph (Shimadzu, Tokyo, Japan) fitted with a capillary column (DB-1) (injector temp. 250 °C; oven starting temp. 50 °C (hold-time 1 min), heating rate 15 °C min^−1^, and final temp. 320 °C (hold-time 11 min); detector temp. 280 °C; and carrier gas helium (rate: 1 mL min^−1^)).

The electron ionization–mass spectrometry (EI-MS) data were acquired using a GC–MS-2020 system (Shimadzu, Tokyo, Japan). The EI ion source was operated at 70 eV. The EI-MS results of the compounds are included in Supplementary File (Pages S24–S40) and presented as the mass-to-charge ratio (m/z) and relative intensities (%) in brackets. Additionally, the high-resolution mass spectrometry (HRMS) data were obtained using a 6530 Accurate-Mass Quadrupole Time-of-Flight (Q-TOF) LC/MS system (Agilent Technologies, Singapore). The compounds were ionized by an Agilent Jet Stream electrospray ion source.

The starting 3-iodoflavone **1** was synthesized according to the reported three-step protocol [44,45,46], as described below. The isolated *N*-substituted β-enaminones **3f** [47,48] and **3h** [43] had been previously described. These derivatives were also characterized, and their spectroscopic data showed good agreement with those published in the literature.

### 4.2. Synthesis of 2-(4′-Methoxyphenyl)-3-Iodo-4H-Chromen-4-One **1**

- Step 1: Claisen–Schmidt Condensation [44]

A mixture of 2-hydroxyacetophenone (5.44 g, 40 mmol) and 4-anisaldehyde (5.44 g, 40 mmol) was stirred in 50 mL of ethanol at 0 °C for 10 min. Then, an ethanolic potassium hydroxide solution (6.72 g, 120 mmol in 40 mL absolute ethanol) was added dropwise. Meanwhile, the temperature was maintained under 10 °C. The obtained orange cake was kept for two days at room temperature and occasionally shaken. The reaction mixture was poured into crushed ice, and the excess potassium hydroxide was neutralized with a hydrochloride solution (6N). The yellow chalcone precipitated was filtered off, washed with water, dried in air, and then recrystallized from ethanol; light yellow crystals were obtained as ((E)-1-(2′-hydroxyphenyl)-3-(4′-methoxyphenyl)prop-2-en-1-one) in an 85% yield.

- Step 2: I2/DMSO-mediated Oxidative Cyclization [45]

To a solution of chalcone (8.6 g, 33.8 mmol) in DMSO (25 mL) was added a catalytic amount of iodine (0.859 g, 0.338 mmol), and the reaction mixture was heated in an oil bath at 130 °C for 60 min. After cooling, iodine was removed by washing with a saturated solution of sodium thiosulphate and water. The flavone was then extracted with CHCl_3_ and purified by recrystallization in methanol in a 95% yield.

- Step 3: I2/CAN-promoted Iodination [46]

A solution of 2-(4′-methoxyphenyl)-*4H*-chromen-4-one (8 g, 31.7 mmol), I_2_ (9.67 g, 38.1 mmol), and (20.28 g, 38 mmol) of CAN (ceric ammonium nitrate) ([(NH_4_)_2_Ce^(IV)^(NO_3_)_6_]) in 80 mL of freshly distilled acetonitrile (CH_3_CN) was stirred at 65 °C (oil bath) under an argon atmosphere until the disappearance of the substrate (TLC). After being cooled to ambient temperature, the reaction mixture was poured into 30 mL of cold saturated solution of sodium thiosulfate (Na_2_S_2_O_3_) and extracted three times with 50 mL of CHCl_3_. The organic layers were combined, washed with brine solution, dried on magnesium sulfate (MgSO_4_), and concentrated. The residue was purified by column chromatography (*n*-Hexane: EtOAc) to afford the desired 2-(4′-methoxyphenyl)-3-iodo-4*H*-chromen-4-one **1** (78%) as a white solid.

### 4.3. General Method for the Synthesis of Flavone-3-Carboxamides 2 and Flavone-3-Esters 4 under Atmospheric Conditions

In a typical experiment, Pd(OAc)_2_ (2.8 mg, 0.0125 mmol), XantPhos (7.2 mg, 0.0125 mmol), 2-(4′-methoxyphenyl)-3-iodo-4*H*-chromen-4-one **1** substrate (0.5 mmol, 188 mg), *N*-nucleophiles ((**a**-**t**): 0.55 mmol of solid amines or 0.75 mmol of liquid amines) or *O*- nucleophiles ((**a**′-**j**′): 0.55 mmol of solid phenols or 0.75 mmol of liquid phenols), and potassium carbonate (104 mg, 0.75 mmol) were dissolved in DMF (5 mL) under argon in a 100 mL three-necked flask equipped with a reflux condenser connected to a balloon filled with argon. The reaction vessel was flushed with argon. The atmosphere was then changed to carbon monoxide (caution: a carbon monoxide atmosphere should only be used with adequate ventilation (hood), using CO sensors as well). The reaction was conducted for the given reaction time upon stirring at 50 and 80 °C using a heat-on block, and it was analyzed by GC and GC-MS. The cooled reaction mixture was then concentrated and evaporated to dryness under a reduced pressure. The residue was dissolved in chloroform (20 mL) and washed twice with water (20 mL). The organic phase was dried over Na_2_SO_4_, filtered, and evaporated under a reduced pressure to generate a viscous material. All compounds were subjected to column chromatography (silica gel 60 (Sigma), 0.063–0.200 mm), using different eluent mixtures (the exact eluents (content and ratio) are specified under the Characterization Section found in Supplementary File (S24–S40)).

### 4.4. General Method of High-Pressure Aminocarbonylation Reactions

The Pd(OAc)_2_ (5.6 mg, 0.025 mmol), PPh_3_ (13.1 mg, 0.05 mmol), 2-(4′-methoxyphenyl)-3-iodo-4*H*-chromen-4-one **1** substrate (0.5 mmol, 189 mg), L-alanine methyl ester hydrochloride (**a**) (0.55 mmol, 77 mg), and triethylamine (0.5 mL) were dissolved in DMF (10 mL) under argon in a 100 mL stainless steel autoclave. The atmosphere was changed to carbon monoxide, and the autoclave was pressurized to 40 bar with carbon monoxide. (caution: high-pressure carbon monoxide should only be used with adequate ventilation (hood), using CO sensors as well). The reaction was stirred for a 72 h reaction time in an oil bath at 50–80 °C. After the given reaction time the reaction mixture was cooled down to room temperature, and the autoclave was carefully depressurized under a well-ventilated hood. The product mixture was filtered and analyzed using GC and GC-MS measurements.

## 5. Conclusions

In this work, we have disclosed an easy and versatile methodology for the construction of C-3 functionalized flavones. Several new flavone-based carboxamides and esters were prepared in moderate-to-excellent yields via chemoselective palladium-catalyzed amino- and aryloxycarbonylation reactions using a panoply of amines and phenols. To the best of our knowledge, this practical carbonylative approach involving Pd(OAc)_2_/XantPhos as a catalyst, under mild conditions, has been first reported in this paper as a means to install new functionalities such as carboxamide and ester groups at the C-3 position in the flavone’s skeleton. The reaction conditions look promising for application in many approved 3-unsubstituted flavone-containing derivatives to design new flavone hybrids with potential applications in the pharmaceutical field. Furthermore, the crystal structures of the new flavone-3-carboxamide and flavone-3-ester were undoubtedly established and studied by single-crystal XRD analysis.

## Figures and Tables

**Table 1:** Optimization study of the aminocarbonylation of 3-iodoflavone **1** with L-alanine methyl ester **a** ^(a)^.

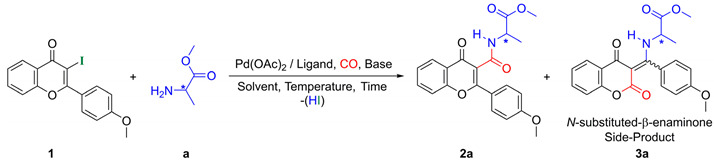									
Entry	Base	Ligand	Solvent	Temp. [°C]	P(CO) [bar]	Time [h]	Conv.	Ratio ^(b)^ (2a/3a)	Yield ^(c)^ (2a/3a)
1	Et_3_N	PPh_3_	DMF	50	1	72	50	100/0	15/0
2	Et_3_N	PPh_3_	DMF	80	1	72	100	81/19	42/10
3	Et_3_N	PPh_3_	DMF	50	40	72	20	100/0	5/0
4	Et_3_N	PPh_3_	DMF	80	40	72	85	100/0	25/0
5	Et_3_N	dppf	DMF	80	1	24	100	100/0	49/0
6	Et_3_N	dppp	DMF	80	1	72	80	100/0	30/0
7	Et_3_N	XantPhos	DMF	80	1	12	100	78/22	56/16
8	Et_3_N	XantPhos	Toluene	80	1	72	100	100/0	40/0
9	Et_3_N	XantPhos	ACN	80	1	48	100	75/25	55/18
10	Et_3_N	XantPhos	Dioxane	80	1	72	80	100/0	32/0
11	K_2_CO_3_	XantPhos	DMF	80	1	12	100	96/4	72/3
**12**	**K_2_CO_3_**	**XantPhos**	**DMF**	**50**	**1**	**24**	**100**	**95/5**	**79/4**

^(a)^ Standard reaction conditions: 0.5 mmol of 3-iodo-2-(4′-methoxyphenyl)-4*H*-chromen-4-one **1**, 0.55 mmol of L-alanine methyl ester hydrochloride (**a**), 0.0125 mmol of Pd(OAc)_2_, 0.025 mmol of monodentate (PPh_3_) or 0.0125 mmol of bidentate (XantPhos, dppp, dppf) ligands, 0.5 mL of Et_3_N, 104 mg of K_2_CO_3_, and 5 mL of dry solvents—dimethylformamide (DMF), toluene, 1,4-dioxane, or acetonitrile (ACN)—at the mentioned temperature under carbon monoxide pressure (1 or 40 bar). The reaction mixture was monitored by GC-MS and ^1^H NMR measurements. The conversion was achieved after the given reaction time. ^(b)^ The ratio was calculated based on the ^1^H NMR and GC-MS measurements of crude reactions and the amounts of isolated products, and the average is given in the table above. ^(c)^ Isolated yield based on the amount of substrate.

**Table 2:** Scope of primary and secondary amines (**a**-**t**) ^(a)^.

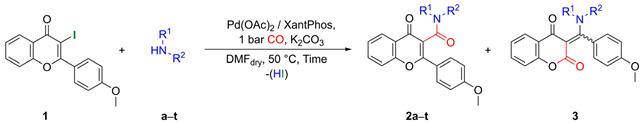				
Entry	Amines	Time [h]	Flavone-3-carboxamides (2) *N*-Substituted β-Enaminones (3)	
			Ratio ^(b)^ (2/3)	Yield ^(c)^ (2/3)
**1**	L-Alanine methyl ester (**a**)	24	95/5	79/4
**2**	L-Valine methyl ester (**b**)	24	90/10	70/7
**3**	(*S*)-2-Phenylglycine methyl ester (**c**)	24	92/8	53/5 ^(d)^
**4**	L-Phenylalanine methyl ester (**d**)	24	96/4	68/3 ^(d)^
**5**	L-Serine methyl ester (**e**)	24	100/0	46/0
**6**	Aniline (**f**)	24	24/76	22/70
**7**	4-Picolylamine (**g**)	48	86/14	36/6 ^(d)^
**8**	Benzylamine (**h**)	48	55/45	44/37
**9**	Phenethylamine (**i**)	48	60/40	41/28
**10**	Piperonylamine (**j**)	48	75/25	45/15
**11**	*N,O*-dimethylhydroxylamine (**k**)	48	95/5	71/4 ^(d)^
**12**	Diethylamine (**l**)	24	95/5	57/3 ^(d)^
**13**	Morpholine (**m**)	24	100/0	48/0
**14**	Pyrrolidine (**n**)	24	100/0	77/0
**15**	*N*-Methylpiperazine (**o**)	24	100/0	41/0
**16**	Piperidine (**p**)	48	100/0	67/0
**17**	*N*-Ethyl-4-picolylamine (**q**)	24	100/0	54/0
**18**	1-Adamantylamine (**r**)	72	100/0	46/0
**19**	Nortropine (**s**)	48	100/0	70/0
**20**	Nortropinone (**t**)	24	100/0	51/0

^(a)^ Experimental protocol: 0.5 mmol of 3-iodoflavone **1**, primary and secondary amine nucleophile, including 0.55 mmol of solid amines (or 0.75 mmol of liquid amines), 0.0125 mmol of Pd(OAc)_2_, 0.0125 mmol of XantPhos, 0.75 mmol of K_2_CO_3_, and 5 mL of DMF, at 50 °C, under 1 bar of carbon monoxide, for 24–72 h. ^(b)^ The ratio was determined based on the amounts of isolated products, supported by ^1^H NMR and GC-MS measurements of the crude reaction, and the average is given in the table above. ^(c)^ Isolated yield based on the amount of substrate. ^(d)^ The compound was isolated in an impure form.

**Table 3:** Crystallographic parameters and refinement details for **2r** and **4f’**.

Compounds	2r	4f′
Chemical formula	C_27_H_27_NO_4_	C_27_H_18_NO_5_
M_r_	429.49	422.41
Crystal system	Orthorhombic	Monoclinic
Space group	P2_1_2_1_2_1_	P2_1_/c
Temperature (K)	293	293
a, b, c (Å)	9.898 (5), 11.606 (7), 18.521 (11)	15.086 (17), 8.042 (8), 17.586 (16)
α, β, γ (°)	90, 90, 90	90, 110.03 (3), 90
V (Å^3^)	2128 (2)	2005 (4)
Z	4	4
D (g cm^−3^)	1.341	1.482
Radiation type	Mo Kα	
μ (mm^−1^)	0.09	0.10
Crystal size (mm)	0.70 × 0.09 × 0.07	0.24 × 0.17 × 0.10
	**Data collection**	
Diffractometer	Bruker D8 VENTURE	
Absorption correction	Multi-scan SADABS2016/2—Bruker AXS area detector scaling and absorption correction	
T_min_, T_max_	0.82, 0.99	0.83, 0.99
No. of measured, independent and observed [I > 2σ(I)] reflections	10,530, 4171, 1812	24,369, 3653, 2407
R_int_	0.179	0.095
(sin θ/λ)_max_ (Å^−1^)	0.620	0.603
Refinement		
R [F^2^ > 2s(F^2^)], wR(F^2^), S	0.078, 0.175, 0.99	0.051, 0.142, 1.09
No. of reflections	4171	3653
No. of parameters	294	291
H-atom treatment	H atoms are treated by a mixture of independent and constrained refinement	
Δ > _max_, Δ > _min_ (e Å^−3^)	0.22, −0.21	0.26, −0.25

**Table 4:** Geometric parameters for some possible inter/intramolecular hydrogen bonds and short interactions (Å, °) for **2r** and **4f**′.

Crystals	Inter/Intramolecular Hydrogen Bonds and				Short Interactions (Å)		
	A···H—D	D—H	H···A	D···A	∠D—H···A	π···π	CH···π
**(2r)**	N12—H12···O11 ^i^	0.850	2.421	3.241	161.03	-	2.907
	C6′—H6′···O11 ^ii^	0.930	2.713	3.417	133.13		
	C5′—H5′ ^i^ ···O9 ^ii^	0.930	2.898	3.599	133.21		
**(4f′** **)**	C2′—H2′···O12 ^i^	0.930	2.867	3.556	132.93	3.74–3.89	-
	C8”—H8”···O9 ^ii^	0.930	2.650	3.544	161.68		
	C3”—H3” ^ii^···O7′ ^i^	0.930	2.766	3.544	121.21		

Symmetry codes: **2r** [(^i^) 1/2 + *x*, −*y* + 3/2, −*z* + 1; (^ii^) 1 − x, +y, +z]; **4f**′ [(i) + x, 3/2 − y, −1/2 + z; (^ii^) 1 − x, 1/2 + y, 1/2 − z].

## Data Availability

The data presented in this study are available in Supplementary Files.

## Supplementary Materials

The following supporting information can be downloaded at https://www.mdpi.com/article/10.3390/ijms251810128/s1. References [49,50,51,52] are cited in the Supplementary Material.
