# Supplementary material for: A Facile Route to Flavone-3-Carboxamides and Flavone-3-Carboxylates via Palladium-Catalyzed Amino- and Aryloxy-Carbonylation Reactions
Source: Int J Mol Sci. 2024 Sep 20;25(18):10128. doi: 10.3390/ijms251810128 (PMC11432944; doi:10.3390/ijms251810128)
Supplement: Supplementary file 1 [file ijms-25-10128-s001.zip › ijms-3198168-supplementary.pdf]

Supporting Information

A Facile Route to Flavone-3-Carboxamides and Flavone-3-Carboxylates *via* Palladium-Catalyzed Amino- and Aryloxycarbonylation Reactions

Sami Chniti[1], László Kollár,[1,2,3] Attila Bényei,[4] Ágnes Dörnyei,[5] Attila Takács[2,3*]

[1] Department of General and Inorganic Chemistry, University of Pécs, Ifjúság u. 6., Pécs, H-7624, Hungary.

[2] HUN-REN-PTE Research Group for Selective Chemical Syntheses, Ifjúság u. 6., Pécs, H-7624, Hungary.

[3] János Szentágothai Research Centre, University of Pécs, Ifjúság u. 20., Pécs, H-7624, Hungary.

[4] Department of Physical Chemistry, University of Debrecen, Egyetem tér 1., Debrecen, H-4032, Hungary.

[5] Department of Analytical and Environmental Chemistry, University of Pécs, Ifjúság u. 6., Pécs, H-7624, Hungary.

*e-mail Attila Takács: takacsattila@gamma.ttk.pte.hu

Table of Contents

1. X-ray Crystallography S1.

2. Characterization of the synthesized compounds S24.

3. Copies of the ^{1}H and $^{13}C\{^{1}H\}$ NMR spectra of the synthesized compounds S41.

1. X-ray Crystallography

Computing details

X-ray quality crystals of both compounds **2r** and **4f′** were grown from the slow evaporation of concentrated solutions of ethyl acetate/dichloromethane and n-hexane/chloroform/ethyl acetate, respectively, upon storing at 4–5 °C or standing at ambient temperature. A properly chosen suitable colorless needle was, then, fixed under a microscope onto a Mitegen loop using high-density oil. Diffraction Intensity data was collected at ambient temperature (294 K) on a Bruker-D8 Venture diffractometer (Bruker AXS GmbH, Karlsruhe, Germany) equipped with INCOATEC IμS 3.0 (Incoatec GmbH, Geesthacht, Germany) dual (Cu and Mo) sealed tube micro sources and a Photon II Charge-Integrating Pixel Array detector (Bruker AXS GmbH, Karlsruhe, Germany) using Mo Kα (λ = 0.71073 Å) radiation.

High-multiplicity data collection and integration were performed using APEX3 (ver-sion 2017.3-0, Bruker AXS Inc., 2017, Madison, WI, USA) software. Data reduction and multiscan absorption correction were performed using SAINT (version 8.38A, Bruker AXS Inc., 2017, Madison, WI, USA). The structure was solved using direct methods and refined on F2 using the SHELXL program incorporated into the APEX3 suite [49]. Refinement was performed anisotropically for all non-hydrogen atoms. Hydrogen atoms were placed in idealized positions on parent atoms in the final refinement.

The CIF file was manually merged using publCIF software [50], while graphics were designed using the Olex2 program [51]. The results for the X-ray diffraction structure de-terminations followed the Checkcif functionality of PLATON software (Utrecht University, Utrecht, the Netherlands) [52], and structural parameters, such as bond length and angle data, are in the expected range.

X-ray Crystallographic Data of compound 2r

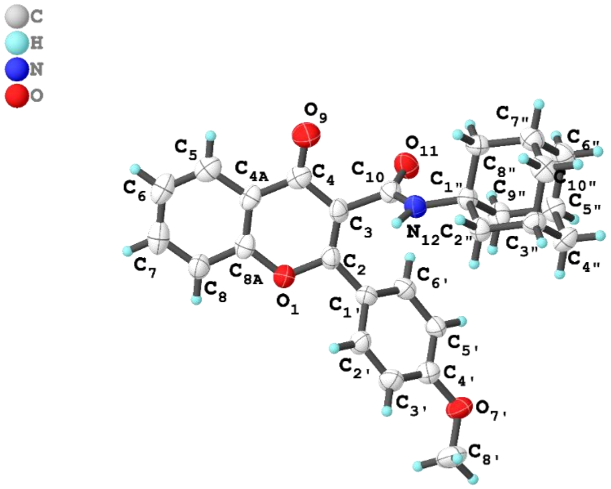

Figure S1. ORTEP diagram of solid-state structure of *N*-(adamantan-1-yl)-2-(4-methoxyphenyl)-4-oxo-4*H*-chromene-3-carboxamide **2r**, showing thermal displacement ellipsoids, drawn at the 50% probability level.

Crystal data

$C_{27}H_{27}NO_4$	D_x = 1.341 Mg m^{-3}
M_r = 429.49	Mo *K*a radiation, l = 0.71073 Å
Orthorhombic, $P2_12_12_1$	Cell parameters from 601 reflections
a = 9.898 (5) Å	q = 2.8–22.2°
b = 11.606 (7) Å	m = 0.09 mm^{-1}
c = 18.521 (11) Å	*T* = 293 K
V = 2128 (2) Å^3	Needle, colorless
Z = 4	0.70 × 0.09 × 0.07 mm
F(000) = 912	

Data collection

Bruker D8 VENTURE diffractometer	4171 independent reflections
Radiation source: microfocus sealed tube, INCOATEC ImS 3.0	1812 reflections with *I* > 2s(*I*)
Multilayer mirror monochromator	R_{int} = 0.179
Detector resolution: 7.3910 pixels mm^{-1}	q_{max} = 26.2°, q_{min} = 2.1°
w and p scan	*h* = -12→9

Absorption correction: multi-scan *SADABS2016*/2 - Bruker AXS area detector scaling and absorption correction	$k = -14 \rightarrow 14$
$T_{min} = 0.82$, $T_{max} = 0.99$	$l = -21 \rightarrow 22$
10530 measured reflections	

Refinement

Refinement on F^2	Hydrogen site location: mixed
Least-squares matrix: full	H atoms treated by a mixture of independent and constrained refinement
$R[F^2 > 2s(F^2)] = 0.078$	$w = 1/[s^2(F_o^2) + (0.0196P)^2]$ where $P = (F_o^2 + 2F_c^2)/3$
$wR(F^2) = 0.175$	$(D/s)_{max} < 0.001$
$S = 0.99$	$D\rho_{max} = 0.22$ e Å^{-3}
4171 reflections	$D\rho_{min} = -0.21$ e Å^{-3}
294 parameters	Extinction correction: *SHELXL2019*/1 (Sheldrick 2019)
1 restraint	Extinction coefficient: 0.0066 (16)
Primary atom site location: structure-invariant direct methods	Absolute structure: Flack x determined using 423 quotients [(I+)-(I-)]/[(I+)+(I-)] (Parsons, Flack and Wagner, Acta Cryst. B69 (2013) 249-259).
Secondary atom site location: difference Fourier map	Absolute structure parameter: -3.4 (10)

Special details

Geometry. All esds (except the esd in the dihedral angle between two l.s. planes) are estimated using the full covariance matrix. The cell esds are taken into account individually in the estimation of esds in distances, angles and torsion angles; correlations between esds in cell parameters are only used when they are defined by crystal symmetry. An approximate (isotropic) treatment of cell esds is used for estimating esds involving l.s. planes.

*Fractional atomic coordinates and isotropic or equivalent isotropic displacement parameters (Å²) for (**2r**)*

	x	*y*	*z*	U_{iso}*/U_{eq}
C1''	0.6287 (8)	0.7343 (7)	0.3690 (4)	0.0364 (19)
C1'	0.5389 (8)	0.5218 (7)	0.5545 (4)	0.040 (2)
C2	0.5690 (8)	0.6266 (7)	0.5951 (4)	0.041 (2)
C2''	0.7549 (8)	0.6741 (8)	0.3411 (4)	0.041 (2)
H2''A	0.756465	0.595256	0.358476	0.050000*
H2''B	0.834497	0.713056	0.359447	0.050000*
C2'	0.6165 (10)	0.4237 (7)	0.5650 (4)	0.048 (2)
H2'	0.690631	0.427547	0.595716	0.058000*
C3	0.5722 (8)	0.7352 (8)	0.5710 (4)	0.040 (2)
C3''	0.7576 (8)	0.6745 (7)	0.2585 (4)	0.043 (2)
H3''	0.839790	0.636263	0.241366	0.052000*
C3'	0.5876 (9)	0.3214 (7)	0.5315 (5)	0.053 (2)
H3'	0.643447	0.257736	0.537767	0.064000*
C4	0.6057 (8)	0.8294 (8)	0.6188 (5)	0.046 (2)
C4''	0.6343 (9)	0.6102 (7)	0.2311 (5)	0.051 (2)
H4''A	0.634436	0.609301	0.178725	0.061000*
H4''B	0.636479	0.531191	0.248051	0.061000*
C4'	0.4740 (9)	0.3144 (8)	0.4882 (5)	0.045 (2)
C5	0.6437 (9)	0.8805 (8)	0.7483 (5)	0.053 (3)
H5	0.648014	0.958195	0.736098	0.064000*
C4A	0.6235 (8)	0.7984 (7)	0.6940 (5)	0.041 (2)
C5''	0.5076 (8)	0.6692 (7)	0.2583 (4)	0.045 (2)
H5''	0.427942	0.628115	0.240248	0.053000*
C5'	0.3968 (9)	0.4105 (7)	0.4764 (4)	0.043 (2)
H5'	0.322519	0.406206	0.445837	0.052000*
C6	0.6572 (9)	0.8484 (9)	0.8184 (5)	0.058 (3)
H6	0.668868	0.904133	0.853970	0.070000*
C6''	0.5052 (9)	0.7914 (7)	0.2312 (5)	0.052 (3)

H6''A	0.423710	0.829405	0.247885	0.062000*
H6''B	0.504604	0.791642	0.178848	0.062000*
C6'	0.4282 (8)	0.5133 (7)	0.5094 (4)	0.044 (2)
H6'	0.374327	0.577656	0.501212	0.053000*
C7	0.6537 (9)	0.7342 (9)	0.8369 (5)	0.060 (3)
H7	0.665586	0.712670	0.884820	0.072000*
C7''	0.6281 (9)	0.8560 (7)	0.2585 (4)	0.047 (2)
H7''	0.625257	0.935457	0.240572	0.057000*
C8	0.6330 (9)	0.6515 (7)	0.7856 (4)	0.049 (2)
H8	0.628510	0.574129	0.798345	0.058000*
C8''	0.6272 (10)	0.8571 (6)	0.3412 (4)	0.043 (2)
H8''A	0.705886	0.898070	0.358998	0.051000*
H8''B	0.547089	0.896463	0.358535	0.051000*
C8'	0.5044 (12)	0.1128 (7)	0.4698 (6)	0.077 (3)
H8'A	0.458243	0.048545	0.448454	0.116000*
H8'B	0.511227	0.101420	0.521012	0.116000*
H8'C	0.593382	0.118964	0.449488	0.116000*
C8A	0.6187 (8)	0.6852 (7)	0.7143 (4)	0.041 (2)
C9''	0.5059 (8)	0.6697 (8)	0.3411 (4)	0.046 (2)
H9''A	0.424052	0.706565	0.358297	0.055000*
H9''B	0.507087	0.591121	0.359023	0.055000*
C10	0.5402 (8)	0.7605 (7)	0.4939 (4)	0.041 (2)
C10''	0.7553 (8)	0.7986 (8)	0.2320 (5)	0.052 (2)
H10A	0.833754	0.839474	0.250106	0.062000*
H10B	0.758040	0.800142	0.179659	0.062000*
H12	0.706 (6)	0.707 (7)	0.471 (4)	0.078000*
O1	0.5944 (6)	0.5988 (5)	0.6660 (3)	0.0457 (16)
O7'	0.4306 (6)	0.2164 (5)	0.4556 (3)	0.0574 (17)
O9	0.6176 (8)	0.9290 (5)	0.5969 (3)	0.0679 (19)
O11	0.4306 (6)	0.8033 (5)	0.4776 (3)	0.0526 (17)
N12	0.6374 (7)	0.7315 (6)	0.4476 (4)	0.0415 (17)

*Atomic displacement parameters (Å^2) for (**2r**)*

	U^{11}	U^{22}	U^{33}	U^{12}	U^{13}	U^{23}
C1''	0.036 (4)	0.042 (5)	0.032 (5)	-0.001 (4)	0.006 (4)	0.000 (4)
C1'	0.043 (5)	0.037 (5)	0.039 (5)	0.007 (4)	-0.003 (4)	0.001 (4)
C2	0.041 (5)	0.048 (6)	0.034 (5)	0.004 (4)	-0.008 (4)	-0.004 (5)
C2''	0.037 (4)	0.049 (5)	0.038 (5)	0.004 (4)	-0.001 (4)	-0.001 (5)
C2'	0.048 (5)	0.049 (5)	0.048 (6)	0.013 (5)	-0.017 (5)	-0.011 (5)
C3	0.039 (5)	0.050 (6)	0.029 (5)	0.006 (4)	0.004 (4)	-0.002 (4)
C3''	0.040 (5)	0.044 (6)	0.046 (6)	0.008 (4)	0.002 (4)	-0.005 (5)
C3'	0.055 (6)	0.046 (5)	0.059 (6)	0.015 (5)	-0.016 (5)	-0.006 (5)
C4	0.039 (5)	0.044 (5)	0.055 (7)	0.000 (5)	-0.002 (4)	-0.005 (5)
C4''	0.051 (6)	0.046 (5)	0.055 (6)	0.002 (5)	0.001 (5)	-0.014 (5)
C4'	0.050 (5)	0.040 (5)	0.046 (6)	-0.002 (4)	-0.001 (4)	0.000 (5)
C5	0.044 (5)	0.053 (6)	0.063 (7)	-0.002 (5)	0.001 (5)	-0.009 (6)
C4A	0.033 (4)	0.047 (6)	0.043 (5)	-0.008 (4)	0.001 (4)	-0.010 (5)
C5''	0.030 (5)	0.054 (6)	0.050 (6)	-0.005 (5)	-0.004 (4)	0.001 (5)
C5'	0.050 (6)	0.039 (5)	0.041 (5)	-0.002 (4)	-0.013 (4)	0.004 (4)
C6	0.052 (6)	0.069 (7)	0.054 (7)	-0.010 (5)	0.004 (5)	-0.022 (6)
C6''	0.042 (5)	0.063 (7)	0.050 (6)	0.012 (5)	-0.006 (5)	0.007 (6)
C6'	0.045 (5)	0.042 (5)	0.045 (6)	0.007 (4)	-0.009 (4)	0.003 (5)
C7	0.048 (6)	0.090 (8)	0.042 (6)	-0.003 (6)	-0.002 (5)	-0.014 (6)
C7''	0.047 (5)	0.048 (6)	0.048 (6)	-0.003 (5)	-0.001 (5)	0.006 (5)
C8	0.055 (5)	0.052 (6)	0.039 (6)	0.005 (5)	-0.001 (5)	-0.006 (5)
C8''	0.047 (5)	0.034 (5)	0.046 (5)	-0.001 (4)	0.000 (5)	0.003 (4)
C8'	0.100 (8)	0.040 (6)	0.091 (9)	0.016 (6)	-0.022 (7)	-0.013 (6)
C8A	0.031 (5)	0.053 (6)	0.041 (6)	-0.004 (5)	-0.005 (4)	-0.009 (5)
C9''	0.041 (5)	0.053 (6)	0.043 (6)	-0.011 (5)	-0.002 (4)	0.001 (5)
C10	0.043 (5)	0.040 (5)	0.039 (6)	-0.003 (4)	-0.003 (4)	-0.001 (4)
C10''	0.039 (5)	0.061 (6)	0.055 (6)	-0.007 (5)	0.007 (5)	0.012 (6)
O1	0.053 (4)	0.043 (4)	0.041 (4)	0.001 (3)	-0.006 (3)	-0.003 (3)
O7'	0.068 (4)	0.037 (4)	0.067 (4)	0.000 (3)	-0.015 (4)	-0.008 (3)

O9	0.088 (5)	0.047 (4)	0.069 (5)	-0.010 (4)	-0.010 (4)	0.002 (4)
O11	0.049 (4)	0.062 (4)	0.047 (4)	0.016 (3)	0.000 (3)	0.003 (3)
N12	0.039 (4)	0.045 (4)	0.041 (4)	0.010 (4)	-0.001 (4)	0.000 (4)

*Geometric parameters (Å, º) for (**2r**)*

C1"—N12	1.460 (10)	C5"—C6"	1.504 (10)
C1"—C8"	1.514 (10)	C5"—C9"	1.535 (10)
C1"—C9"	1.519 (10)	C5"—H5"	0.9800
C1"—C2"	1.522 (10)	C5'—C6'	1.376 (10)
C1'—C6'	1.381 (11)	C5'—H5'	0.9300
C1'—C2'	1.387 (10)	C6—C7	1.370 (12)
C1'—C2	1.460 (11)	C6—H6	0.9300
C2—C3	1.338 (11)	C6"—C7"	1.516 (11)
C2—O1	1.375 (9)	C6"—H6"A	0.9700
C2"—C3"	1.530 (11)	C6"—H6"B	0.9700
C2"—H2"A	0.9700	C6'—H6'	0.9300
C2"—H2"B	0.9700	C7—C8	1.365 (11)
C2'—C3'	1.369 (10)	C7—H7	0.9300
C2'—H2'	0.9300	C7"—C10"	1.507 (11)
C3—C4	1.446 (11)	C7"—C8"	1.533 (11)
C3—C10	1.491 (11)	C7"—H7"	0.9800
C3"—C4"	1.518 (11)	C8—C8A	1.385 (11)
C3"—C10"	1.521 (11)	C8—H8	0.9300
C3"—H3"	0.9800	C8"—H8"A	0.9700
C3'—C4'	1.384 (11)	C8"—H8"B	0.9700
C3'—H3'	0.9300	C8'—O7'	1.432 (10)
C4—O9	1.231 (9)	C8'—H8'A	0.9600
C4—C4A	1.449 (11)	C8'—H8'B	0.9600
C4"—C5"	1.515 (11)	C8'—H8'C	0.9600
C4"—H4"A	0.9700	C8A—O1	1.366 (9)
C4"—H4"B	0.9700	C9"—H9"A	0.9700

C4'—O7'	1.358 (9)	C9"—H9"B	0.9700
C4'—C5'	1.369 (10)	C10—O11	1.231 (9)
C5—C6	1.358 (12)	C10—N12	1.332 (10)
C5—C4A	1.400 (11)	C10"—H10A	0.9700
C5—H5	0.9300	C10"—H10B	0.9700
C4A—C8A	1.367 (10)	N12—H12	0.85 (3)
N12—C1"—C8"	111.1 (7)	C5—C6—C7	120.1 (10)
N12—C1"—C9"	112.0 (7)	C5—C6—H6	119.9000
C8"—C1"—C9"	110.0 (7)	C7—C6—H6	119.9000
N12—C1"—C2"	106.2 (6)	C5"—C6"—C7"	110.1 (7)
C8"—C1"—C2"	108.9 (7)	C5"—C6"—H6"A	109.6000
C9"—C1"—C2"	108.3 (6)	C7"—C6"—H6"A	109.6000
C6'—C1'—C2'	117.7 (8)	C5"—C6"—H6"B	109.6000
C6'—C1'—C2	122.2 (7)	C7"—C6"—H6"B	109.6000
C2'—C1'—C2	119.9 (7)	H6"A—C6"—H6"B	108.2000
C3—C2—O1	122.4 (8)	C5'—C6'—C1'	120.6 (8)
C3—C2—C1'	128.1 (8)	C5'—C6'—H6'	119.7000
O1—C2—C1'	109.5 (7)	C1'—C6'—H6'	119.7000
C1"—C2"—C3"	110.6 (7)	C8—C7—C6	120.7 (9)
C1"—C2"—H2"A	109.5000	C8—C7—H7	119.7000
C3"—C2"—H2"A	109.5000	C6—C7—H7	119.7000
C1"—C2"—H2"B	109.5000	C10"—C7"—C6"	110.1 (7)
C3"—C2"—H2"B	109.5000	C10"—C7"—C8"	109.5 (8)
H2"A—C2"—H2"B	108.1000	C6"—C7"—C8"	109.4 (8)
C3'—C2'—C1'	122.2 (8)	C10"—C7"—H7"	109.3000
C3'—C2'—H2'	118.9000	C6"—C7"—H7"	109.3000
C1'—C2'—H2'	118.9000	C8"—C7"—H7"	109.3000
C2—C3—C4	120.9 (8)	C7—C8—C8A	118.7 (9)
C2—C3—C10	120.0 (8)	C7—C8—H8	120.6000
C4—C3—C10	119.1 (8)	C8A—C8—H8	120.6000

C4"—C3"—C10"	110.2 (7)	C1"—C8"—C7"	109.4 (7)
C4"—C3"—C2"	108.6 (7)	C1"—C8"—H8"A	109.8000
C10"—C3"—C2"	109.0 (7)	C7"—C8"—H8"A	109.8000
C4"—C3"—H3"	109.7000	C1"—C8"—H8"B	109.8000
C10"—C3"—H3"	109.7000	C7"—C8"—H8"B	109.8000
C2"—C3"—H3"	109.7000	H8"A—C8"—H8"B	108.2000
C2'—C3'—C4'	118.9 (8)	O7'—C8'—H8'A	109.5000
C2'—C3'—H3'	120.6000	O7'—C8'—H8'B	109.5000
C4'—C3'—H3'	120.6000	H8'A—C8'—H8'B	109.5000
O9—C4—C3	122.0 (8)	O7'—C8'—H8'C	109.5000
O9—C4—C4A	122.6 (8)	H8'A—C8'—H8'C	109.5000
C3—C4—C4A	115.4 (8)	H8'B—C8'—H8'C	109.5000
C5"—C4"—C3"	109.4 (7)	O1—C8A—C4A	122.1 (7)
C5"—C4"—H4"A	109.8000	O1—C8A—C8	115.8 (8)
C3"—C4"—H4"A	109.8000	C4A—C8A—C8	122.0 (8)
C5"—C4"—H4"B	109.8000	C1"—C9"—C5"	109.4 (7)
C3"—C4"—H4"B	109.8000	C1"—C9"—H9"A	109.8000
H4"A—C4"—H4"B	108.2000	C5"—C9"—H9"A	109.8000
O7'—C4'—C5'	115.8 (7)	C1"—C9"—H9"B	109.8000
O7'—C4'—C3'	124.4 (8)	C5"—C9"—H9"B	109.8000
C5'—C4'—C3'	119.9 (8)	H9"A—C9"—H9"B	108.2000
C6—C5—C4A	120.9 (9)	O11—C10—N12	125.5 (8)
C6—C5—H5	119.5000	O11—C10—C3	120.1 (8)
C4A—C5—H5	119.5000	N12—C10—C3	114.4 (7)
C8A—C4A—C5	117.5 (8)	C7"—C10"—C3"	109.0 (7)
C8A—C4A—C4	119.9 (8)	C7"—C10"—H10A	109.9000
C5—C4A—C4	122.5 (8)	C3"—C10"—H10A	109.9000

C6"—C5"—C4"	109.2 (7)	C7"—C10"—H10B	109.9000
C6"—C5"—C9"	109.2 (7)	C3"—C10"—H10B	109.9000
C4"—C5"—C9"	110.0 (8)	H10A—C10"—H10B	108.3000
C6"—C5"—H5"	109.5000	C8A—O1—C2	119.0 (6)
C4"—C5"—H5"	109.5000	C4'—O7'—C8'	117.4 (7)
C9"—C5"—H5"	109.5000	C10—N12—C1"	126.4 (7)
C4'—C5'—C6'	120.7 (8)	C10—N12—H12	109 (6)
C4'—C5'—H5'	119.7000	C1"—N12—H12	124 (6)
C6'—C5'—H5'	119.7000		
C6'—C1'—C2—C3	-48.1 (13)	C5—C6—C7—C8	-1.8 (15)
C2'—C1'—C2—C3	137.0 (9)	C5"—C6"—C7"—C10"	60.1 (9)
C6'—C1'—C2—O1	131.4 (8)	C5"—C6"—C7"—C8"	-60.3 (9)
C2'—C1'—C2—O1	-43.4 (11)	C6—C7—C8—C8A	1.5 (14)
N12—C1"—C2"—C3"	-178.8 (7)	N12—C1"—C8"—C7"	176.0 (7)
C8"—C1"—C2"—C3"	-59.0 (10)	C9"—C1"—C8"—C7"	-59.3 (10)
C9"—C1"—C2"—C3"	60.7 (9)	C2"—C1"—C8"—C7"	59.3 (10)
C6'—C1'—C2'—C3'	1.0 (14)	C10"—C7"—C8"—C1"	-61.5 (10)
C2—C1'—C2'—C3'	176.0 (9)	C6"—C7"—C8"—C1"	59.2 (10)
O1—C2—C3—C4	1.2 (13)	C5—C4A—C8A—O1	177.5 (8)
C1'—C2—C3—C4	-179.3 (8)	C4—C4A—C8A—O1	-1.5 (13)
O1—C2—C3—	-178.4 (7)	C5—C4A—	0.1 (13)

C10		C8A—C8	
C1'—C2—C3—C10	1.1 (13)	C4—C4A—C8A—C8	-178.9 (8)
C1''—C2''—C3''—C4''	-60.8 (9)	C7—C8—C8A—O1	-178.2 (7)
C1''—C2''—C3''—C10''	59.3 (10)	C7—C8—C8A—C4A	-0.6 (14)
C1'—C2'—C3'—C4'	-2.8 (15)	N12—C1''—C9''—C5''	-176.3 (7)
C2—C3—C4—O9	175.4 (9)	C8''—C1''—C9''—C5''	59.5 (9)
C10—C3—C4—O9	-5.0 (13)	C2''—C1''—C9''—C5''	-59.5 (9)
C2—C3—C4—C4A	-4.9 (12)	C6''—C5''—C9''—C1''	-59.6 (9)
C10—C3—C4—C4A	174.7 (7)	C4''—C5''—C9''—C1''	60.2 (9)
C10''—C3''—C4''—C5''	-59.6 (9)	C2—C3—C10—O11	104.7 (10)
C2''—C3''—C4''—C5''	59.7 (9)	C4—C3—C10—O11	-74.9 (10)
C2'—C3'—C4'—O7'	-176.1 (8)	C2—C3—C10—N12	-74.4 (10)
C2'—C3'—C4'—C5'	3.5 (14)	C4—C3—C10—N12	106.0 (9)
C6—C5—C4A—C8A	-0.4 (14)	C6''—C7''—C10''—C3''	-58.7 (9)
C6—C5—C4A—C4	178.6 (9)	C8''—C7''—C10''—C3''	61.6 (10)
O9—C4—C4A—C8A	-175.3 (9)	C4''—C3''—C10''—C7''	58.9 (9)
C3—C4—C4A—C8A	5.0 (12)	C2''—C3''—C10''—C7''	-60.2 (10)
O9—C4—C4A—C5	5.8 (14)	C4A—C8A—O1—C2	-2.5 (11)
C3—C4—C4A—	-173.9 (8)	C8—C8A—O1—	175.1 (7)

C5		C2	
C3''—C4''—C5''—C6''	59.8 (9)	C3—C2—O1—C8A	2.6 (11)
C3''—C4''—C5''—C9''	-60.1 (9)	C1'—C2—O1—C8A	-176.9 (6)
O7'—C4'—C5'—C6'	177.2 (8)	C5'—C4'—O7'—C8'	-176.7 (8)
C3'—C4'—C5'—C6'	-2.5 (14)	C3'—C4'—O7'—C8'	3.0 (13)
C4A—C5—C6—C7	1.2 (15)	O11—C10—N12—C1''	-6.1 (14)
C4''—C5''—C6''—C7''	-60.0 (10)	C3—C10—N12—C1''	172.9 (8)
C9''—C5''—C6''—C7''	60.3 (10)	C8''—C1''—N12—C10	71.3 (11)
C4'—C5'—C6'—C1'	0.6 (13)	C9''—C1''—N12—C10	-52.2 (11)
C2'—C1'—C6'—C5'	0.2 (13)	C2''—C1''—N12—C10	-170.4 (8)
C2—C1'—C6'—C5'	-174.8 (8)		

*Hydrogen-bond geometry (Å, º) for (**2r**)*

D—H···*A*	*D*—H	H···*A*	*D*···*A*	*D*—H···*A*
C9''—H9''*A*···O11	0.97	2.48	3.058 (10)	118
N12—H12···O11^{i}	0.85 (3)	2.42 (4)	3.241 (9)	161 (8)

Symmetry code: (i) *x*+1/2, -*y*+3/2, -*z*+1.

X-ray Crystallographic Data of compound 4f′

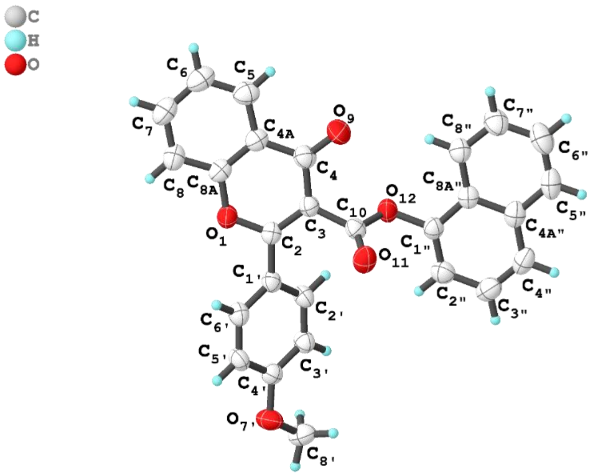

Figure S2. ORTEP diagram of solid-state structure of *N*-(adamantan-1-yl)-2-(4-methoxyphenyl)-4-oxo-4*H*-chromene-3-carboxamide **4f'**, showing thermal displacement ellipsoids, drawn at the 50% probability level.

Crystal data

$C_{27}H_{18}NO_5$	$F(000)$ = 880
M_r = 422.41	D_x = 1.400 Mg m^{-3}
Monoclinic, $P2_1/c$	Mo *K*a radiation, l = 0.71073 Å
a = 15.086 (17) Å	Cell parameters from 3228 reflections
b = 8.042 (8) Å	q = 2.8–25.0°
c = 17.586 (16) Å	m = 0.10 mm^{-1}
b = 110.03 (3)°	*T* = 293 K
V = 2005 (4) $Å^3$	Prism, colourless
Z = 4	0.24 × 0.17 × 0.10 mm

Data collection

Bruker D8 VENTURE diffractometer	3653 independent reflections
Radiation source: microfocus sealed tube, INCOATEC ImS 3.0	2407 reflections with *I* > 2s(*I*)
Multilayer mirror monochromator	R_{int} = 0.095
Detector resolution: 7.3910 pixels mm^{-1}	q_{max} = 25.4°, q_{min} = 1.4°

w and p scan	h = -18→18
Absorption correction: multi-scan *SADABS2016*/2 - Bruker AXS area detector scaling and absorption correction	k = -9→9
T_{min} = 0.83, T_{max} = 0.99	l = -21→21
24369 measured reflections	

Refinement

Refinement on F^2	Secondary atom site location: difference Fourier map
Least-squares matrix: full	Hydrogen site location: inferred from neighbouring sites
$R[F^2 > 2s(F^2)]$ = 0.051	H-atom parameters constrained
$wR(F^2)$ = 0.142	$w = 1/[s^2(F_o^2) + (0.0592P)^2 + 0.4105P]$ where $P = (F_o^2 + 2F_c^2)/3$
S = 1.09	$(D/s)_{max}$ < 0.001
3653 reflections	$D\rho_{max}$ = 0.26 e Å^{-3}
291 parameters	$D\rho_{min}$ = -0.25 e Å^{-3}
0 restraints	Extinction correction: *SHELXL2019*/1 (Sheldrick 2019)
Primary atom site location: structure-invariant direct methods	Extinction coefficient: 0.033 (3)

Special details

Geometry. All esds (except the esd in the dihedral angle between two l.s. planes) are estimated using the full covariance matrix. The cell esds are taken into account individually in the estimation of esds in distances, angles and torsion angles; correlations between esds in cell parameters are only used when they are defined by crystal symmetry. An approximate (isotropic) treatment of cell esds is used for estimating esds involving l.s. planes.

*Fractional atomic coordinates and isotropic or equivalent isotropic displacement parameters (Å^2) for (**4f′**)*

	x	*y*	*z*	U_{iso}*/U_{eq}
C1''	0.68722 (17)	0.3767 (3)	0.27686 (13)	0.0421 (6)
C1'	0.69777 (16)	0.8420 (3)	0.45890 (14)	0.0392 (6)
C2	0.77425 (16)	0.8665 (3)	0.42629 (13)	0.0395 (6)
C2''	0.61462 (19)	0.3246 (3)	0.29954 (15)	0.0521 (7)
H2''	0.586562	0.397300	0.325624	0.063000*
C2'	0.61287 (17)	0.7705 (3)	0.41151 (14)	0.0432 (6)
H2'	0.605322	0.737276	0.359009	0.052000*
C3	0.80162 (16)	0.7597 (3)	0.37840 (14)	0.0397 (6)
C3''	0.5817 (2)	0.1600 (4)	0.28352 (16)	0.0580 (7)
H3''	0.533019	0.122701	0.300247	0.070000*
C3'	0.53975 (17)	0.7477 (3)	0.44052 (14)	0.0442 (6)
H3'	0.483738	0.699190	0.407740	0.053000*
C4	0.87428 (18)	0.8033 (3)	0.34426 (15)	0.0467 (6)
C4''	0.62166 (19)	0.0558 (3)	0.24340 (15)	0.0554 (7)
H4''	0.599993	-0.053021	0.233335	0.067000*
C4'	0.54954 (17)	0.7971 (3)	0.51870 (14)	0.0414 (6)
C5	0.98381 (18)	1.0330 (4)	0.33767 (17)	0.0549 (7)
H5	1.001725	0.972542	0.300236	0.066000*
C4A	0.91499 (16)	0.9689 (3)	0.36690 (14)	0.0436 (6)
C5''	0.7355 (2)	0.0055 (3)	0.17195 (16)	0.0599 (8)

H5''	0.714314	-0.103443	0.161031	0.072000*
C5'	0.63350 (17)	0.8712 (3)	0.56662 (14)	0.0461 (6)
H5'	0.640616	0.905460	0.618901	0.055000*
C6	1.0249 (2)	1.1849 (4)	0.36413 (19)	0.0631 (8)
H6	1.070266	1.227022	0.344488	0.076000*
C6''	0.8042 (2)	0.0616 (4)	0.14474 (18)	0.0667 (8)
H6''	0.827383	-0.007023	0.113309	0.080000*
C6'	0.70598 (17)	0.8940 (3)	0.53693 (14)	0.0449 (6)
H6'	0.761424	0.944808	0.569318	0.054000*
C7	0.9985 (2)	1.2747 (4)	0.42000 (19)	0.0615 (8)
H7	1.027773	1.375820	0.438674	0.074000*
C7''	0.8400 (2)	0.2220 (4)	0.16390 (17)	0.0596 (7)
H7''	0.888439	0.258288	0.146496	0.072000*
C8	0.92939 (18)	1.2170 (3)	0.44866 (17)	0.0528 (7)
H8	0.911302	1.278329	0.485731	0.063000*
C8''	0.80491 (17)	0.3267 (3)	0.20793 (15)	0.0485 (6)
H8''	0.829706	0.433177	0.220393	0.058000*
C8'	0.39363 (18)	0.7085 (4)	0.50447 (17)	0.0576 (7)
H8'A	0.349745	0.712518	0.533115	0.086000*
H8'B	0.403163	0.595012	0.492193	0.086000*
H8'C	0.368925	0.770669	0.455080	0.086000*
C8A	0.88802 (16)	1.0649 (3)	0.42039 (14)	0.0432 (6)
C10	0.76614 (16)	0.5859 (3)	0.37025 (14)	0.0401 (6)
C4A''	0.69554 (18)	0.1096 (3)	0.21662 (14)	0.0475 (6)
C8A''	0.73125 (16)	0.2740 (3)	0.23455 (13)	0.0398 (6)
O1	0.81623 (11)	1.0167 (2)	0.44827 (10)	0.0467 (4)
O7'	0.48190 (12)	0.7792 (2)	0.55382 (10)	0.0565 (5)
O9	0.90145 (15)	0.7069 (2)	0.30221 (13)	0.0710 (6)
O11	0.77757 (13)	0.4925 (2)	0.42593 (10)	0.0564 (5)
O12	0.71946 (12)	0.5429 (2)	0.29152 (9)	0.0470 (5)

*Atomic displacement parameters (Å²) for (**4f′**)*

	U^{11}	U^{22}	U^{33}	U^{12}	U^{13}	U^{23}
C1''	0.0517 (14)	0.0356 (13)	0.0370 (12)	-0.0008 (11)	0.0126 (11)	0.0014 (10)
C1'	0.0428 (13)	0.0337 (12)	0.0421 (13)	0.0039 (10)	0.0159 (11)	-0.0010 (10)
C2	0.0371 (13)	0.0367 (13)	0.0418 (13)	0.0009 (10)	0.0099 (10)	-0.0010 (10)
C2''	0.0585 (17)	0.0549 (16)	0.0454 (14)	0.0001 (13)	0.0211 (13)	0.0007 (12)
C2'	0.0445 (14)	0.0465 (14)	0.0388 (12)	0.0011 (11)	0.0147 (11)	-0.0064 (11)
C3	0.0418 (13)	0.0366 (13)	0.0408 (12)	0.0021 (10)	0.0141 (11)	-0.0013 (10)
C3''	0.0585 (17)	0.0604 (18)	0.0540 (16)	-0.0133 (14)	0.0179 (14)	0.0044 (14)
C3'	0.0421 (14)	0.0464 (14)	0.0426 (13)	-0.0023 (11)	0.0126 (11)	-0.0064 (11)
C4	0.0476 (15)	0.0426 (14)	0.0532 (15)	0.0088 (12)	0.0214 (12)	0.0015 (12)
C4''	0.0643 (18)	0.0416 (14)	0.0508 (15)	-0.0118 (13)	0.0074 (14)	0.0032 (12)
C4'	0.0449 (14)	0.0390 (13)	0.0419 (13)	0.0038 (11)	0.0167 (11)	0.0038 (11)
C5	0.0481 (15)	0.0578 (17)	0.0622 (16)	0.0044 (13)	0.0232 (13)	0.0070 (14)
C4A	0.0401 (13)	0.0415 (14)	0.0477 (14)	0.0051 (11)	0.0132 (11)	0.0057 (11)
C5''	0.0668 (19)	0.0406 (15)	0.0601 (17)	0.0056 (13)	0.0060 (15)	-0.0098 (13)
C5'	0.0563 (16)	0.0469 (15)	0.0361 (12)	0.0005 (12)	0.0171 (12)	-0.0067 (11)
C6	0.0504 (16)	0.0593 (18)	0.080 (2)	-0.0030 (14)	0.0236 (15)	0.0123 (16)
C6''	0.0658	0.0616	0.0688	0.0164	0.0179	-0.0150

	(19)	(19)	(19)	(16)	(16)	(16)
C6'	0.0446 (14)	0.0435 (14)	0.0442 (13)	-0.0010 (11)	0.0123 (11)	-0.0062 (12)
C7	0.0531 (17)	0.0510 (16)	0.0754 (19)	-0.0100 (13)	0.0155 (15)	0.0022 (15)
C7''	0.0502 (16)	0.0616 (18)	0.0681 (18)	0.0054 (14)	0.0216 (14)	-0.0096 (15)
C8	0.0493 (15)	0.0435 (15)	0.0621 (16)	-0.0020 (12)	0.0146 (13)	-0.0042 (13)
C8''	0.0468 (15)	0.0468 (15)	0.0493 (14)	0.0003 (12)	0.0130 (12)	-0.0043 (12)
C8'	0.0504 (16)	0.0667 (19)	0.0582 (16)	-0.0092 (14)	0.0219 (13)	0.0061 (14)
C8A	0.0354 (13)	0.0409 (14)	0.0502 (14)	0.0024 (10)	0.0107 (11)	0.0063 (11)
C10	0.0408 (13)	0.0381 (13)	0.0428 (13)	0.0069 (11)	0.0161 (11)	-0.0015 (12)
C4A''	0.0517 (15)	0.0391 (14)	0.0419 (13)	0.0031 (12)	0.0034 (12)	0.0029 (11)
C8A''	0.0415 (13)	0.0382 (13)	0.0331 (11)	0.0045 (10)	0.0044 (10)	0.0032 (10)
O1	0.0461 (10)	0.0395 (9)	0.0566 (10)	-0.0032 (8)	0.0205 (8)	-0.0074 (8)
O7'	0.0532 (11)	0.0741 (13)	0.0486 (10)	-0.0068 (9)	0.0255 (9)	-0.0027 (9)
O9	0.0797 (14)	0.0585 (12)	0.0965 (16)	-0.0046 (11)	0.0580 (13)	-0.0199 (11)
O11	0.0710 (13)	0.0444 (11)	0.0470 (10)	0.0006 (9)	0.0116 (9)	0.0059 (9)
O12	0.0634 (11)	0.0364 (9)	0.0416 (9)	-0.0022 (8)	0.0184 (8)	-0.0025 (7)

*Geometric parameters (Å, º) for (**4f′**)*

C1"—C2"	1.354 (4)	C4A—C8A	1.382 (4)
C1"—O12	1.416 (3)	C5"—C6"	1.359 (4)
C1"—C8A"	1.420 (3)	C5"—C4A"	1.416 (4)
C1'—C2'	1.391 (3)	C5"—H5"	0.9300
C1'—C6'	1.399 (3)	C5'—C6'	1.376 (4)
C1'—C2	1.467 (3)	C5'—H5'	0.9300
C2—O1	1.357 (3)	C6—C7	1.383 (4)
C2—C3	1.362 (3)	C6—H6	0.9300
C2"—C3"	1.407 (4)	C6"—C7"	1.395 (4)
C2"—H2"	0.9300	C6"—H6"	0.9300
C2'—C3'	1.377 (3)	C6'—H6'	0.9300
C2'—H2'	0.9300	C7—C8	1.385 (4)
C3—C4	1.462 (4)	C7—H7	0.9300
C3—C10	1.486 (4)	C7"—C8"	1.368 (4)
C3"—C4"	1.362 (4)	C7"—H7"	0.9300
C3"—H3"	0.9300	C8—C8A	1.385 (4)
C3'—C4'	1.390 (3)	C8—H8	0.9300
C3'—H3'	0.9300	C8"—C8A"	1.411 (4)
C4—O9	1.235 (3)	C8"—H8"	0.9300
C4—C4A	1.463 (4)	C8'—O7'	1.435 (3)
C4"—C4A"	1.418 (4)	C8'—H8'A	0.9600
C4"—H4"	0.9300	C8'—H8'B	0.9600
C4'—O7'	1.369 (3)	C8'—H8'C	0.9600
C4'—C5'	1.392 (4)	C8A—O1	1.388 (3)
C5—C6	1.376 (4)	C10—O11	1.199 (3)
C5—C4A	1.405 (4)	C10—O12	1.365 (3)
C5—H5	0.9300	C4A"—C8A"	1.421 (4)
C2"—C1"—O12	120.0 (2)	C4'—C5'—H5'	119.9000
C2"—C1"—C8A"	123.0 (2)	C5—C6—C7	119.9 (3)

O12—C1"—C8A"	116.8 (2)	C5—C6—H6	120.1000
C2'—C1'—C6'	117.7 (2)	C7—C6—H6	120.1000
C2'—C1'—C2	120.5 (2)	C5"—C6"—C7"	120.1 (3)
C6'—C1'—C2	121.8 (2)	C5"—C6"—H6"	120.0000
O1—C2—C3	121.8 (2)	C7"—C6"—H6"	120.0000
O1—C2—C1'	111.14 (19)	C5'—C6'—C1'	121.2 (2)
C3—C2—C1'	127.1 (2)	C5'—C6'—H6'	119.4000
C1"—C2"—C3"	119.8 (3)	C1'—C6'—H6'	119.4000
C1"—C2"—H2"	120.1000	C6—C7—C8	121.3 (3)
C3"—C2"—H2"	120.1000	C6—C7—H7	119.4000
C3'—C2'—C1'	121.5 (2)	C8—C7—H7	119.4000
C3'—C2'—H2'	119.2000	C8"—C7"—C6"	120.9 (3)
C1'—C2'—H2'	119.2000	C8"—C7"—H7"	119.6000
C2—C3—C4	122.1 (2)	C6"—C7"—H7"	119.6000
C2—C3—C10	118.5 (2)	C7—C8—C8A	117.9 (3)
C4—C3—C10	118.8 (2)	C7—C8—H8	121.0000
C4"—C3"—C2"	119.6 (3)	C8A—C8—H8	121.0000
C4"—C3"—H3"	120.2000	C7"—C8"—C8A"	120.3 (3)
C2"—C3"—H3"	120.2000	C7"—C8"—H8"	119.8000
C2'—C3'—C4'	120.1 (2)	C8A"—C8"—H8"	119.8000
C2'—C3'—H3'	120.0000	O7'—C8'—H8'A	109.5000
C4'—C3'—H3'	120.0000	O7'—C8'—H8'B	109.5000
O9—C4—C3	123.0 (2)	H8'A—C8'—H8'B	109.5000
O9—C4—C4A	122.7 (2)	O7'—C8'—H8'C	109.5000
C3—C4—C4A	114.2 (2)	H8'A—C8'—H8'C	109.5000
C3"—C4"—C4A"	121.5 (3)	H8'B—C8'—H8'C	109.5000
C3"—C4"—H4"	119.3000	C4A—C8A—C8	122.4 (2)
C4A"—C4"—H4"	119.3000	C4A—C8A—O1	122.1 (2)
O7'—C4'—C3'	125.0 (2)	C8—C8A—O1	115.5 (2)
O7'—C4'—C5'	115.9 (2)	O11—C10—O12	123.3 (2)
C3'—C4'—C5'	119.2 (2)	O11—C10—C3	124.3 (2)

C6—C5—C4A	120.4 (3)	O12—C10—C3	112.3 (2)
C6—C5—H5	119.8000	C5"—C4A"—C4"	122.4 (3)
C4A—C5—H5	119.8000	C5"—C4A"—C8A"	118.2 (2)
C8A—C4A—C5	118.1 (2)	C4"—C4A"—C8A"	119.4 (2)
C8A—C4A—C4	120.0 (2)	C8"—C8A"—C1"	124.2 (2)
C5—C4A—C4	121.9 (2)	C8"—C8A"—C4A"	119.2 (2)
C6"—C5"—C4A"	121.3 (3)	C1"—C8A"—C4A"	116.6 (2)
C6"—C5"—H5"	119.3000	C2—O1—C8A	119.51 (18)
C4A"—C5"—H5"	119.3000	C4'—O7'—C8'	116.7 (2)
C6'—C5'—C4'	120.2 (2)	C10—O12—C1"	116.70 (18)
C6'—C5'—H5'	119.9000		
C2'—C1'—C2—O1	138.3 (2)	C6—C7—C8—C8A	0.8 (4)
C6'—C1'—C2—O1	-39.6 (3)	C6"—C7"—C8"—C8A"	-0.2 (4)
C2'—C1'—C2—C3	-40.2 (4)	C5—C4A—C8A—C8	-3.1 (4)
C6'—C1'—C2—C3	141.9 (2)	C4—C4A—C8A—C8	175.0 (2)
O12—C1"—C2"—C3"	178.9 (2)	C5—C4A—C8A—O1	175.7 (2)
C8A"—C1"—C2"—C3"	2.1 (4)	C4—C4A—C8A—O1	-6.2 (3)
C6'—C1'—C2'—C3'	-1.4 (4)	C7—C8—C8A—C4A	1.7 (4)
C2—C1'—C2'—C3'	-179.4 (2)	C7—C8—C8A—O1	-177.2 (2)
O1—C2—C3—C4	-3.3 (3)	C2—C3—C10—O11	-57.5 (3)
C1'—C2—C3—	175.1 (2)	C4—C3—C10—	114.1 (3)

C4		O11	
O1—C2—C3—C10	168.0 (2)	C2—C3—C10—O12	123.2 (2)
C1'—C2—C3—C10	-13.6 (3)	C4—C3—C10—O12	-65.2 (3)
C1''—C2''—C3''—C4''	-1.7 (4)	C6''—C5''—C4A''—C4''	177.9 (3)
C1'—C2'—C3'—C4'	0.3 (4)	C6''—C5''—C4A''—C8A''	-1.9 (4)
C2—C3—C4—O9	178.4 (2)	C3''—C4''—C4A''—C5''	-177.4 (2)
C10—C3—C4—O9	7.1 (4)	C3''—C4''—C4A''—C8A''	2.4 (4)
C2—C3—C4—C4A	0.8 (3)	C7''—C8''—C8A''—C1''	-176.6 (2)
C10—C3—C4—C4A	-170.5 (2)	C7''—C8''—C8A''—C4A''	1.4 (3)
C2''—C3''—C4''—C4A''	-0.5 (4)	C2''—C1''—C8A''—C8''	177.9 (2)
C2'—C3'—C4'—O7'	-179.4 (2)	O12—C1''—C8A''—C8''	1.0 (3)
C2'—C3'—C4'—C5'	0.7 (4)	C2''—C1''—C8A''—C4A''	-0.2 (3)
C6—C5—C4A—C8A	2.1 (4)	O12—C1''—C8A''—C4A''	-177.09 (19)
C6—C5—C4A—C4	-175.9 (2)	C5''—C4A''—C8A''—C8''	-0.4 (3)
O9—C4—C4A—C8A	-173.8 (2)	C4''—C4A''—C8A''—C8''	179.8 (2)
C3—C4—C4A—C8A	3.8 (3)	C5''—C4A''—C8A''—C1''	177.8 (2)
O9—C4—C4A—C5	4.2 (4)	C4''—C4A''—C8A''—C1''	-2.0 (3)
C3—C4—C4A—C5	-178.2 (2)	C3—C2—O1—C8A	1.1 (3)
O7'—C4'—C5'—	179.7 (2)	C1'—C2—O1—	-177.51 (18)

C6'		C8A	
C3'—C4'—C5'—C6'	-0.5 (4)	C4A—C8A—O1—C2	3.8 (3)
C4A—C5—C6—C7	0.2 (4)	C8—C8A—O1—C2	-177.4 (2)
C4A''—C5''—C6''—C7''	3.1 (4)	C3'—C4'—O7'—C8'	-2.0 (3)
C4'—C5'—C6'—C1'	-0.8 (4)	C5'—C4'—O7'—C8'	177.9 (2)
C2'—C1'—C6'—C5'	1.7 (4)	O11—C10—O12—C1''	-2.6 (3)
C2—C1'—C6'—C5'	179.6 (2)	C3—C10—O12—C1''	176.72 (19)
C5—C6—C7—C8	-1.7 (4)	C2''—C1''—O12—C10	70.6 (3)
C5''—C6''—C7''—C8''	-2.0 (4)	C8A''—C1''—O12—C10	-112.4 (2)

*Hydrogen-bond geometry (Å, º) for (**4f'**)*

D—H···*A*	*D*—H	H···*A*	*D*···*A*	*D*—H···*A*
C8—H8···O11^{i}	0.93	2.59	3.113 (4)	116

Symmetry code: (i) *x*, *y*+1, *z*.

References:

49. Sheldrick, G. A short history of SHELX. *Acta Crystallogr. A* **2008**, *64*, 112-122, doi:http://dx.doi.org/10.1107/S0108767307043930.
50. Westrip, S. publCIF: software for editing, validating and formatting crystallographic information files. *J. Appl. Crystallogr.* **2010**, *43*, 920-925, doi:http://dx.doi.org/10.1107/S0021889810022120.
51. Dolomanov, O.; Bourhis, L.; Gildea, R.; Howard, J.; Puschmann, H. OLEX2: a complete structure solution, refinement and analysis program. *J. Appl. Crystallogr.* **2009**, *42*, 339-341, doi:http://dx.doi.org/10.1107/S0021889808042726.
52. Spek, A. Single-crystal structure validation with the program PLATON. *J. Appl. Crystallogr.* **2003**, *36*, 7-13, doi:http://dx.doi.org/10.1107/S0021889802022112.

2. Characterization of the Synthesized Compounds

2.1. Flavone-3-Carboxamides (2)

Methyl (2-(4'-methoxyphenyl)-4-oxo-4*H*-chromene-3-carbonyl)alaninate (2a).

Yield: 150 mg (79%); Yellow semi-solid; Eluent (Column Chromatography & TLC) [45% $CHCl_3$, 45% *n*-Hexane, 10% EtOAc, R_f : 0.3]. **^{1}H NMR** (500 MHz, $CDCl_3$) δ 8.56 (NH, *s*, 1H), 8.26 (H_{Ar}, *dd*, *J* = 8.0, 1.2 *Hz*, 1H), 7.76 – 7.70 (H_{Ar}, *m*, 3H), 7.52 (H_{Ar}, *d*, *J* = 8.3 *Hz*, 1H), 7.46 (H_{Ar}, *t*, *J* = 7.5 *Hz*, 1H), 7.00 (H_{Ar}, *d*, *J* = 8.8 *Hz*, 2H), 4.72 (*br s*, (-*C$\underline{H}$-(CH_3))$_{Ala}$, 1H), 3.89 ((-OC$\underline{H}_3$), s, 3H), 3.78 ((-OC$\underline{H}_3$), *s*, 3H), 1.55 (H_{Ar}, *d*, *J* = 7.2 *Hz*, (-*CH-(C$\underline{H}_3$))$_{Ala}$, 3H). **^{13}C{^{1}H} NMR** (125 MHz, $CDCl_3$) δ 177.1, 173.2, 168.2, 163.6, 162.2, 155.3, 134.3, 130.88(2xC_{Ar}), 126.1, 125.7, 125.1, 123.3, 117.9, 115.1, 113.7 (2xC_{Ar}), 55.4(-O$\underline{C}H_3$), 52.4(-*$\underline{C}$H-)$_{Ala}$, 48.5(-O$\underline{C}H_3$), 18.1(-*CH-($\underline{C}H_3$))$_{Ala}$. **IR** (KBr, ν (cm^{-1})): 3283 *m*, 1753 *m*, 1736 *m*, 1658 *vs*, 1647 *vs*, 1606 *s*, 1511 *m*, 1465 *s*, 1383 *vs*, 1260 *vs*, 1185 *m*, 1020 *w*, 761 *s*. **MS** (EI): *m/z* (rel. int, %): 381 (15, [M^+]), 322 (5), 295 (35), 279 (100), 159 (45), 144 (5), 116 (5). **HRMS** (ESI-Q-TOF) *m/z* calcd for $C_{21}H_{19}NO_6$ $[M+H]^+$: 382.1285; found: 382.1288.

Methyl (2-(4'-methoxyphenyl)-4-oxo-4*H*-chromene-3-carbonyl)valinate (2b).

Yield: 143 mg (70%); Yellowish oil; Eluent (Column Chromatography & TLC) [70% *n*-Hexane, 29% EtOAc, 1% MeOH, R_f : 0.3]. **^{1}H NMR** (500 MHz, $CDCl_3$) δ 8.58 (NH, *d*, *J* = 6.5 *Hz*, 1H), 8.29 (H_{Ar}, *dd*, *J* = 8.0, 1.2 *Hz*, 1H), 7.70 – 7.73 (H_{Ar}, *m*, 3H), 7.52 (H_{Ar}, *d*, *J* = 8.3 Hz, 1H), 7.47 (H_{Ar}, *t*, *J* = 7.6 *Hz*, 1H), 6.99 (H_{Ar}, *d*, *J* = 8.8 *Hz*, 2H), 4.68 ((-*C$\underline{H}$-CH(CH_3)$_2$)$_{Val}$, *t*, *J* = 5.4 *Hz*, 1H), 3.88 ((-OC$\underline{H}_3$), *s*, 3H), 3.77 ((-OC$\underline{H}_3$), *s*, 3H), 2.28 – 2.73 ((C$\underline{H}$(CH_3)$_2$)$_{Val}$, *m*, 1H), 1.07 ((CH(C$\underline{H}_3$)$_2$)$_{Val}$, *d*, *J* = 1.0 *Hz*, 3H), 1.06 ((CH(C$\underline{H}_3$)$_2$)$_{Val}$, *d*, *J* = 1.0 *Hz*, 3H). **^{13}C{^{1}H} NMR** (125 MHz, $CDCl_3$) δ 177.2, 172.1, 168.4, 163.9, 162.2, 155.3, 134.3, 130.8(2xC_{Ar}), 126.1, 125.7, 125.2, 123.3, 117.9, 115.2, 113.7(2xC_{Ar}), 57.8(-O$\underline{C}H_3$), 55.4(-O$\underline{C}H_3$), 52.1(-*$\underline{C}$H-CH(CH_3)$_2$)$_{Val}$, 31.0(-$\underline{C}$H(CH_3)$_2$)$_{Val}$, 19.2(CH($\underline{C}H_3$)$_2$)$_{Val}$, 18.0(CH($\underline{C}H_3$)$_2$)$_{Val}$. **IR** (KBr, ν (cm^{-1})): 3299 *m*, 1739 *s*, 1656 *vs*, 1646 *vs*, 1606 *s*, 1510 *m*, 1467 *m*, 1381 *vs*, 1261 *vs*, 1185 *m*, 1011 *w*, 767 *s*. **MS** (EI): *m/z* (rel. int, %): 409 (2, [M^+]),

350 (10), 294 (30), 279 (100), 252 (5), 159 (40), 144 (5), 116 (5). **HRMS** (ESI-Q-TOF) *m/z* calcd for $C_{23}H_{23}NO_6$ $[M+H]^+$: 410.1598; found: 410.1603.

Methyl 2-(2-(4'-methoxyphenyl)-4-oxo-4*H*-chromene-3-carboxamido)-2-phenylacetate (2c).

Yield: 117 mg (53%); Yellow semi-solid; Eluent (Column Chromatography & TLC) [70% *n*-Hexane, 29% EtOAc, 1% MeOH, R_f : 0.3]. ^{1}**H NMR** (500 MHz, $CDCl_3$) δ 9.14 (NH, *d*, *J* = 5.2 *Hz*, 1H), 8.28 (H_{Ar}, *d*, *J* = 7.9 *Hz*, 1H), 7.73 (H_{Ar}, *t*, *J* = 7.8 *Hz*, 1H), 7.65 (H_{Ar}, *d*, *J* = 8.7 *Hz*, 2H), 7.55 – 7.44 (H_{Ar}, m, 4H), 7.35 – 7.41 (H_{Ar}, m, 3H), 6.91 (*d*, *J* = 8.7 *Hz*, 2H), 5.72 ((-*C<u>H</u>-)$_{Ph}$, *d*, *J* = 5.2 *Hz*, 1H), 3.87 ((-OC<u>H</u>$_3$), *s*, 3H), 3.76 ((-OC<u>H</u>$_3$), *s*, 3H). ^{13}**C{^{1}H} NMR** (125 MHz, $CDCl_3$) δ 186.6, 177.1, 171.1, 168.5, 163.5, 162.2, 155.3, 136.4, 134.4, 130.9(2xC_{Ar}), 128.9, 128.4, 127.6(2xC_{Ar}), 126.2, 125.80, 124.9(2xC_{Ar}), 123.3, 117.9, 114.9, 113.7(2xC_{Ar}), 57.1(-O<u>C</u>H$_3$), 55.4(-O<u>C</u>H$_3$), 52.7(-*<u>C</u>H-)$_{Ph}$. **IR** (KBr, ν (cm^{-1})): 3245 *w*, 1757 *m*, 1749 *m*, 1652 *vs*, 1628 *s*, 1607 *s*, 1511 *m*, 1466 *m*, 1386 *s*, 1258 *s*, 1179 *m*, 1009 *w*, 702 *w*. **MS** (EI): *m/z* (rel. int, %): 443 (1, [M^+]), 367 (100), 350 (100), 323 (10), 294 (5), 264 (50), 249 (5), 221 (20), 176 (10), 147 (8), 73 (10). **HRMS** (ESI-Q-TOF) *m/z* calcd for $C_{26}H_{21}NO_6$ $[M+H]^+$: 444.1442; found: 444.1441.

Methyl (2-(4'-methoxyphenyl)-4-oxo-4*H*-chromene-3-carbonyl)phenylalaninate (2d).

Yield: 155 mg (68%); Yellow semi-solid; Eluent (Column Chromatography & TLC) [70% *n*-Hexane, 29% EtOAc, 1% MeOH, R_f : 0.3]. ^{1}**H NMR** (500 MHz, $CDCl_3$) δ 8.60 (NH, s, 1H), 8.28 (H_{Ar}, *dd*, *J* = 7.9, 1.2 *Hz*, 1H), 7.76 – 7.69 (H_{Ar}, *td*, *J* = 8.5, 1.5 *Hz*, 1H), 7.62 (H_{Ar}, *d*, *J* = 8.8 *Hz*, AA'BB', 2H), 7.51 (H_{Ar}, *d*, *J* = 8.4 *Hz*, 1H), 7.46 (H_{Ar}, *t*, *J* = 7.5 *Hz*, 1H), 7.36 – 7.24 (H_{Ar}, *m*, 3H + C<u>H</u>Cl$_3$ *residual*), 7.22 (H_{Ar}, *d*, *J* = 7.0 *Hz*, 2H), 6.97 (H_{Ar}, *d*, *J* = 8.8 *Hz*, AA'BB', 2H), 5.05 ((-*C<u>H</u>-)$_{Phe}$, *s*, 1H), 3.89 ((-OC<u>H</u>$_3$), s, 3H), 3.74 ((-OC<u>H</u>$_3$), s, 3H), 3.25 ((-H$_b$C<u>H</u>$_a$-)$_{Phe}$, *dd*, *J* = 13.8, 5.6 *Hz*, 1H), 3.20 ((-H$_a$C<u>H</u>$_b$-)$_{Phe}$, *dd*, *J* = 13.8, 7.0 *Hz*, 1H). ^{13}**C{^{1}H} NMR** (125 MHz, $CDCl_3$) δ 177.0, 171.8, 168.2, 163.8, 162.2, 155.3, 136.3, 134.3, 130.8(2xC_{Ar}), 129.4(2xC_{Ar}), 128.5(2xC_{Ar}), 126.9, 126.2, 125.7, 125.1, 123.3, 117.9, 115.1, 113.7(2xC_{Ar}), 55.4(-O<u>C</u>H$_3$), 53.7(-O<u>C</u>H$_3$), 52.2(-*<u>C</u>H-)$_{Phe}$, 38.2(-H$_a$<u>C</u>H$_b$-)$_{Phe}$. **IR** (KBr, ν (cm^{-1})): 3293 *w*, 1745 *s*, 1688 *s*, 1634 *s*, 1616 *s*, 1606 *vs*, 1510 *s*, 1466 *s*, 1381 *vs*, 1259 *m*, 1179 *m*, 1027 *w*, 762 *w*. **MS** (EI): *m/z* (rel. int, %): 457 (5, [M^+]), 396 (2), 367 (8), 294 (35),

279 (100), 159 (45), 116 (5), 91 (5). **HRMS** (ESI-Q-TOF) *m/z* calcd for $C_{27}H_{23}NO_6$ $[M+H]^+$: 458.1598; found: 458.1596.

Methyl (2-(4'-methoxyphenyl)-4-oxo-4*H*-chromene-3-carbonyl)serinate (2e).

Yield: 92 mg (46%); White solid; m.p: 164-166 °C; Eluent (Column Chromatography & TLC) [70% *n*-Hexane, 29% EtOAc, 1% MeOH, R_f : 0.3]. **^{1}H NMR** (500 MHz, $CDCl_3$) δ 8.24 (H_{Ar}, *dd*, *J* = 7.9, 1.0 *Hz*, 1H), 8.10 (NH, *s*, 1H), 7.83 (H_{Ar}, *d*, *J* = 8.8 *Hz*, AA'BB', 2H), 7.74 (H_{Ar}, *td*, *J* = 8.5, 1.0 *Hz*, 1H), 7.52 (H_{Ar}, *d*, *J* = 8.4 *Hz*, 1H), 7.44 (H_{Ar}, *t*, *J* = 7.5 *Hz*, 1H), 7.02 (H_{Ar}, *d*, *J* = 8.8 *Hz*, AA'BB', 2H), 4.83 ((-*C<u>H</u>-)$_{Ser}$, *br s*, 1H), 4.22 ((-H_bC<u>H</u>$_a$-)$_{Ser}$, *dd*, *J* = 11.6, 3.5 *Hz*, 1H), 4.05 ((-H_aC<u>H</u>$_b$-)$_{Ser}$, *dd*, *J* = 11.6, 3.9 *Hz*, 1H), 3.91 ((-OC<u>H</u>$_3$), *s*, 3H), 3.84 ((-OC<u>H</u>$_3$), *s*, 3H), 2.96 (-OH, *br s*, 1H). **^{13}C{^{1}H} NMR** (125 MHz, $CDCl_3$) δ 177.0, 170.7, 166.4, 164.4, 162.5, 155.5, 134.5, 130.7(2xC_{Ar}), 126.1, 125.8, 124.3, 123.0, 117.9, 116.4, 114.0(2xC_{Ar}), 62.5, 55.6, 55.5, 52.7. **IR** (KBr, ν (cm^{-1})): 3457 *m*, 3286 *m*, 1735 *s*, 1654 *vs*, 1637 *vs*, 1604 *s*, 1606 *vs*, 1511 *m*, 1386 *s*, 1254 *w*, 1188 *m*, 1033 *w*, 760 *w*. **HRMS** (ESI-Q-TOF) *m/z* calcd for $C_{21}H_{19}NO_7$ $[M+H]^+$: 398.1234; found: 398.1236.

2-(4'-Methoxyphenyl)-4-oxo-*N*-phenyl-4*H*-chromene-3-carboxamide (2f).

Yield: 41 mg (22%); Yellow gummy; Eluent (Column Chromatography & TLC) [70% *n*-Hexane, 29% EtOAc, 1% MeOH, R_f : 0.2]. **^{1}H NMR** (500 MHz, $CDCl_3$) δ 10.56 (NH, *s*, 1H), 8.28 (H_{Ar}, *d*, *J* = 7.9 *Hz*, 1H), 7.76 (H_{Ar}, *d*, *J* = 8.6 *Hz*, 3H), 7.69 (H_{Ar}, *d*, *J* = 8.0 *Hz*, 2H), 7.54 (H_{Ar}, *d*, *J* = 8.4 *Hz*, 1H), 7.49 (H_{Ar}, *t*, *J* = 7.5 *Hz*, 1H), 7.34 (H_{Ar}, *t*, *J* = 7.7 *Hz*, 2H), 7.13 (H_{Ar}, *t*, *J* = 7.3 *Hz*, 1H), 7.03 (H_{Ar}, *d*, *J* = 8.6 *Hz*, 2H), 3.91 ((-OC<u>H</u>$_3$), *s*, 3H). **^{13}C{^{1}H} NMR** (125 MHz, $CDCl_3$) δ 177.7, 169.6, 162.3, 161.7, 155.3, 138.3, 134.5, 130.9(2xC_{Ar}), 128.8(2xC_{Ar}), 126.1, 125.9, 125.4, 124.2, 123.3, 120.4(2xC_{Ar}), 117.9, 115.2, 113.9(2xC_{Ar}), 55.4(-O<u>C</u>H$_3$). **IR** (KBr, ν (cm^{-1})): 3266 *w*, 1685m *s*, 1617 *s*, 1604 *vs*, 1543 *w*, 1381 *vs*, 1261 *vs*, 1180 *s*, 1096 *w*, 758 *w*. **MS** (EI): *m/z* (rel. int, %): 371 (55, [M^+]), 279 (100), 159 (65), 144 (5), 116 (5). **HRMS** (ESI-Q-TOF) *m/z* calcd for $C_{23}H_{17}NO_4$ $[M+H]^+$: 372.1230; found: 372.1216.

2-(4-Methoxyphenyl)-4-oxo-*N*-(pyridin-4-ylmethyl)-4*H*-chromene-3-carboxamide (2g).

Yield: 70 mg (36%); Fluffy-White solid; m.p: 180-182 °C, R_f (96% $CHCl_3$, 4% MeOH) 0.4 Eluent (Column Chromatography & TLC) [96% $CHCl_3$, 4% MeOH, R_f : 0.4]. **^{1}H NMR** (500 MHz, $CDCl_3$) δ 9.11 (NH, *t*, *J* = 5.8 *Hz*, 1H), 8.59 (H_{Ar}, *d*, *J* = 6.0 *Hz*, AA'BB', 2H), 8.27 (H_{Ar}, *dd*, *J* = 8.0, 1.4 *Hz*, 1H), 7.81 – 7.76 (H_{Ar}, *dt*, *J* = 7.5, 1.5 *Hz*, 1H), 7.70 (H_{Ar}, *d*, *overlapped*, *J* = 8.8 *Hz*, AA'BB', 2H), 7.68 (H_{Ar}, *d*, *overlapped*, *J* = 6.0 *Hz*, AA'BB', 2H), 7.57 (H_{Ar}, *d*, *J* = 8.4 *Hz*, 1H), 7.51 (H_{Ar}, *t*, *J* = 7.5 *Hz*, 1H), 7.00 (H_{Ar}, *d*, *J* = 8.8 *Hz*, AA'BB', 2H),

4.77 ((-C<u>H</u>$_2$-), *d*, *J* = 5.9 *Hz*, 2H), 3.91 ((-OC<u>H</u>$_3$), *s*, 3H). **^{13}C{^{1}H} NMR** (125 MHz, $CDCl_3$) δ 177.5, 168.6, 164.9, 162.4, 161.3, 155.4, 144.3, 144.3, 134.7, 130.7(2xC$_{AA'BB'}$), 126.1, 126.0, 124.9, 124.1, 124.0, 123.1, 118.1, 115.1, 113.9(2xC$_{AA'BB'}$), 55.5(-O<u>C</u>H$_3$), 42.7(-<u>C</u>H$_2$-). **IR** (KBr, ν (cm^{-1})): 3442 *w*, 3057 *w*, 1647 *s*, 1640 *s*, 1617 *vs*, 1605 *vs*, 1510 *m*, 1465 *m*, 1389 *s*, 1259 *s*, 1181 *m*, 764 *w*. **HRMS** (ESI-Q-TOF) *m*/*z* calcd for $C_{23}H_{18}N_2O_4$ $[M+H]^+$: 387.1339; found: 387.1340.

N-Benzyl-2-(4-methoxyphenyl)-4-oxo-4H-chromene-3-carboxamide (2h).

Yield: 151 mg (44%); White solid; m.p: 193-195 °C, Eluent (Column Chromatography & TLC) [45% $CHCl_3$, 45% *n*-Hexane 10% EtOAc, R_f : 0.2]. **^{1}H NMR** (500 MHz, $CDCl_3$) δ 8.26 (H$_{Ar}$, *dd*, *J* = 8.0, 1.4 *Hz*, 1H), 8.22 (NH, *br s*, 1H), 7.74 (H$_{Ar}$, *dd*, *J* = 8.5, 1.5 *Hz*, 1H), 7.72 (H$_{Ar}$, *d*, *J* = 8.8 *Hz*, AA'BB', 2H), 7.53 (H$_{Ar}$, *d*, *J* = 8.3 *Hz*, 1H), 7.47 (H$_{Ar}$, *t*, *J* = 7.5 *Hz*, 1H), 7.40 – 7.33 (H$_{Ar}$, *m*, 4H), 6.99 (H$_{Ar}$, *d*, *J* = 8.8 *Hz*, AA'BB', 2H), 4.63 ((-C<u>H</u>$_2$-), *s*, 2H), 3.91 ((-OC<u>H</u>$_3$), *s*, 3H). **^{13}C{^{1}H} NMR** (125 MHz, $CDCl_3$) δ 177.1, 167.6, 163.9, 162.1, 155.4, 138.2, 134.3, 130.6(2xC$_{AA'BB'}$), 128.6(2xC$_{Ar}$), 127.8(2xC$_{Ar}$), 127.2, 126.1, 125.7, 125.2, 123.3, 117.9, 116.0, 113.8(2xC$_{AA'BB'}$), 55.4(-O<u>C</u>H$_3$), 43.8(-<u>C</u>H$_2$-). **IR** (KBr, ν (cm^{-1})): 3290 *s*, 1663 *s*, 1624 *s*, 1603 *vs*, 1513 *s*, 1474 *m*, 1267 *s*, 1184 *s*, 1110 *m*, 1020 *w*, 760 *w*. **MS** (EI): *m*/*z* (rel. int, %): 385 (25, $[M^+]$), 279 (80), 252 (45), 159 (50), 132 (25), 106 (100), 91 (40). **HRMS** (ESI-Q-TOF) *m*/*z* calcd for $C_{24}H_{19}NO_4$ $[M+H]^+$: 386.1387; found: 386.1386.

2-(4'-Methoxyphenyl)-4-oxo-N-phenethyl-4H-chromene-3-carboxamide (2i).

Yield: 82 mg (41%); White solid; m.p: 155-157 °C, Eluent (Column Chromatography & TLC) [45% $CHCl_3$, 45% *n*-Hexane, 10% EtOAc, R_f : 0.3]. **^{1}H NMR** (500 MHz, $CDCl_3$) δ 8.26 (H$_{Ar}$, *dd*, *J* = 8.0, 1.4 *Hz*, 1H), 7.85 (NH, *br s*, 1H), 7.76 – 7.71 (H$_{Ar}$, *td*, *J* = 8.8, 1.5 *Hz*, 1H), 7.68 (H$_{Ar}$, *d*, *J* = 8.8 *Hz*, AA'BB', 2H), 7.52 (H$_{Ar}$, *d*, *J* = 8.5 *Hz*, 1H), 7.46 (H$_{Ar}$, *td*, *J* = 8.0, 0.5 *Hz*, 1H), 7.31 (H$_{Ar}$, *t*, *J* = 7.0 *Hz*, 1H), 7.25 (H$_{Ar}$, *d*, *J* = 7.3 *Hz*, 1H), 7.22 (H$_{Ar}$, *d*, *J* = 7.1 *Hz*, 2H), 7.00 (H$_{Ar}$, *d*, *J* = 8.8 *Hz*, AA'BB', 2H), 3.91 ((-OC<u>H</u>$_3$), *s*, 3H), 3.71 ((-C<u>H</u>$_2$-), *t*, *J* = 7.1 *Hz*, 2H), 2.92 ((-C<u>H</u>$_2$-), *t*, *J* = 7.1 *Hz*, 2H). **^{13}C{^{1}H} NMR** (125 MHz, $CDCl_3$) δ 177.0, 167.3, 164.0, 162.1, 155.4, 139.1, 134.2, 130.6(2xC$_{Ar}$), 128.8(2xC$_{Ar}$), 128.5(2xC$_{Ar}$), 126.3, 126.1, 125.6, 125.2, 123.4, 117.9, 116.2, 113.8(2xC$_{Ar}$), 55.4(-O<u>C</u>H$_3$), 40.9(-<u>C</u>H$_2$-), 35.7(-<u>C</u>H$_2$-). **IR** (KBr, ν (cm^{-1})): 3439 *w*, 3316 *m*, 1660 *vs*, 1614 *vs*, 1608 *vs*, 1512 *s*, 1473 *m*, 1385 *s*, 1257 *s*, 1186 *s*, 1114 *w*, 1038 *w*, 771 *m*. **MS** (EI): *m*/*z* (rel. int, %): 399 (4, $[M^+]$), 294 (25), 279 (100), 159 (45), 116 (5). **HRMS** (ESI-Q-TOF) *m*/*z* calcd for $C_{25}H_{21}NO_4$ $[M+H]^+$: 400.1543; found: 400.1546.

***N*-(Benzo[d][1,3]dioxol-5''-ylmethyl)-2-(4'-methoxyphenyl)-4-oxo-4*H*-chromene-3-carboxamide (2j).**

Yield: 97 mg (45%); White solid, m.p: 198-200 °C, Eluent (Column Chromatography & TLC) [45% $CHCl_3$, 45% *n*-Hexane, 10% EtOAc, R_f : 0.2]. **^{1}H NMR** (500 MHz, DMSO-d_6) δ 8.82 (NH, *t*, *J* = 5.8 *Hz*, 1H), 8.12 (H_{Ar}, *d*, *J* = 7.8 *Hz*, 1H), 7.87 (H_{Ar}, *t*, *J* = 7.7 *Hz*, 1H), 7.80 (H_{Ar}, *d*, *J* = 8.8 *Hz*, AA'BB', 2H), 7.76 (H_{Ar}, *d*, *J* = 8.4 *Hz*, 1H), 7.55 (H_{Ar}, *t*, *J* = 7.5 *Hz*, 1H), 7.04 (H_{Ar}, *d*, *J* = 8.7 *Hz*, AA'BB', 2H), 6.82 (H_{Ar}, *d*, *J* = 7.9 *Hz*, 1H), 6.79 (H_{Ar}, *s*, 1H), 6.72 (*d*, *J* = 7.9 *Hz*, 1H), 6.00 ((-O-C$\underline{H}_2$-O-), *s*, 2H), 4.29 ((-C$\underline{H}_2$-), *d*, *J* = 5.8 *Hz*, 2H), 3.86 ((-O-C$\underline{H}_3$), *s*, 3H). **^{13}C{^{1}H} NMR** (125 MHz, DMSO-d_6) δ 175.4, 164.2, 162.1, 161.1, 155.8, 147.6, 146.5, 135.0, 133.0, 130.4(2x$C_{AA'BB'}$), 126.1, 125.5, 124.2, 123.1, 121.0, 120.4, 118.9, 114.5(2x$C_{AA'BB'}$), 108.5, 108.3, 101.2(-O-$\underline{C}H_2$-O-), 55.9(-O-$\underline{C}H_3$), 42.5(-$\underline{C}H_2$-). **IR** (KBr, ν (cm^{-1})): 3266 *m*, 1654 *vs*, 1632 *vs*, 1606 *s*, 1512 *m*, 1467 *m*, 1376 *s*, 1259 *s*, 1189 *s*, 1105 *w*, 1039 *w*, 763 *m*. **HRMS** (ESI-Q-TOF) *m/z* calcd for $C_{25}H_{19}NO_6$ $[M+H]^+$: 430.1285; found: 430.1287.

***N*-Methoxy-2-(4'-methoxyphenyl)-*N*-methyl-4-oxo-4*H*-chromene-3-carboxamide (2k).**

Yield: 121 mg (71%); Yellow gummy; Eluent (Column Chromatography & TLC) [45% $CHCl_3$, 18% *n*-Hexane, 37% EtOAc, R_f : 0.3]. (***Rotameric Mixture***: **3/1**); ***<u>Major Rotamer</u>*:** **^{1}H NMR** (500 *MHz*, $CDCl_3$) δ 8.28 (H_{Ar}, *dd*, *J* = 7.9, 1.2 *Hz*, 1H), 7.89 (H_{Ar}, *d*, *J* = 8.7 *Hz*, AA'BB', 2H), 7.83 (H_{Ar}, *d*, *J* = 8.8 *Hz*, 1H), 7.73 (H_{Ar}, *td*, *J* = 8.0, 1.0 *Hz*, 1H), 7.56 (H_{Ar}, *d*, *J* = 8.4 *Hz*, 1H), 7.45 (H_{Ar}, *t*, J = 7.5 *Hz*, 1H), 7.02 (*d*, J = 8.9 *Hz*, AA'BB', 2H), 3.90 ((O-C$\underline{H}_3$), *s*, 3H), 3.55 ((O-C$\underline{H}_3$), *s*, 3H), 3.34 ((N-C$\underline{H}_3$), *s*, 3H). **^{13}C{^{1}H} NMR** (125 MHz, $CDCl_3$) δ 175.5, 166.3, 162.1, 160.8, 155.9, 133.9, 129.8(2x$C_{AA'BB'}$), 126.1, 125.3, 124.4, 123.1, 118.4, 117.9, 114.3(2x$C_{AA'BB'}$), 61.8(O-$\underline{C}H_3$), 55.4(O-$\underline{C}H_3$), 32.4(N-$\underline{C}H_3$). ***<u>Minor Rotamer</u>*:** **^{1}H NMR** (500 *MHz*, $CDCl_3$) δ 8.28 (H_{Ar}, *dd*, *J* = 7.9, 1.2 *Hz*, 1H), 7.89 (H_{Ar}, *d*, *J* = 8.7 *Hz*, AA'BB', 2H), 7.83 (H_{Ar}, *d*, *overlapped*, *J* = 8.8 *Hz*, 1H), 7.73 (H_{Ar}, *td*, *overlapped*, *J* = 8.0, 1.0 *Hz*, 1H), 7.56 (H_{Ar}, *d*, *overlapped*, *J* = 8.4 *Hz*, 1H), 7.45 (H_{Ar}, *t*, *overlapped*, J = 7.5 *Hz*, 1H), 7.02 (*d*, *overlapped*, J = 8.9 *Hz*, AA'BB', 2H), <u>3.90</u> (<u>2x</u>(O-C$\underline{H}_3$), *s*, *overlapped*, 3H), 3.15 ((N-C$\underline{H}_3$), *s*, 3H). **^{13}C{^{1}H} NMR** (125 MHz, $CDCl_3$) δ 175.16, 166.30, 162.11, 160.83, 155.98, 134.26, 130.05(2x$C_{AA'BB'}$), 125.94, 125.62, 123.57, 120.01, 117.97, 117.38, 114.46(2x$C_{AA'BB'}$), 60.55(O-$\underline{C}H_3$), 55.45(O-$\underline{C}H_3$), 35.76(N-$\underline{C}H_3$). **IR** (KBr, ν (cm^{-1})): 3476 *w*, 3066 *w*, 1659 *vs*, 1636 *vs*, 1617 *vs*, 1606 *vs*, 1511 *s*, 1466 *s*, 1385 *m*, 1260 *m*, 1180 *m*, 1180 *m*, 1025 *w*, 761 *m*. **MS** (EI): *m/z* (rel. int, %): 339 (1, $[M^+]$), 308 (5), 279 (100), 159 (50), 144 (15), 116 (10). **HRMS** (ESI-Q-TOF) *m/z* calcd for $C_{19}H_{17}NO_5$ $[M+H]^+$: 340.1179; found: 340.1166.

_N,N_-diethyl-2-(4'-methoxyphenyl)-4-oxo-4_H_-chromene-3-carboxamide (2l).

Yield: 100 mg (57%); Yellow oil; Eluent (Column Chromatography & TLC) [45% $CHCl_3$, 45% *n*-Hexane, 10% EtOAc, R_f : 0.3]. **^{1}H NMR** (500 MHz, $CDCl_3$) δ 8.26 (H_{Ar}, *dd*, *J* = 7.9, 1.4 *Hz*, 1H), 7.92 (H_{Ar}, *d*, *J* = 8.9 *Hz*, AA'BB', 2H), 7.75 – 7.70 (H_{Ar}, *td*, *J* = 8.5, 1.5 *Hz*, 1H), 7.55 (H_{Ar}, *d*, *J* = 8.4 *Hz*, 1H), 7.44 (H_{Ar}, *t*, *J* = 7.5 *Hz*, 1H), 7.00 (H_{Ar}, *d*, *J* = 8.9 *Hz*, AA'BB', 2H), 3.90 ((O-C$\underline{H}_3$), *s*, 3H), 3.73 ((-H_bC-$\underline{H}_a$), *dq*, *J* = 14.2, 7.1 *Hz*, 1H), 3.43 ((-H_aC-$\underline{H}_b$), *dq*, *J* = 14.4, 7.2 *Hz*, 1H), 3.23 ((-H_dC-$\underline{H}_c$), *dq*, *J* = 14.4, 7.2 *Hz*, 1H), 3.14 ((-H_cC-$\underline{H}_d$), *dq*, *J* = 14.4, 7.2 *Hz*, 1H), 1.21 ((-C$\underline{H}_3$), *t*, *J* = 7.1 *Hz*, 3H), 0.91 ((-C$\underline{H}_3$), *t*, *J* = 7.2 *Hz*, 3H). **^{13}C{^{1}H} NMR** (125 MHz, $CDCl_3$) δ 175.6, 164.9, 162.1, 159.9, 155.9, 133.9, 130.1(2x$C_{AA'BB'}$), 126.0, 125.3, 124.2, 123.1, 119.0, 117.9, 114.1(2x$C_{AA'BB'}$), 55.5(-O$\underline{C}H_3$), 43.2(-$\underline{C}H_2$-), 39.2(-$\underline{C}H_2$-), 13.8(-$\underline{C}H_3$), 12.3(-$\underline{C}H_3$). **IR** (KBr, ν (cm^{-1})): 3483 *w*, 2966 *w*, 1640 *vs*, 1636 *vs*, 1615 *vs*, 1606 *vs*, 1511 *s*, 1466 *s*, 1374 *vs*, 1260 *vs*, 1182 *m*, 1085 *m*, 1024 *w*, 762 *m*. **MS** (EI): *m*/*z* (rel. int, %): 351 (25, [M^+]), 279 (40), 252 (25), 231 (10), 159 (55), 72 (100). **HRMS** (ESI-Q-TOF) *m*/*z* calcd for $C_{21}H_{21}NO_4$ $[M+H]^+$: 352.1543; found: 352.1534.

2-(4'-Methoxyphenyl)-3-(morpholine-4"-carbonyl)-4_H_-chromene-4-one (2m).

Yield: 88 mg (48%); Off-White solid, m.p: 180-182 °C, Eluent (Column Chromatography & TLC) [45% $CHCl_3$, 45% *n*-Hexane, 10% EtOAc, R_f : 0.2]. **^{1}H NMR** (500 MHz, $CDCl_3$) δ 8.24 (H_{Ar}, *dd*, *J* = 7.9, 1.5 *Hz*, 1H), 7.84 (H_{Ar}, *d*, *J* = 8.9 *Hz*, AA'BB', 2H), 7.73 (H_{Ar}, *td*, *J* = 8.5, 1.5 *Hz*, 1H), 7.55 (H_{Ar}, *d*, *J* = 8.4 *Hz*, 1H), 7.44 (H_{Ar}, *td*, *J* = 8.0, 0.5 *Hz*, 1H), 7.03 (H_{Ar}, *d*, *J* = 8.9 *Hz*, AA'BB', 2H), 3.91 ((-OC$\underline{H}_3$), *s*, 3H), 3.88 – 3.81 ((-HC$\underline{H}$-)$_{Morpholine}$, *m*, 1H), 3.71-3.80 ((-C$\underline{H}_2$-)$_{Morpholine}$, *m*, 2H), 3.62 – 3.56 ((-C$\underline{H}_2$-)$_{Morpholine}$, *m*, 2H), 3.37-3.40 ((-HC$\underline{H}$-)$_{Morpholine}$, *m*, 1H), 3.23 – 3.10 ((-C$\underline{H}_2$-)$_{Morpholine}$, *m*, 2H). **^{13}C{^{1}H} NMR** (125 MHz, $CDCl_3$) δ 175.3, 164.3, 162.4, 160.8, 155.9, 134.1, 129.9(2x$C_{AA'BB'}$), 126.0, 125.5, 123.8, 122.9, 117.9, 117.6, 114.3(2x$C_{AA'BB'}$), 66.5(-$\underline{C}H_2$-)$_{Morpholine}$, 66.4(-$\underline{C}H_2$-)$_{Morpholine}$, 55.5(-O$\underline{C}H_3$), 47.0(-$\underline{C}H_2$-)$_{Morpholine}$, 45.6(-$\underline{C}H_2$-)$_{Morpholine}$. **IR** (KBr, ν (cm^{-1})): 3447 *w*, 2966 *w*, 1632 *vs*, 1627 *vs*, 1615 *vs*, 1605 *vs*, 1506 *m*, 1466 *m*, 1374 *s*, 1258 *m*, 1110 *m*, 1085 *m*, 1023 *w*, 757 *m*. **MS** (EI): *m*/*z* (rel. int, %): 365 (25, [M^+]), 294 (7), 279 (90), 252 (75), 159 (100), 144 (15), 116 (20), 86 (25). **HRMS** (ESI-Q-TOF) *m*/*z* calcd for $C_{21}H_{19}NO_5$ $[M+H]^+$: 366.1336; found: 366.1328.

2-(4'-Methoxyphenyl)-3-(pyrrolidine-1-carbonyl)-4_H_-chromen-4-one (2n).

Yield: 134 mg (77 %); Colourless oil; Eluent (Column Chromatography & TLC) [45% $CHCl_3$, 45% *n*-Hexane, 10% EtOAc, R_f : 0.2]. **^{1}H NMR** (500 MHz, $CDCl_3$) δ 8.27 (H_{Ar}, *dd*, *J* = 7.9, 1.5 *Hz*, 1H), 7.89 (H_{Ar}, *d*, *J* = 8.9 *Hz*, AA'BB', 2H), 7.75 – 7.69 (H_{Ar}, *dd*, *J* = 7.9, 1.5 *Hz*, 1H), 7.55 (H_{Ar}, *d*, *J* = 8.4 *Hz*, 1H), 7.44 (H_{Ar}, *t*, *J* = 7.5 *Hz*, 1H), 7.01 (H_{Ar}, *d*, *J* = 8.9 *Hz*, AA'BB', 2H), 3.91 ((-OC$\underline{H}_3$), *s*, 3H), 3.85 – 3.77 ((-HC$\underline{H}$-)$_{Pyrrolidine}$, *m*, 1H), 3.55 – 3.45 ((-

HC$\underline{H}$-)$_{Pyrrolidine}$, *m*, 1H), 3.38 – 3.40 ((-HC$\underline{H}$-)$_{Pyrrolidine}$, *m*, 1H), 2.99 ((-HC$\underline{H}$-)$_{Pyrrolidine}$, *m*, 1H), 1.99 – 1.91 ((-HC$\underline{H}$-)$_{Pyrrolidine}$, *m*, 1H), 1.91 – 1.85 ((-HC$\underline{H}$-)$_{Pyrrolidine}$, *m*, 1H), 1.84 – 1.77 ((-HC$\underline{H}$-)$_{Pyrrolidine}$, *m*, 1H), 1.76 – 1.66 ((-HC$\underline{H}$-)$_{Pyrrolidine}$, *m*, 1H). **^{13}C{^{1}H} NMR** (125 MHz, $CDCl_3$) δ 175.3, 164.1, 162.2, 160.5, 155.9, 133.9, 129.9(2xC$_{AA'BB'}$), 126.0, 125.3, 124.0, 123.3, 119.5, 117.9, 114.3(2xC$_{AA'BB'}$), 55.4(-O$\underline{C}$H$_3$), 47.1, 45.7, 25.6, 24.5. **IR** (KBr, ν (cm^{-1})): 3447 *w*, 2964 *w*, 1618 *vs*, 1616 *vs*, 1615 *vs*, 1511 *m*, 1466 *m*, 1372 *m*, 1261 *vs*, 1181 *m*, 1103 *m*, 1023 *w*, 767 *m*. **MS** (EI): *m/z* (rel. int, %): 349 (20, [M$^+$]), 279 (50), 252 (75), 159 (75), 132 (20), 116 (10), 70 (100). **HRMS** (ESI-Q-TOF) *m/z* calcd for $C_{21}H_{19}NO_4$ [M+H]$^+$: 350.1387; found: 350.1380.

2-(4'-Methoxyphenyl)-3-(4"-methylpiperazine-1"-carbonyl)-4*H*-chromen-4-one (2o).

Yield: 78 mg (41%); Semi-solid; R$_f$ (70% EtOAc, 30% MeOH) 0.3 Eluent (Column Chromatography & TLC) [70% EtOAc, 30% MeOH, R$_f$: 0.3]. **^{1}H NMR** (500 MHz, $CDCl_3$) δ 8.23 (H$_{Ar}$, *dd*, *J* = 7.9, 1.2 *Hz*, 1H), 7.82 (H$_{Ar}$, *d*, *J* = 8.8 *Hz*, AA'BB', 2H), 7.74 – 7.69 (H$_{Ar}$, *m*, 1H), 7.53 (H$_{Ar}$, *d*, *J* = 8.4 *Hz*, 1H), 7.43 (H$_{Ar}$, *t*, *J* = 7.5 *Hz*, 1H), 7.00 (H$_{Ar}$, *d*, *J* = 8.9 *Hz*, AA'BB', 2H), 3.92-4.02 ((-HC$\underline{H}$-)$_{Piperazine}$, *m*, 1H), 3.89 (-OC$\underline{H}_3$, *s*, 3H), 3.78 – 3.67 ((-HC$\underline{H}$-)$_{Piperazine}$, *m*, 1H), 3.48 – 3.32 ((-HC$\underline{H}$-)$_{Piperazine}$, *m*, 1H), 3.29 – 3.18 ((-HC$\underline{H}$-)$_{Piperazine}$, *m*, 1H), 2.58 – 2.52 ((-HC$\underline{H}$-)$_{Piperazine}$, *m*, 1H), 2.44 – 2.37 ((-HC$\underline{H}$-)$_{Piperazine}$, *m*, 2H), 2.26 (-O-C$\underline{H}_3$, *s*, 3H), 1.96-1.98 ((-HC$\underline{H}$-)$_{Piperazine}$, *m*, 1H). **^{13}C{^{1}H} NMR** (125 MHz, $CDCl_3$) δ 175.4, 164.1, 162.3, 160.8, 155.9, 134.1, 130.0(2xC$_{AA'BB'}$), 125.9, 125.4, 123.9, 123.0, 117.9, 117.8, 114.3(2xC$_{AA'BB'}$), 55.5, 54.6, 54.1, 46.2, 45.7, 41.2. **IR** (KBr, ν (cm^{-1})): 3460 *w*, 2963 *w*, 1627 *vs*, 1624 *vs*, 1604 *vs*, 1511 *m*, 1376 *m*, 1262 *m*, 1182 *m*, 1020 *w*, 765 *m*. **MS** (EI): *m/z* (rel. int, %): 378 (5, [M$^+$]), 321 (18), 294 (25), 279 (100), 159 (38), 98 (20), 83 (75), 70 (40), 56 (25), 42 (10). **HRMS** (ESI-Q-TOF) *m/z* calcd for $C_{22}H_{22}N_2O_4$ [M+H]$^+$: 379.1652; found: 379.1650.

2-(4'-Methoxyphenyl)-3-(piperidine-1-carbonyl)-4*H*-chromen-4-one (2p).

Yield: 122 mg (67%); Colourless oil; Eluent (Column Chromatography & TLC) [90% $CHCl_3$, 9% EtOAc, 1% MeOH, R$_f$: 0.3]. **^{1}H NMR** (500 MHz, $CDCl_3$) δ 8.25 (H$_{Ar}$, *dd*, *J* = 7.9, 1.5 *Hz*, 1H), 7.88 (H$_{Ar}$, *d*, *J* = 8.9 *Hz*, AA'BB', 2H), 7.74 – 7.69 (H$_{Ar}$, *dd*, *J* = 8.5, 1.5 *Hz*, 1H), 7.54 (H$_{Ar}$, *d*, *J* = 8.3 *Hz*, 1H), 7.44 (H$_{Ar}$, *t*, *J* = 7.5 *Hz*, 1H), 7.01 (H$_{Ar}$, *d*, *J* = 8.9 *Hz*, AA'BB', 2H), 3.90 (-OC$\underline{H}_3$, *s*, *overlapped*, 3H), 3.90 – 3.85 ((-C$\underline{H}$-)$_{Piperidine}$, *m*, *overlapped*, 1H), 3.62 – 3.55 ((-HC$\underline{H}$-)$_{Piperidine}$, *m*, 1H), 3.32 – 3.20 ((-HC$\underline{H}$-)$_{Piperidine}$, *m*, 2H), 1.75 – 1.66 ((-HC$\underline{H}$-)$_{Piperidine}$, *m*, 1H), 1.58 – 1.38 ((-HC$\underline{H}$-)$_{Piperidine}$, *m*, 4H), 1.02 – 0.92 ((-HC$\underline{H}$-)$_{Piperidine}$, *m*, 1H). **^{13}C{^{1}H} NMR** (125 MHz, $CDCl_3$) δ 175.4, 163.9, 162.2, 160.1, 155.9, 133.9, 130.0(2xC$_{AA'BB'}$), 126.0, 125.3, 124.1, 123.0, 118.5, 117.9, 114.2(2xC$_{AA'BB'}$), 55.5(-O$\underline{C}$H$_3$), 47.8(-$\underline{C}$H-)$_{Piperidine}$, 42.5(-H$\underline{C}$H-)$_{Piperidine}$, 26.0(-H$\underline{C}$H-)$_{Piperidine}$, 25.2(-H$\underline{C}$H-)$_{Piperidine}$, 24.4(-

H$\underline{C}$H-)$_{Piperidine}$. **IR** (KBr, ν (cm^{-1})): 2936 *w*, 1635 *vs*, 1630 *vs*, 1629 *vs*, 1511 *m*, 1466 *m*, 1377 *m*, 1258 *m*, 1180 *m*, 1094 *w*, 1025 *w*, 761 *w*. **MS** (EI): *m/z* (rel. int, %): 363 (25, [M$^+$]), 279 (20), 269 (25), 252 (30), 159 (50), 132 (10), 84 (100), 56 (5). **HRMS** (ESI-Q-TOF) *m/z* calcd for $C_{22}H_{21}NO_4$ [M+H]$^+$: 364.1543; found: 364.1535.

N-Ethyl-2-(4'-methoxyphenyl)-4-oxo-*N*-(pyridin-4"-ylmethyl)-4*H*-chromene-3-carboxamide (2q).

Yield: 112 mg (54%); Pink oil; Eluent (Column Chromatography & TLC) [40% $CHCl_3$, 30% *n*-Hexane, 30% EtOAc, R_f : 0.2]. (***Rotameric Mixture***: **3/1**); ***Major Rotamer*: ^{1}H NMR** (500 MHz, $CDCl_3$) δ 8.61 (H_{Ar}, *d*, *J* = 5.8 *Hz*, AA'BB', 2H), 8.28 (H_{Ar}, *dd*, *J* = 8.0, 1.4 *Hz*, 1H), 7.86 (H_{Ar}, *d*, *overlapped*, *J* = 8.9 *Hz*, AA'BB', 2H), 7.79 – 7.74 (H_{Ar}, *td*, *J* = 8.0, 1.4 *Hz*, 1H), 7.61 – 7.55 (H_{Ar}, *d*, *J* = 5.8 *Hz*, AA'BB', 2H), 7.53 – 7.46 (H_{Ar}, *t*, *J* = 8.0 *Hz*, 1H), 7.01 (H_{Ar}, *d*, *overlapped*, *J* = 9.0 *Hz*, AA'BB', 2H), 5.12 ((-H_aC$\underline{H}_b$-), *d*, *J* = 16.4 *Hz*, 1H), 4.53 ((-H_bC$\underline{H}_a$-), *d*, *J* = 16.4 *Hz*, 1H), 3.91 ((-OC$\underline{H}_3$), *s*, 3H), 3.49 – 3.51 ((-H_dC$\underline{H}_c$-), *m*, 1H), 3.34 – 3.26 ((-H_cC$\underline{H}_d$-), *m*, 1H), 0.89 ((-C$\underline{H}_3$), *t*, *J* = 7.2 *Hz*, 3H). **^{13}C{^{1}H} NMR** (125 MHz, $CDCl_3$) δ 175.8, 166.3, 162.3, 160.7, 156., 148.9, 147.0(2x$C_{AA'BB'}$), 134.3, 130.1(2x$C_{AA'BB'}$), 125.9, 125.6, 123.5(2x$C_{AA'BB'}$), 122.8, 122.6, 121.1, 118.1, 114.4(2x$C_{AA'BB'}$), 55.5(-O$\underline{C}H_3$), 46.9(-$\underline{C}H_2$-), 43.8(-$\underline{C}H_2$-), 13.6(-$\underline{C}H_3$). ***Minor Rotamer*: ^{1}H NMR** (500 MHz, $CDCl_3$) δ 8.40 (H_{Ar}, *d*, *J* = 5.7 *Hz*, AA'BB', 2H), 8.22 (H_{Ar}, *dd*, *J* = 8.0, 1.4 *Hz*, 1H), 7.84 (H_{Ar}, *d*, *J* = 8.9 *Hz*, AA'BB', 2H), 7.73 – 7.69 (H_{Ar}, *td*, *J* = 8.0, 1.4 *Hz*, 1H), 7.58 (H_{Ar}, *d*, *J* = 5.7 *Hz*, AA'BB', 2H), 7.44 (H_{Ar}, *t*, *J* = 7.2 *Hz*, 1H), 7.01 (H_{Ar}, *d*, *J* = 9.0 *Hz*, AA'BB', 2H), 4.45 ((-H_bC$\underline{H}_a$-), *d*, *J* = 16.7 *Hz*, 1H), 4.21 ((-H_aC$\underline{H}_b$-), *d*, *J* = 16.7 *Hz*, 1H), 3.56 – 3.61 ((-H_dC$\underline{H}_c$-), *m*, 1H), 3.55 – 3.48 ((-H_cC$\underline{H}_d$-), *m*, 1H), 1.16 ((-C$\underline{H}_3$), *t*, *J* = 7.1 *Hz*, 3H). **^{13}C{^{1}H} NMR** (125 MHz, $CDCl_3$) δ 175.69, 165.97, 162.48, 160.57, 155.93, 150.23, 149.92, 146.61, 134.25, 130.27(2x$C_{AA'BB'}$), 125.93, 125.55, 123.95, 123.90, 122.88, 118.40, 118.35, 117.99, 114.34(2x$C_{AA'BB'}$), 55.60(-O$\underline{C}H_3$), 51.01(-$\underline{C}H_2$-), 39.93(-$\underline{C}H_2$-), 11.63(-$\underline{C}H_3$). **IR** (KBr, ν (cm^{-1})): 2968 *w*, 1618 *vs*, 1617 *vs*, 1616 *vs*, 1606 *vs*, 1511 *m*, 1466 *m*, 1376 *m*, 1259 *m*, 1181 *m*, 1024 *w*, 762 *w*. **MS** (EI): *m/z* (rel. int, %): 414 (25, [M$^+$]), 279 (85), 252 (25), 159 (70), 135 (100), 92 (10), 65 (5). **HRMS** (ESI-Q-TOF) *m/z* calcd for $C_{25}H_{22}N_2O_4$ [M+H]$^+$: 415.1652; found: 415.1634.

N-(Adamantan-1"-yl)-2-(4'-methoxyphenyl)-4-oxo-4*H*-chromene-3-carboxamide (2r).

Yield: 99 mg (46%); White solid; m.p: >250 °C, Eluent (Column Chromatography & TLC) [45% $CHCl_3$, 45% *n*-Hexane, 10% EtOAc, R_f : 0.3]. **^{1}H NMR** (500 MHz, $CDCl_3$) δ 8.23 (H_{Ar}, *dd*, *J* = 7.9, 1.2 *Hz*, 1H), 7.81 (H_{Ar}, *d*, *J* = 8.8 *Hz*, AA'BB', 2H), 7.71 (H_{Ar}, *t*, *J* = 8.5 *Hz*, *J* = 1.5 *Hz*, 1H), 7.51 (H_{Ar}, *d*, *J* = 8.4 *Hz*, 1H), 7.44 (H_{Ar}, *t*, *J* = 7.5 *Hz*, 1H), 7.19 (NH, *br s*, 1H), 7.01 (H_{Ar}, *d*, *J* = 8.8 *Hz*, AA'BB', 2H), 3.91 (-OC$\underline{H}_3$, *s*, 3H), 3.91 ($H_{Adamantyl}$, *br s*, 6H),

2.15 (H$_{Adamantyl}$, *br s*, 3H), 1.69-1.73- (H$_{Adamantyl}$, *m*, 6H). **^{13}C{^{1}H} NMR** (125 MHz, CDCl$_3$) δ 176.9, 166.0, 162.9, 162.2, 155.4, 134.0, 130.6(2xC$_{AA'BB'}$), 125.9, 125.5, 125.0, 123.5, 117.8, 117.7, 113.8(2xC$_{AA'BB'}$), 55.4(-O$\underline{C}$H$_3$), 52.6(3xC$_{Adamantyl}$), 41.4(3xC$_{Adamantyl}$), 36.4(3xC$_{Adamantyl}$), 29.5(3xC$_{Adamantyl}$). **Crystal data**: moiety formula: $C_{27}H_{27}NO_4$, M_r = 429.49 g/mol, Orthorhombic, a = 9.898 (5) Å, b = 11.606 (7) Å, c = 18.521 (11) Å, V = 2128 (2) Å^3, α = 90°, β = 90°, γ = 90°, space group: $P2_12_12_1$, Z = 4, D_{calc} = 1.341 g/cm^3, no. of reflections measured 4171, $2\theta_{max}$ = 52.3°, the refinement converged at R = 0.0780 and R_w = 0.1752 for all data (CCDC 2369639). **IR** (KBr, ν (cm^{-1})): 3320 *w*, 2921 *m*, 2851 *w*, 1668 *vs*, 1630 *vs*, 1617 *vs*, 1606 *vs*, 1507 *m*, 1466 *s*, 1383 *vs*, 1260 *vs*, 1176 *m*, 1016 *m*, 762 *s*. **MS** (EI): *m*/*z* (rel. int, %): 429 (3, [M$^+$]), 323 (25), 322 (100), 279 (50), 252 (5), 159 (40), 135 (5). **HRMS** (ESI-Q-TOF) *m*/*z* calcd for $C_{27}H_{27}NO_4$ [M+H]$^+$: 430.2013; found: 430.2000.

3-(3''-Hydroxy-8''-azabicyclo[3.2.1]octane-8''-carbonyl)-2-(4'-methoxyphenyl)-4*H*-chromen-4-one (2s).

Yield: 142 mg (70%); Fluffy semi-solid; Eluent (Column Chromatography & TLC) [40% CHCl$_3$, 40% *n*-Hexane, 20% EtOAc, R$_f$: 0.2]. (***Rotameric Mixture***: **3**/**2**); ***Major Rotamer***: **^{1}H NMR** (500 MHz, CDCl$_3$) δ 8.20 (H$_{Ar}$, *dd, overlapped, J* = 7.9, 1.4 Hz, 1H), 7.82 (H$_{Ar}$, *d, J = 8.9 Hz*, AA'BB', 2H), 7.71 (H$_{Ar}$, *td, overlapped, J* = 8.0, 1.5 *Hz*, 1H), 7.53 (H$_{Ar}$, *d, overlapped, J* = 8.4 *Hz*, 1H), 7.42 (H$_{Ar}$, *td, overlapped, J* = 7.2, 2.7 *Hz*, 1H), 6.97 (H$_{Ar}$, *d, overlapped, J* = 8.9 *Hz*, AA'BB', 2H), 4.80 – 4.72 (*m*, 1H)$_{Nortropine}$, 4.12 – 4.14 (*m*, 1H)$_{Nortropine}$, 3.88 ((-OC$\underline{H}_3$), *s*, 3H), 3.76 (*m*, 1H)$_{Nortropine}$, 2.64 (-C-OH, *br* s, *overlapped*, 1H)$_{Nortropine}$, 2.04 (*m*, 2H)$_{Nortropine}$, 1.95 (*m*, 2H)$_{Nortropine}$, 1.84 (*d, overlapped, J* = 13.3 *Hz*, 1H), 1.65 (*d, J* = 14.3 *Hz*, 1H)$_{Nortropine}$, 1.57 (*m*, 1H)$_{Nortropine}$, 0.97 – 0.87 (*m, overlapped*, 1H)$_{Nortropine}$. **^{13}C{^{1}H} NMR** (125 MHz, CDCl$_3$) δ 175.8, 162.3, 160.4, 160.3, 155.9, 134.1, 129.8(2xC$_{AA'BB'}$), 125.9, 125.4, 123.8, 123.2, 118.2, 118.0, 114.1(2xC$_{AA'BB'}$), 64.9(-$\underline{C}$-OH)$_{Nortropine}$, 55.7(-$\underline{C}$H-N-)$_{Nortropine}$, 55.5(-O$\underline{C}$H$_3$), 50.8(-$\underline{C}$H-N-)$_{Nortropine}$, 40.4(-$\underline{C}$H$_2$)$_{Nortropine}$, 39.0(-$\underline{C}$H$_2$)$_{Nortropine}$, 28.1(-$\underline{C}$H$_2$)$_{Nortropine}$, 27.3(-$\underline{C}$H$_2$)$_{Nortropine}$. ***Minor Rotamer***: **^{1}H NMR** (500 MHz, CDCl$_3$) δ 8.20 (H$_{Ar}$, *dd, overlapped, J* = 7.9, 1.4 Hz, 1H), 7.97 (H$_{Ar}$, *d, overlapped, J* = 8.9 *Hz*, AA'BB', 2H), 7.71 (H$_{Ar}$, *td, overlapped, J* = 8.0, 1.5 *Hz*, 1H), 7.53 (H$_{Ar}$, *d, overlapped, J* = 8.4 *Hz*, 1H), 7.42 (H$_{Ar}$, *td, overlapped, J* = 7.2, 2.7 *Hz*, 1H), 6.97 (H$_{Ar}$, *d, overlapped, J* = 8.9 *Hz*, AA'BB', 2H), 4.91 – 4.84 (*m*, 1H)$_{Nortropine}$, 3.87 ((-OC$\underline{H}_3$), *s*, 3H), 3.74 – 3.68 (*m*, 1H)$_{Nortropine}$, 2.64 (-C-OH, *br* s, , *overlapped*, 1H)$_{Nortropine}$, 2.35 (*t, J* = 3.9 *Hz*, 1H)$_{Nortropine}$, 2.32 (*t, J* = 3.9 *Hz*, 1H)$_{Nortropine}$, 2.26 – 2.18 (*m*, 1H)$_{Nortropine}$, 2.18 – 2.11 (*m*, 1H)$_{Nortropine}$, 2.06-2.08 (*m*, 1H)$_{Nortropine}$, 1.89 (*t, J* = 3.9 *Hz*, 1H)$_{Nortropine}$, 1.82 (*d, overlapped, J* = 10.8 *Hz*, 1H)$_{Nortropine}$, 1.47 (*d, J* = 14.3 *Hz*, 1H)$_{Nortropine}$, 0.97 – 0.87 (*m, overlapped*, 1H)$_{Nortropine}$. **^{13}C{^{1}H} NMR** (125 MHz, CDCl$_3$) δ 175.4, 162.2, 160.42, 160.36, 155.9, 134.0, 130.3(2xC$_{AA'BB'}$), 126.0, 125.3, 124.2, 123.0, 118.8, 117.9, 114.1(2xC$_{AA'BB'}$), 64.6(-$\underline{C}$-OH)$_{Nortropine}$, 55.7(-$\underline{C}$H-N-)$_{Nortropine}$, 55.5(-O$\underline{C}$H$_3$), 50.5(-$\underline{C}$H-N-

)$_{Nortropine}$, 39.5(-$\underline{C}H_2$)$_{Nortropine}$, 38.8(-$\underline{C}H_2$)$_{Nortropine}$, 28.9(-$\underline{C}H_2$)$_{Nortropine}$, 27.1(-$\underline{C}H_2$)$_{Nortropine}$. **IR** (KBr, ν (cm^{-1})): 3433 *w*, 2969 *m*, 2851 *w*, 1610 *s*, 1608 *s*, 1606 *vs*, 1511 *m*, 1465 *m*, 1378 *m*, 1261 *vs*, 1104 *s*, 1085 *s*, 1023 *s*, 804 *s*. **MS** (EI): *m/z* (rel. int, %): 405 (20, [M^+]), 279 (100), 252 (10), 159 (50), 126 (20), 68 (5). **HRMS** (ESI-Q-TOF) *m/z* calcd for $C_{24}H_{23}NO_5$ $[M+H]^+$: 406.1649; found: 406.1634.

8"-(2-(4'-Methoxyphenyl)-4-oxo-4*H*-chromene-3-carbonyl)-8"-azabicyclo[3.2.1]octan-3"-one (2t).

Yield: 103 mg (51%); Fluffy semi-solid;Eluent (Column Chromatography & TLC) [40% $CHCl_3$, 40% *n*-Hexane, 20% EtOAc, R_f : 0.2]. (***Rotameric Mixture***: **1/1**); ***Rotamer* I**: **^{1}H NMR** (500 MHz, $CDCl_3$) δ 8.24 (H_{Ar}, *d*, *overlapped*, *J* = 8.9 *Hz*, 1H), 7.86 (H_{Ar}, *d*, *J* = 8.9 *Hz*, AA'BB', 2H), 7.75 (H_{Ar}, *t*, *overlapped*, *J* = 7.7 *Hz*, 1H), 7.56 (H_{Ar}, *d*, *overlapped*, *J* = 8.5 *Hz*, 1H), 7.46 (H_{Ar}, *t*, *overlapped*, *J* = 7.5 *Hz*, 1H), 7.00 (H_{Ar}, *d*, *overlapped*, *J* = 9.0 *Hz*, AA'BB', 2H), 5.10 (*br s*, 1H)$_{Nortropinone}$, 4.12 – 4.05 (*m*, 1H)$_{Nortropinone}$, 3.89 ((-$OC\underline{H}_3$), *s*, 3H), 2.81 (*dd*, *J* = 15.6, 2.8 *Hz*, 1H)$_{Nortropinone}$, 2.38-2.46 (*m*, 1H)$_{Nortropinone}$, 2.11-2.19 (*m*, 1H)$_{Nortropinone}$, 2.02 (*d*, *J* = 15.9 *Hz*, 1H)$_{Nortropinone}$, 1.76 – 1.87 (*m*, *overlapped*, 1H)$_{Nortropinone}$, 1.57 – 1.72 (*m*, *overlapped*, 1H)$_{Nortropinone}$, 1.46– 1.55 (*m*, 1H)$_{Nortropinone}$, 1.35 – 1.22 (*m*, 1H)$_{Nortropinone}$. **^{13}C{^{1}H} NMR** (125 MHz, $CDCl_3$) δ 207.2, 175.9, 162.4, 161.5, 160.7, 155.9, 134.3, 129.8(2x$C_{AA'BB'}$), 126.0, 125.6, 123.7, 123.1, 118.14, 118.03, 114.2(2x$C_{AA'BB'}$), 55.55(-$O\underline{C}H_3$), 55.34(-$\underline{C}$H-N-)$_{Nortropinone}$, 50.8(-$\underline{C}$H-N-)$_{Nortropinone}$, 50.1(-$\underline{C}H_2$)$_{Nortropinone}$, 48.9(-$\underline{C}H_2$)$_{Nortropinone}$, 28.15(-$\underline{C}H_2$)$_{Nortropinone}$, 28.11(-$\underline{C}H_2$)$_{Nortropinone}$. ***Rotamer* II**: **^{1}H NMR** (500 MHz, $CDCl_3$) δ 8.24 (H_{Ar}, *d*, *overlapped*, *J* = 8.9 *Hz*, 1H), 7.99 (H_{Ar}, *d*, *J* = 8.9 *Hz*, AA'BB', 2H), 7.75 (H_{Ar}, *t*, *overlapped*, *J* = 7.7 *Hz*, 1H), 7.56 (H_{Ar}, *d*, *overlapped*, *J* = 8.5 *Hz*, 1H), 7.46 (H_{Ar}, *t*, *overlapped*, *J* = 7.5 *Hz*, 1H), 6.98 (H_{Ar}, *d*, *overlapped*, *J* = 9.0 *Hz*, AA'BB', 2H), 5.24 (*br s*, 1H)$_{Nortropinone}$, 4.17 – 4.12 (*m*, 1H)$_{Nortropinone}$, 3.86 ((-$OC\underline{H}_3$), *s*, 3H), 3.01 (*dd*, *J* = 15.8, 3.1 *Hz*, 1H)$_{Nortropinone}$, 2.54 (*dd*, *J* = 16.0, 3.2 *Hz*, 1H) $_{Nortropinone}$, 2.38-2.46 (*m*, 1H)$_{Nortropinone}$, 2.26-2.30 (*m*, 1H)$_{Nortropinone}$, 2.23 (*d*, *overlapped*, *J* = 15.7 *Hz*, 1H)$_{Nortropinone}$, 1.57 – 1.72 (*m*, *overlapped*, 1H)$_{Nortropinone}$, 1.57 – 1.72 (*m*, *overlapped*, 1H)$_{Nortropinone}$, 1.35 – 1.22 (*m*, 1H)$_{Nortropinone}$. **^{13}C{^{1}H} NMR** (125 MHz, $CDCl_3$) δ 206.9, 175.5, 162.5, 161.5, 160.6, 155.9, 134.3, 130.2(2x$C_{AA'BB'}$), 126.0, 125.5, 123.8, 122.9, 118.6, 118.0, 114.4(2x$C_{AA'BB'}$), 55.55(-$O\underline{C}H_3$), 55.21(-$\underline{C}$H-N-)$_{Nortropinone}$, 50.3(-$\underline{C}$H-N-)$_{Nortropinone}$, 49.1(-$\underline{C}H_2$)$_{Nortropinone}$, 48.8(-$\underline{C}H_2$)$_{Nortropinone}$, 30.2(-$\underline{C}H_2$)$_{Nortropinone}$, 29.4(-$\underline{C}H_2$)$_{Nortropinone}$. **IR** (KBr, ν (cm^{-1})): 2952 *w*, 1717 *m*, 1651 *s*, 1614 *vs*, 1606 *vs*, 1511 *m*, 1463 *m*, 1379 *m*, 1263 *m*, 1108 *m*, 1025 *w*, 764 *w*. **MS** (EI): *m/z* (rel. int, %): 403 (20, [M^+]), 279 (100), 252 (5), 159 (50), 124 (10). **HRMS** (ESI-Q-TOF) *m/z* calcd for $C_{24}H_{21}NO_5$ $[M+H]^+$: 404.1492; found: 404.1482.

2.2. Coumarin-Based *N*-Substituted-β-Enaminones (3)

Methyl (*E* or *Z*)-((2,4-dioxochroman-3-ylidene)(4-methoxyphenyl)methyl)alaninate (3a).

Yield: 8 mg (4%); Yellow oil; Eluent (Column Chromatography & TLC) [45% $CHCl_3$, 45% *n*-Hexane, 10% EtOAc, R_f : 0.5]. **^{1}H NMR** (500 MHz, $CDCl_3$) δ 14.11 (NH, *s*, 1H), 8.10 (H_{Ar}, *d*, *J* = 7.7 *Hz*, 1H), 7.59 – 7.53 (H_{Ar}, *td*, *J* = 8.5, 1.5 *Hz*, 1H), 7.25 (H_{Ar}, *t*, *J* = 7.5 Hz, 1H), 7.18 (H_{Ar}, *d*, *overlapped*, *J* = 8.0 *Hz*, 1H), 7.18 (H_{Ar}, *d*, *overlapped*, *J* = 8.5 *Hz*, AA'BB', 2H), 7.04 (H_{Ar}, *d*, *J* = 8.6 *Hz*, AA'BB', 2H), 4.23 ((-*C$\underline{H}$-CH_3)$_{Ala}$, *br s*, 1H), 3.89 ((-OC$\underline{H}_3$), *s*, 3H), 3.78 ((-OC$\underline{H}_3$), *s*, 3H), 1.54 ((-*CH-C$\underline{H}_3$)$_{Ala}$, *d*, *J* = 7.1 *Hz*, 3H). **^{13}C{^{1}H} NMR** (125 MHz, $CDCl_3$) δ 181.3, 175.0, 171.0, 162.1, 160.6, 154.2, 134.1, 127.5(2x$C_{AA'BB'}$), 126.2, 125.3, 123.6, 120.6, 116.8, 114.5(2x$C_{AA'BB'}$), 97.7, 55.3(-O$\underline{C}H_3$), 53.4(-O$\underline{C}H_3$), 53.0(-*$\underline{C}$H-CH_3)$_{Ala}$, 19.4(-*CH-$\underline{C}H_3$)$_{Ala}$. **IR** (KBr, ν (cm^{-1})): 3425 *w*, 2955 *w*, 2838 *w*, 1746 *s*, 1719 *s*, 1611 *vs*, 1561 *s*, 1466 *vs*, 1343 *m*, 1250 *m*, 1027 *w*, 836 *w*, 762 *w*. **MS** (EI): *m/z* (rel. int, %): 381 (50, [M^+]), 350 (10), 322 (96), 294 (50), 279 (100), 249 (35), 228 (25), 202 (30), 159 (75), 135 (35), 121 (25), 73 (20). **HRMS** (ESI-Q-TOF) *m/z* calcd for $C_{21}H_{19}NO_6$ $[M+H]^+$: 382.1285; found: 382.1275.

Methyl (*E* or *Z*)-((2,4-dioxochroman-3-ylidene)(4'-methoxyphenyl)methyl)valinate (3b).

Yield: 14 mg (7%); Yellowish oil; Eluent (Column Chromatography & TLC) [70% *n*-Hexane, 29 % EtOAc, 1% MeOH, R_f : 0.5]. **^{1}H NMR** (500 MHz, $CDCl_3$) δ 14.38 (NH, *d*, *J* = 6.3 *Hz*, 1H), 8.16 (H_{Ar}, *d*, *J* = 7.3 *Hz*, 1H), 7.57 (H_{Ar}, *t*, *J* = 7.4 *Hz*, 1H), 7.27 (H_{Ar}, *d*, *J* = 7.3 *Hz*, 1H), 7.20 (H_{Ar}, *d*, *J* = 8.1 *Hz*, 1H), 7.16 (H_{Ar}, *d*, *J* = 7.8 *Hz*, 1H), 7.12 (H_{Ar}, *d*, *J* = 8.0 *Hz*, 1H), 7.04 (H_{Ar}, *d*, *J* = 6.8 *Hz*, 2H), 4.10 – 3.99 ((-*C$\underline{H}$-CH(CH_3)$_2$)$_{Val}$, *m*, 1H), 3.90 ((-OC$\underline{H}_3$), *s*, 3H), 3.79 ((-OC$\underline{H}_3$), *s*, 3H), 2.30-2.36 ((C$\underline{H}$(CH_3)$_2$)$_{Val}$, *m*, 1H), 1.10 ((CH(C$\underline{H}_3$)$_2$)$_{Val}$, *d*, *J* = 6.7 *Hz*, 3H), 0.98 ((CH(C$\underline{H}_3$)$_2$)$_{Val}$, *d*, *J* = 6.9 *Hz*, 3H). **^{13}C{^{1}H} NMR** (125 MHz, $CDCl_3$) δ 182.2, 175.9, 170.1, 166.0, 160.6, 154.2, 134.1, 132.9, 127.7, 126.3, 125.3, 124.6, 123.5, 123.4, 116.8, 114.5, 97.7, 63.6, 55.3, 52.7, 31.5, 19.1, 17.7. **IR** (KBr, ν (cm^{-1})): 3468 *w*, 2964 *w*, 2839 *w*, 1744 *s*, 1721 *s*, 1610 *vs*, 1560 *s*, 1466 *vs*, 1343 *m*, 1251 *m*, 1028 *w*, 835 *w*, 762 *w*. **MS** (EI): *m/z* (rel. int, %): 409 (40, [M^+]), 394 (10), 367 (50), 350 (100), 334 (25), 307 (25), 294 (50), 279 (30), 249 (30), 159 (70), 135 (25), 121 (35), 55 (20). **HRMS** (ESI-Q-TOF) *m/z* calcd for $C_{23}H_{23}NO_6$ $[M+H]^+$: 410.1598; found: 410.1585.

(*E* or *Z*)-3-((4'-Methoxyphenyl)(phenylamino)methylene)chromane-2,4-dione (3f).

Yield: 130 mg (70%); Yellow solid, m.p: 123-125 °C; Eluent (Column Chromatography & TLC) [48% $CHCl_3$, 48% *n*-Hexane, 4% EtOAc, R_f : 0.4]. **^{1}H NMR** (500 MHz, $CDCl_3$) δ 15.50 (NH, *s*, 1H), 8.13 (H_{Ar}, *d*, *J* = 6.8 *Hz*, 1H), 7.60 (H_{Ar}, *t*, *J* = 7.8 *Hz*, 1H), 7.33 – 7.15 (H_{Ar}, *m*, 6H + *overlapped with* C<u>H</u>Cl_3 *residual peak*), 6.89 (H_{Ar}, *d*, *J* = 7.3 *Hz*, 4H), 3.84 ((-OC<u>H</u>$_3$), *s*, 3H). **^{13}C{^{1}H} NMR** (125 MHz, $CDCl_3$) δ 182.0, 172.9, 165.4, 160.9, 149.1, 140.3, 137.2, 134.3, 129.8(2xC_{Ar}), 129.0(2xC_{Ar}), 126.9, 126.2, 125.1(2xC_{Ar}), 124.9, 123.7, 116.8, 114.0(2xC_{Ar}), 97.8, 55.2(-O<u>C</u>H_3). **IR** (KBr, ν (cm^{-1})): 3421 *w*, 2962 *w*, 2851 *w*, 1716 *s*, 1610 *vs*, 1548 *vs*, 1465 *vs*, 1343 *s*, 1255 *s*, 1028 *w*, 831 *w*, 754 *w*. **MS** (EI): *m/z* (rel. int, %): 371 (100, [M^+]), 354 (80), 343 (35), 326 (5), 294 (5), 279 (60), 250 (80), 223 (90), 210 (40), 159 (45), 135 (20), 121 (25), 77 (60). **HRMS** (ESI-Q-TOF) *m/z* calcd for $C_{23}H_{17}NO_4$ $[M+H]^+$: 372.1230; found: 372.1207.

(*E* or *Z*)-3-((Benzylamino)(4'-methoxyphenyl)methylene)chromane-2,4-dione (3h).

Yield: 71 mg (37%); Yellowish oil; Eluent (Column Chromatography & TLC) [48% $CHCl_3$, 48% *n*-Hexane, 4% EtOAc, R_f : 0.4]. **^{1}H NMR** (500 MHz, $CDCl_3$) δ 14.07 (NH, *s*, 1H), 8.07 (H_{Ar}, *dd*, *J* = 7.8, 1.2 *Hz*, 1H), 7.59 – 7.54 (H_{Ar}, *td*, *J* = 7.8, 1.2 *Hz*, 1H), 7.42 – 7.32 (H_{Ar}, *m*, 3H), 7.27 – 7.19 (H_{Ar}, *m*, 6H), 7.05 (H_{Ar}, *d*, *J* = 8.6 *Hz*, 2H), 4.48 ((-C<u>H</u>$_2$-), *d*, *J* = 5.7 *Hz*, 2H), 3.90 ((-OC<u>H</u>$_3$), *s*, 3H). **^{13}C{^{1}H} NMR** (125 MHz, $CDCl_3$) δ 181.4, 175.6, 162.6, 160.6, 154.2, 135.8, 133.9, 129.1(2xC_{Ar}), 128.2, 127.8(2xC_{Ar}), 127.3(2xC_{Ar}), 126.1, 125.5, 123.5, 120.7, 116.7, 114.4(2xC_{Ar}), 97.3, 55.3(-O<u>C</u>H_3), 49.2(-<u>C</u>H_2-). **IR** (KBr, ν (cm^{-1})): 3432 *w*, 2933 *w*, 2836 *w*, 1715 *s*, 1610 *vs*, 1560 *s*, 1465 *vs*, 1342 *m*, 1250 *m*, 1028 *w*, 836 *w*, 761 *w*. **MS** (EI): *m/z* (rel. int, %): 385 (100, [M^+]), 368 (10), 322 (96), 294 (25), 249 (35), 223 (70), 208 (30), 91 (75), 65 (25). **HRMS** (ESI-Q-TOF) *m/z* calcd for $C_{24}H_{19}NO_4$ $[M+H]^+$: 386.1387; found: 386.1375.

(*E* or *Z*)-3-((4'-Methoxyphenyl)(phenethylamino)methylene)chromane-2,4-dione (3i).

Yield: 56 mg (28%); Orange solid, m.p: 105-107 °C; Eluent (Column Chromatography & TLC) [45% $CHCl_3$, 45% *n*-Hexane, 10% EtOAc, R_f : 0.5]. **^{1}H NMR** (500 MHz, $CDCl_3$) δ 13.76 (NH, *s*, 1H), 8.09 (H_{Ar}, *d*, *J* = 7.8 *Hz*, 1H), 7.58 – 7.53 (H_{Ar}, *dt*, *J* = 8.2, 1.5 *Hz*, 1H), 7.36 – 7.22 (H_{Ar}, *m*, 4H + C<u>H</u>Cl_3), 7.20 (H_{Ar}, *d*, *J* = 8.2 *Hz*, 1H), 7.11 (H_{Ar}, *d*, *J* = 7.0 *Hz*, 2H), 7.00 – 6.92 (H_{Ar}, *m*, 4H), 3.89 ((-OC<u>H</u>$_3$), *s*, 9H), 3.52 ((-C<u>H</u>$_2$-), *qd*, *J* = 6.9 *Hz*, 2H), 2.94 ((-C<u>H</u>$_2$-), *t*, *J* = 7.1 *Hz*, 2H). **^{13}C{^{1}H} NMR** (125 MHz, $CDCl_3$) δ 181.0, 175.6, 162.5, 160.4, 154.1, 137.1, 133.8, 128.8(3x$C_{AA'BB'}$), 127.6(2x$C_{AA'BB'}$), 127.1, 126.1, 125.6, 123.5, 120.8, 116.7, 114.2(2x$C_{AA'BB'}$), 97.0, 55.3(-O<u>C</u>H_3), 46.9(-<u>C</u>H_2-), 36.4(-<u>C</u>H_2-). **IR** (KBr, ν (cm^{-1})): 3318 *w*, 3025 *w*, 2959 *w*, 1660 *vs*, 1614 *s*, 1512 *s*, 1473 *s*, 1256 *s*, 1186 *s*, 1114 *w*, 771 *m*, 705

w. **MS** (EI): *m/z* (rel. int, %): 399 (36, [M+]), 308 (100), 294 (18), 251 (20), 188 (70), 159 (10), 134 (20), 121 (43), 77 (10). **HRMS** (ESI-Q-TOF) *m/z* calcd for $C_{25}H_{21}NO_4$ [M+H]+: 400.1543; found: 400.1530.

(*E* or *Z*)-3-(((Benzo[d][1,3]dioxol-5''-ylmethyl)amino)(4'-methoxyphenyl)methylene)chromane-2,4-dione (3j).

Yield: 31 mg (15%); Off-White solid, m.p: 168-170 °C; Eluent (Column Chromatography & TLC) [45% $CHCl_3$, 45% *n*-Hexane, 10% EtOAc, R_f : 0.4]. **^{1}H NMR** (500 MHz, $CDCl_3$) δ 13.98 (NH, *s*, 1H), 8.06 (H_{Ar}, *dd*, *J* = 7.9, 1.4 *Hz*, 1H), 7.58 – 7.53 (H_{Ar}, *td*, *J* = 7.9, 1.4 *Hz*, 1H), 7.27 – 7.19 (H_{Ar}, *m*, 4H), 7.06 (H_{Ar}, *d*, *J* = 8.7 *Hz*, 2H), 6.80 (H_{Ar}, *d*, *J* = 7.8 *Hz*, 1H), 6.70 – 6.64 (H_{Ar}, *m*, 2H), 6.00 ((-O-C$\underline{H}_2$-O-), *s*, 2H), 4.36 ((-C$\underline{H}_2$-), *d*, *J* = 5.8 *Hz*, 2H), 3.90 ((-OC$\underline{H}_3$), *s*, 3H). **^{13}C{^{1}H} NMR** (125 MHz, $CDCl_3$) δ 181.3, 175.3, 162.4, 160.6, 154.2, 148.3, 147.6, 133.9, 129.3, 127.8(2x$C_{AA'BB'}$), 126.1, 125.5, 123.5, 121.0, 120.7, 116.7, 114.4(2x$C_{AA'BB'}$), 108.7, 107.9, 101.3(-O-$\underline{C}H_2$-O-), 97.3, 55.3(-O$\underline{C}H_3$), 49.1(-$\underline{C}H_2$-). **IR** (KBr, ν (cm^{-1})): 3418 *w*, 2920 *w*, 2851 *w*, 1714 *s*, 1611 *vs*, 1562 *m*, 1467 *vs*, 1342 *m*, 1249 *s*, 1030 *w*, 920 *w*, 768 *w*. **HRMS** (ESI-Q-TOF) *m/z* calcd for $C_{25}H_{19}NO_6$ [M+H]+: 430.1285; found: 430.1280.

2.3. Flavone-3-Carboxylates (4)

Phenyl-2-(4'-methoxyphenyl)-4-oxo-4*H*-chromene-3-carboxylate (4a').

Yield: 164 mg (88%); Brown solid, m.p: 153-155 °C; Eluent (Column Chromatography & TLC) [48% $CHCl_3$, 48% *n*-Hexane, 4% EtOAc, R_f : 0.5]. **^{1}H NMR** (500 MHz, $CDCl_3$) δ 8.32 (H_{Ar}, *d*, *J* = 7.9 *Hz*, 1H), 7.88 (H_{Ar}, *d*, *J* = 8.9 *Hz*, AA'BB', 2H), 7.76 (H_{Ar}, *t*, *J* = 7.8 *Hz*, 1H), 7.58 (H_{Ar}, *d*, *J* = 8.3 *Hz*, 1H), 7.50 (H_{Ar}, *t*, *J* = 7.5 *Hz*, 1H), 7.41 (H_{Ar}, *t*, *J* = 7.9 *Hz*, 2H), 7.27 (H_{Ar}, *t*, *J* = 7.4 *Hz*, 1H), 7.13 (H_{Ar}, *d*, *J* = 7.6 *Hz*, 2H), 7.09 (H_{Ar}, *d*, *J* = 8.8 *Hz*, AA'BB', 2H), 3.93 ((-OC$\underline{H}_3$), *s*, 3H). **^{13}C{^{1}H} NMR** (125 MHz, $CDCl_3$) δ 174.9, 164.0, 163.2, 162.6, 155.8, 150.6, 134.3, 130.1(2xC_{Ar}), 129.4(2xC_{Ar}), 126.1, 126.1, 125.7, 124.0, 123.1, 121.5(2xC_{Ar}), 118.1, 116.8, 114.4(2xC_{Ar}), 55.6(-O$\underline{C}H_3$). **IR** (KBr, ν (cm^{-1})): 1745 *s*, 1634 *vs*, 1606 *vs*, 1469 *m*, 1386 *vs*, 1286 *vs*, 1076 *s*, 745 *m*. **MS** (EI): *m/z* (rel. int, %): 372 (1, [M+]), 279 (100), 159 (45), 144 (5), 116 (5). **HRMS** (ESI-Q-TOF) m/z calcd for $C_{23}H_{16}O_5$ [M+H]+: 373.1071; found: 373.1070.

4''-Tolyl 2-(4′-methoxyphenyl)-4-oxo-4*H*-chromene-3-carboxylate (4b′).

Yield: 143 mg (71%); White solid, m.p: 140-142 °C; Eluent (Column Chromatography & TLC) [48% $CHCl_3$, 48% *n*-Hexane, 4% EtOAc, R_f : 0.5]. **^{1}H NMR** (500 MHz, $CDCl_3$) δ 8.31 (H_{Ar}, *dd*, *J* = 7.9, 1.5 *Hz*, 1H), 7.87 (H_{Ar}, *d*, *J* = 8.8 *Hz*, AA'BB', 2H), 7.77 – 7.71 (H_{Ar}, *td*,

J = 7.9, 1.5 *Hz*, 1H), 7.56 (H_{Ar}, *d*, *J* = 8.4 *Hz*, 1H), 7.47 (H_{Ar}, *t*, *J* = 7.4 *Hz*, 1H), 7.20 (H_{Ar}, *d*, *J* = 8.3 *Hz*, AA'BB', 2H), 7.07 (H_{Ar}, *d*, *J* = 8.8 *Hz*, AA'BB', 2H), 7.02 (H_{Ar}, *d*, *J* = 8.4 *Hz*, AA'BB', 2H), 3.92 ((-OC$\underline{H}_3$), *s*, 3H), 2.36 ((-C$\underline{H}_3$), *s*, 3H). **^{13}C{^{1}H} NMR** (125 MHz, $CDCl_3$) δ 175.0, 164.2, 163.1, 162.6, 155.8, 148.4, 135.8, 134.3, 130.1(2x$C_{AA'BB'}$), 129.9(2x$C_{AA'BB'}$), 126.0, 125.7, 123.9, 123.1, 121.1(2x$C_{AA'BB'}$), 118.1, 116.8, 114.4(2x$C_{AA'BB'}$), 55.6(-O$\underline{C}H_3$), 20.9(-$\underline{C}H_3$). **IR** (KBr, ν (cm^{-1})): 1749 *s*, 1630 *vs*, 1605 *s*, 1469 *m*, 1516 *m*, 1382 *vs*, 1190 *vs*, 1075 *s*, 765 *m*. **MS** (EI): *m/z* (rel. int, %): 386 (1, [M^+]), 279 (100), 159 (40), 144 (5), 116 (4), 77 (3). **HRMS** (ESI-Q-TOF) m/z calcd for $C_{24}H_{18}O_5$ $[M+H]^+$: 387.1227; found: 387.1220.

3"-Tolyl 2-(4'-methoxyphenyl)-4-oxo-4*H*-chromene-3-carboxylate (4c′).

Yield: 110 mg (57%); White solid, m.p: 143-145 °C; Eluent (Column Chromatography & TLC) [48% $CHCl_3$, 48% *n*-Hexane, 4% EtOAc, R_f : 0.5]. **^{1}H NMR** (500 MHz, $CDCl_3$) δ 8.30 (H_{Ar}, *dd*, *J* = 7.9, 1.3 *Hz*, 1H), 7.87 (H_{Ar}, *d*, *J* = 8.8 *Hz*, AA'BB', 2H), 7.77 – 7.70 (H_{Ar}, *td*, *J* = 7.9, 1.5 *Hz*, 1H), 7.56 (H_{Ar}, *d*, *J* = 8.3 *Hz*, 1H), 7.47 (H_{Ar}, *t*, *J* = 7.5 *Hz*, 1H), 7.28 (H_{Ar}, *t*, *J* = 7.8 *Hz*, 1H), 7.07 (H_{Ar}, *d*, *J* = 8.8 *Hz*, (AA'BB' + H_{Ar}), 3H), 6.97 (H_{Ar}, *s*, 1H), 6.92 (H_{Ar}, *d*, *J* = 8.0 *Hz*, 1H), 3.91 ((-OC$\underline{H}_3$), *s*, 3H), 2.38 ((-C$\underline{H}_3$), *s*, 3H). **^{13}C{^{1}H} NMR** (125 MHz, $CDCl_3$) δ 175.0, 164.2, 163.1, 162.6, 155.8, 150.5, 139.7, 134.3, 130.1(2x$C_{AA'BB'}$), 129.1, 127.0, 126.0, 125.7, 123.9, 123.1, 122.1, 118.4, 118.1, 116.8, 114.4(2x$C_{AA'BB'}$), 55.6(-O$\underline{C}H_3$), 21.3(-$\underline{C}H_3$). **IR** (KBr, ν (cm^{-1})): 1748 *vs*, 1639 *s*, 1607 *m*, 1466 *m*, 1388 *vs*, 1261 *s*, 1077 *s*, 760 *m*. **MS** (EI): *m/z* (rel. int, %): 386 (1, [M^+]), 279 (100), 159 (40), 144 (5), 116 (4), 77 (3). **HRMS** (ESI-Q-TOF) m/z calcd for $C_{24}H_{18}O_5$ $[M+H]^+$: 387.1227; found: 387.1224.

2"-Tolyl 2-(4'-methoxyphenyl)-4-oxo-4*H*-chromene-3-carboxylate (4d′).

Yield: 103 mg (53%); White crystals, m.p: 168-170 °C; Eluent (Column Chromatography & TLC) [48% $CHCl_3$, 48% *n*-Hexane, 4% EtOAc, R_f : 0.5]. **^{1}H NMR** (500 MHz, $CDCl_3$) δ 8.33 (H_{Ar}, *dd*, *J* = 7.9, 1.4 *Hz*, 1H), 7.90 (H_{Ar}, *d*, *J* = 8.9 *Hz*, AA'BB', 2H), 7.80 – 7.74 (H_{Ar}, *td*, *J* = 8.5, 1.4 *Hz*, 1H), 7.59 (H_{Ar}, *d*, *J* = 8.3 *Hz*, 1H), 7.52 – 7.47 (H_{Ar}, *td*, *J* = 8.0, 0.5 *Hz*, 1H), 7.26 (H_{Ar}, *d*, *J* = 7.4 *Hz*, 1H), 7.16-7.24 (H_{Ar}, *m*, 2H), 7.10 (H_{Ar}, *d*, *J* = 8.9 *Hz*, AA'BB', 2H), 7.03 – 6.99 (H_{Ar}, *dd*, *J* = 7.6, 1.4 *Hz*, 1H)), 3.94 ((-OC$\underline{H}_3$), *s*, 3H), 2.26 ((-C$\underline{H}_3$), *s*, 3H). **^{13}C{^{1}H} NMR** (125 MHz, $CDCl_3$) δ 175.1, 163.9, 163.0, 162.6, 155.9, 149.2, 134.3, 131.3, 130.7, 130.2(2x$C_{AA'BB'}$), 126.8, 126.3, 126.1, 125.7, 124.1, 123.1, 121.5, 118.1, 117.3, 114.4(2x$C_{AA'BB'}$), 55.6(-O$\underline{C}H_3$), 16.2(-$\underline{C}H_3$). **IR** (KBr, ν (cm^{-1})): 1750 *s*, 1636 *vs*, 1606 *s*, 1512 *m*, 1468 *m*, 1384 *vs*, 1267 *s*, 1171 *m*, 1076 *s*, 765 *m*. **MS** (EI): *m/z* (rel. int, %): 386 (1, [M^+]), 279 (100), 253 (5), 207 (25), 159 (35), 116 (5) 73 (10). **HRMS** (ESI-Q-TOF) m/z calcd for $C_{24}H_{18}O_5$ $[M+H]^+$: 387.1227; found: 387.1210.

3'',4''-Dimethylphenyl 2-(4'-methoxyphenyl)-4-oxo-4*H*-chromene-3-carboxylate (4e′).

Yield: 138 mg (69%); White Crystals, m.p: 160-162 °C; Eluent (Column Chromatography & TLC) [48% $CHCl_3$, 48% *n*-Hexane, 4% EtOAc, R_f : 0.5]. **^{1}H NMR** (500 MHz, $CDCl_3$) δ 8.32 (H_{Ar}, *dd*, *J* = 7.9, 1.5 *Hz*, 1H), 7.89 (H_{Ar}, *d*, *J* = 8.9 *Hz*, AA'BB', 2H), 7.75 (H_{Ar}, *td*, *J* = 7.9, 1.5 *Hz*, 1H), 7.57 (H_{Ar}, *d*, *J* = 8.2 *Hz*, 1H), 7.48 (H_{Ar}, *td*, *J* = 7.5, 1.0 *Hz*, 1H), 7.15 (H_{Ar}, *d*, *J* = 8.2 *Hz*, 1H), 7.08 (H_{Ar}, *d*, *J* = 8.9 *Hz*, AA'BB', 2H), 6.92 (H_{Ar}, *d*, *J* = 2.1 *Hz*, 1H), 6.84 (H_{Ar}, *dd*, *J* = 8.1, 2.3 *Hz*, 1H), 3.93 ((-OC$\underline{H}_3$), *s*, 3H), 2.28 ((-C$\underline{H}_3$), *s*, 3H), 2.26 ((-C$\underline{H}_3$), *s*, 3H). **^{13}C{^{1}H} NMR** (125 MHz, $CDCl_3$) δ 175.0, 164.3, 163.0, 162.5, 155.8, 148.5, 137.9, 134.5, 134.3, 130.3, 130.1(2x$C_{AA'BB'}$), 126.0, 125.7, 124.0, 123.1, 122.4, 118.4, 118.1, 116.9, 114.4(2x$C_{AA'BB'}$), 55.6(-O$\underline{C}H_3$), 19.9(-$\underline{C}H_3$), 19.2(-$\underline{C}H_3$). **IR** (KBr, ν (cm^{-1})): IR (KBr, ν (cm^{-1})): 1748 *vs*, 1632 *vs*, 1605 *s*, 1514 *m*, 1468 *m*, 1381 *vs*, 1269 *m*, 1185 *s*, 1072 *vs*, 767 *m*. **MS** (EI): *m/z* (rel. int, %): 400 (1, [M^+]), 279 (100), 159 (40), 144 (5), 116 (4), 77 (3). **HRMS** (ESI-Q-TOF) m/z calcd for $C_{25}H_{20}O_5$ $[M+H]^+$: 401.1384; found: 401.1370.

Naphthalen-1''-yl-2-(4'-methoxyphenyl)-4-oxo-4*H*-chromene-3-carboxylate (4f′).

Yield: 171 mg (81%); Pale Yellow Crystals; m.p: 166-168 °C, Eluent (Column Chromatography & TLC) [48% $CHCl_3$, 48% *n*-Hexane, 4% EtOAc, R_f : 0.5]. **^{1}H NMR** (500 MHz, $CDCl_3$) δ 8.39 (H_{Ar}, *dd*, *J* = 7.9, 1.5 *Hz*, 1H), 7.98 (H_{Ar}, *d*, *J* = 9.0 *Hz*, 1H), 7.95 (H_{Ar}, *d*, *J* = 8.9 *Hz*, AA'BB', 2H), 7.87 (H_{Ar}, *d*, *J* = 7.4 *Hz*, 1H), 7.78 (H_{Ar}, *d*, *J* = 8.5 *Hz*, 2H), 7.61 (H_{Ar}, *d*, *J* = 8.3 *Hz*, 1H), 7.55 – 7.44 (H_{Ar}, *m*, 4H), 7.28 (H_{Ar}, *d*, *J* = 7.5 *Hz*, 1H), 7.11 (H_{Ar}, *d*, *J* = 8.9 *Hz*, AA'BB', 2H), 3.93 ((-OC$\underline{H}_3$, *s*, 3H). **^{13}C{^{1}H} NMR** (125 MHz, $CDCl_3$) δ 175.2, 164.2, 163.2, 162.7, 155.9, 146.5, 134.7, 134.4, 130.3(2x$C_{AA'BB'}$), 127.7, 126.8, 126.5, 126.5, 126.3, 126.1, 125.8, 125.2, 124.1, 123.2, 121.9, 118.1, 117.7, 117.4, 114.6(2x$C_{AA'BB'}$), 55.6(-O$\underline{C}H_3$). **Crystal data**: moiety formula: $C_{27}H_{18}NO_5$, M_r = 422.41 g/mol, Monoclinic, *a* = 15.086 (17) Å, *b* = 8.042 (8) Å, *c* = 17.586 (16) Å, *V* = 2005 (4) Å^3, α = 90°, β =110.03° (3), γ = 90° , space group: $P2_1/c$, Z =4 (Z′ =1), D_{calc} = 1.399 g/cm^3, no. of reflections measured 3653, $2\theta_{max}$ = 50.74°, the refinement converged at *R* = 0.0506 and R_w = 0.1424 for all data (CCDC 2369640). **IR** (KBr, ν (cm^{-1})): 1743 *s*, 1637 *s*, 1608 *m*, 1511 *m*, 1465 *m*, 1381 *vs*, 1264 *s*, 1176 *m*, 1080 *s*, 765 *vs*. **MS** (EI): *m/z* (rel. int, %): 422 (1, [M^+]), 279 (100), 159 (40), 115 (5). **HRMS** (ESI-Q-TOF) *m/z* calcd for $C_{27}H_{18}O_5$ $[M+H]^+$: 423.1227; found: 423.1211.

Naphthalen-2''-yl-2-(4'-methoxyphenyl)-4-oxo-4*H*-chromene-3-carboxylate (4g′).

Yield: 182 mg (86%); White Crystals, m.p: 168-170 °C; Eluent (Column Chromatography & TLC) [48% $CHCl_3$, 48% *n*-Hexane, 4% EtOAc, R_f : 0.5]. **^{1}H NMR**

(500 MHz, $CDCl_3$) δ 8.35 (H_{Ar}, *dd*, *J* = 7.9, 1.5 *Hz*, 1H), 7.92 (H_{Ar}, *d*, *J* = 8.8 *Hz*, AA'BB', 2H), 7.88 (H_{Ar}, *t*, *J* = 7.3 *Hz*, 2H), 7.83 (H_{Ar}, *d*, *J* = 7.7 *Hz*, 1H), 7.79 – 7.74 (H_{Ar}, *dd*, *J* = 7.9, 1.5 *Hz*, 1H), 7.59 (H_{Ar}, *d*, *J* = 8.2 *Hz*, 1H), 7.55 – 7.47 (H_{Ar}, *m*, 3H), 7.29 (H_{Ar}, *dd*, *J* = 8.9, 2.1 *Hz*, 1H), 7.11 (H_{Ar}, *d*, *J* = 8.9 *Hz*, AA'BB', 2H), 3.94 ((-OC<u>H</u>$_3$), *s*, 3H). **^{13}C{^{1}H} NMR** (125 MHz, $CDCl_3$) δ 175.0, 164.2, 163.3, 162.6, 155.8, 148.3, 134.4, 133.7, 131.7, 130.2(2x$C_{AA'BB'}$), 129.4, 127.8, 127.7, 126.6, 126.1, 125.8, 125.8, 124.0, 123.1, 120.9, 118.6, 118.1, 116.8, 114.5(2x$C_{AA'BB'}$), 55.6(-O<u>C</u>H$_3$). IR (KBr, ν (cm^{-1})): **IR** (KBr, ν (cm^{-1})): 1747 *vs*, 1642 *s*, 1607 *s*, 1507 *m*, 1465 *s*, 1389 *vs*, 1258 *s*, 1117 *m*, 1074 *s*, 758 *m*. **MS** (EI): *m/z* (rel. int, %): 422 (1, [M$^+$]), 279 (100), 159 (40), 144 (6), 115 (5). **HRMS** (ESI-Q-TOF) m/z calcd for $C_{27}H_{18}O_5$ [M+H]$^+$: 423.1227; found: 423.1206.

(4''-(Methylamino)phenyl)-2-(4'-methoxyphenyl)-4-oxo-4*H*-chromene-3-carboxylate (4h').

Yield: 100 mg (50%); Yellow Oil, Eluent (Column Chromatography & TLC) [45% $CHCl_3$, 45% *n*-Hexane, 10% EtOAc, R_f : 0.5]. **^{1}H NMR** (500 MHz, $CDCl_3$) δ 8.31 (H_{Ar}, *dd*, *J* = 7.9, 1.4 *Hz*, 1H), 7.87 (H_{Ar}, *d*, *J* = 8.8 *Hz*, AA'BB', 2H), 7.79 – 7.73 (H_{Ar}, *td*, *J* = 8.5, 1.5 *Hz*, 1H), 7.57 (H_{Ar}, *d*, *J* = 8.4 *Hz*, 1H), 7.48 (H_{Ar}, *t*, *J* = 7.5 *Hz*, 1H), 7.08 (H_{Ar}, *d*, *J* = 8.8 *Hz*, AA'BB', 2H), 6.96 (H_{Ar}, *d*, *J* = 8.8 *Hz*, AA'BB', 2H), 6.67 (H_{Ar}, *d*, *J* = 8.8 *Hz*, AA'BB', 2H), 3.93 ((-O-C<u>H</u>$_3$), *s*, 3H), 2.85 ((-N-C<u>H</u>$_3$), *s*, 3H). **^{13}C{^{1}H} NMR** (125 MHz, $CDCl_3$) δ 175.0, 164.6, 163.0, 162.5, 155.8, 146.7, 142.4, 134.2, 130.1(2x$C_{AA'BB'}$), 126.1, 125.7, 124.1, 123.1, 122.0(2x$C_{AA'BB'}$), 118.0, 117.0, 114.4(2x$C_{AA'BB'}$), 113.4(2x$C_{AA'BB'}$), 55.6(-O<u>C</u>H$_3$), 31.4(-N-<u>C</u>H$_3$). **IR** (KBr, ν (cm^{-1})): IR (KBr, ν (cm^{-1})): 3394 *w*, 1745 *s*, 1640 *s*, 1618 *s*, 1607 *s*, 1512 *vs*, 1465 *s*, 1384 *vs*, 1263 *s*, 1190 *s*, 1078 *s*, 763 *m*. **HRMS** (ESI-Q-TOF) m/z calcd for $C_{24}H_{19}NO_5$ [M+H]$^+$: 402.1336; found: 402.1320.

4''-(Hydroxymethyl)phenyl 2-(4'-methoxyphenyl)-4-oxo-4*H*-chromene-3-carboxylate (4i').

Yield: 48 mg (24%); White semi-solid, Eluent (Column Chromatography & TLC) [45% $CHCl_3$, 45% *n*-Hexane, 9% EtOAc, 1% MeOH, R_f : 0.3]. **^{1}H NMR** (500 MHz, $CDCl_3$) δ 8.31 (H_{Ar}, *dd*, *J* = 7.9, 1.4 *Hz*, 1H), 7.87 (H_{Ar}, *d*, *J* = 8.8 *Hz*, AA'BB', 2H), 7.79 – 7.75 (H_{Ar}, *td*, *J* = 7.9, 1.4 *Hz*, 1H), 7.58 (H_{Ar}, *d*, *J* = 8.3 *Hz*, 1H), 7.50 (H_{Ar}, *t*, *J* = 7.3 *Hz*, 1H), 7.40 (H_{Ar}, *d*, *J* = 8.4 *Hz*, AA'BB', 2H), 7.10 (H_{Ar}, *d*, *overlapped*, *J* = 9.0 *Hz*, AA'BB', 2H), 7.08 (H_{Ar}, *d*, *overlapped*, *J* = 9.5 *Hz*, AA'BB', 2H), 4.71 ((-C<u>H</u>$_2$-OH), *s*, 2H), 3.93 ((-O-C<u>H</u>$_3$), s, 3H). **^{13}C{^{1}H} NMR** (125 MHz, $CDCl_3$) δ 175.1, 164.1, 163.4, 162.6, 155.9, 149.9, 138.9, 134.4, 130.1(2x$C_{AA'BB'}$), 128.1(2x$C_{AA'BB'}$), 126.1, 125.8, 123.9, 123.1, 121.5(2x$C_{AA'BB'}$), 118.1, 116.7, 114.5(2x$C_{AA'BB'}$), 64.7(-<u>C</u>H$_2$-OH), 55.6(-O-<u>C</u>H$_3$). **IR** (KBr, ν (cm^{-1})): 3386 *w*, 3112 *w*, 1757 m, 1632 w, 1613 vs, 1599 vs, 1519 m, 1464 m, 1384 m, 1262 m, 1236 m, 1209 m, 1176 m,

995 vs, 838 s. **HRMS** (ESI-Q-TOF) m/z calcd for $C_{24}H_{18}O_6$ $[M+H]^+$: 403.1176 found: 403.1161.

4"-Formyl-2"-methoxyphenyl 2-(4'-methoxyphenyl)-4-oxo-4*H*-chromene-3-carboxylate (4j').

Yield: 86 mg (40%); Pale Yellow crystals, m.p: 170-173 °C; Eluent (Column Chromatography & TLC) [47% $CHCl_3$, 47% *n*-Hexane, 5% EtOAc, 1% MeOH, R_f : 0.4]. **^{1}H NMR** (500 MHz, $CDCl_3$) δ 9.99 (H̲CO, *s*, 1H), 8.32 (H_{Ar}, *dd*, *J* = 7.9, 1.5 *Hz*, 1H), 7.97 (H_{Ar}, *d*, *J* = 8.9 *Hz*, AA'BB', 2H), 7.77 (H_{Ar}, *td*, *J* = 7.9, 1.5 *Hz*, 1H), 7.58 (H_{Ar}, *d*, *J* = 8.3 *Hz*, 1H), 7.56 – 7.48 (H_{Ar}, *m*, 3H), 7.44 (H_{Ar}, *d*, *J* = 7.9 *Hz*, 1H), 7.07 (H_{Ar}, *d*, *J* = 8.9 *Hz*, AA'BB', 2H), 3.93 ((-O-C$\underline{H}_3$), *s*, 3H), 3.93 ((-O-C$\underline{H}_3$), *s*, 3H). **^{13}C{^{1}H} NMR** (125 MHz, $CDCl_3$) δ 191.0, 174.9, 163.7, 163.1, 162.6, 155.8, 152.3, 144.6, 135.4, 134.4, 130.5(2x$C_{AA'BB'}$), 126.0, 125.8, 124.8, 123.9, 123.7, 123.1, 118.1, 116.1, 114.2(2x$C_{AA'BB'}$), 110.9, 56.1(-O-$\underline{C}H_3$), 55.5(-O-$\underline{C}H_3$). **IR** (KBr, ν (cm^{-1})): 1760 *s*, 1701 *m*, 1634 *m*, 1605 *m*, 1512 *m*, 1500 *m*, 1466 *m*, 1381 *vs*, 1265 *vs*, 1185 *m*, 1066 *vs*, 1030 *m*, 734 *m*. **HRMS** (ESI-Q-TOF) m/z calcd for $C_{25}H_{18}O_7$ $[M+H]^+$: 431.1125 found: 431.1108.

3. Copies of the ^{1}H and ^{13}C{^{1}H} NMR spectra of the synthesized compounds

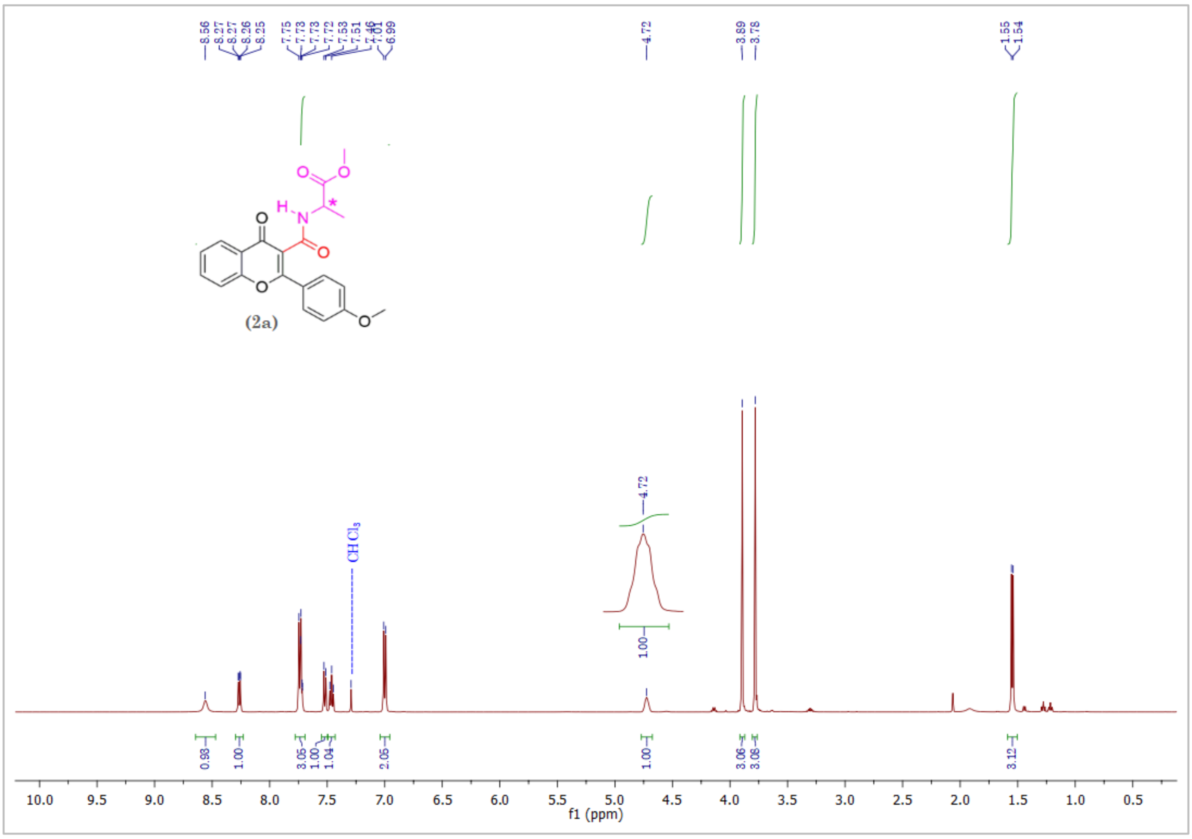

Figure S3. ^{1}H NMR spectrum of compound **2a** (500 MHz, $CDCl_3$)

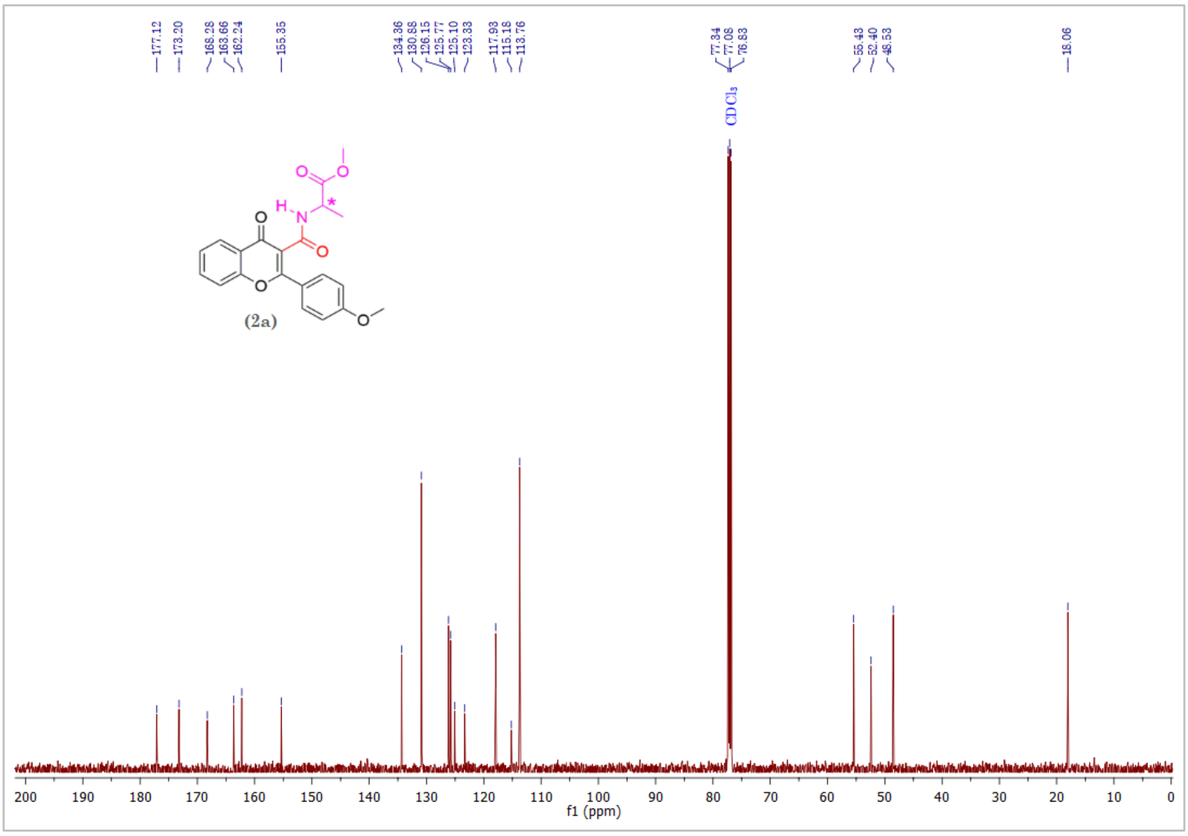

Figure S4. ^{13}C{^{1}H} NMR spectrum of compound **2a** (125 MHz, $CDCl_3$)

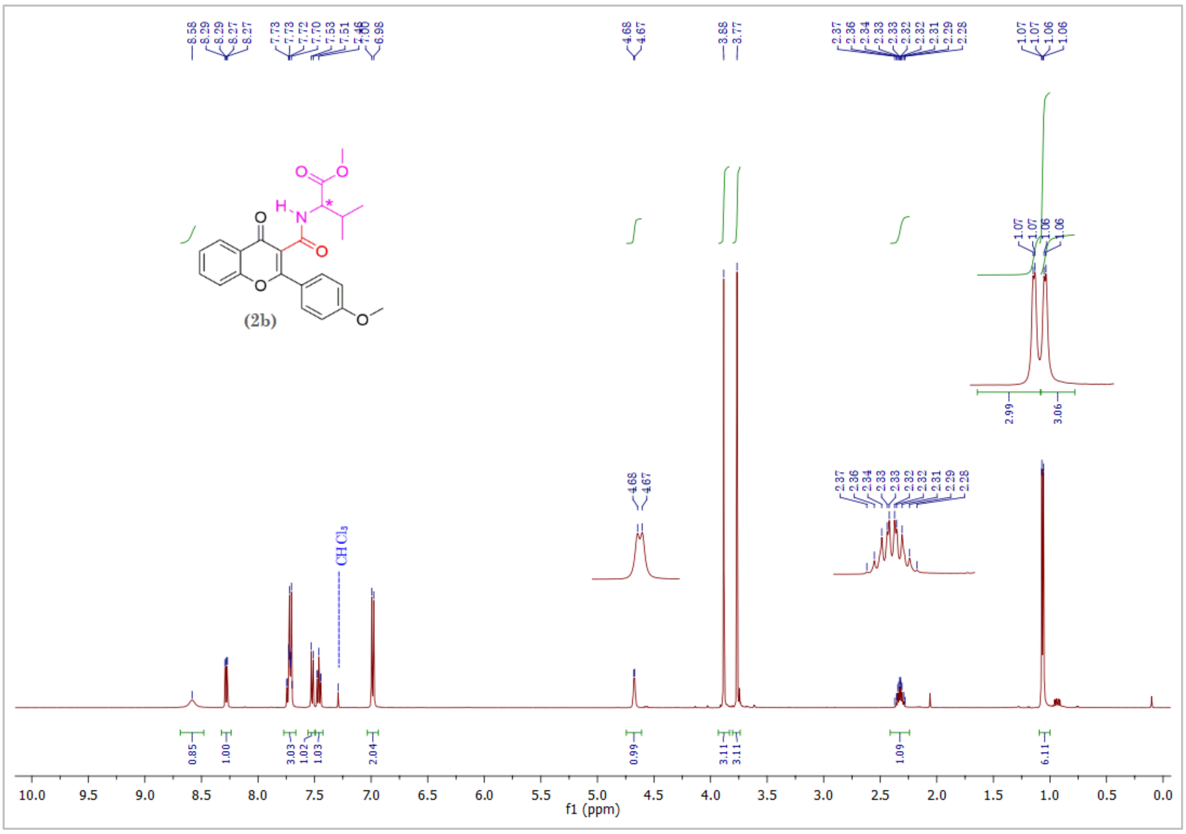

Figure S5. ^{1}H NMR spectrum of compound **2b** (500 MHz, $CDCl_3$)

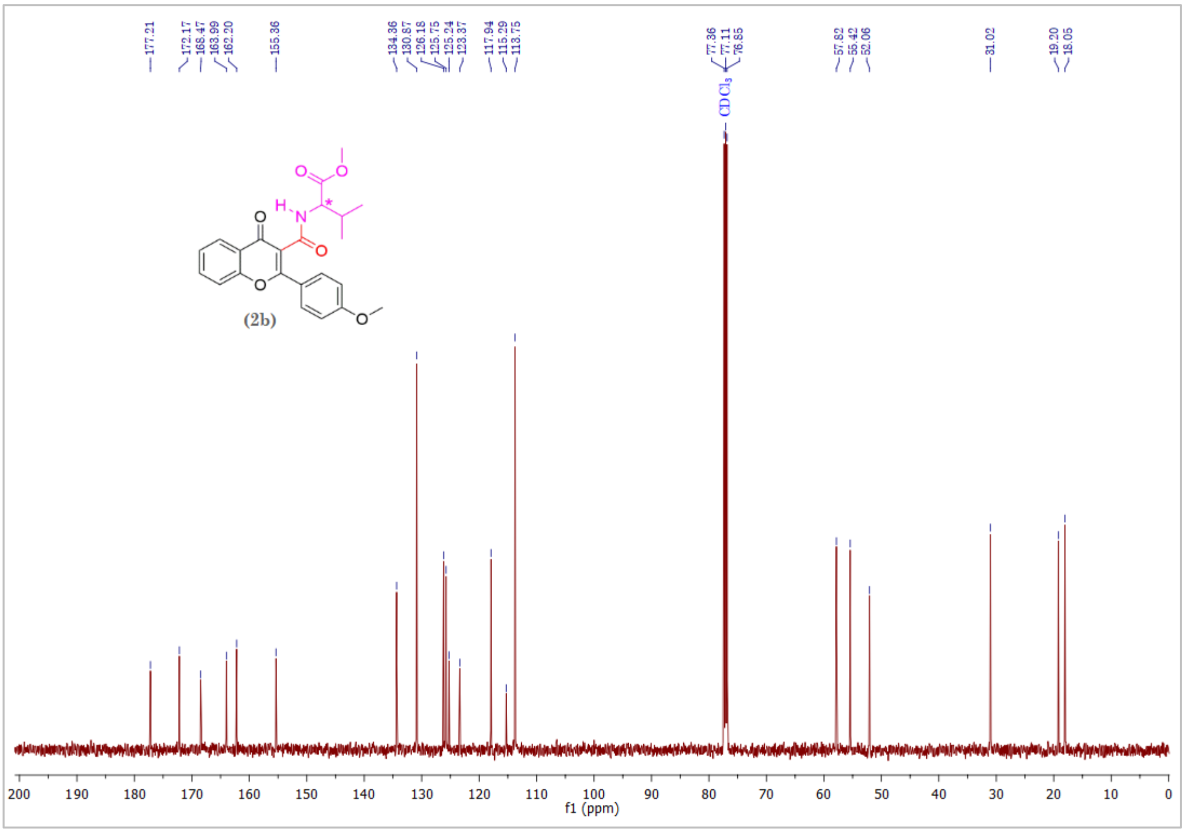

Figure S6. $^{13}C\{^{1}H\}$ NMR spectrum of compound **2b** (125 MHz, $CDCl_3$)

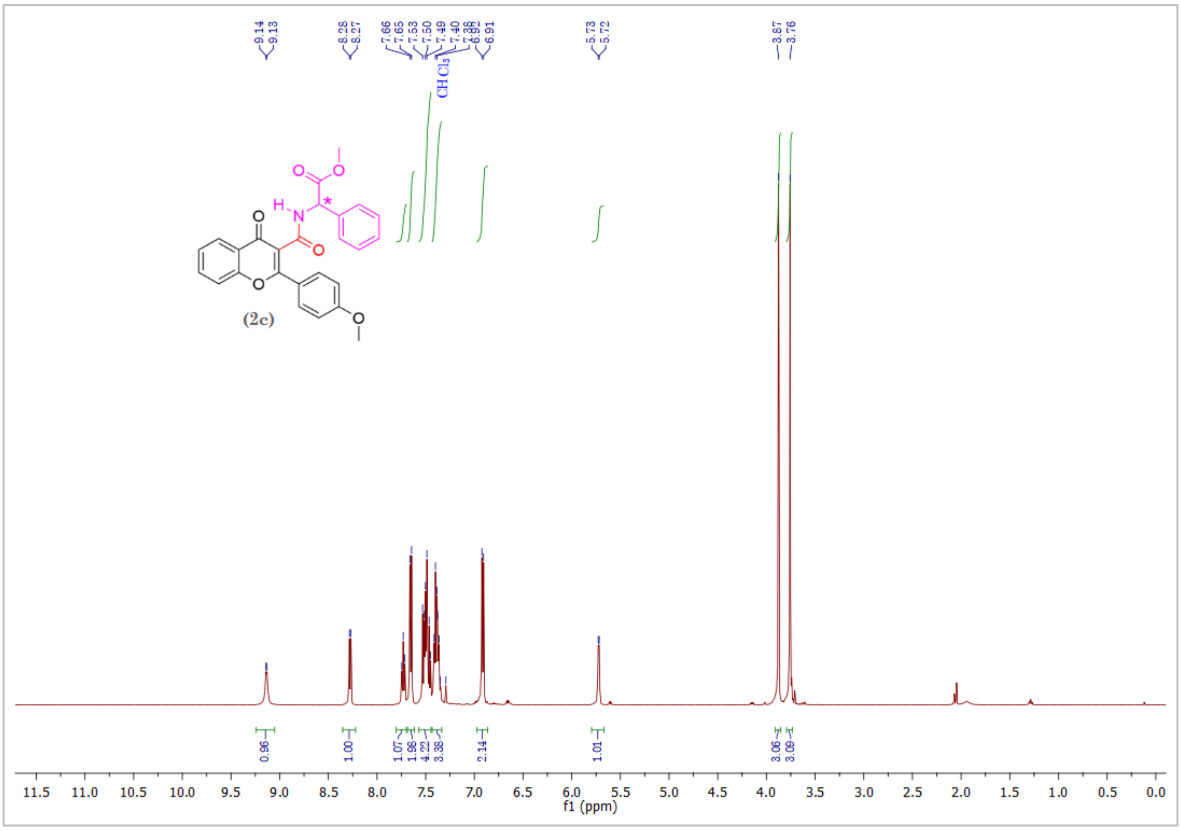

Figure S7. ^{1}H NMR spectrum of compound **2c** (500 MHz, $CDCl_3$)

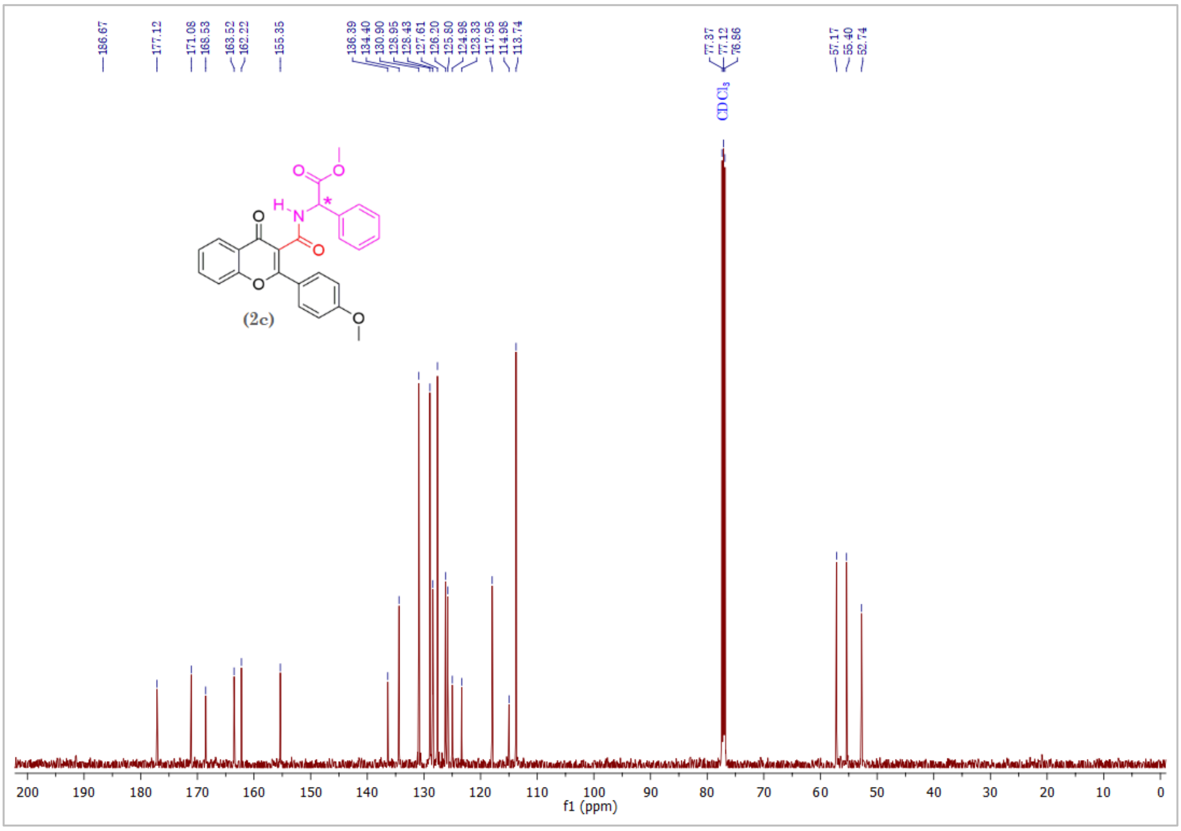

Figure S8. $^{13}C\{^{1}H\}$ NMR spectrum of compound **2c** (125 MHz, $CDCl_3$)

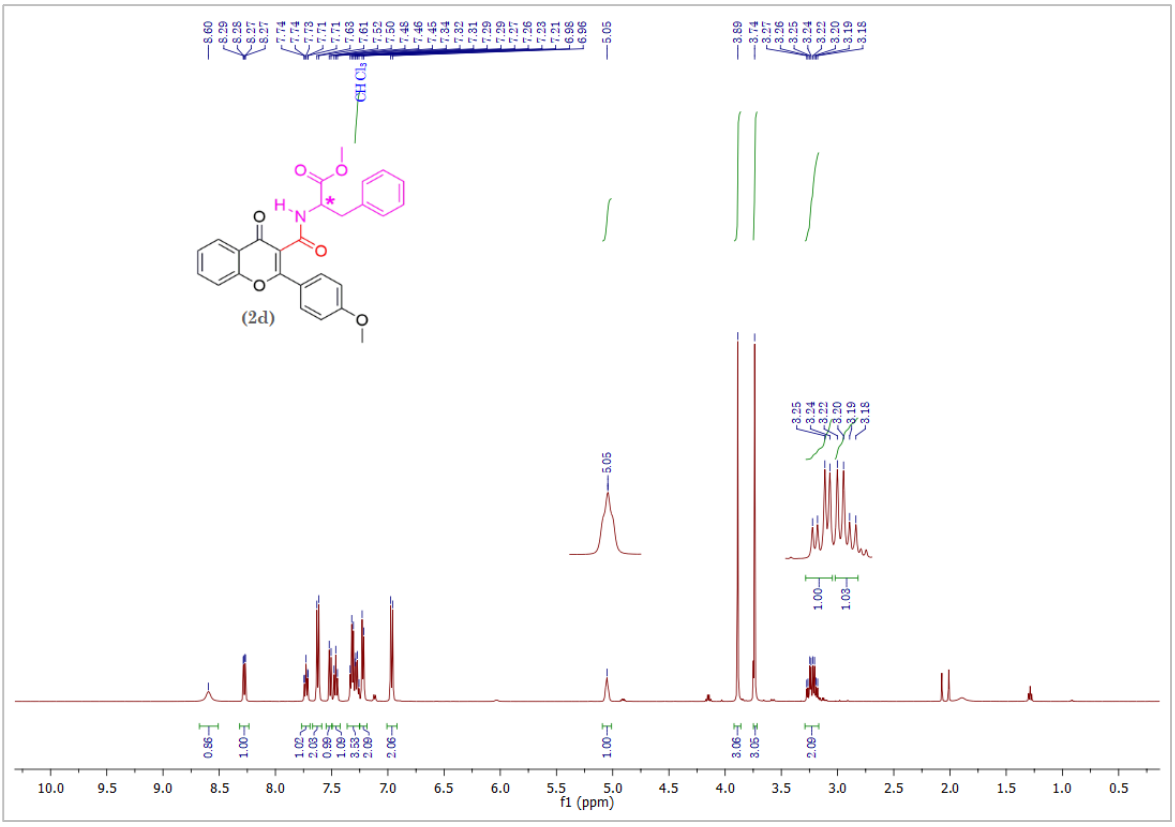

Figure S9. ^{1}H NMR spectrum of compound **2d** (500 MHz, $CDCl_3$)

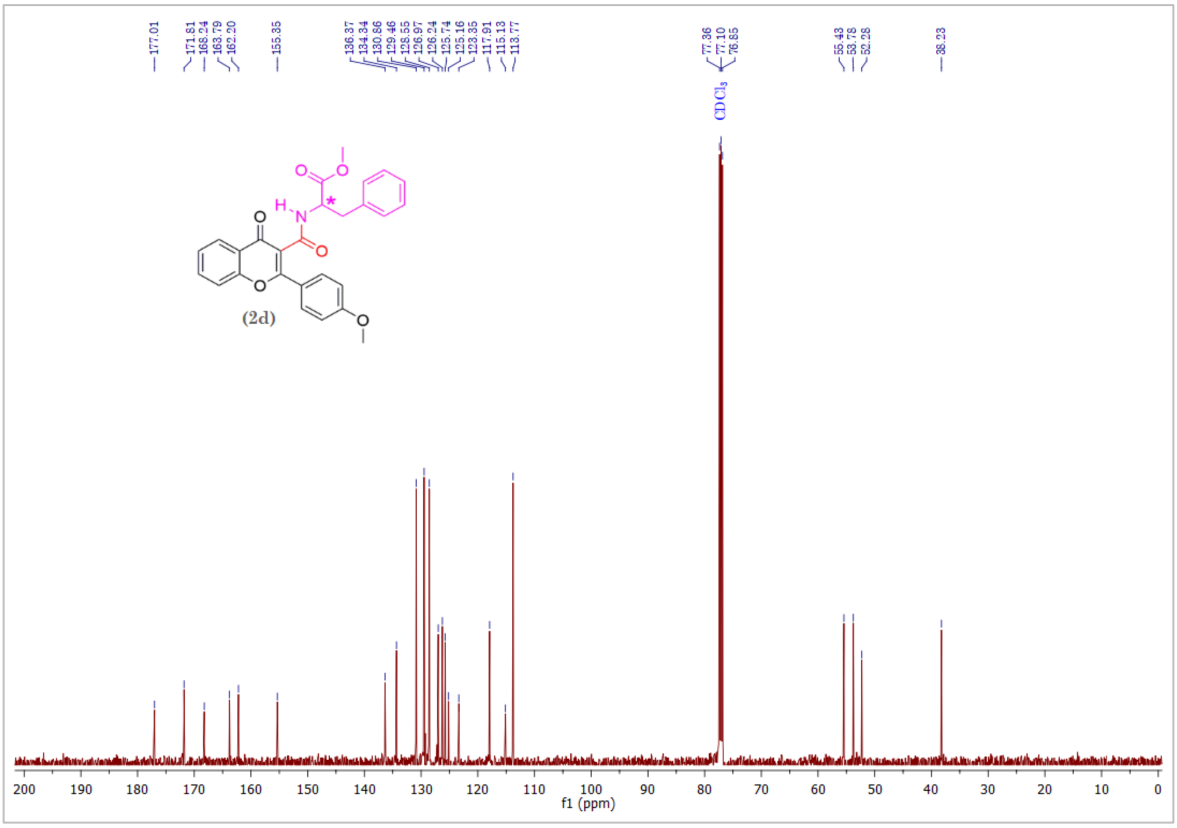

Figure S10. $^{13}C\{^1H\}$ NMR spectrum of compound **2d** (125 MHz, $CDCl_3$)

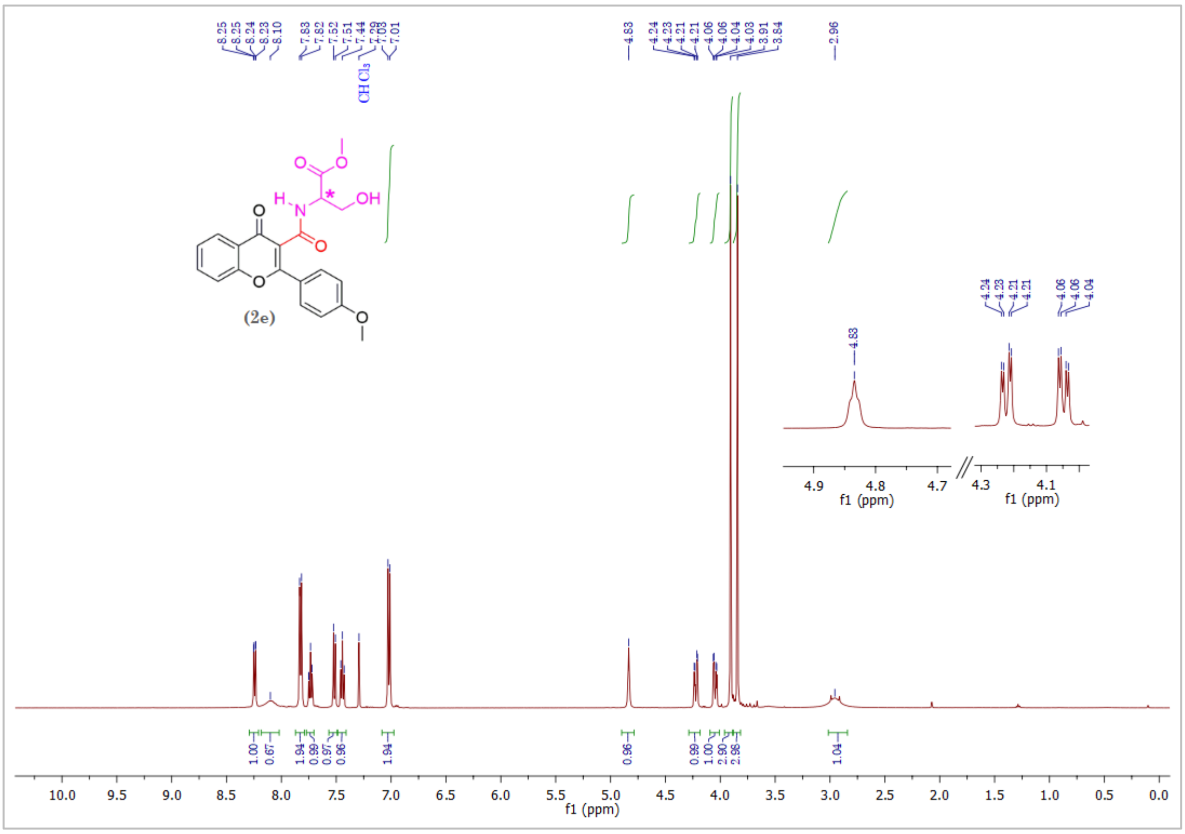

Figure S11. ^{1}H NMR spectrum of compound **2e** (500 MHz, $CDCl_3$)

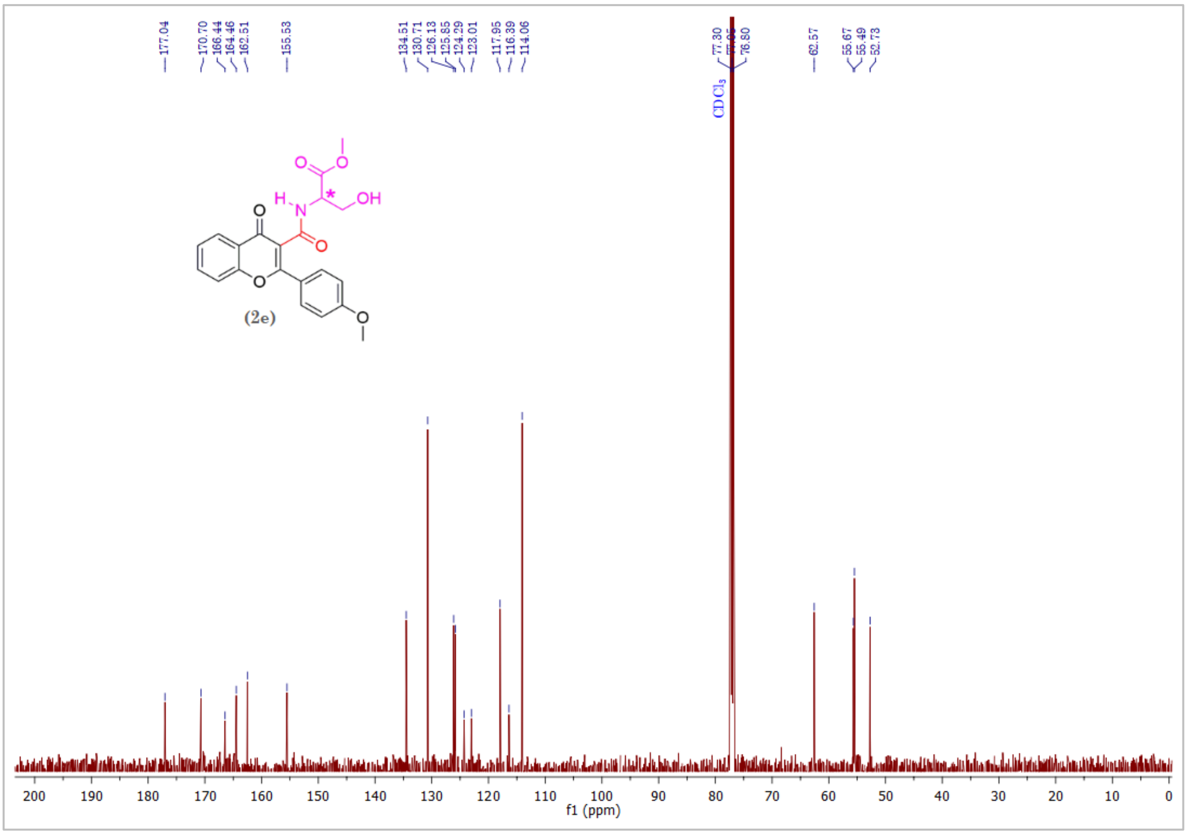

Figure S12. $^{13}C\{^{1}H\}$ NMR spectrum of compound **2e** (125 MHz, $CDCl_3$)

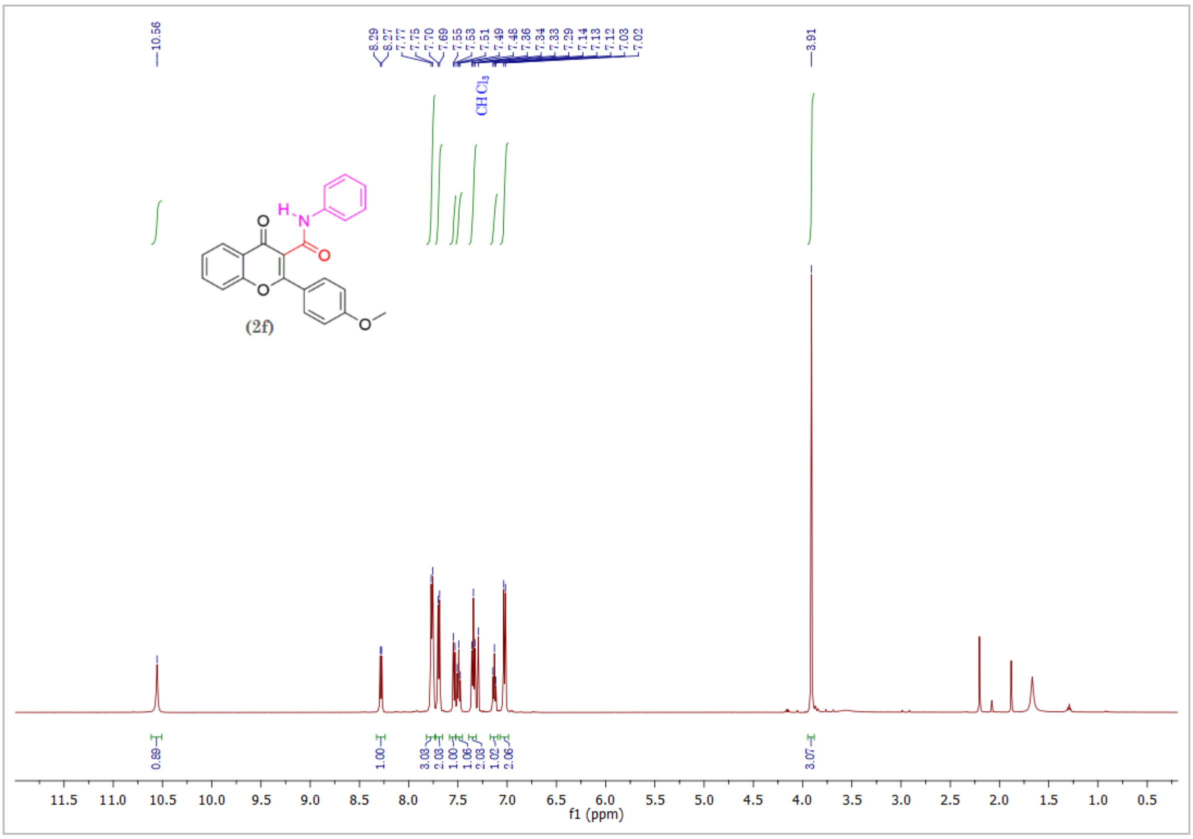

Figure S13. ^{1}H NMR spectrum of compound **2f** (500 MHz, $CDCl_3$)

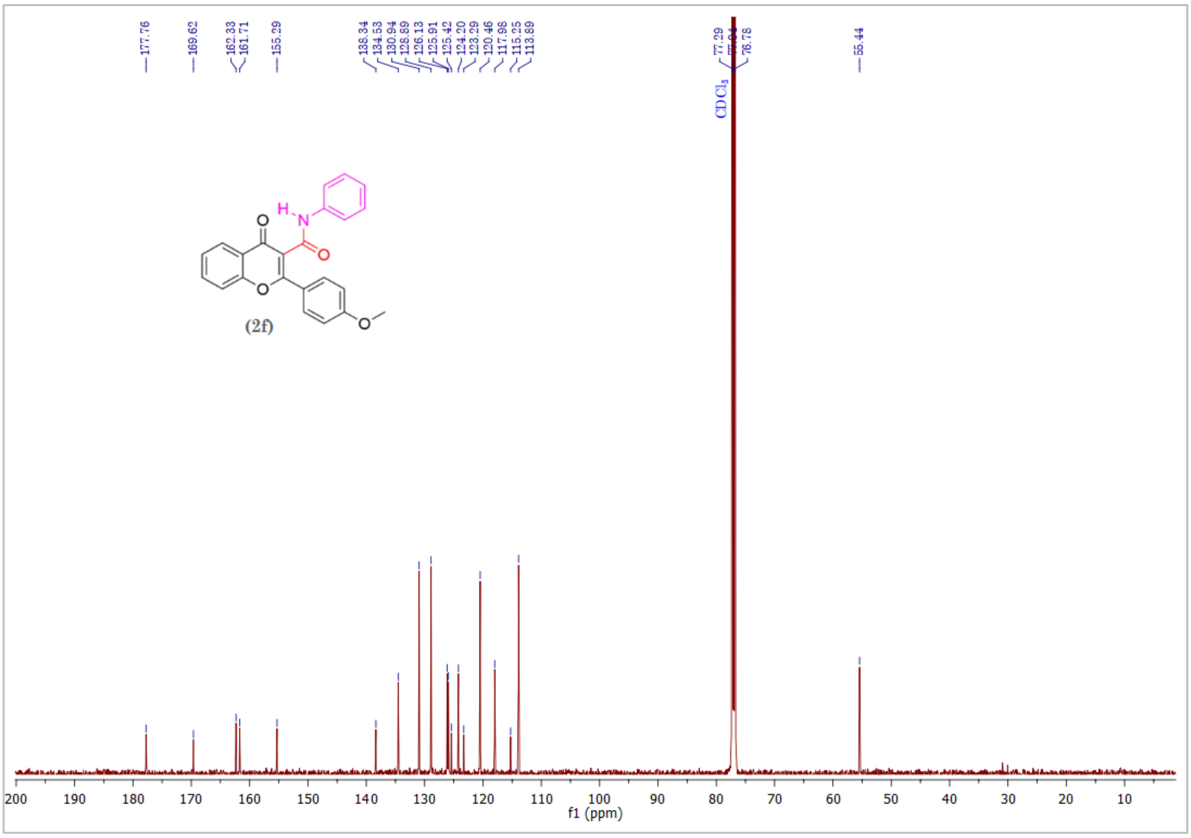

Figure S14. $^{13}C\{^{1}H\}$ NMR spectrum of compound **2f** (125 MHz, $CDCl_3$)

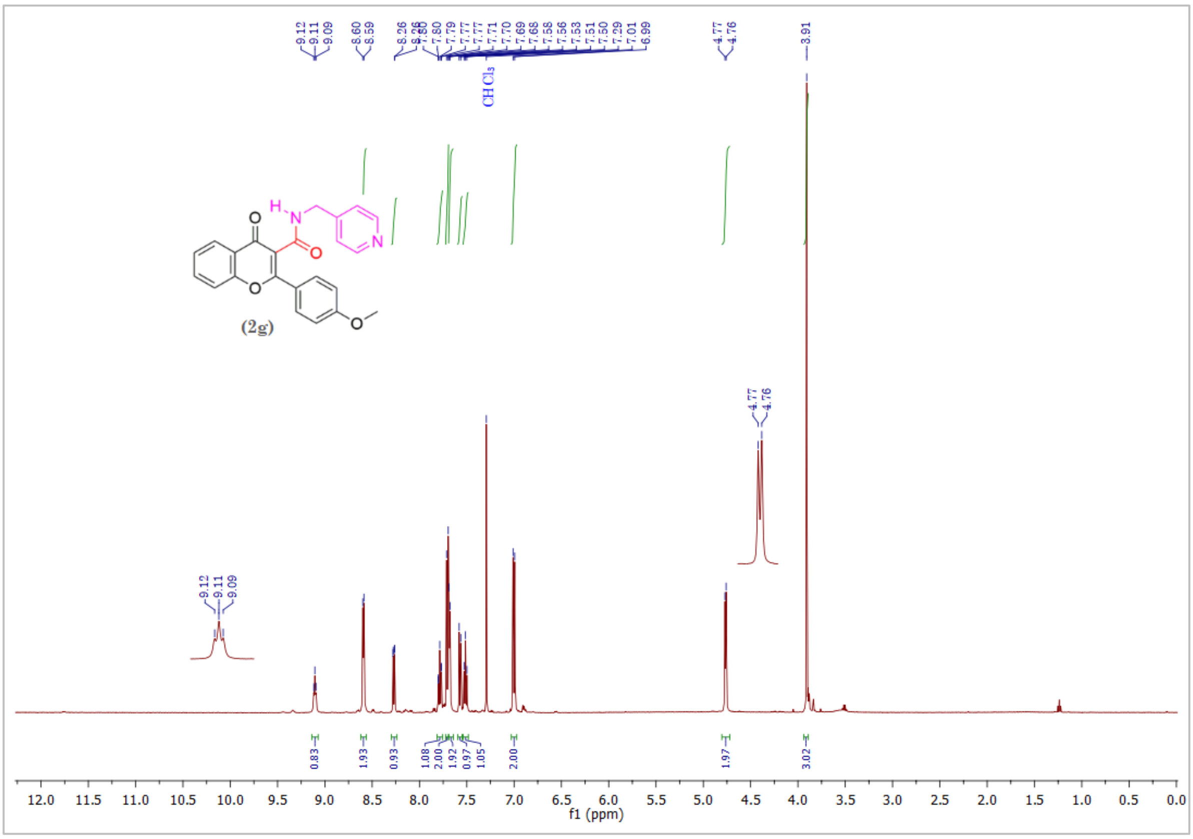

Figure S15. ^{1}H NMR spectrum of compound **2g** (500 MHz, $CDCl_3$)

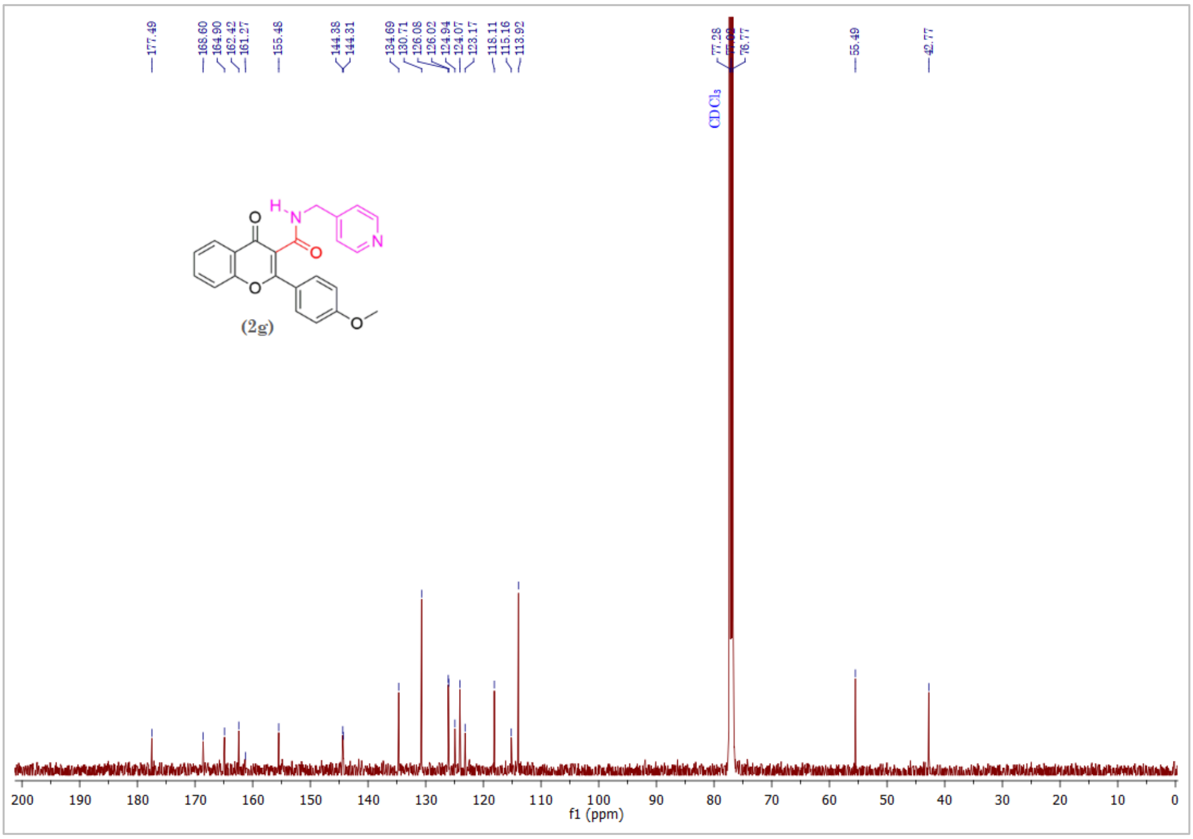

Figure S16. $^{13}C\{^{1}H\}$ NMR spectrum of compound **2g** (125 MHz, $CDCl_3$)

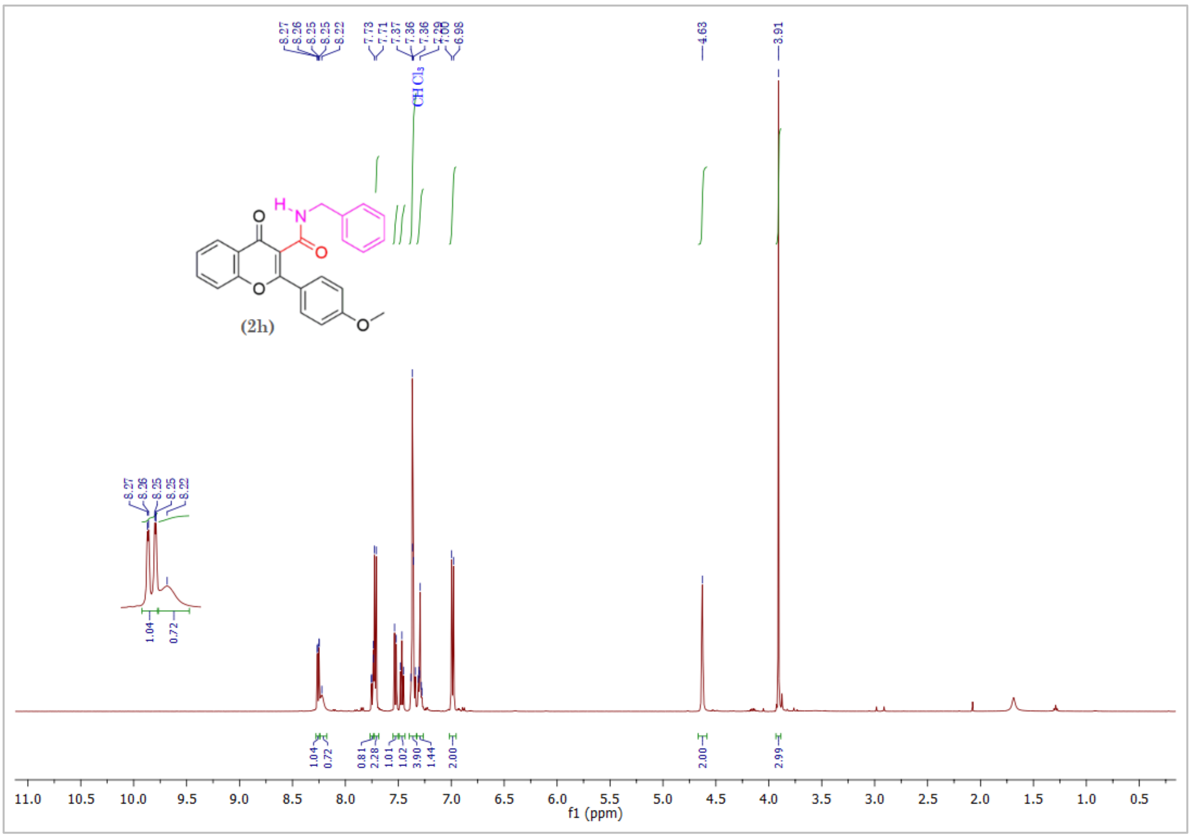

Figure S17. ^{1}H NMR spectrum of compound **2h** (500 MHz, $CDCl_3$)

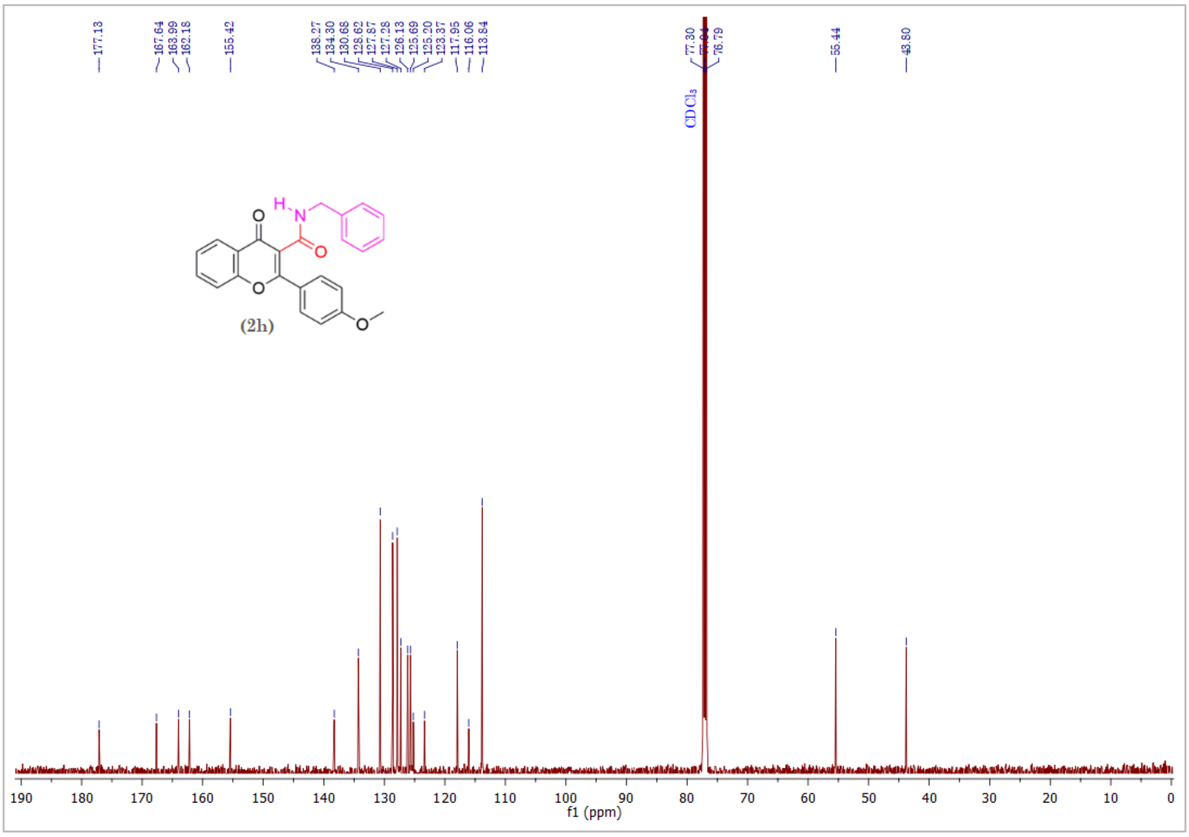

Figure S18. $^{13}C\{^{1}H\}$ NMR spectrum of compound **2h** (125 MHz, $CDCl_3$)

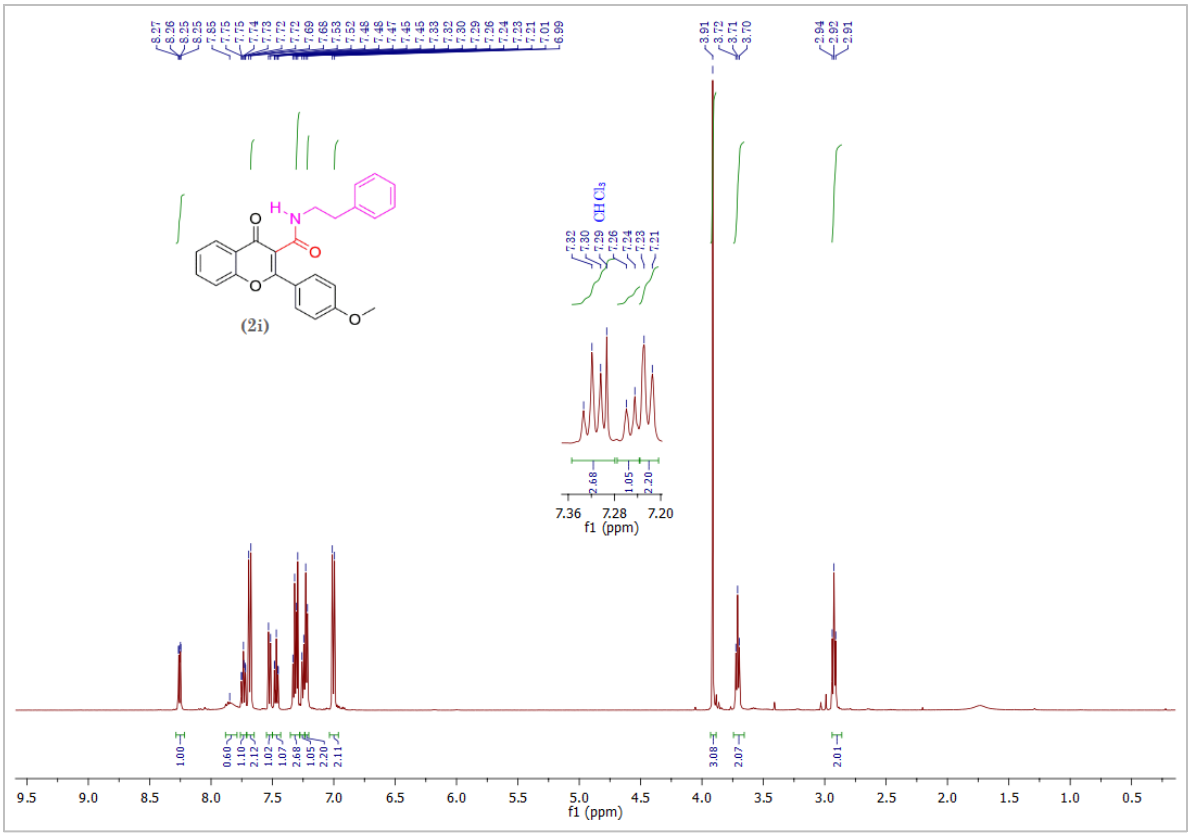

Figure S19. ^{1}H NMR spectrum of compound **2i** (500 MHz, $CDCl_3$)

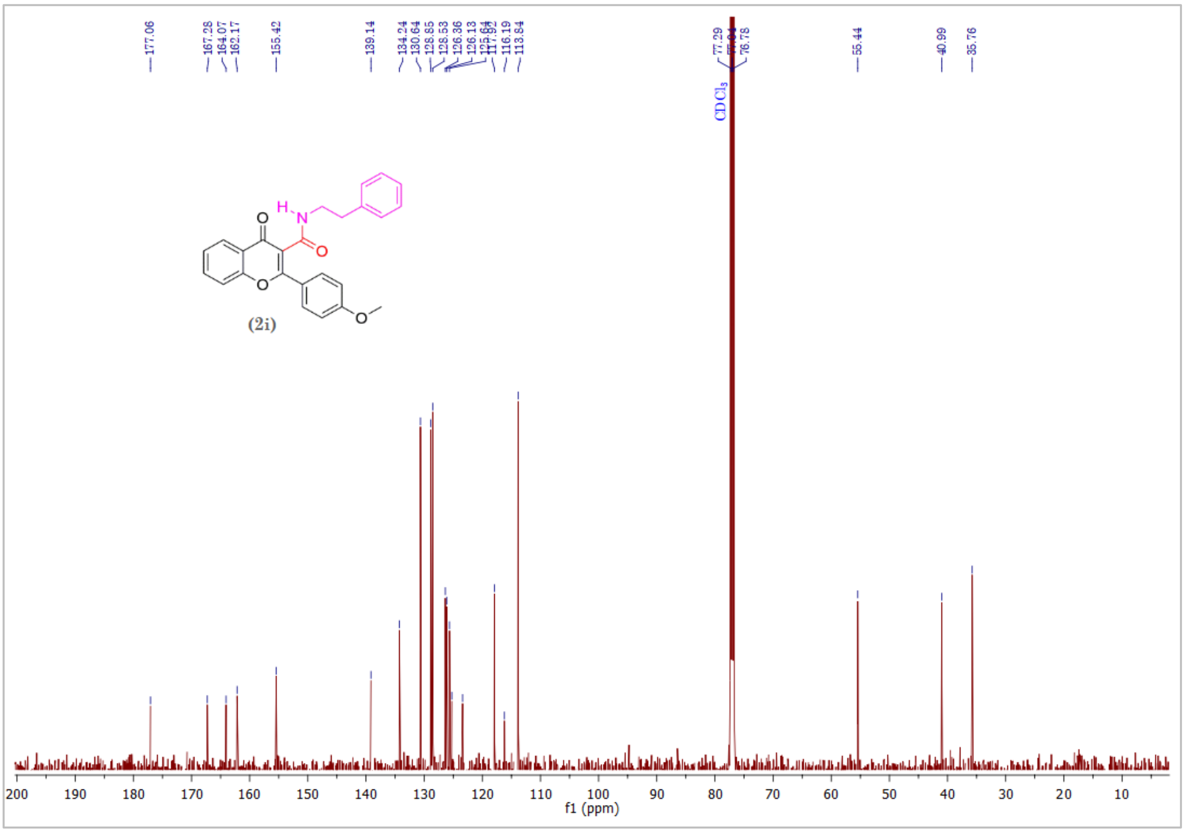

Figure S20. $^{13}C\{^1H\}$ NMR spectrum of compound **2i** (125 MHz, $CDCl_3$)

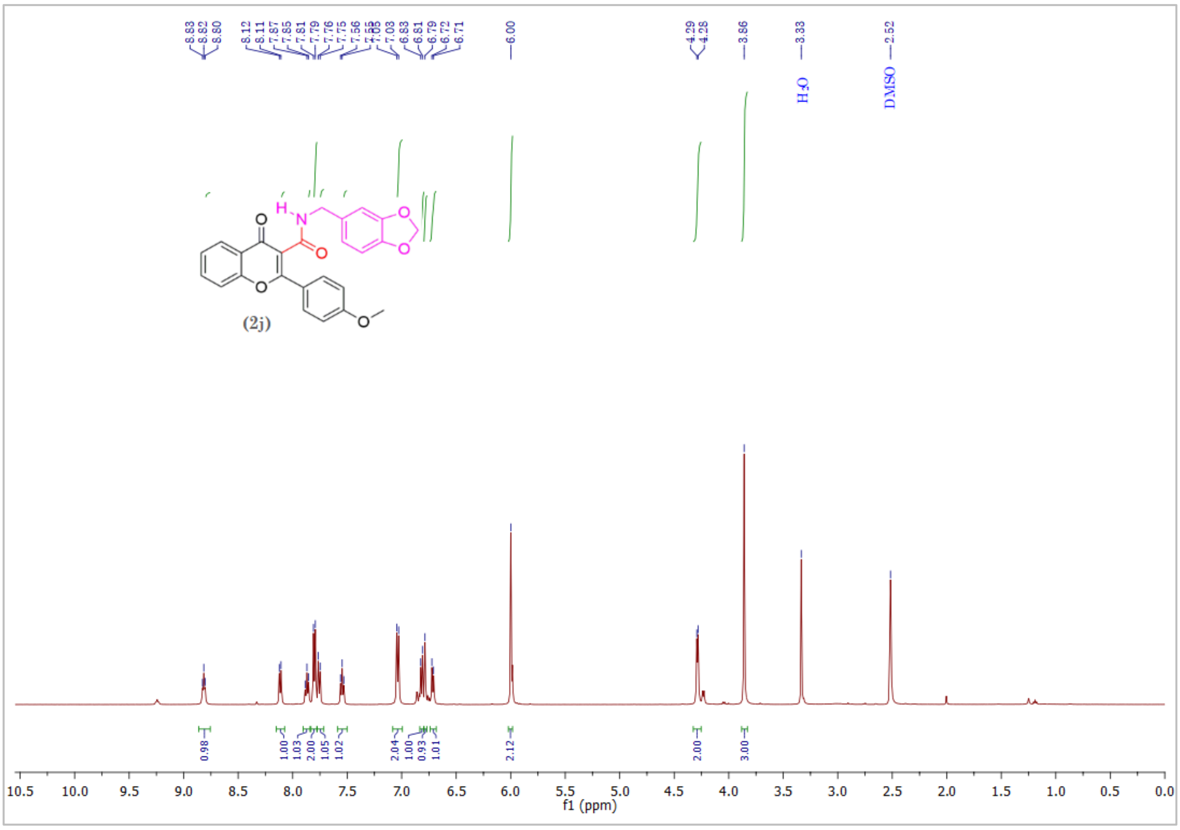

Figure S21. ^{1}H NMR spectrum of compound **2j** (500 MHz, DMSO-d6)

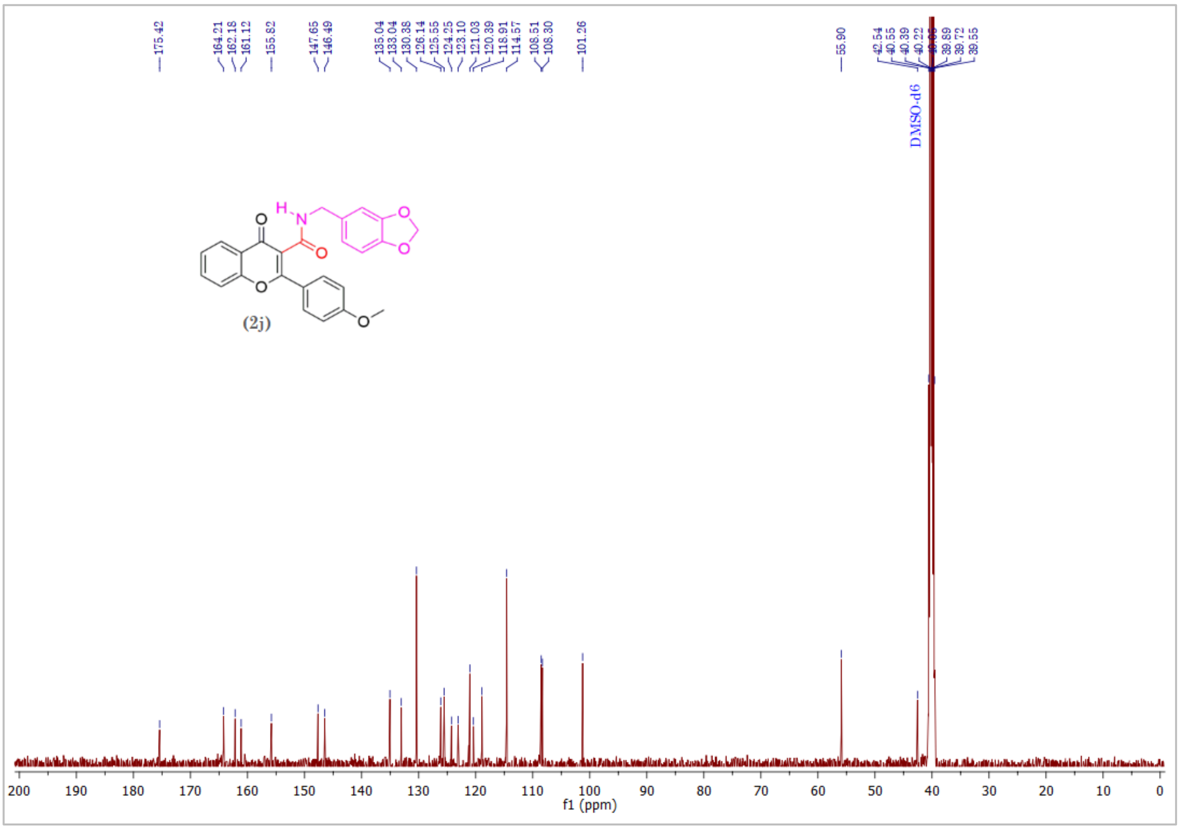

Figure S22. $^{13}C\{^{1}H\}$ NMR spectrum of compound **2j** (125 MHz, DMSO-d6)

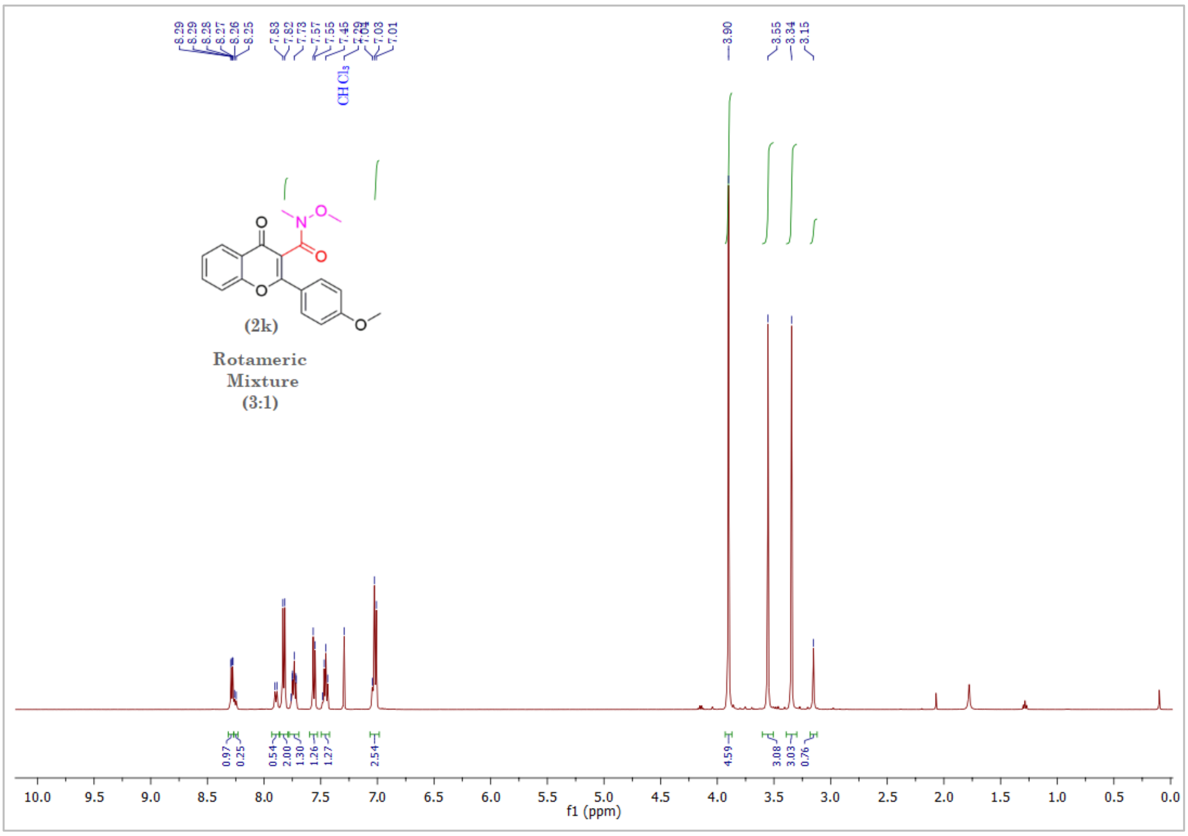

Figure S23. ^{1}H NMR spectrum of compound **2k** (500 MHz, $CDCl_3$)

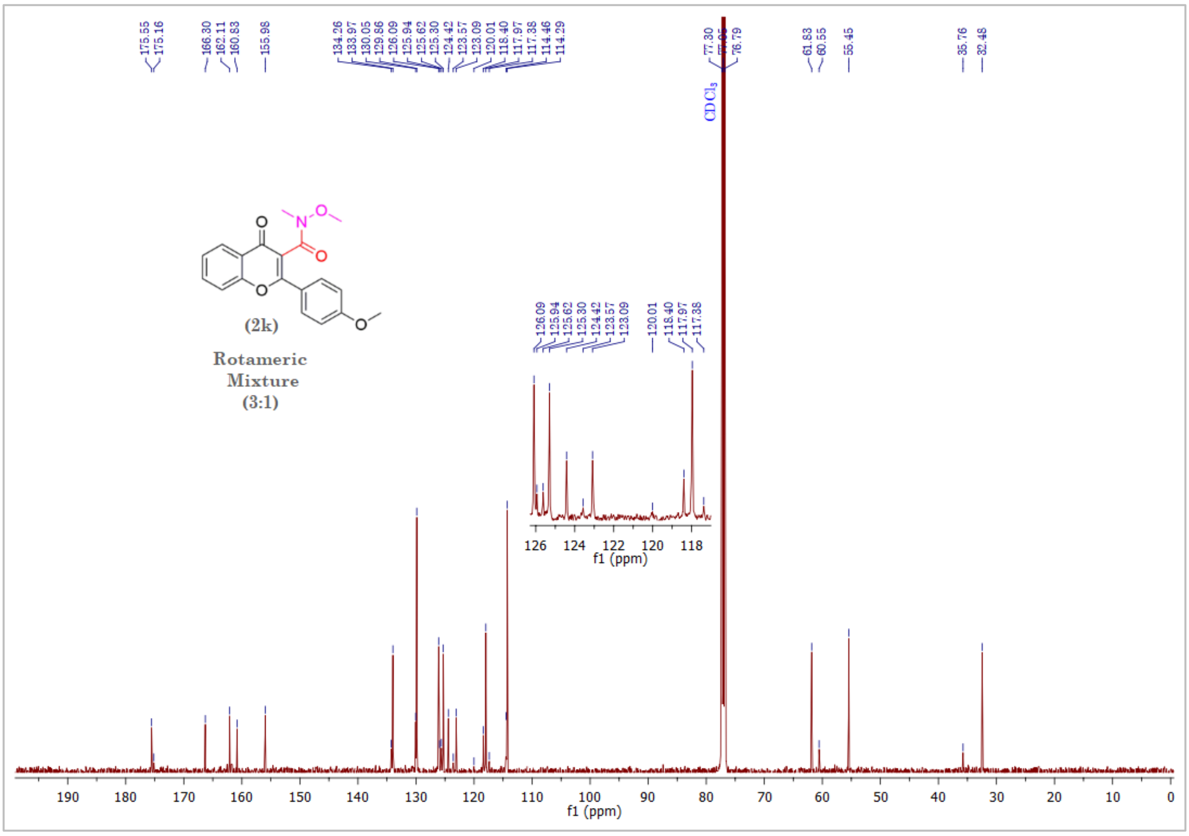

Figure S24. $^{13}C\{^{1}H\}$ NMR spectrum of compound **2k** (125 MHz, $CDCl_3$)

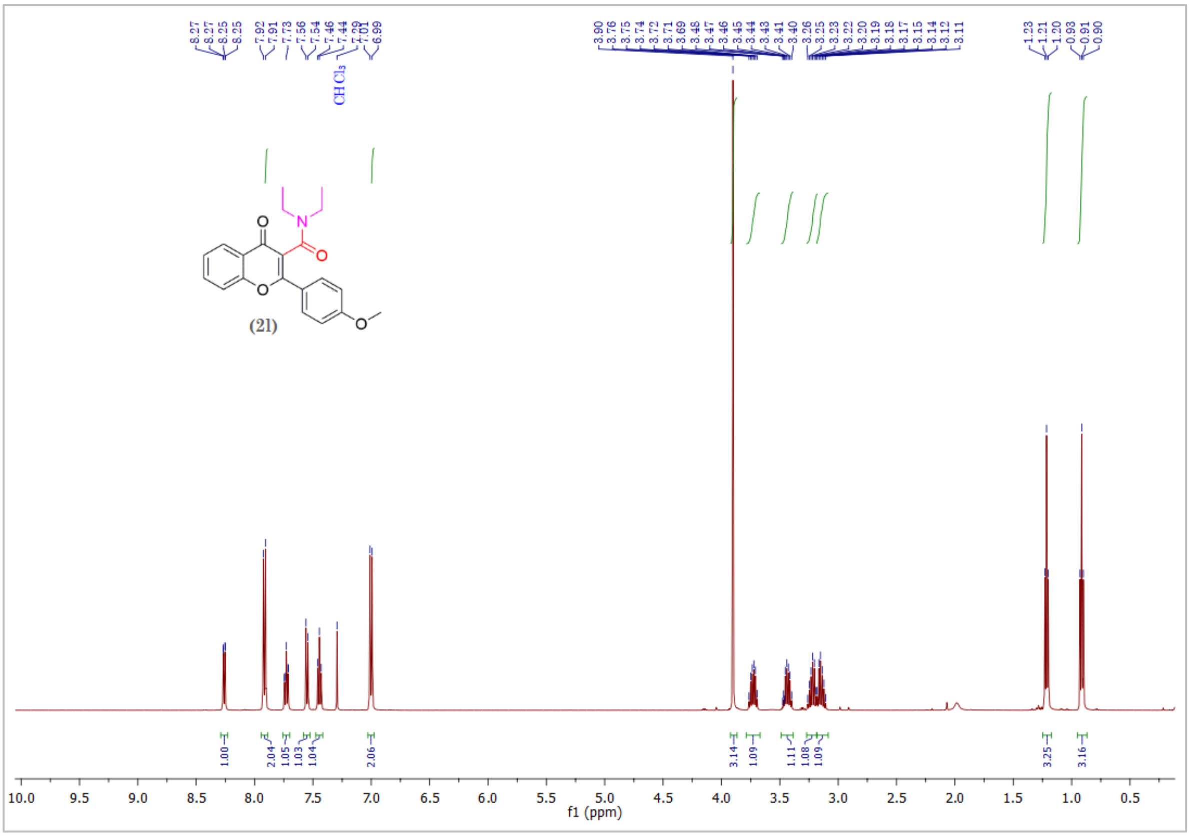

Figure S25. ^{1}H NMR spectrum of compound **2l** (500 MHz, $CDCl_3$)

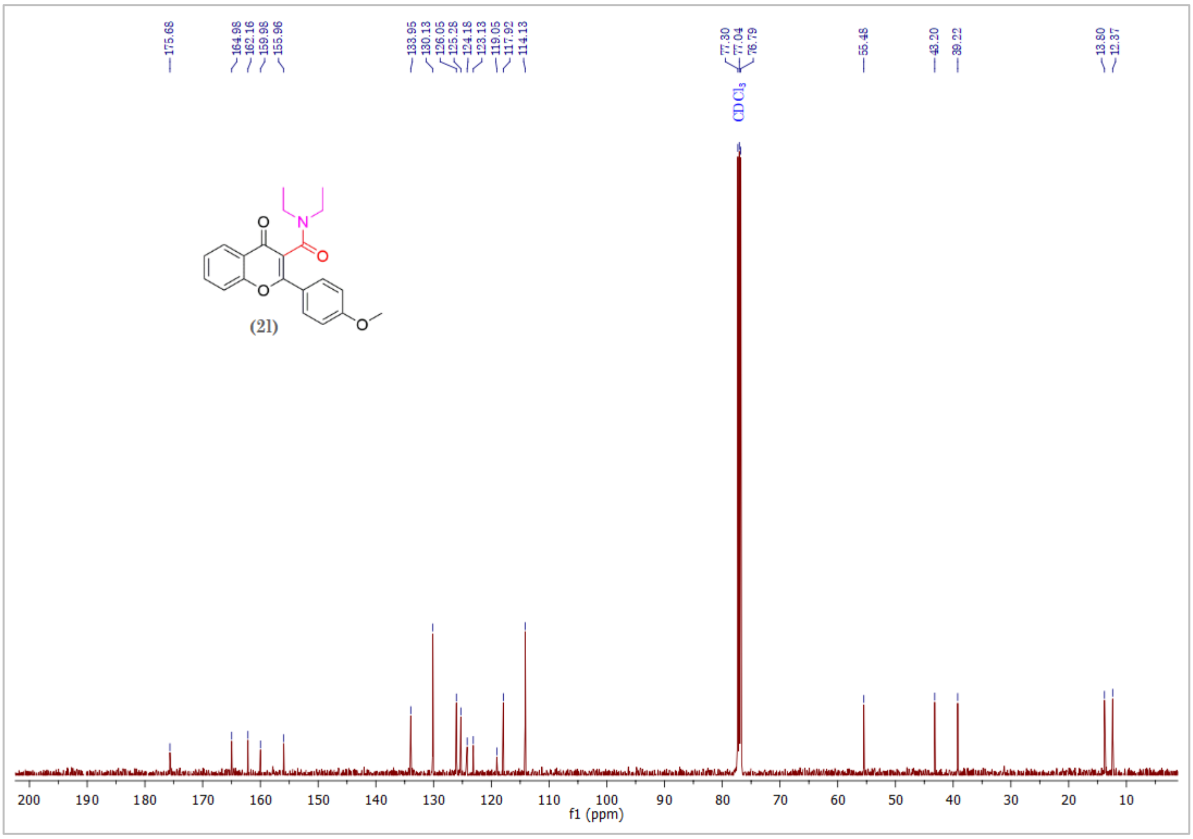

Figure S26. ^{13}C{^{1}H} NMR spectrum of compound **2l** (125 MHz, $CDCl_3$)

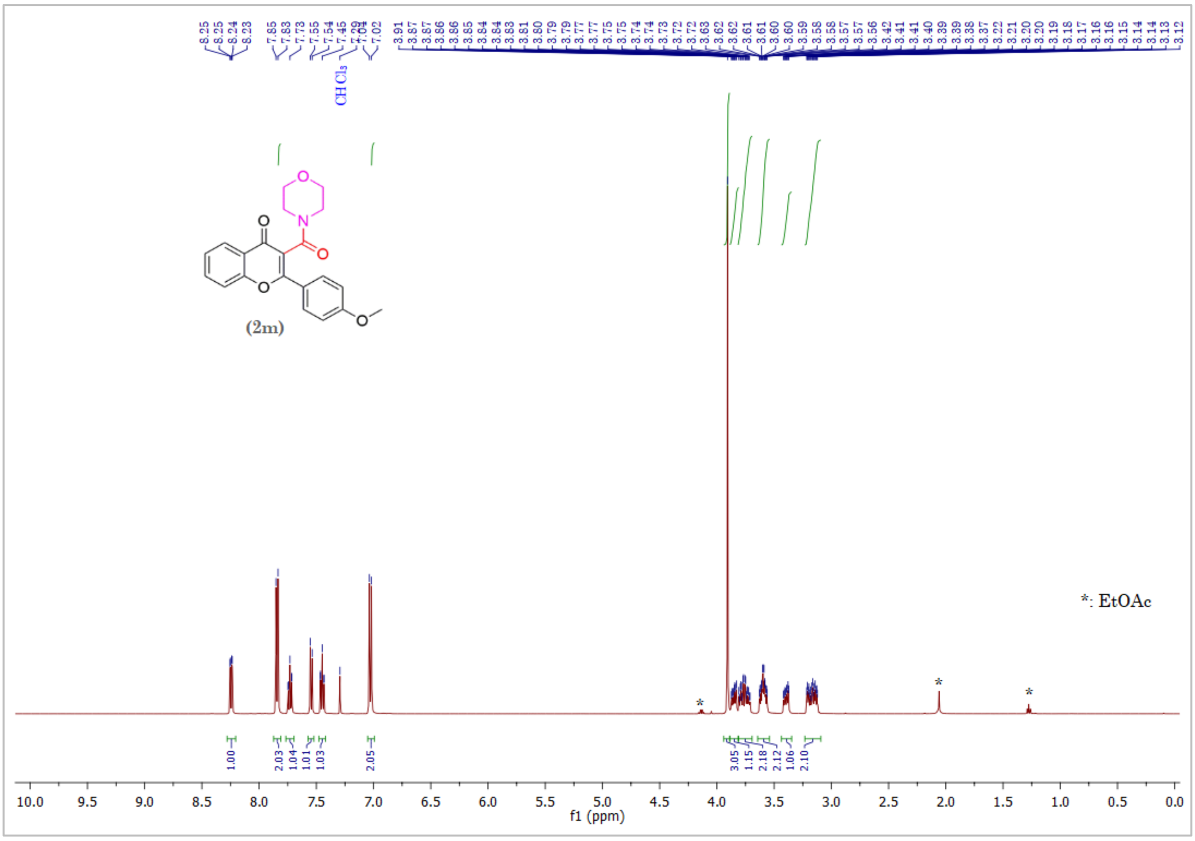

Figure S27. ^{1}H NMR spectrum of compound **2m** (500 MHz, $CDCl_3$)

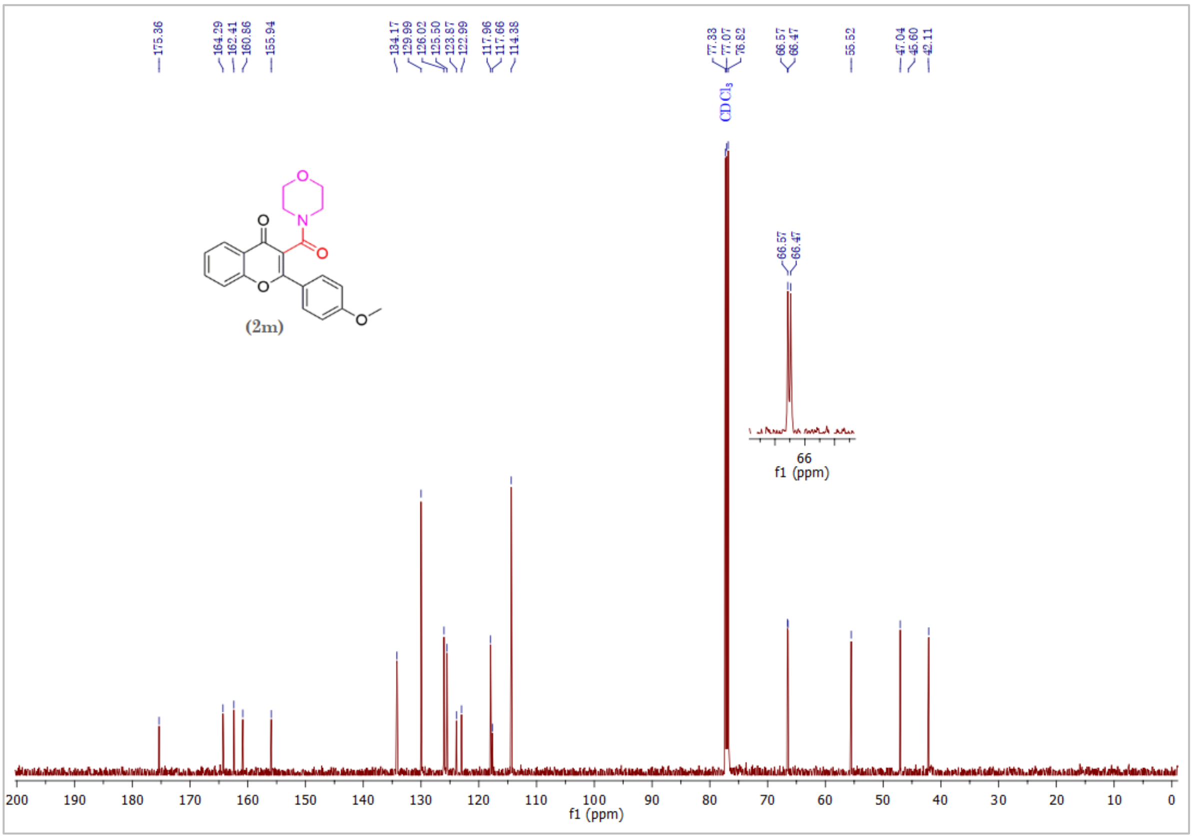

Figure S28. $^{13}C\{^{1}H\}$ NMR spectrum of compound **2m** (125 MHz, $CDCl_3$)

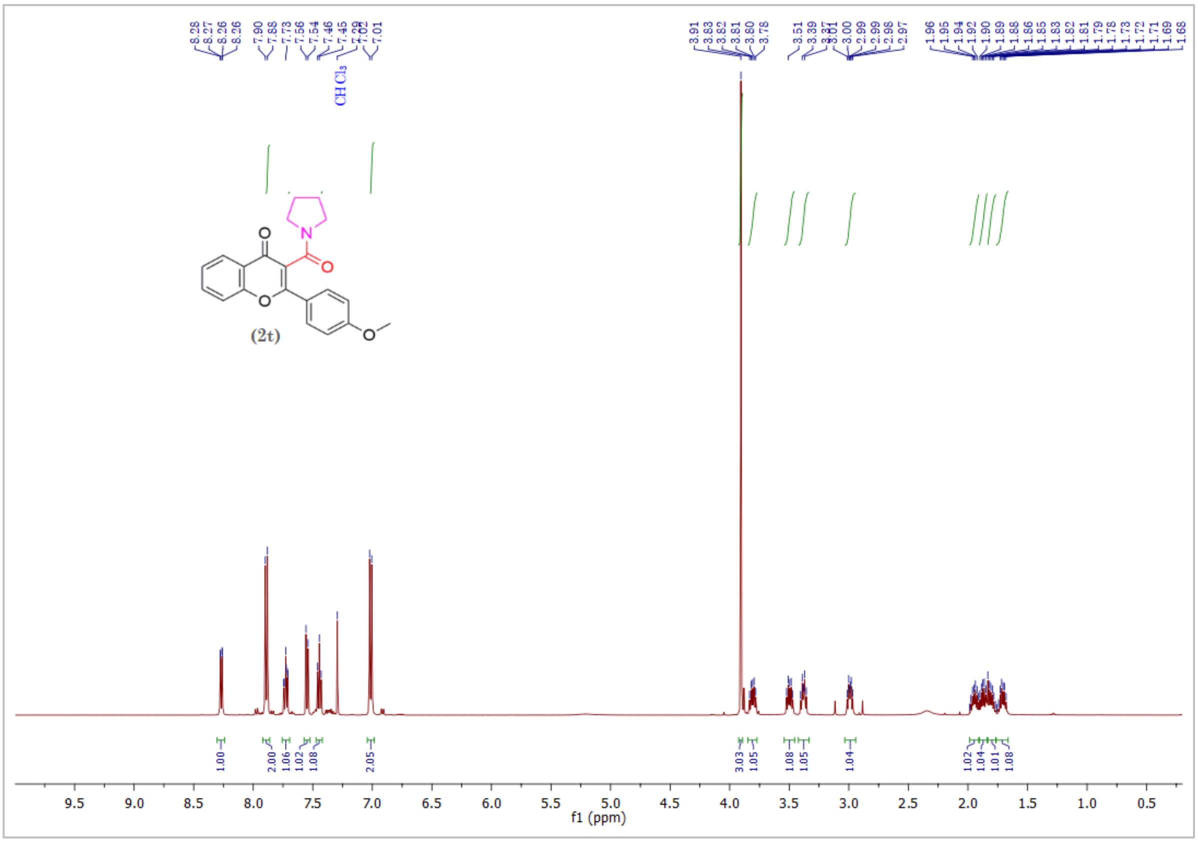

Figure S29. ^{1}H NMR spectrum of compound **2n** (500 MHz, $CDCl_3$)

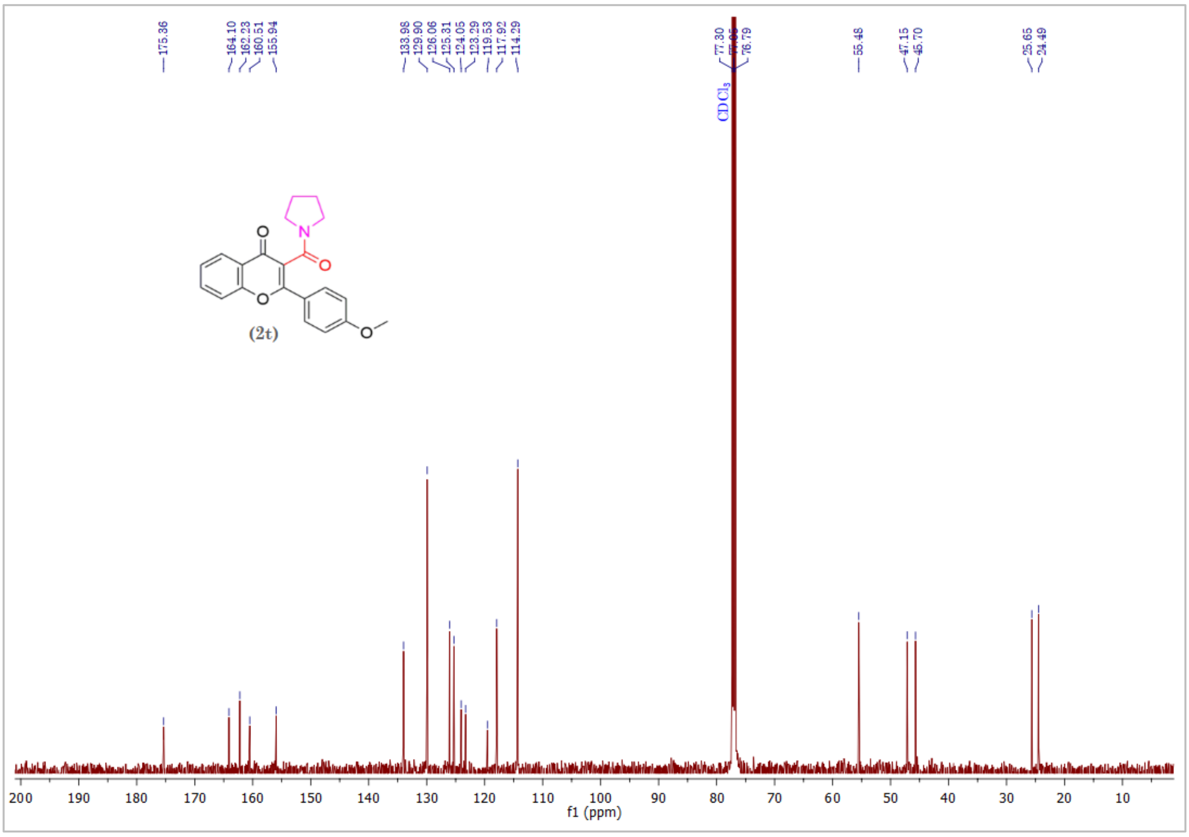

Figure S30. $^{13}C\{^{1}H\}$ NMR spectrum of compound **2n** (125 MHz, $CDCl_3$)

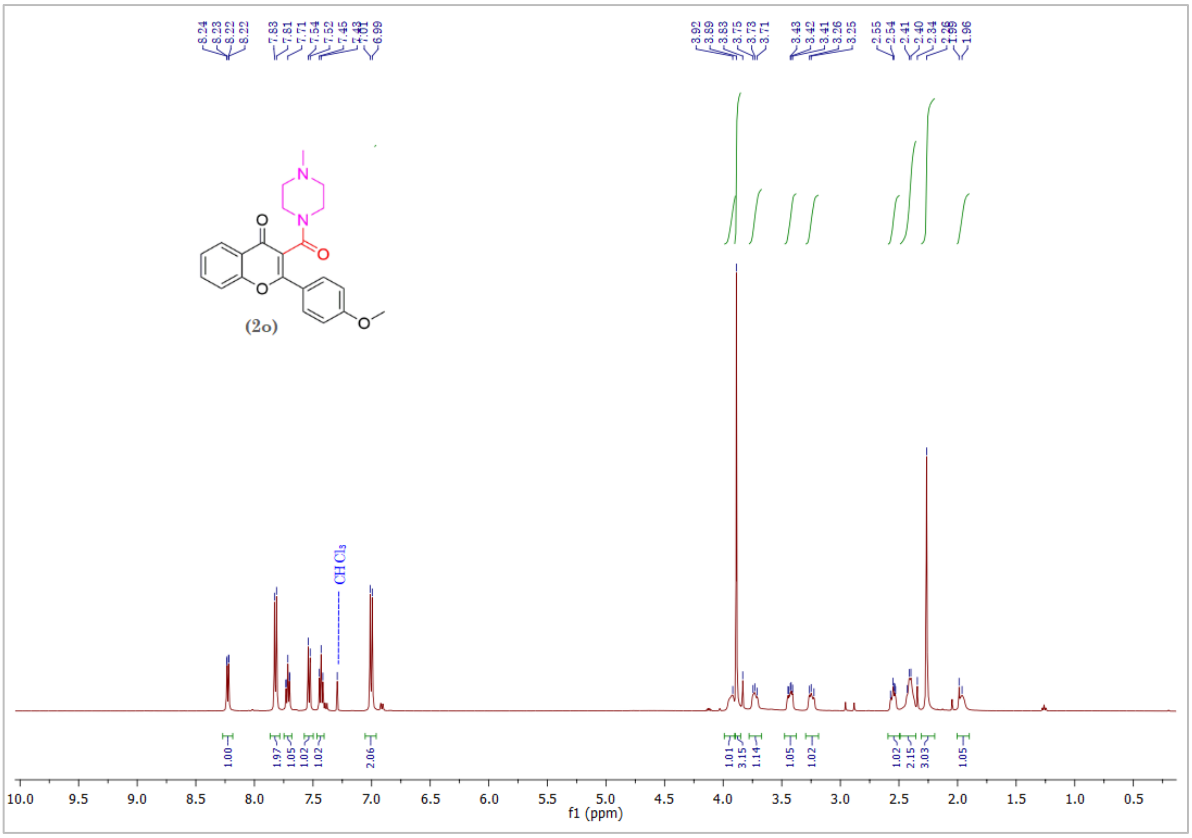

Figure S31. ^{1}H NMR spectrum of compound **2o** (500 MHz, $CDCl_3$)

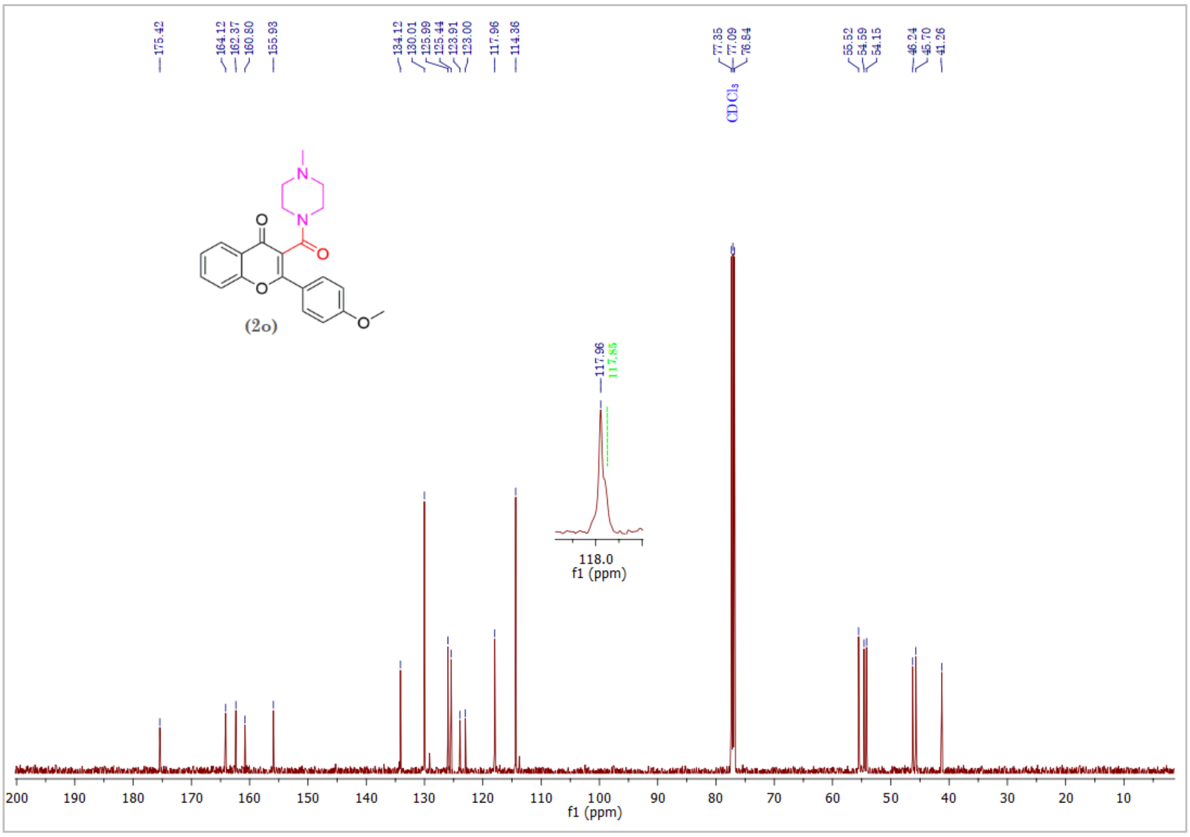

Figure S32. $^{13}C\{^{1}H\}$ NMR spectrum of compound **2o** (125 MHz, $CDCl_3$)

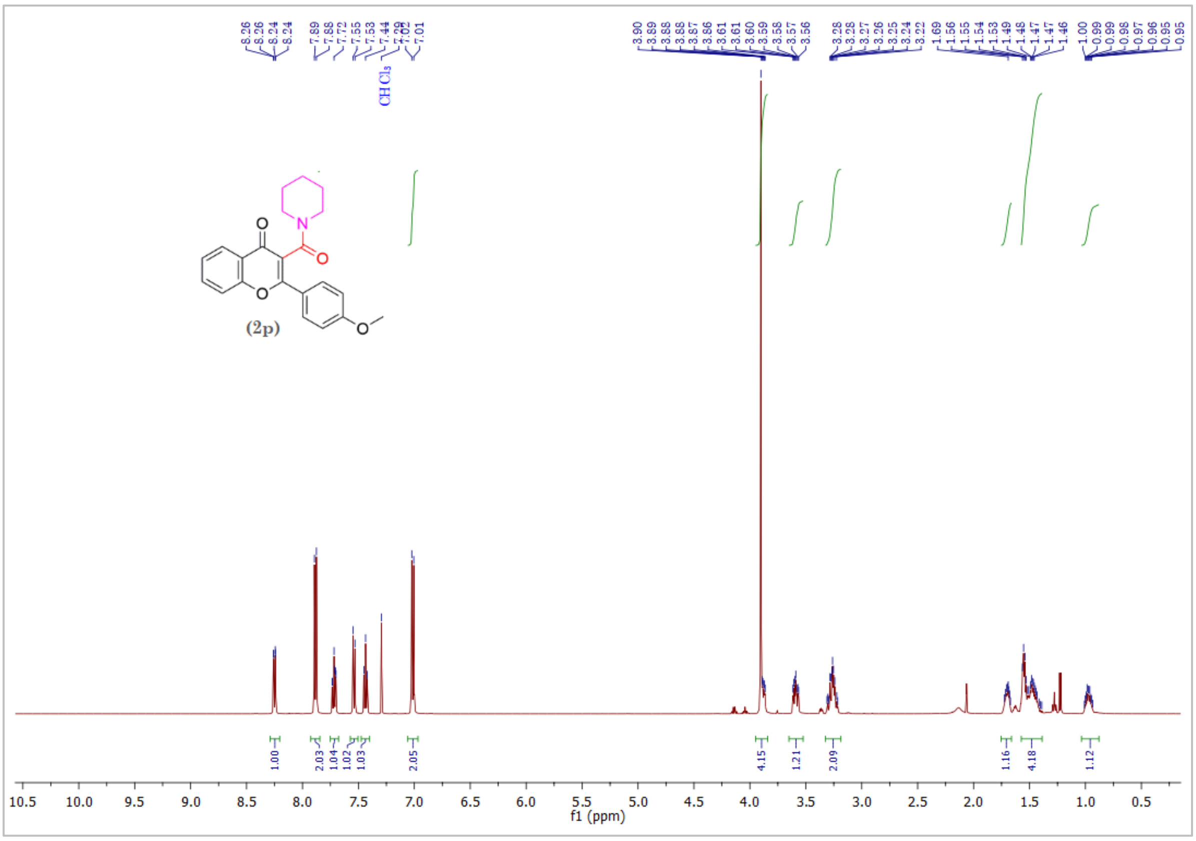

Figure S33. ^{1}H NMR spectrum of compound **2p** (500 MHz, $CDCl_3$)

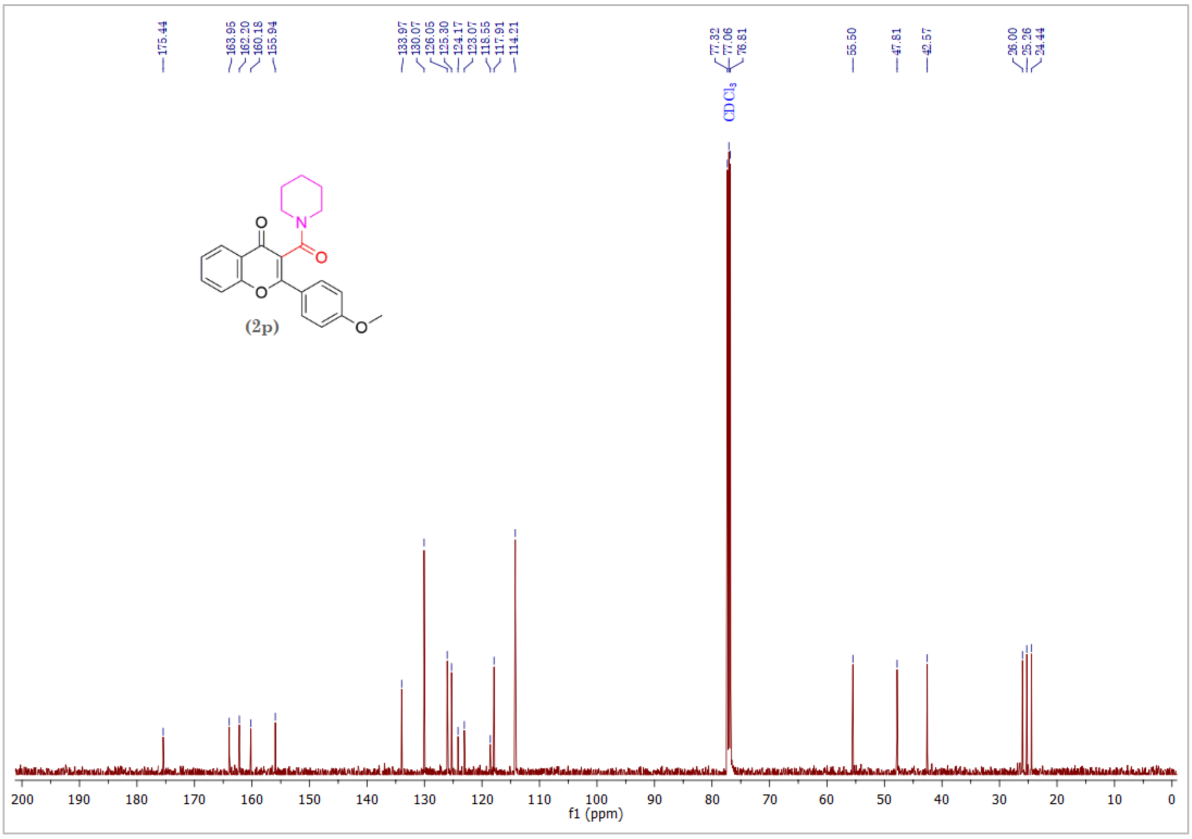

Figure S34. $^{13}C\{^{1}H\}$ NMR spectrum of compound **2p** (125 MHz, $CDCl_3$)

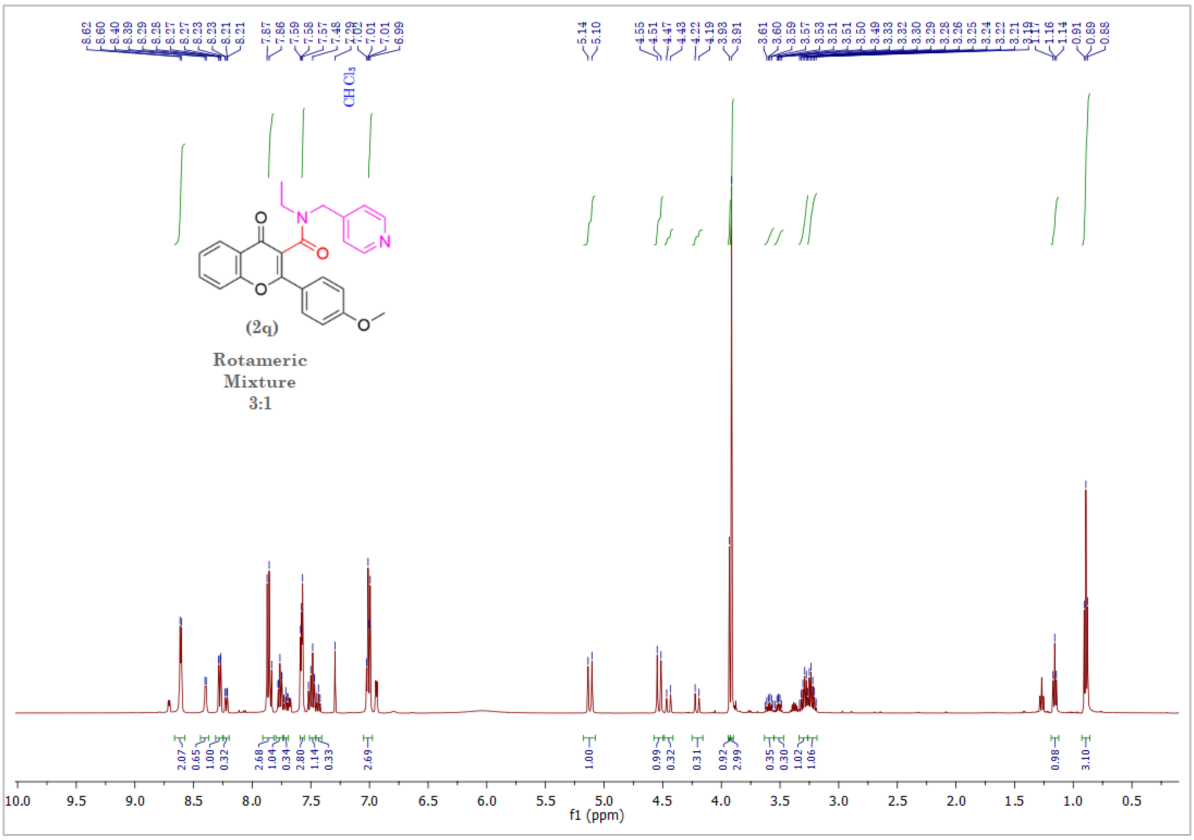

Figure S35. ^{1}H NMR spectrum of compound **2q** (500 MHz, $CDCl_3$)

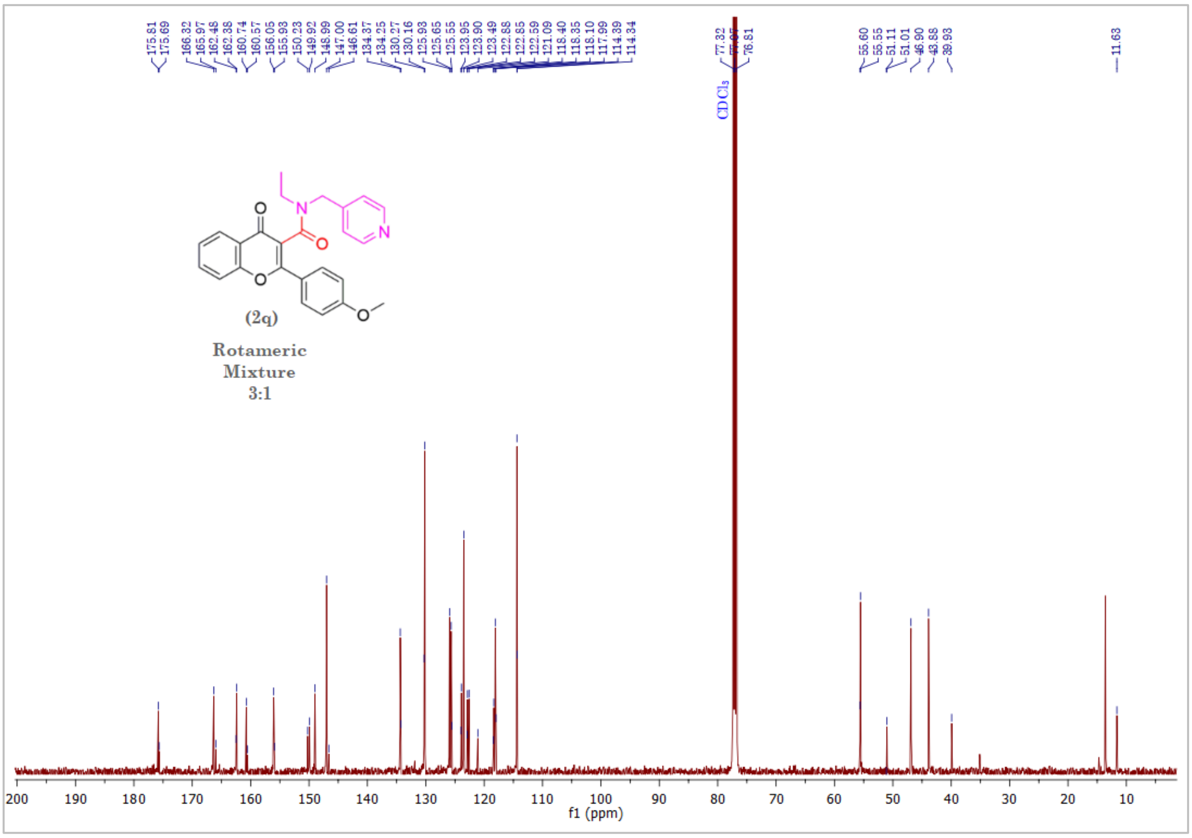

Figure S36. $^{13}C\{^{1}H\}$ NMR spectrum of compound **2q** (125 MHz, $CDCl_3$)

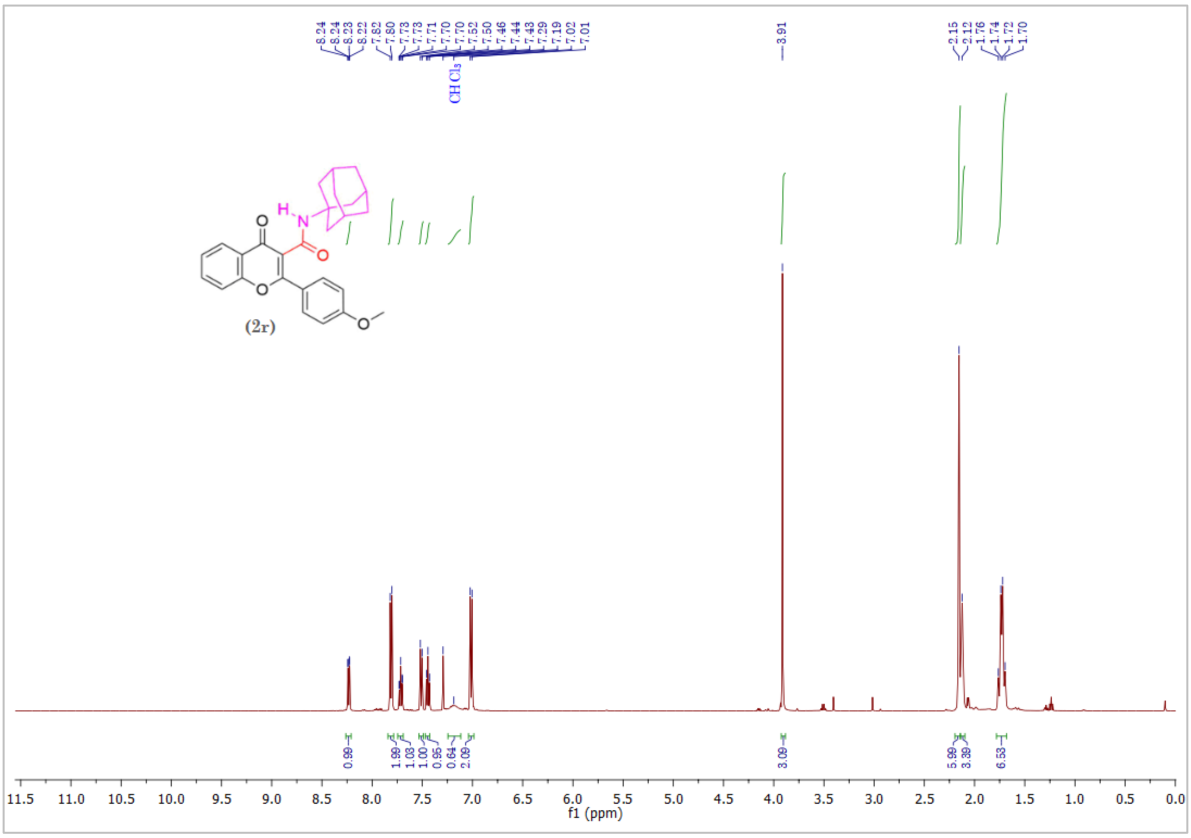

Figure S37. ^{1}H NMR spectrum of compound **2r** (500 MHz, $CDCl_3$)

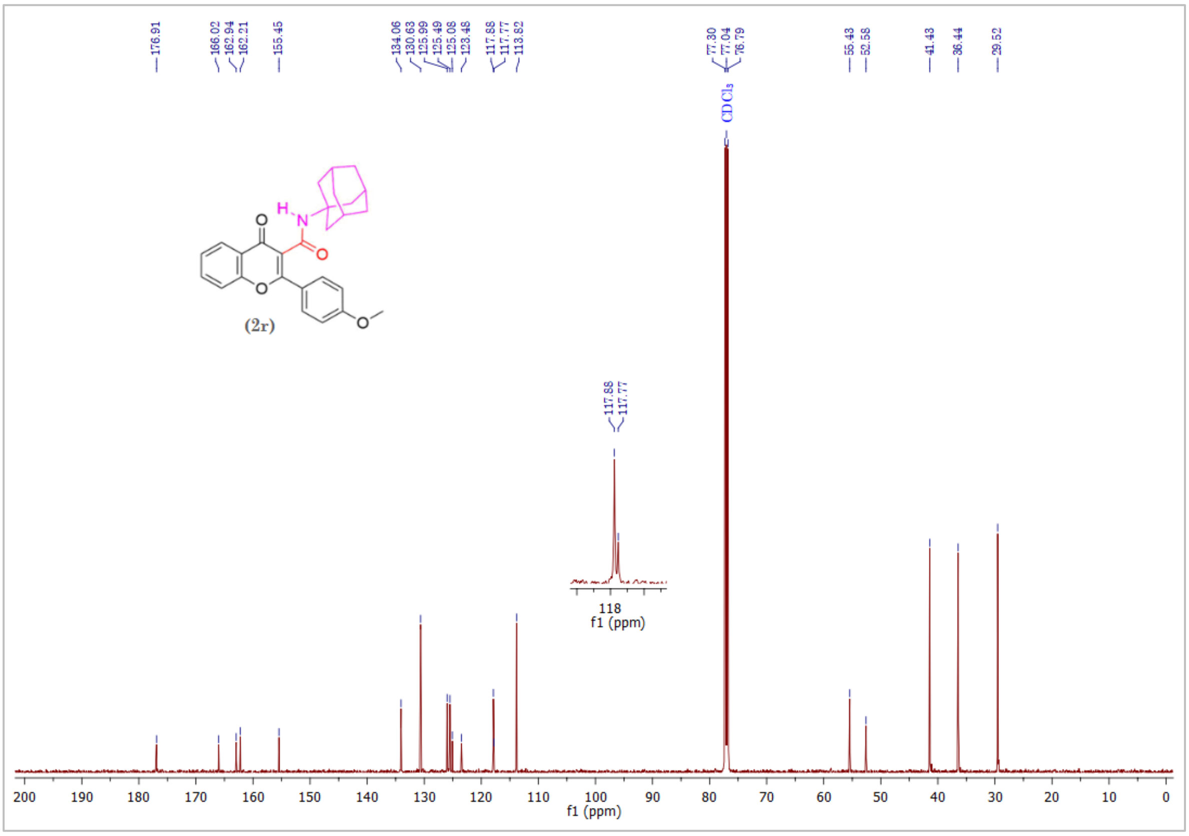

Figure S38. $^{13}C\{^{1}H\}$ NMR spectrum of compound **2r** (125 MHz, $CDCl_3$)

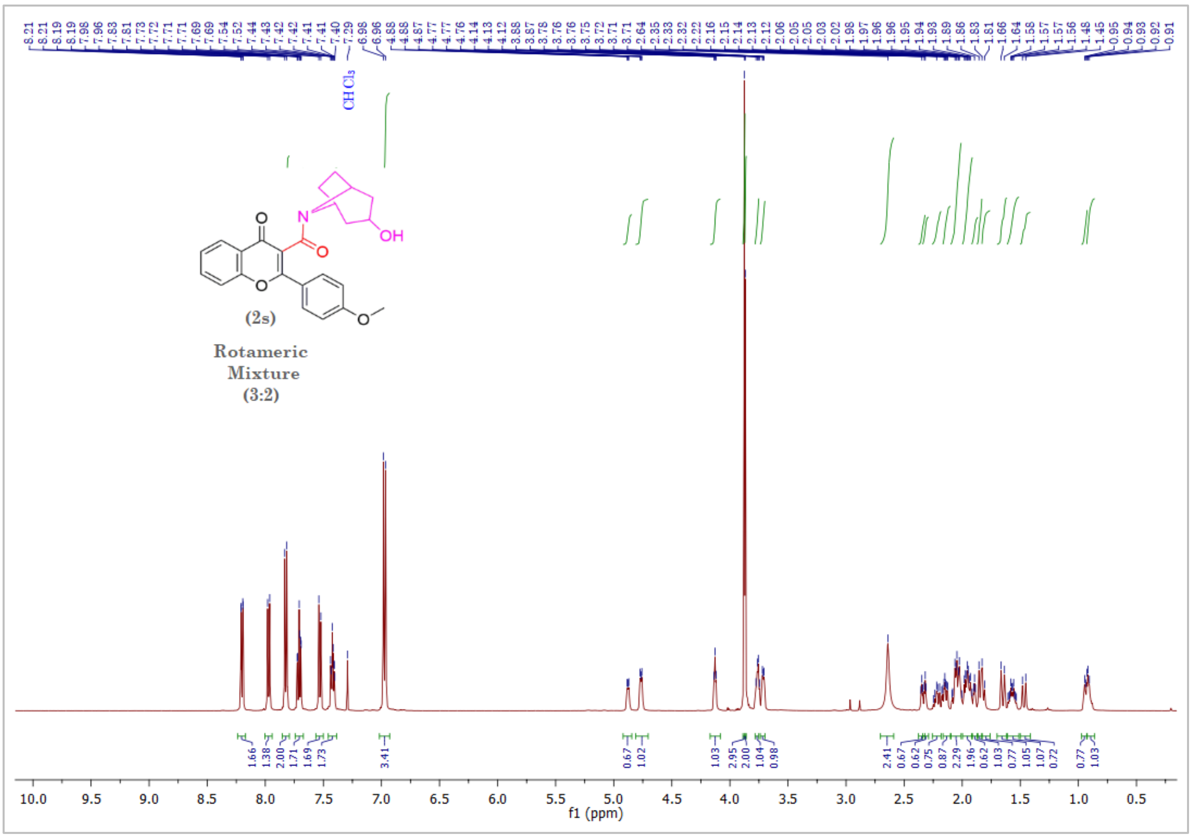

Figure S39. ^{1}H NMR spectrum of compound **2s** (500 MHz, $CDCl_3$)

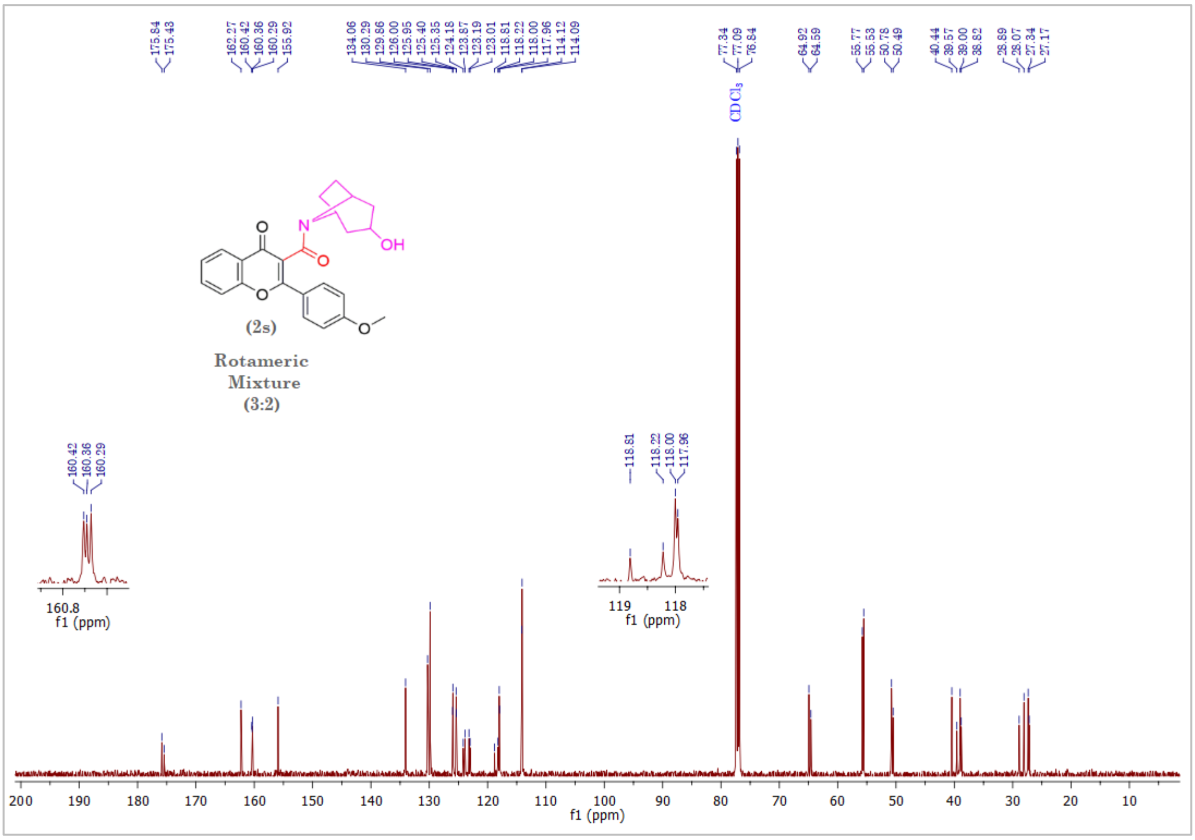

Figure S40. $^{13}C\{^{1}H\}$ NMR spectrum of compound **2s** (125 MHz, $CDCl_3$)

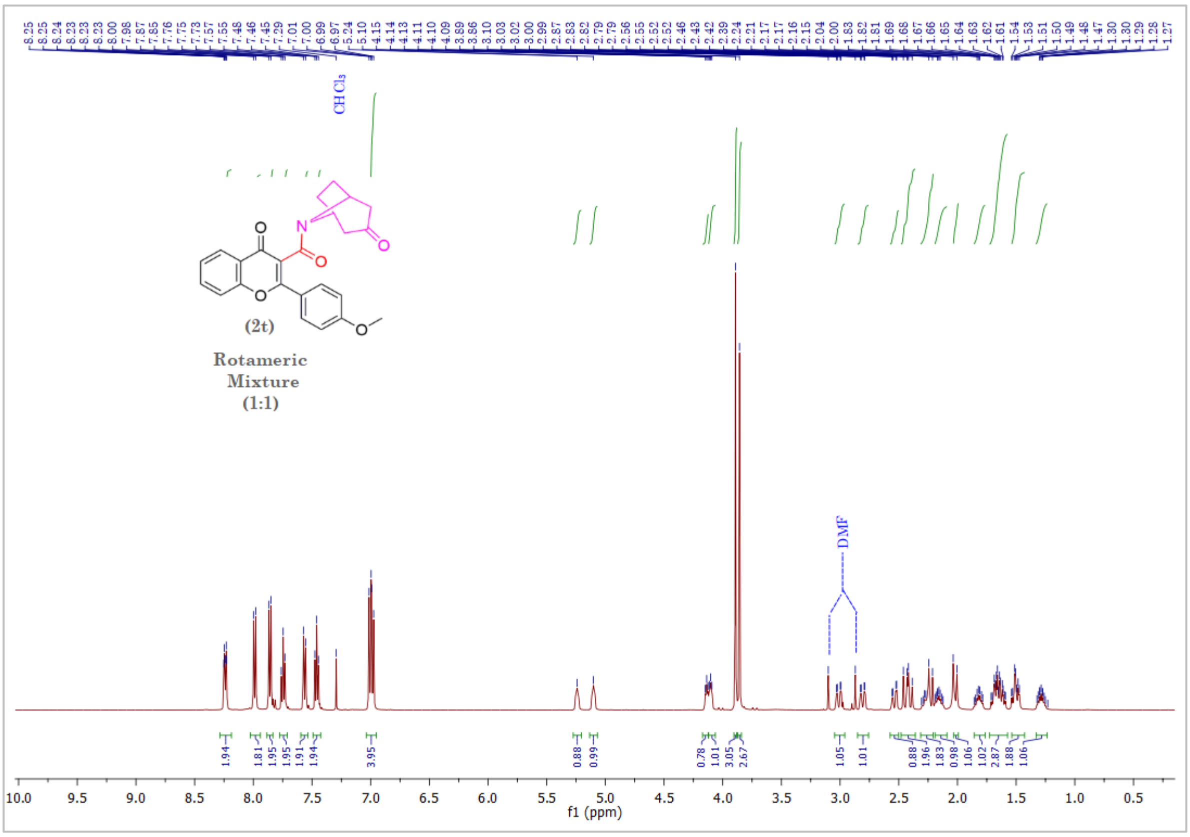

Figure S41. ^{1}H NMR spectrum of compound **2t** (500 MHz, $CDCl_3$)

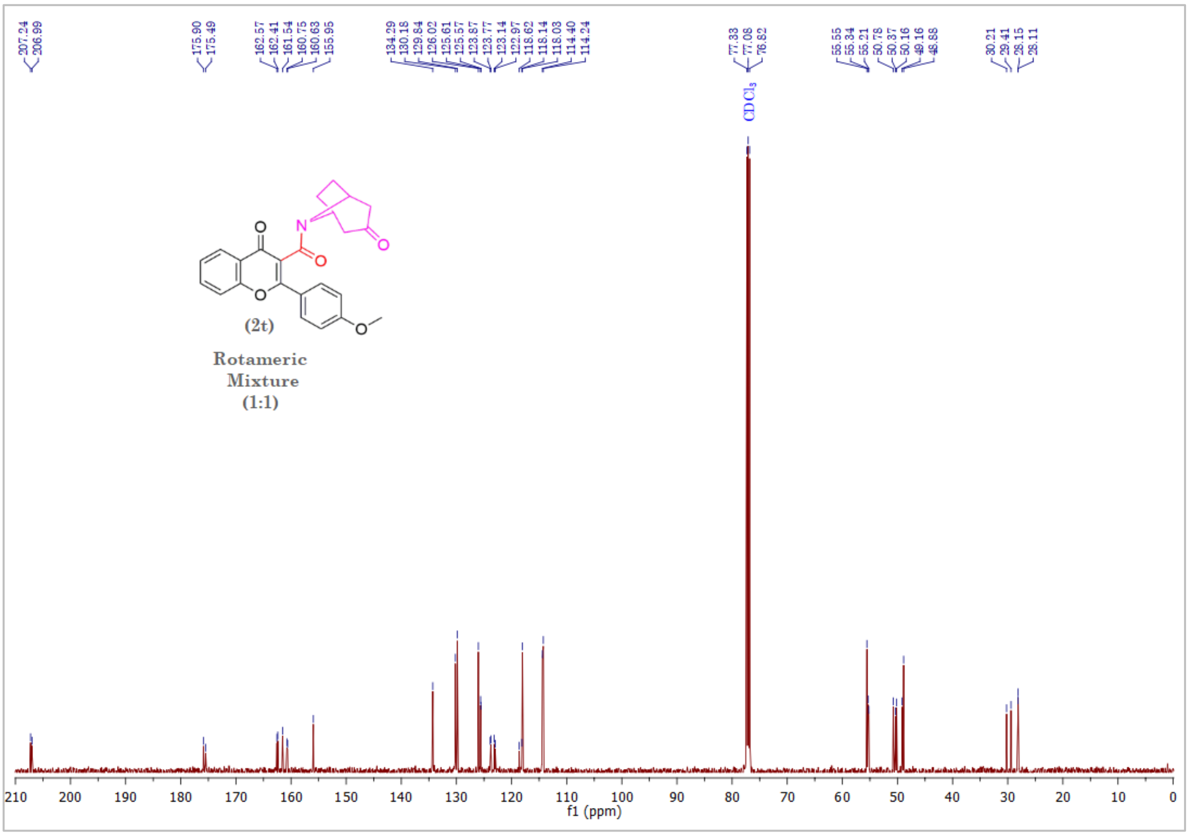

Figure S42. $^{13}C\{^{1}H\}$ NMR spectrum of compound **2t** (125 MHz, $CDCl_3$)

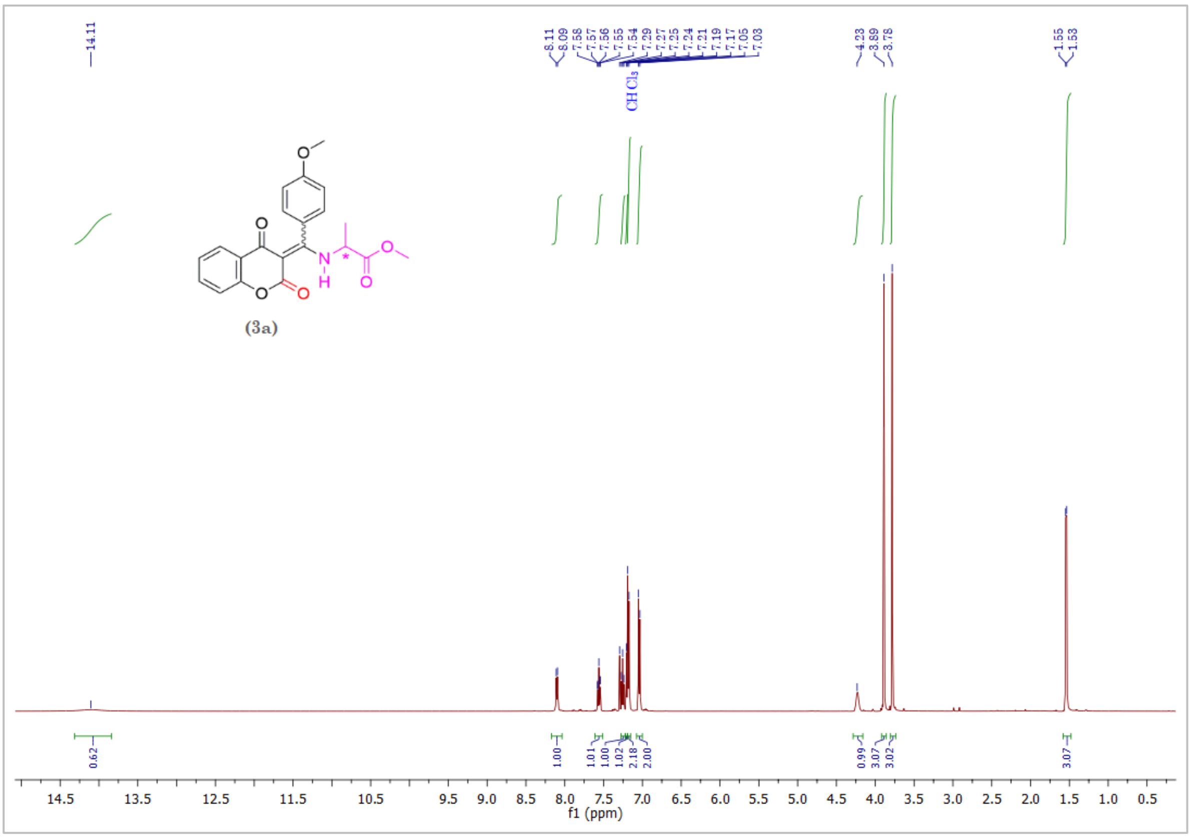

Figure S43. ^{1}H NMR spectrum of compound **3a** (500 MHz, $CDCl_3$)

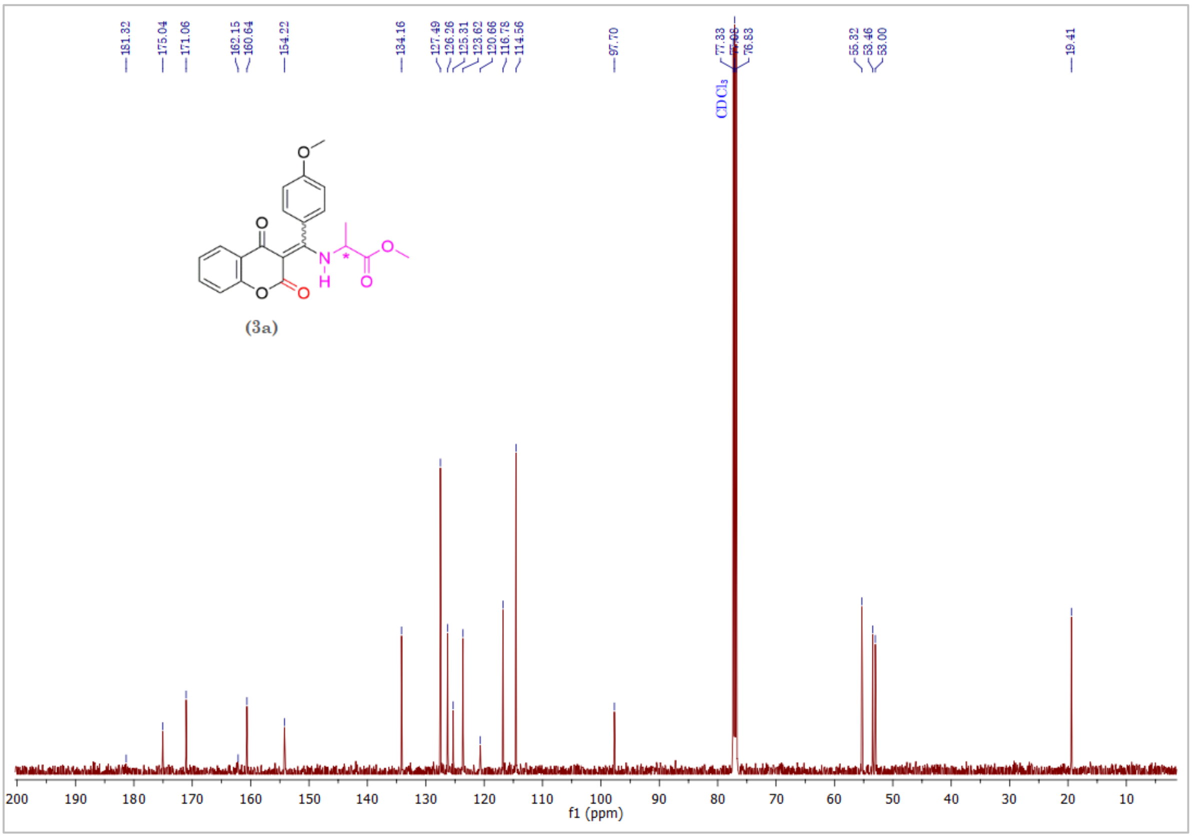

Figure S44. $^{13}C\{^{1}H\}$ NMR spectrum of compound **3a** (125 MHz, $CDCl_3$)

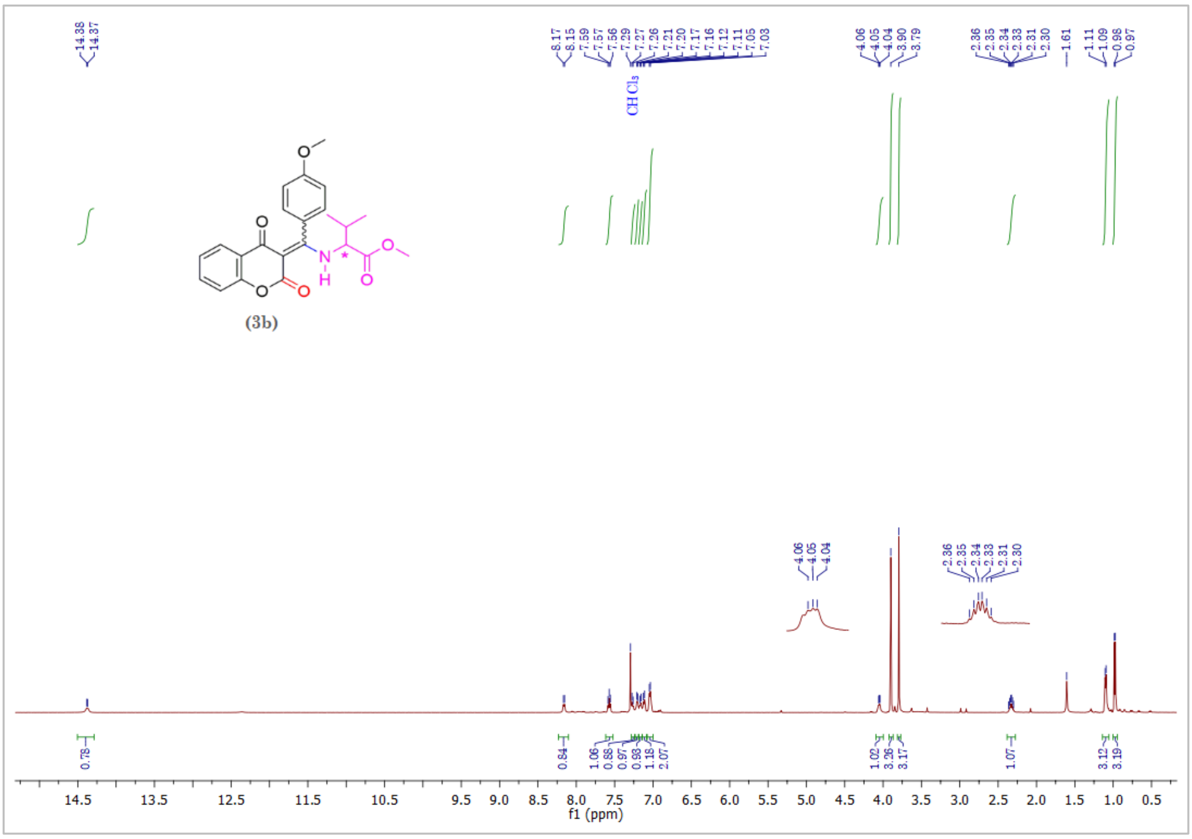

Figure S45. ^{1}H NMR spectrum of compound **3b** (500 MHz, $CDCl_3$)

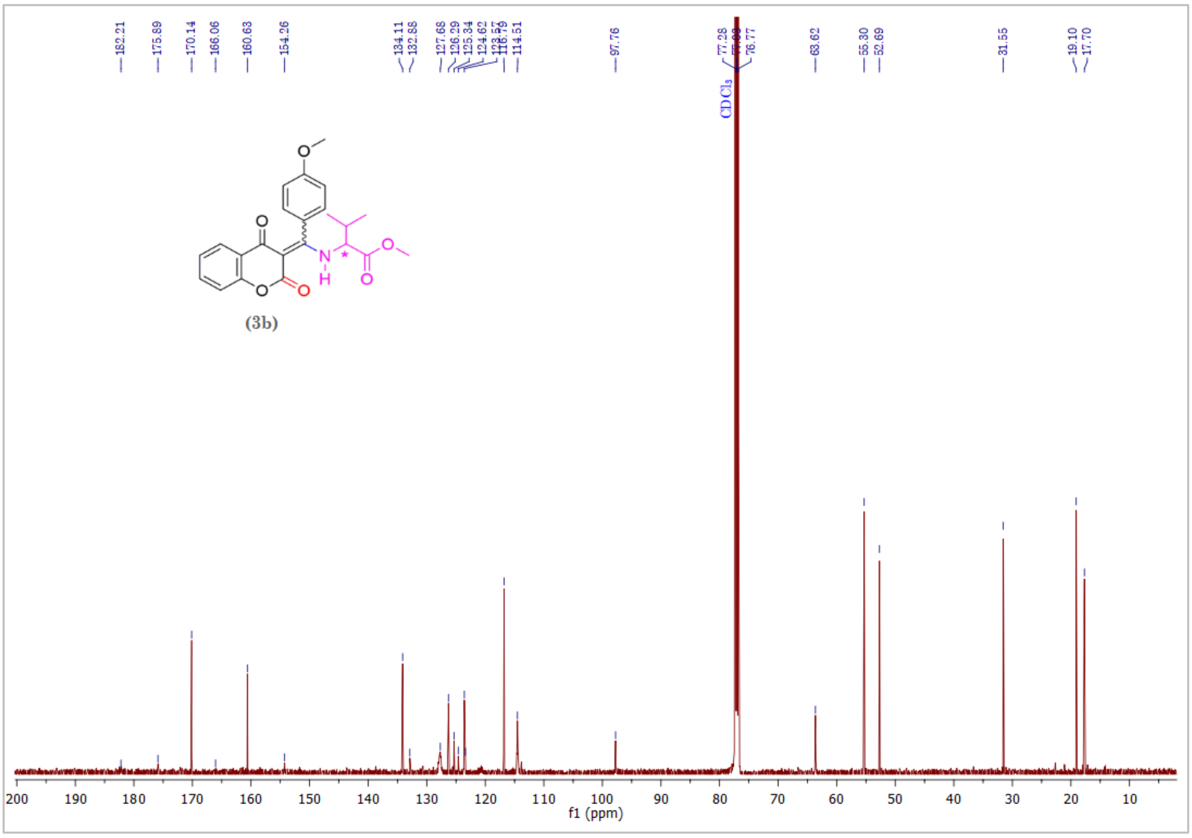

Figure S46. $^{13}C\{^{1}H\}$ NMR spectrum of compound **3b** (125 MHz, $CDCl_3$)

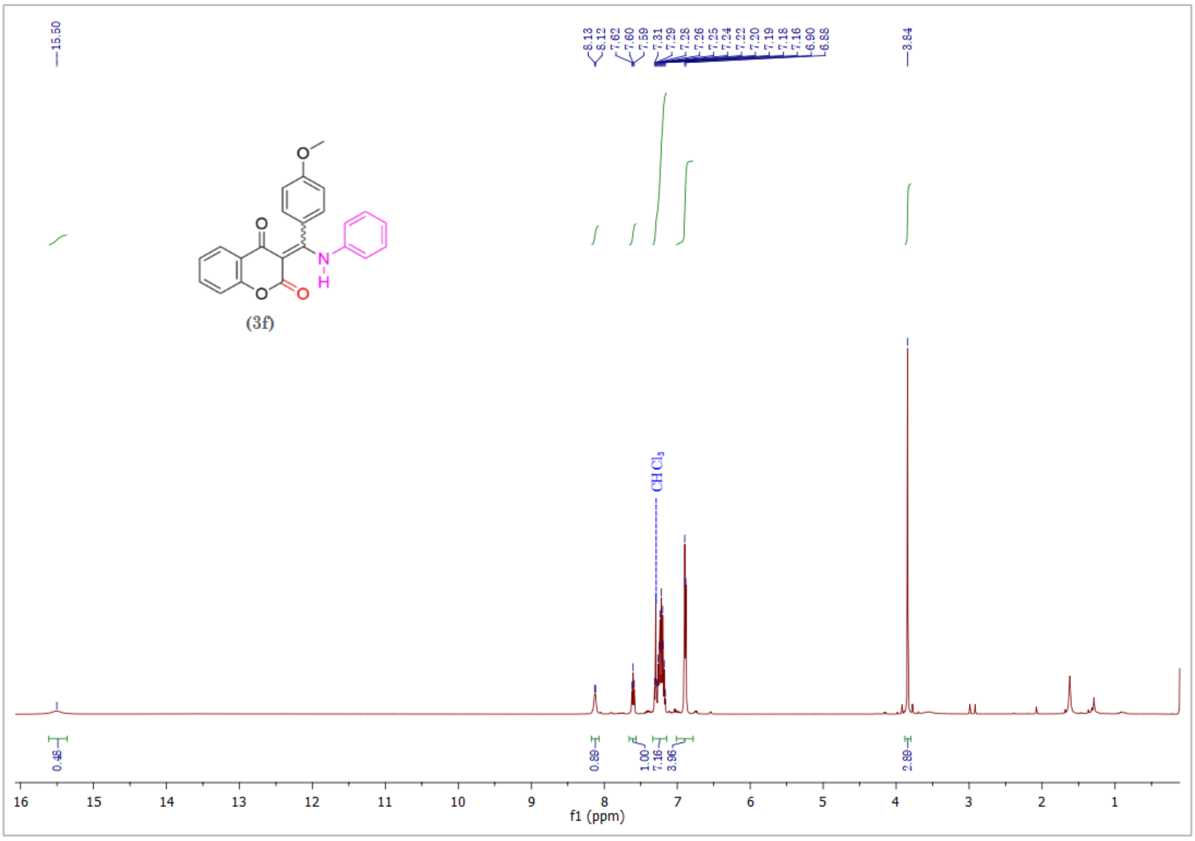

Figure S47. ^{1}H NMR spectrum of compound **3f** (500 MHz, $CDCl_3$)

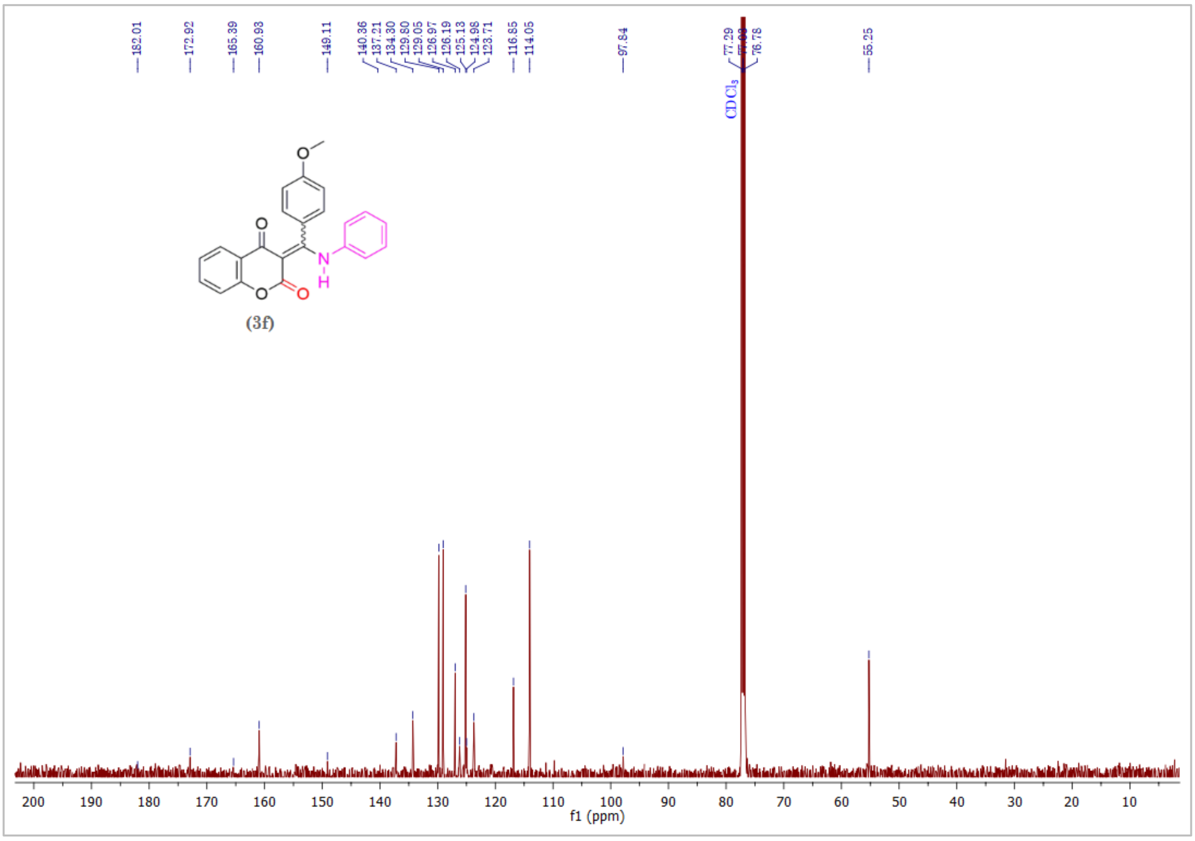

Figure S48. $^{13}C\{^{1}H\}$ NMR spectrum of compound **3f** (125 MHz, $CDCl_3$)

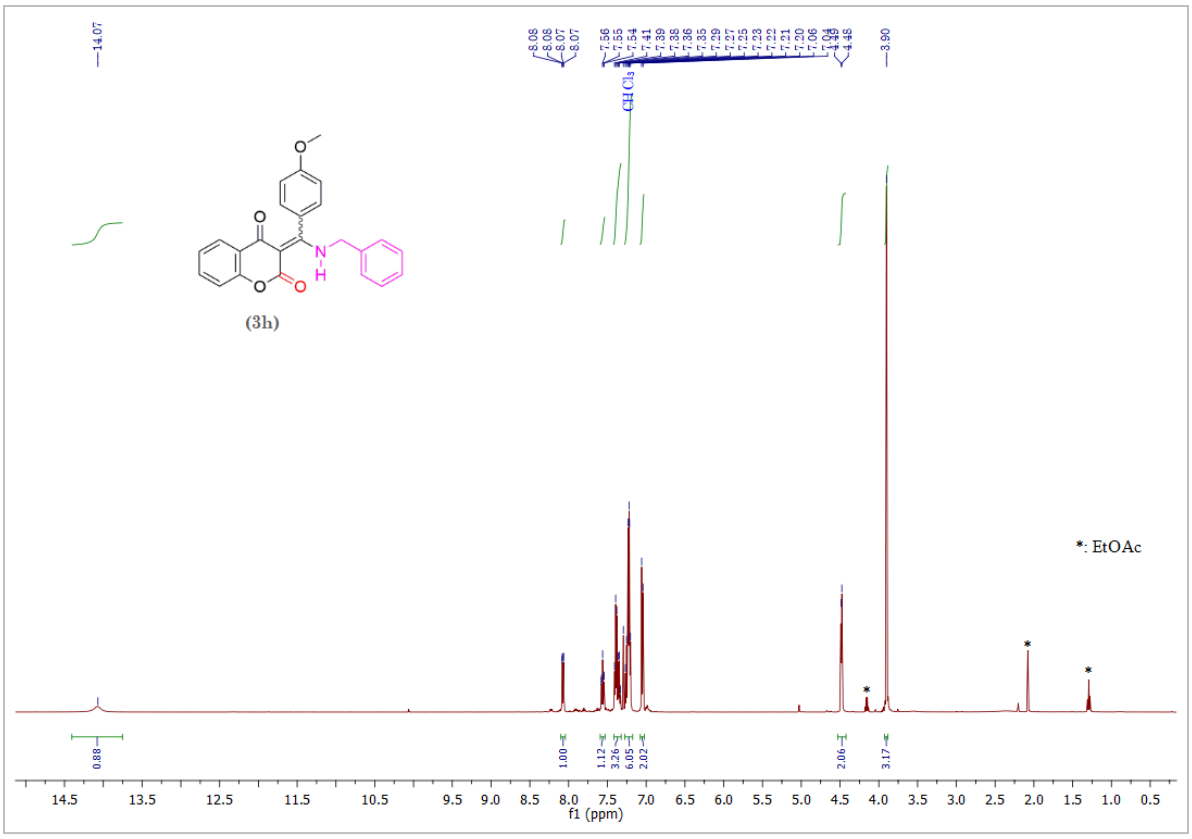

Figure S49. ^{1}H NMR spectrum of compound **3h** (500 MHz, $CDCl_3$)

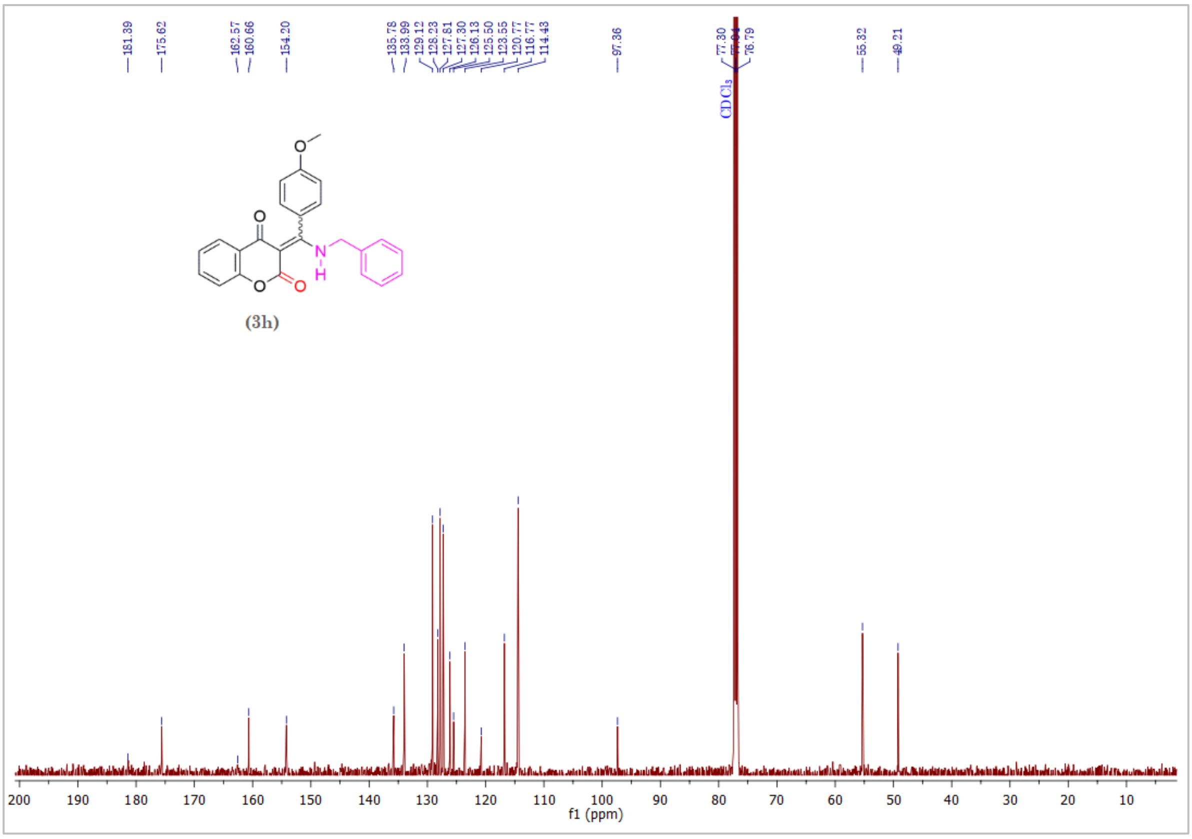

Figure S50. $^{13}C\{^{1}H\}$ NMR spectrum of compound **3h** (125 MHz, $CDCl_3$)

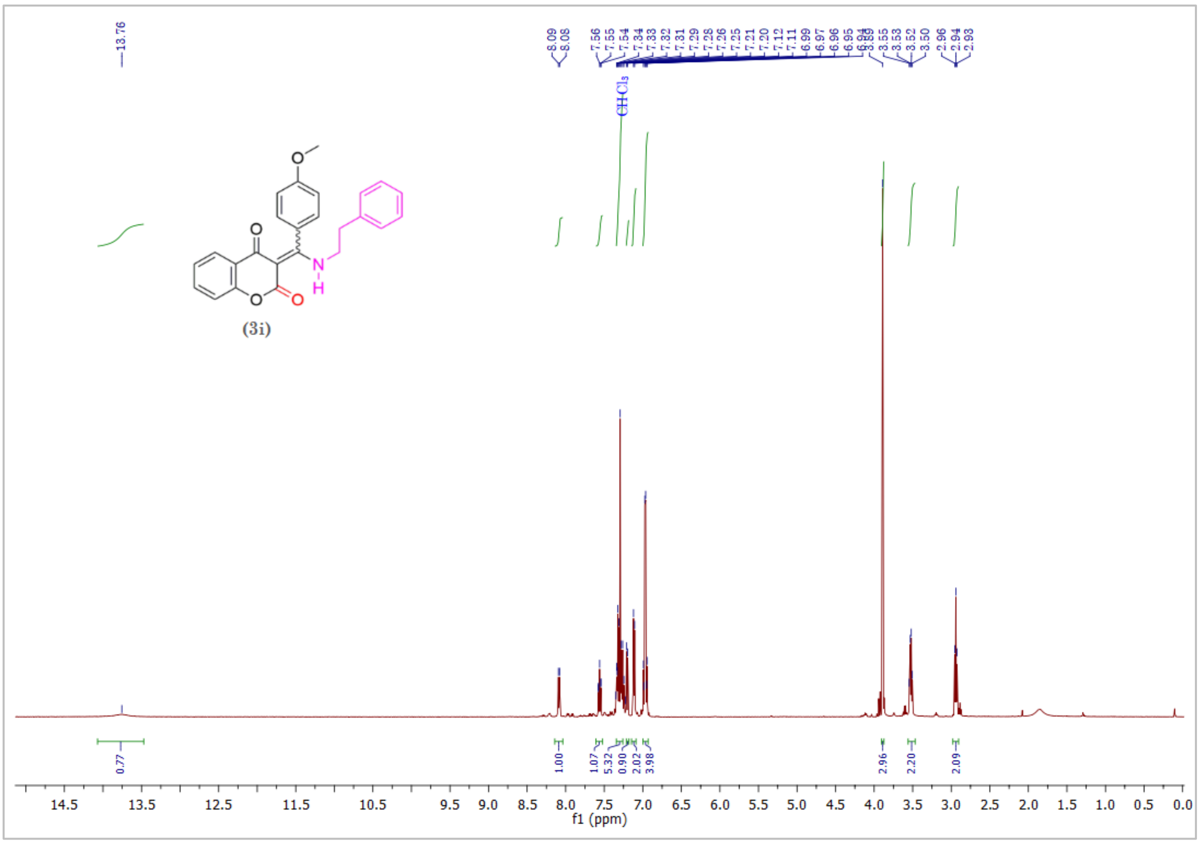

Figure S51. ^{1}H NMR spectrum of compound **3i** (500 MHz, $CDCl_3$)

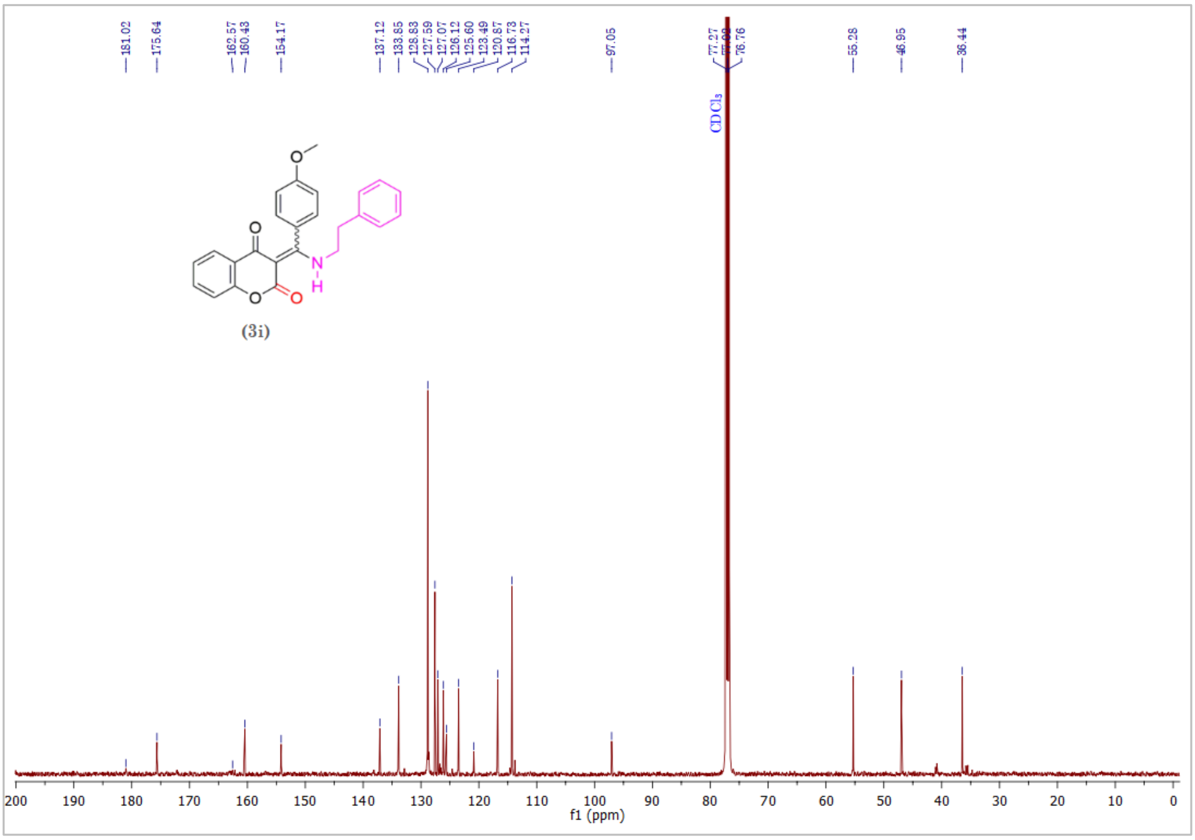

Figure S52. $^{13}C\{^{1}H\}$ NMR spectrum of compound **3i** (125 MHz, $CDCl_3$)

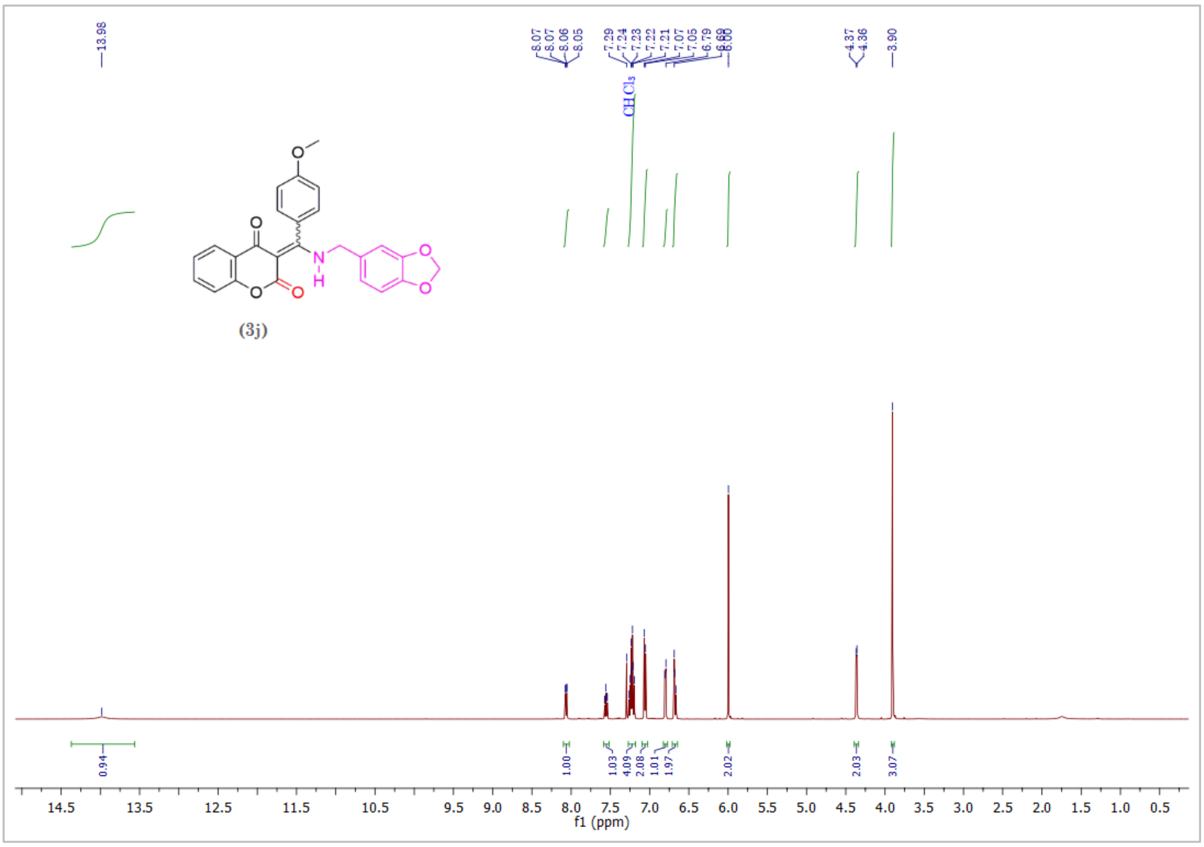

Figure S53. ^{1}H NMR spectrum of compound **3j** (500 MHz, $CDCl_3$)

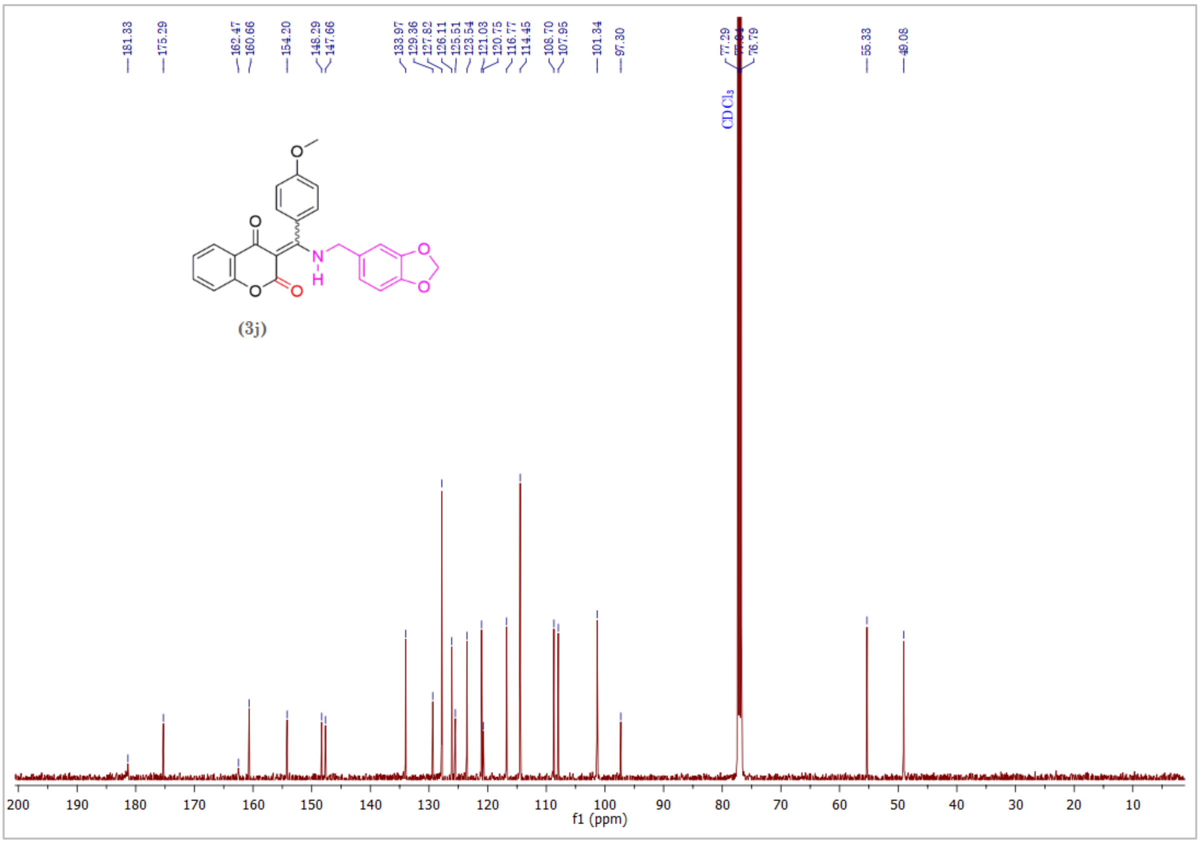

Figure S54. $^{13}C\{^{1}H\}$ NMR spectrum of compound **3j** (125 MHz, $CDCl_3$)

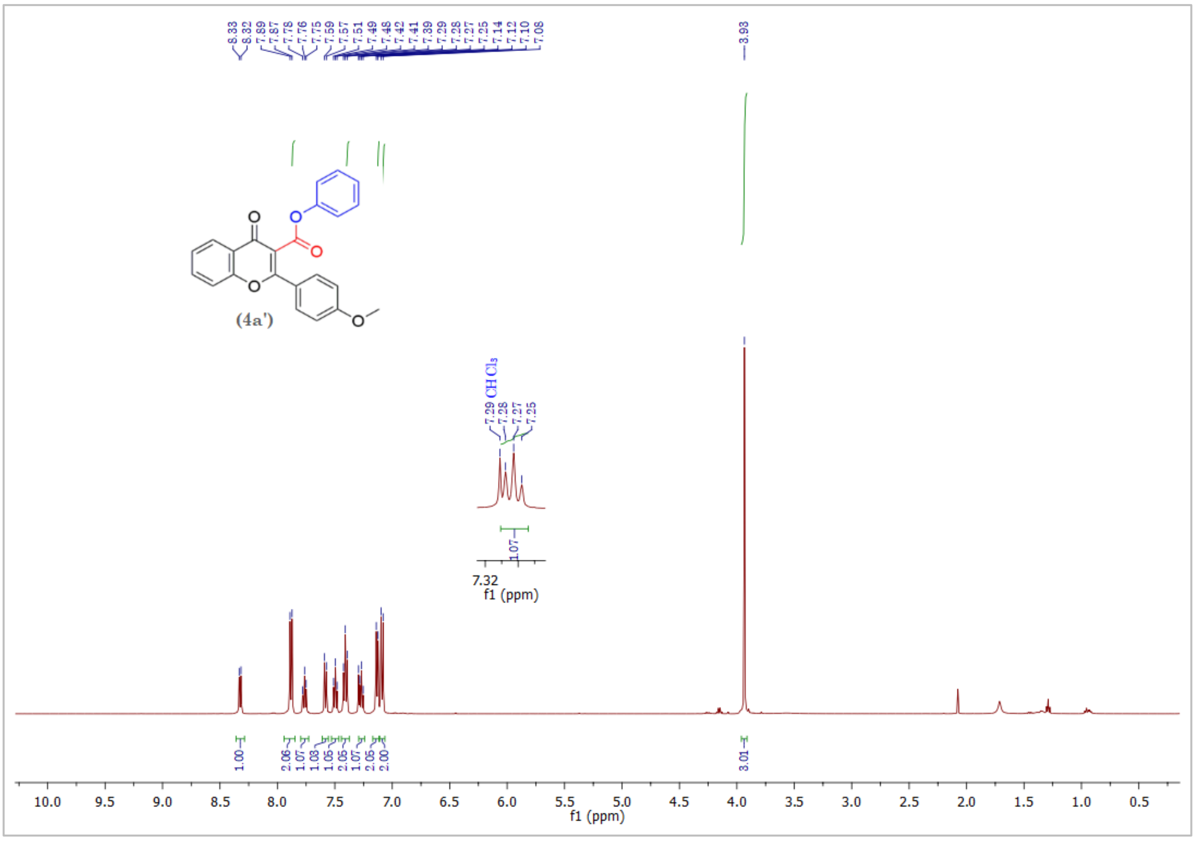

Figure S55. ^{1}H NMR spectrum of compound **4a'** (500 MHz, $CDCl_3$)

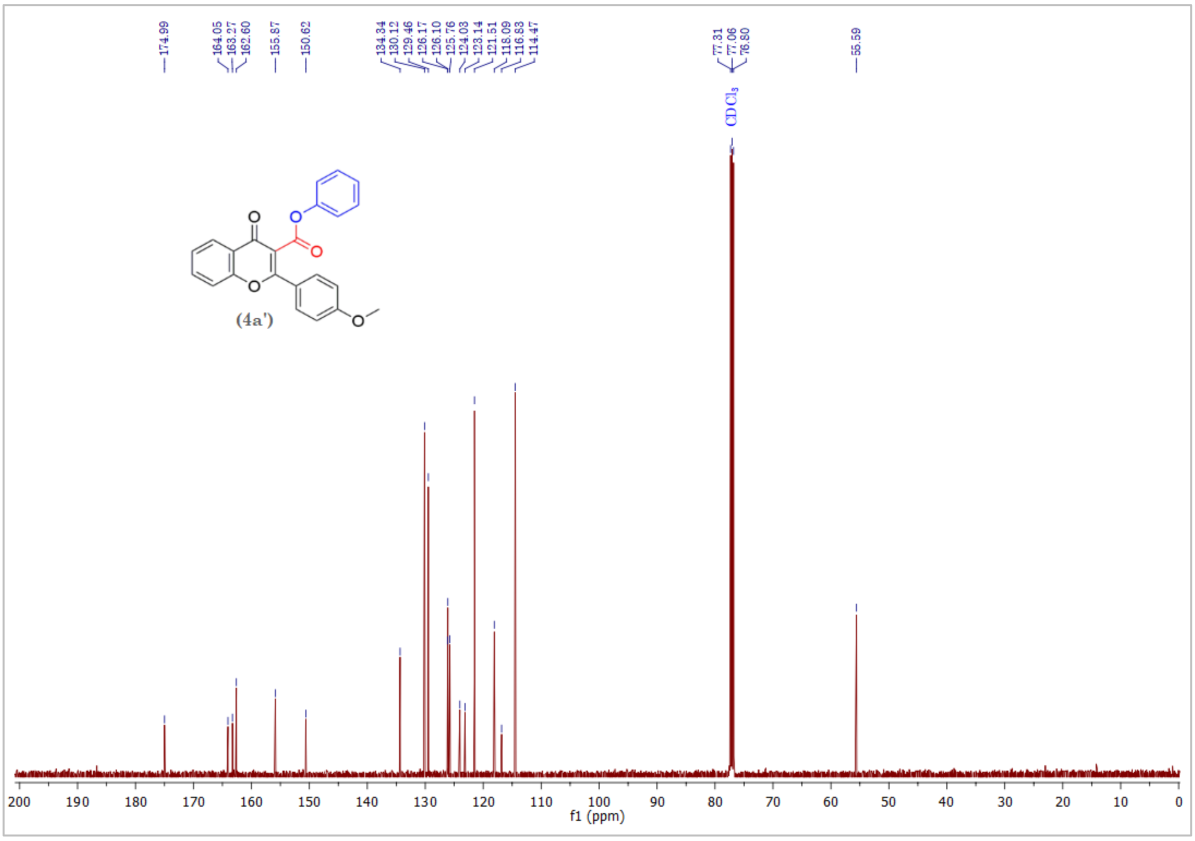

Figure S56. $^{13}C\{^{1}H\}$ NMR spectrum of compound **4a'** (125 MHz, $CDCl_3$)

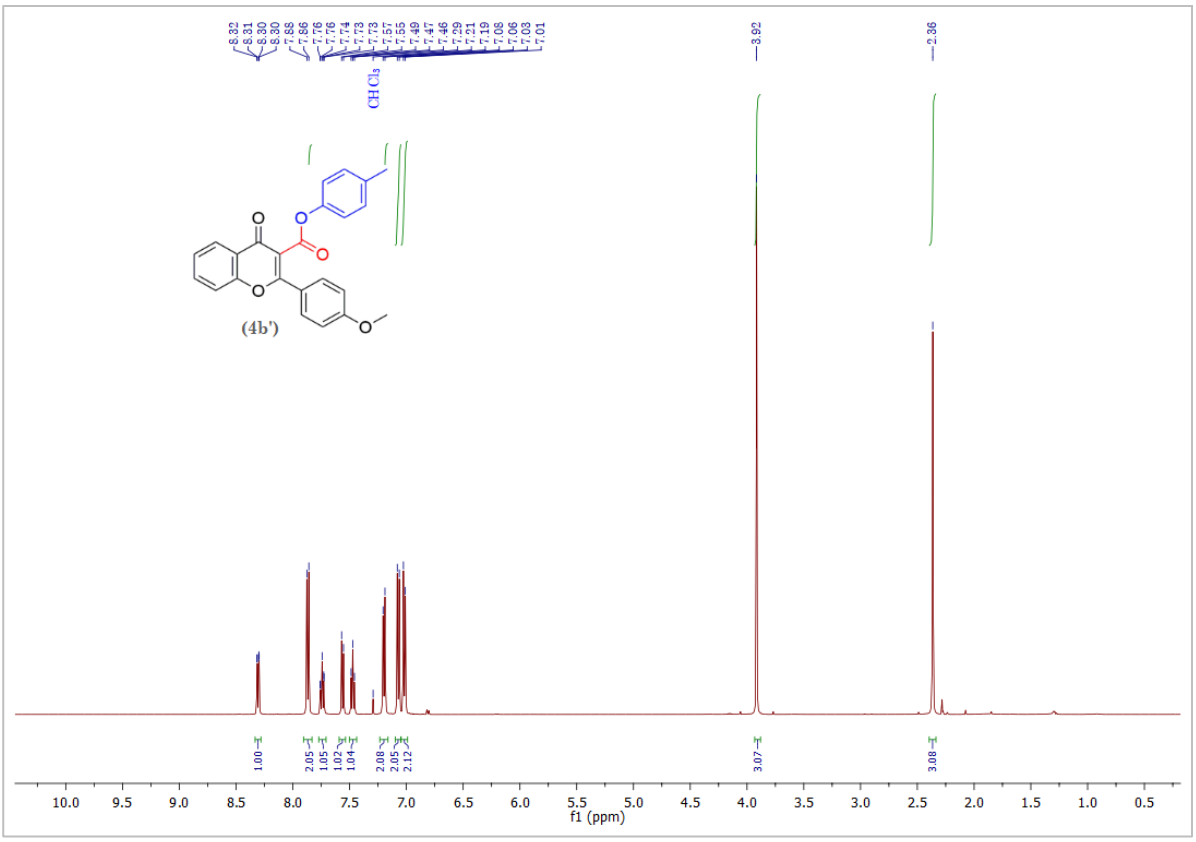

Figure S57. ^{1}H NMR spectrum of compound **4b'** (500 MHz, $CDCl_3$)

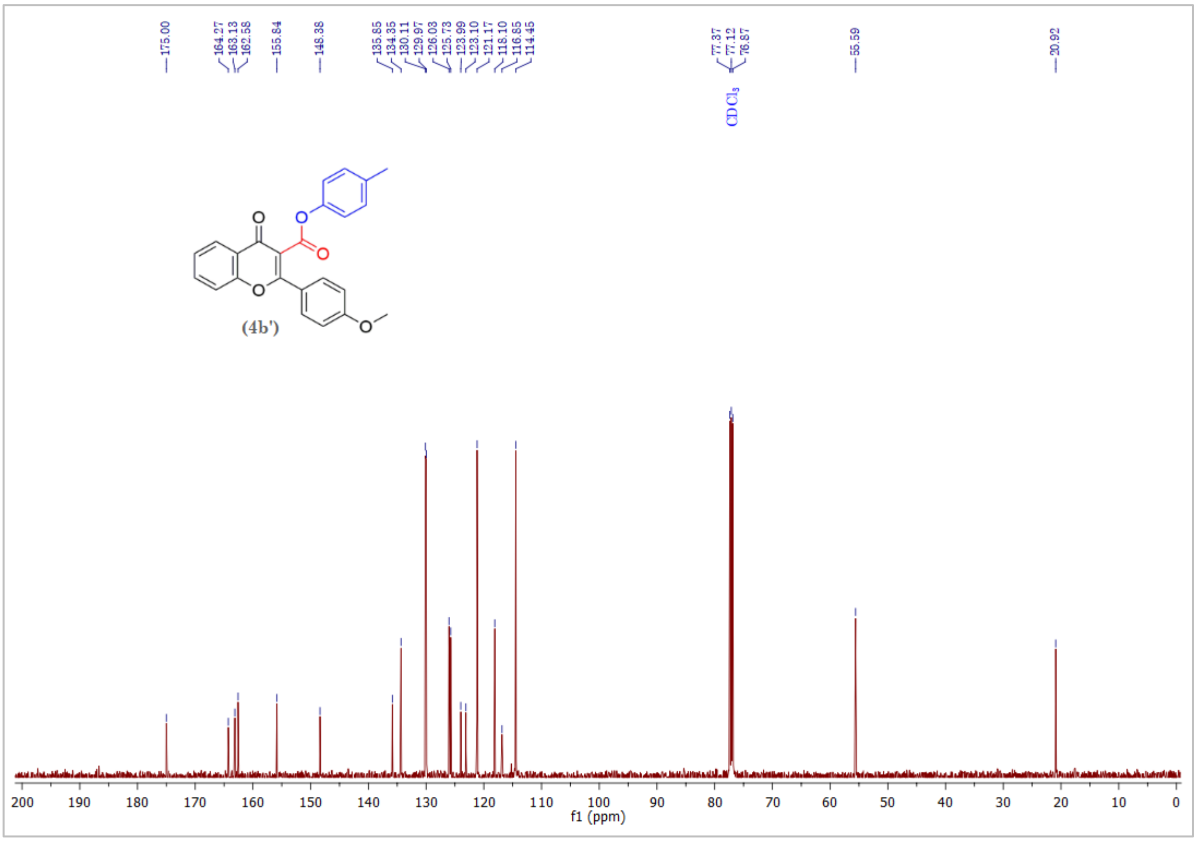

Figure S58. $^{13}C\{^{1}H\}$ NMR spectrum of compound **4b'** (125 MHz, $CDCl_3$)

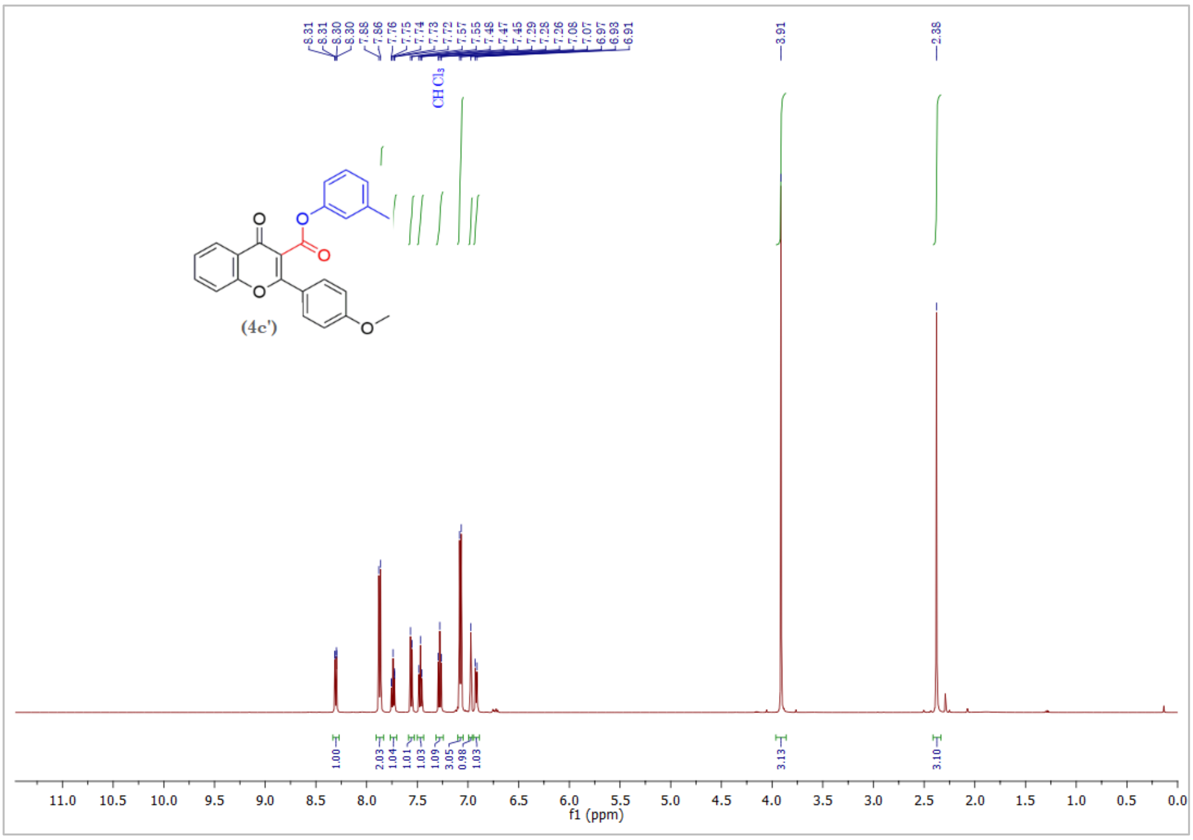

Figure S59. ^{1}H NMR spectrum of compound **4c'** (500 MHz, $CDCl_3$)

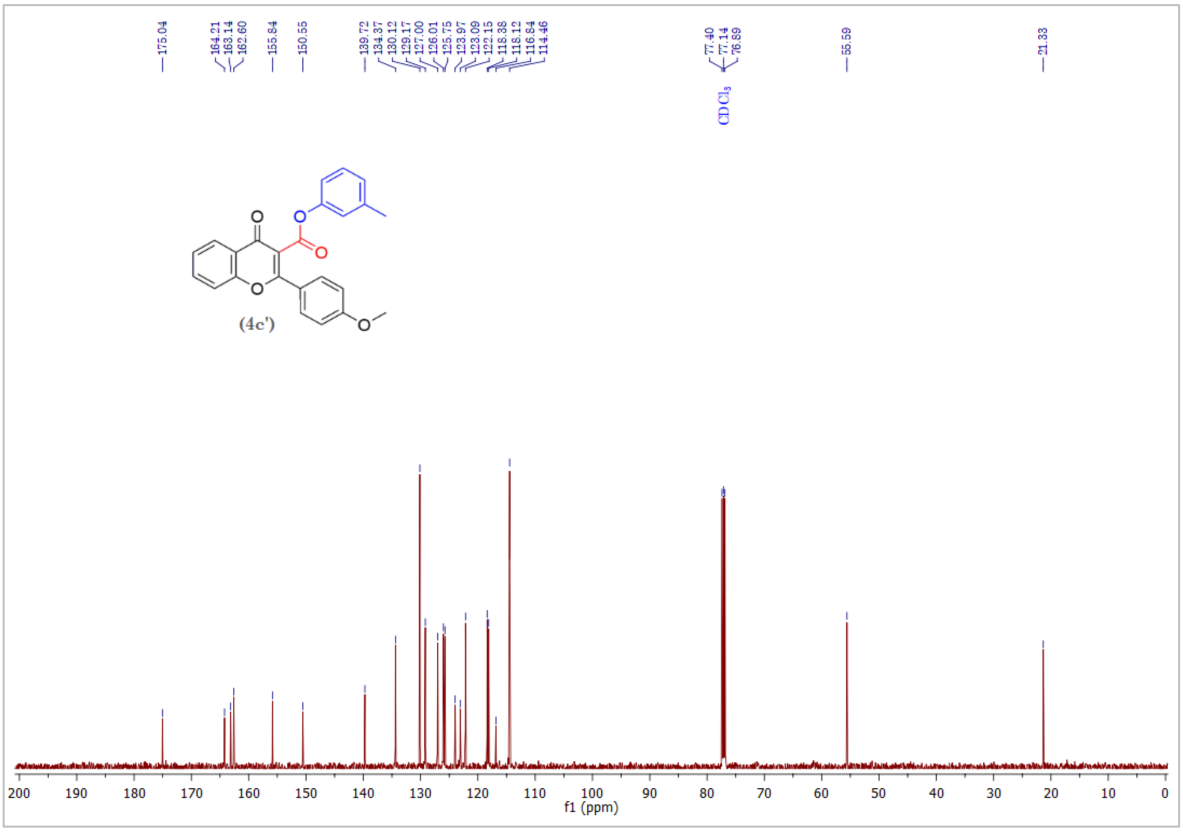

Figure S60. $^{13}C\{^{1}H\}$ NMR spectrum of compound **4c'** (125 MHz, $CDCl_3$)

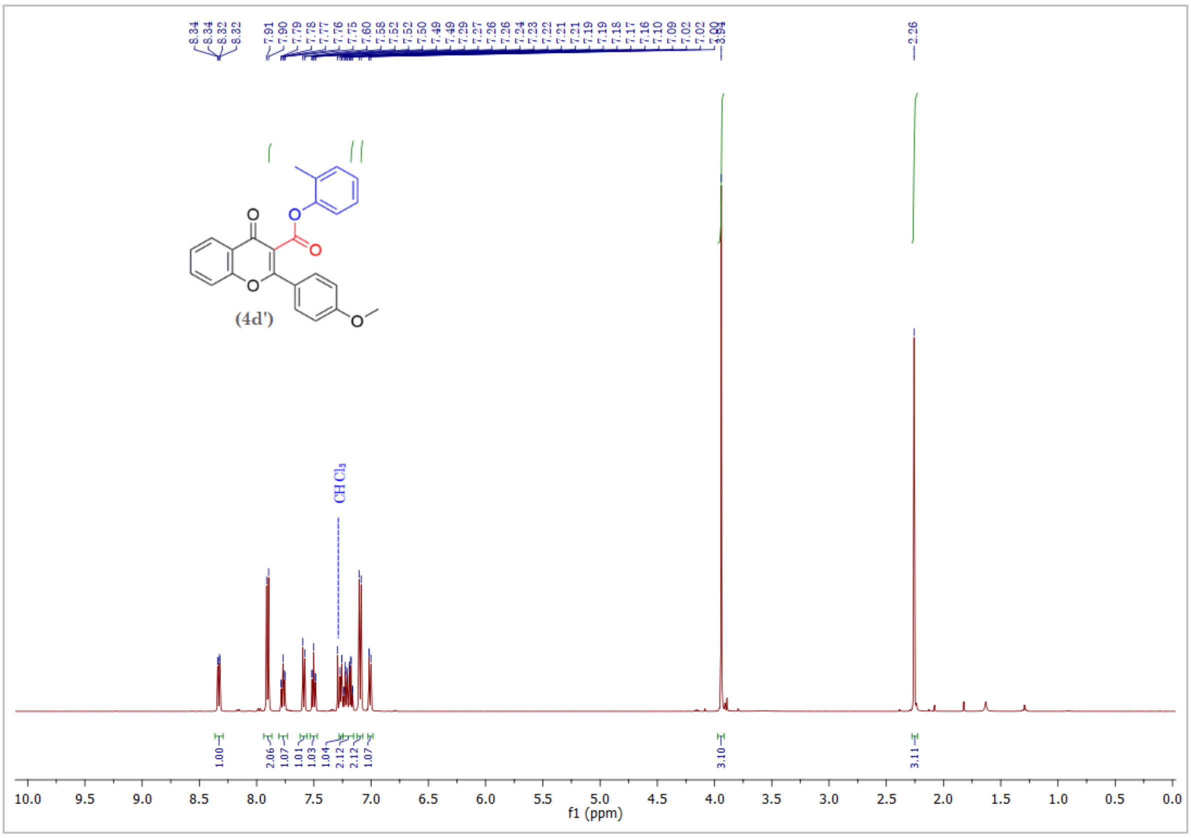

Figure S61. ^{1}H NMR spectrum of compound **4d'** (500 MHz, $CDCl_3$)

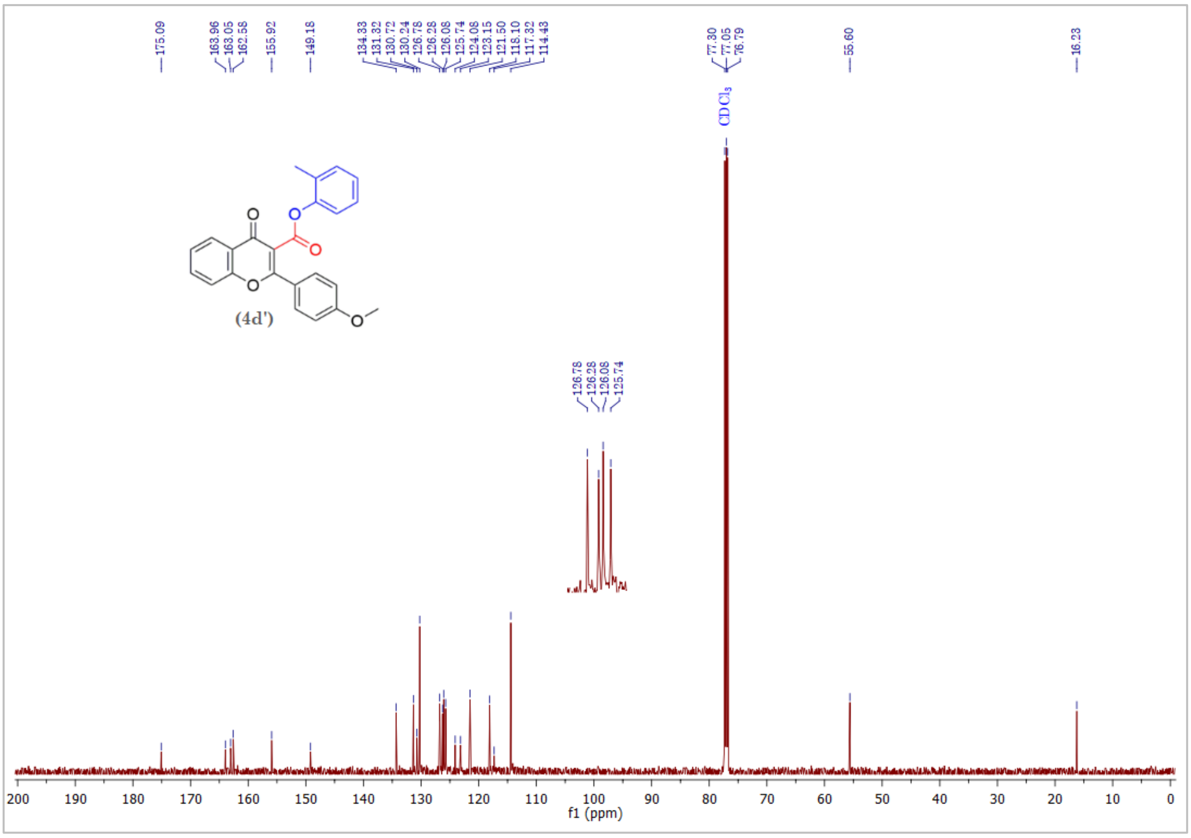

Figure S62. $^{13}C\{^{1}H\}$ NMR spectrum of compound **4d'** (125 MHz, $CDCl_3$)

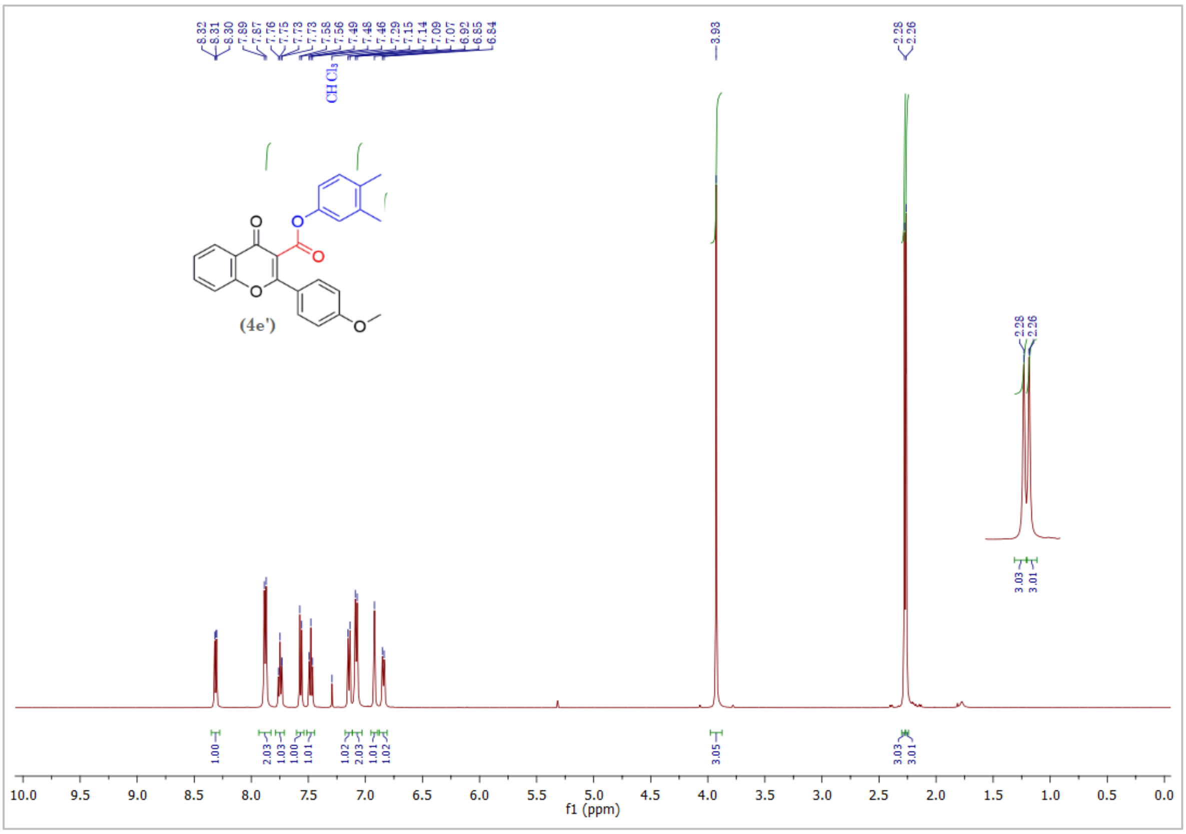

Figure S63. ^{1}H NMR spectrum of compound **4e'** (500 MHz, $CDCl_3$)

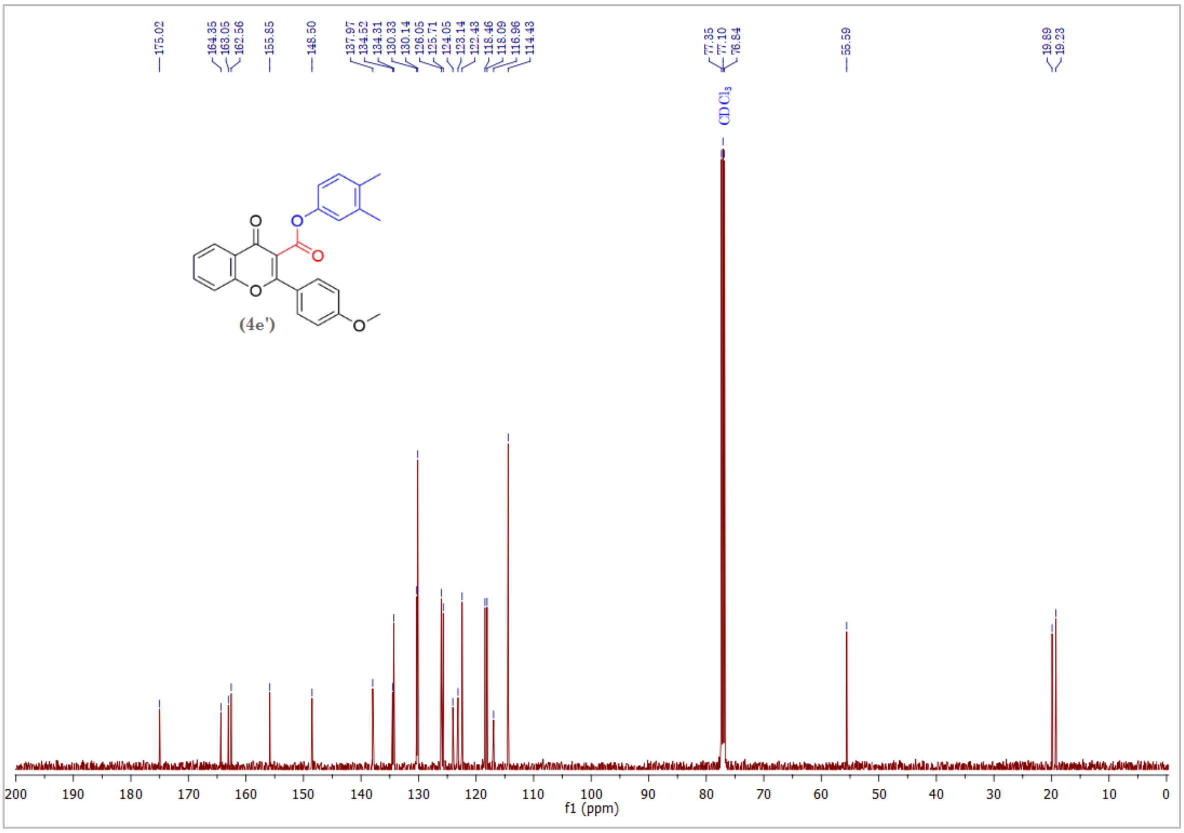

Figure S64. $^{13}C\{^{1}H\}$ NMR spectrum of compound **4e'** (125 MHz, $CDCl_3$)

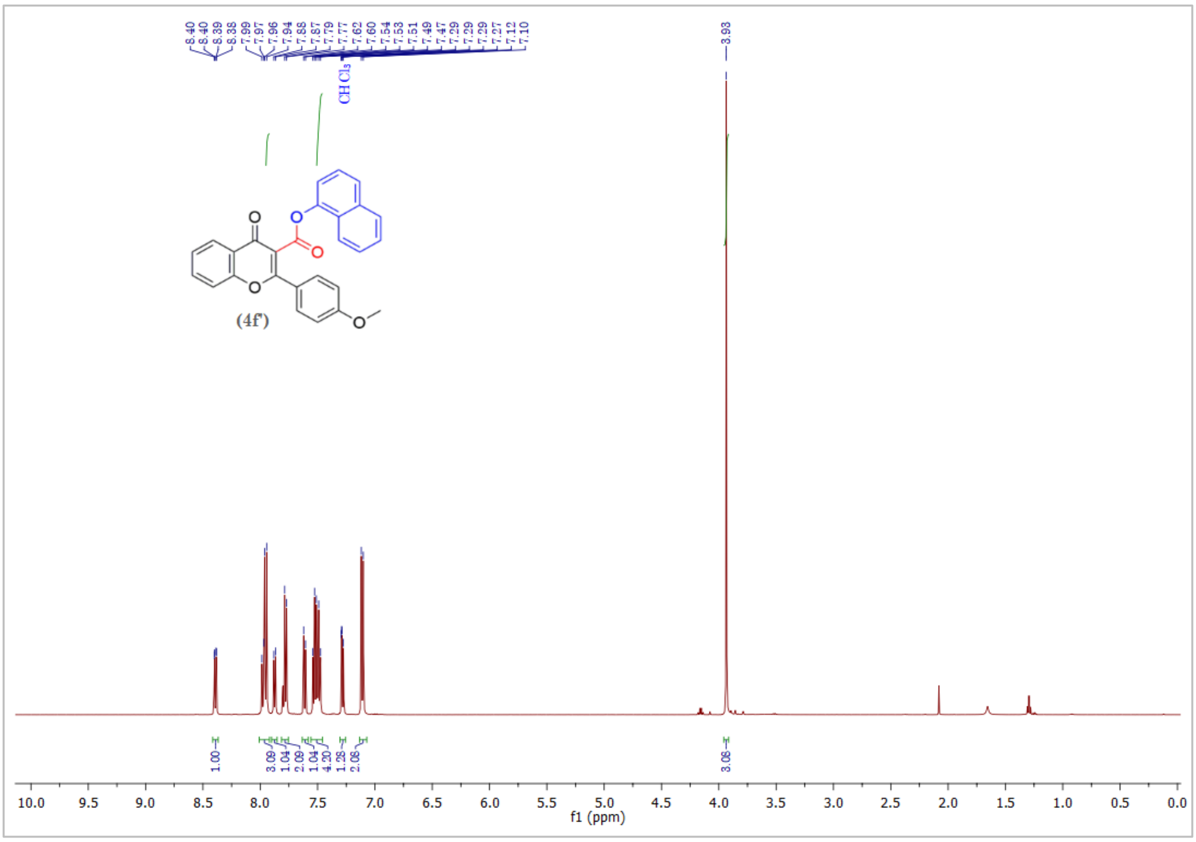

Figure S65. ^{1}H NMR spectrum of compound **4f'** (500 MHz, $CDCl_3$)

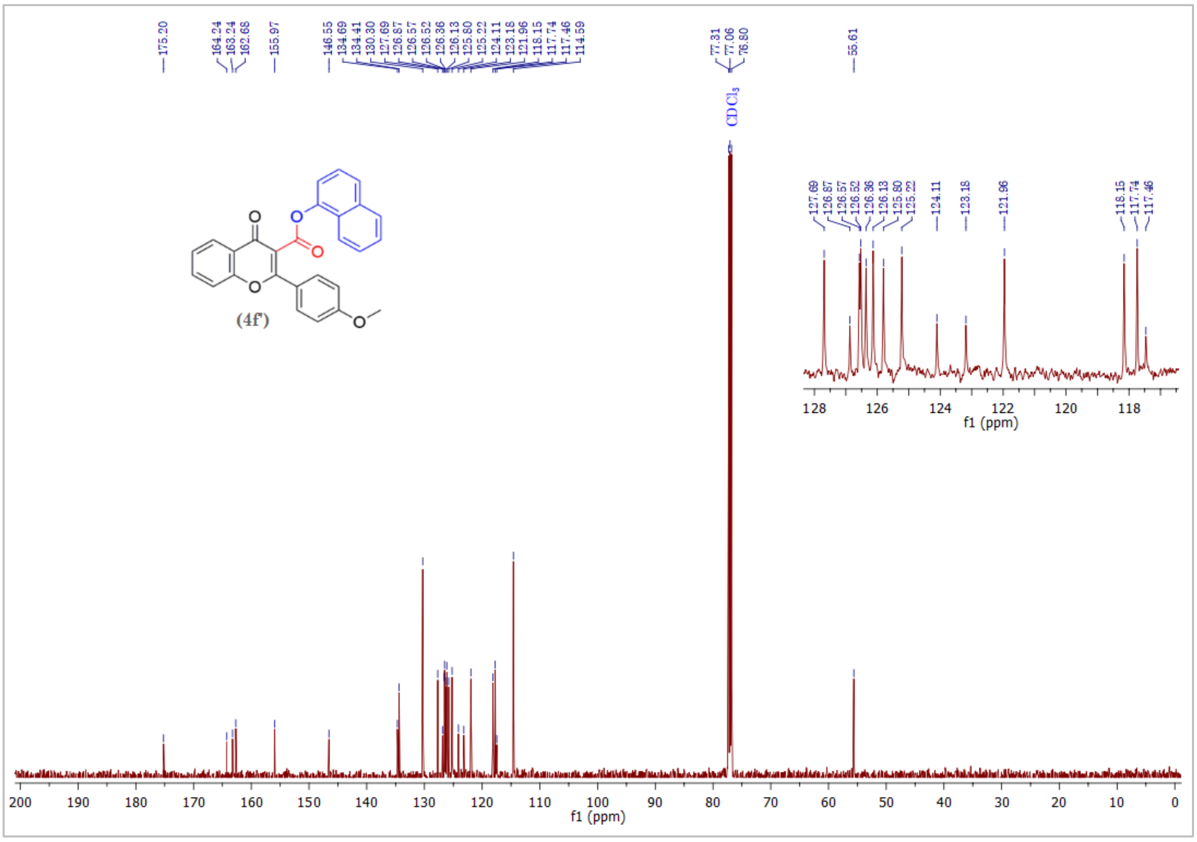

Figure S66. $^{13}C\{^1H\}$ NMR spectrum of compound **4f'** (125 MHz, $CDCl_3$)

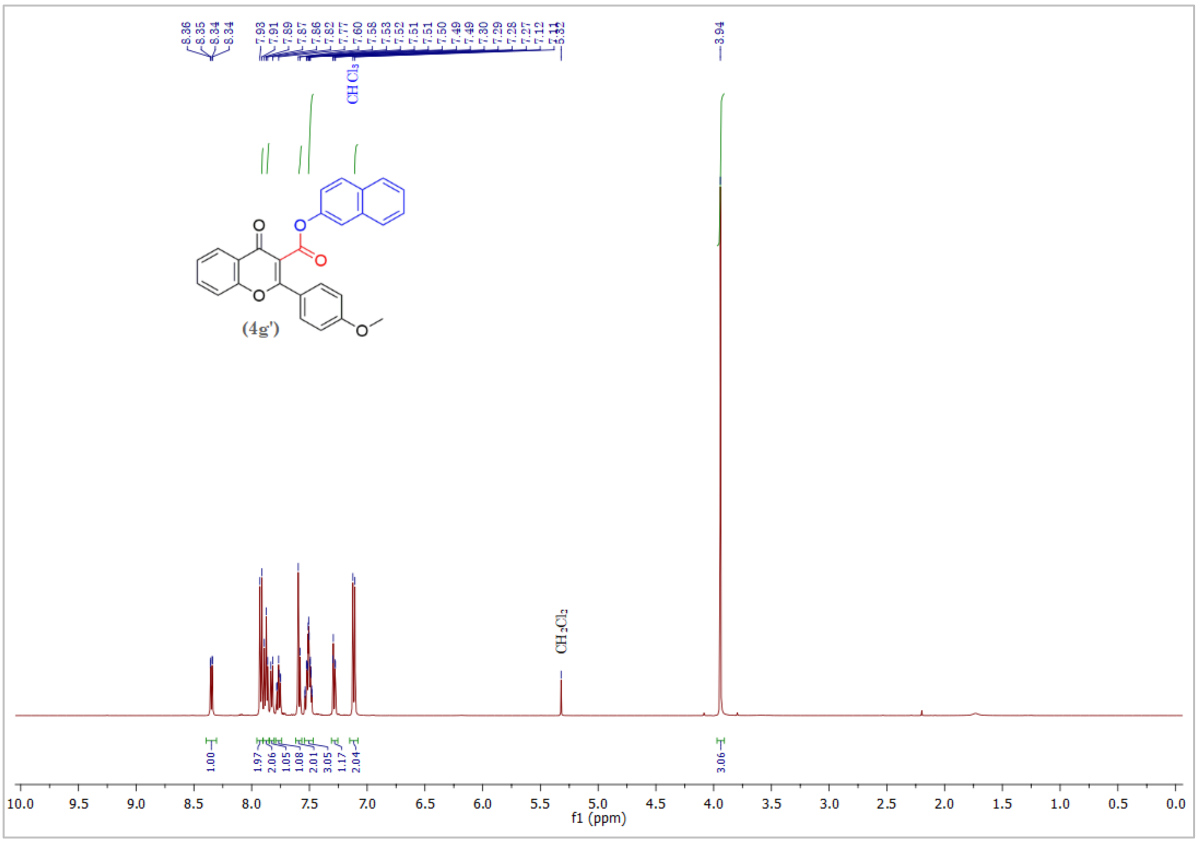

Figure S67. ^{1}H NMR spectrum of compound **4g'** (500 MHz, $CDCl_3$)

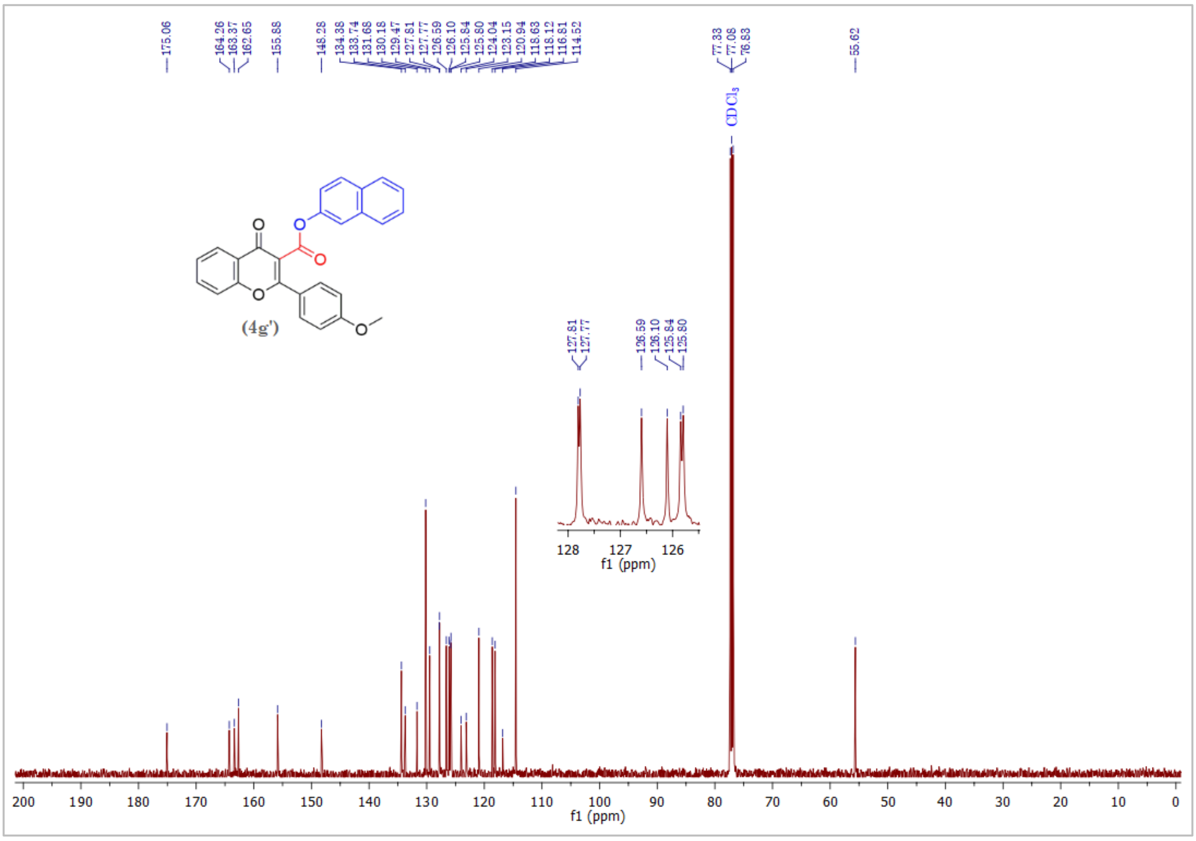

Figure S68. $^{13}C\{^{1}H\}$ NMR spectrum of compound **4g'** (125 MHz, $CDCl_3$)

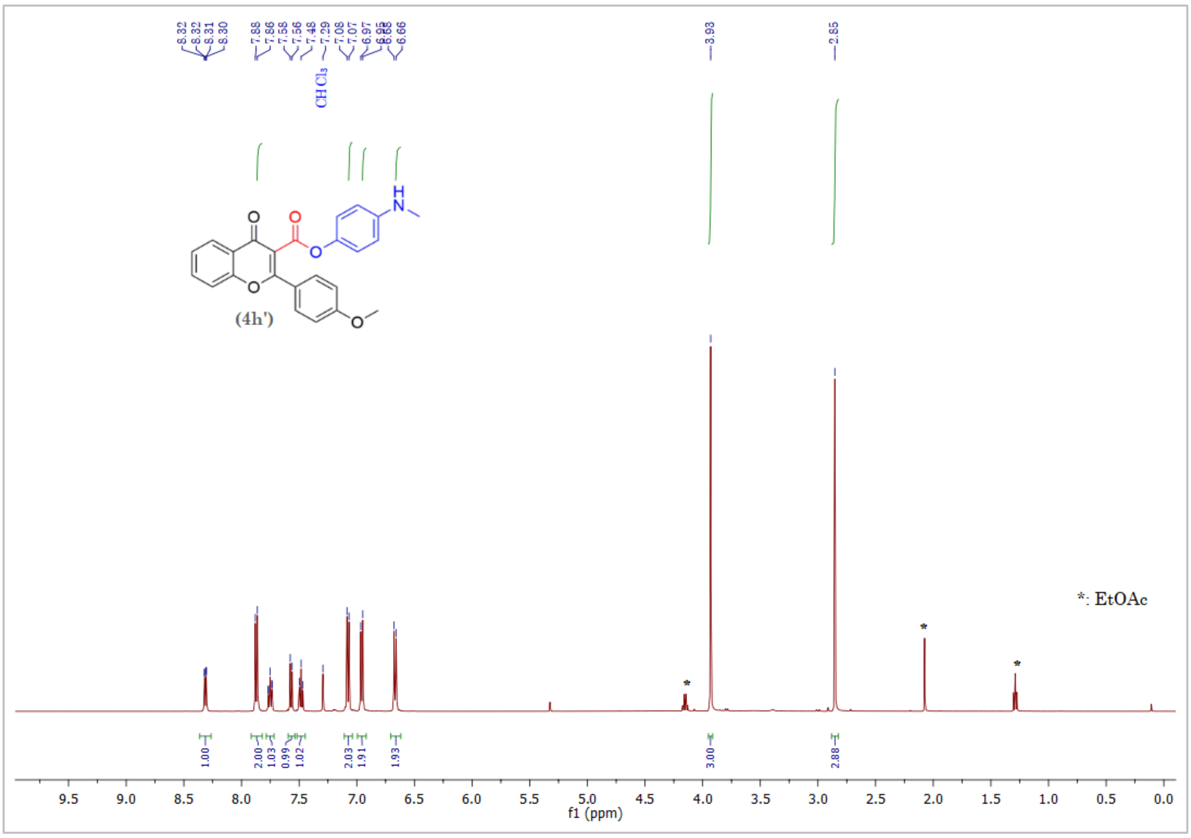

Figure S69. ^{1}H NMR spectrum of compound **4h'** (500 MHz, $CDCl_3$)

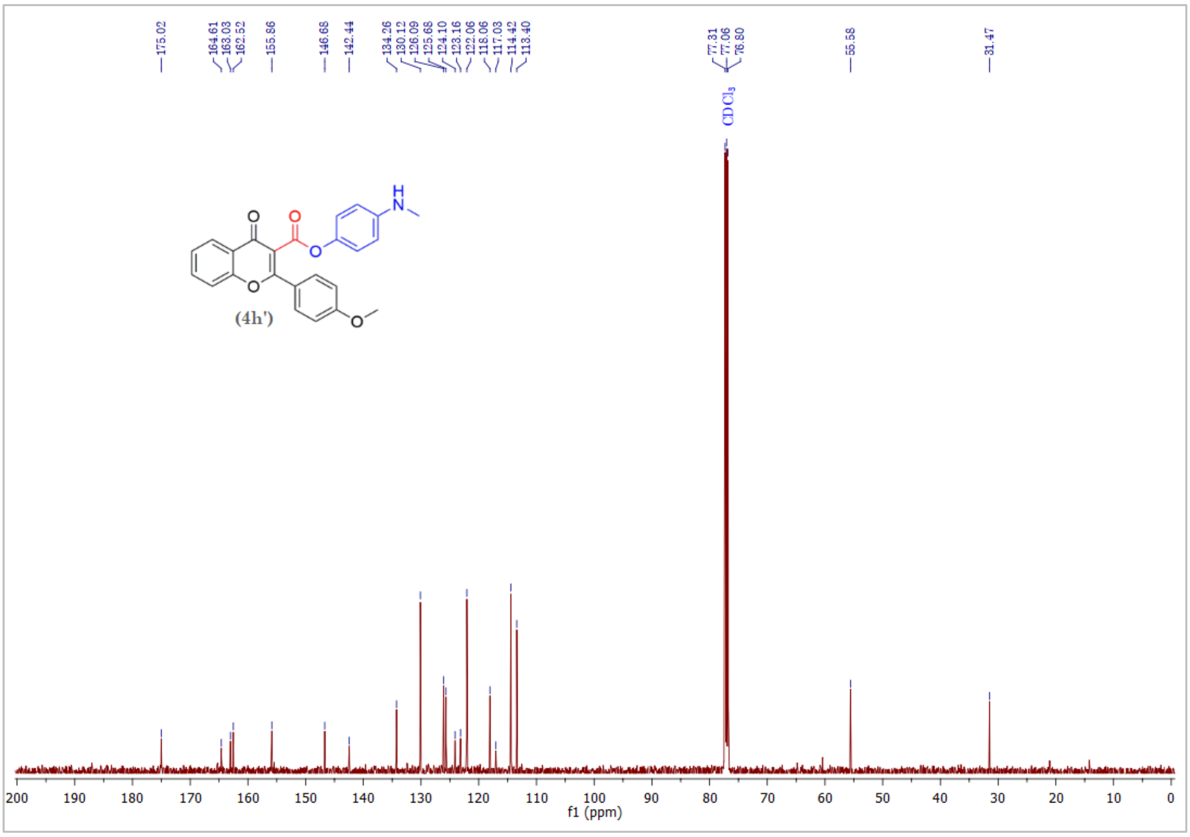

Figure S70. $^{13}C\{^{1}H\}$ NMR spectrum of compound **4h'** (125 MHz, $CDCl_3$)

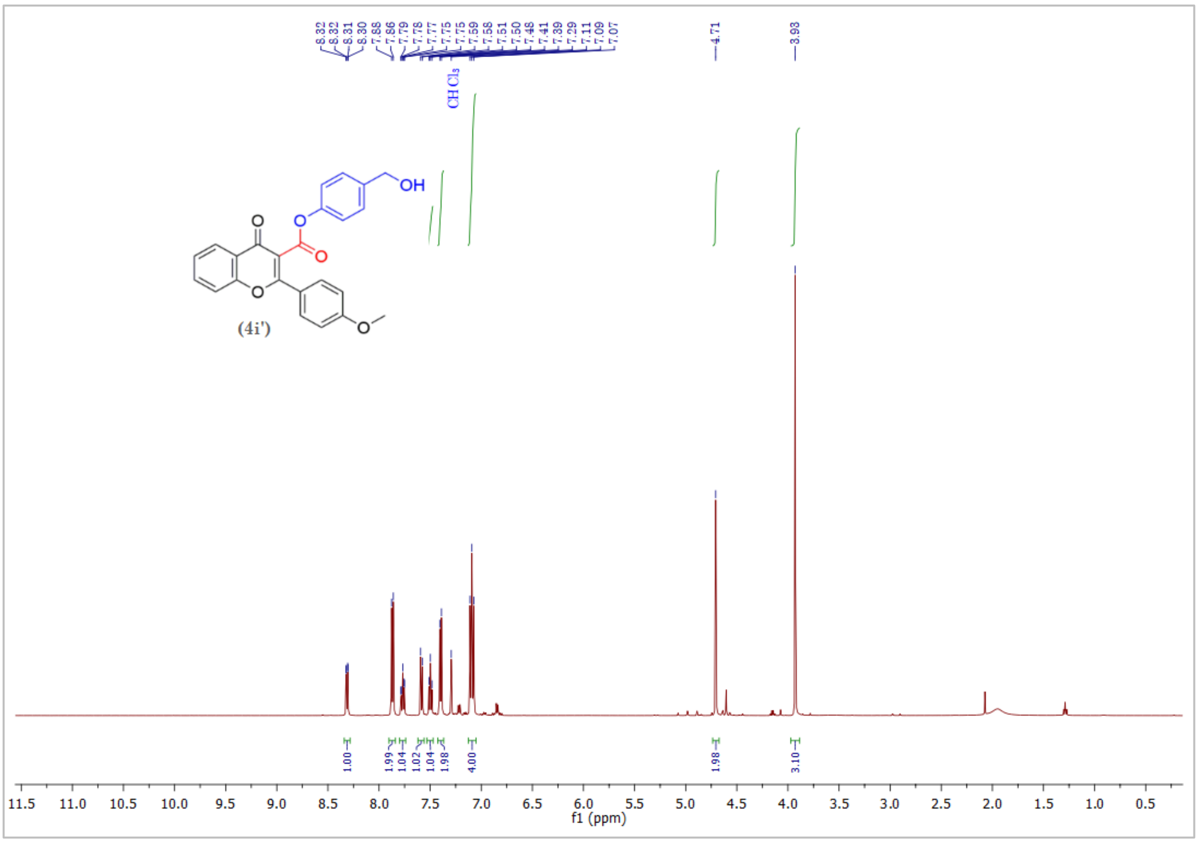

Figure S71. ^{1}H NMR spectrum of compound **4i'** (500 MHz, $CDCl_3$)

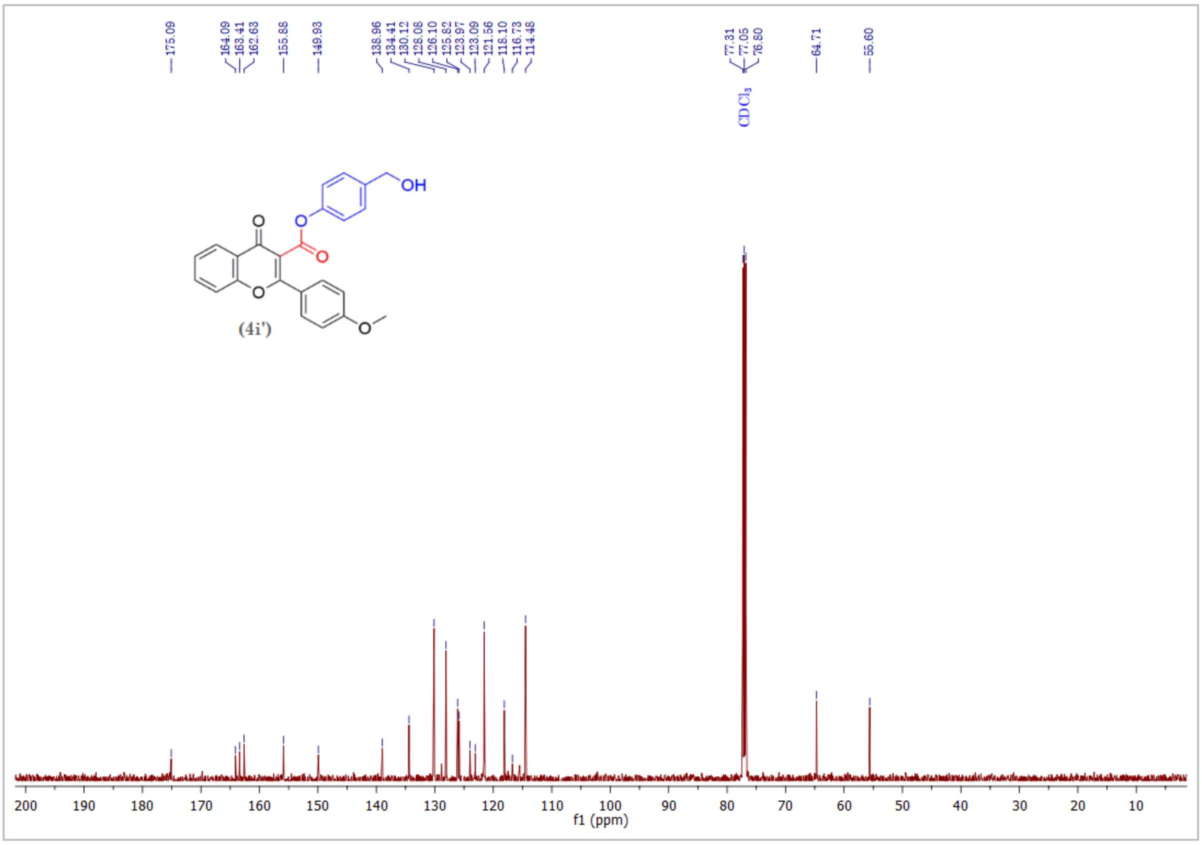

Figure S72. $^{13}C\{^{1}H\}$ NMR spectrum of compound **4i'** (125 MHz, $CDCl_3$)

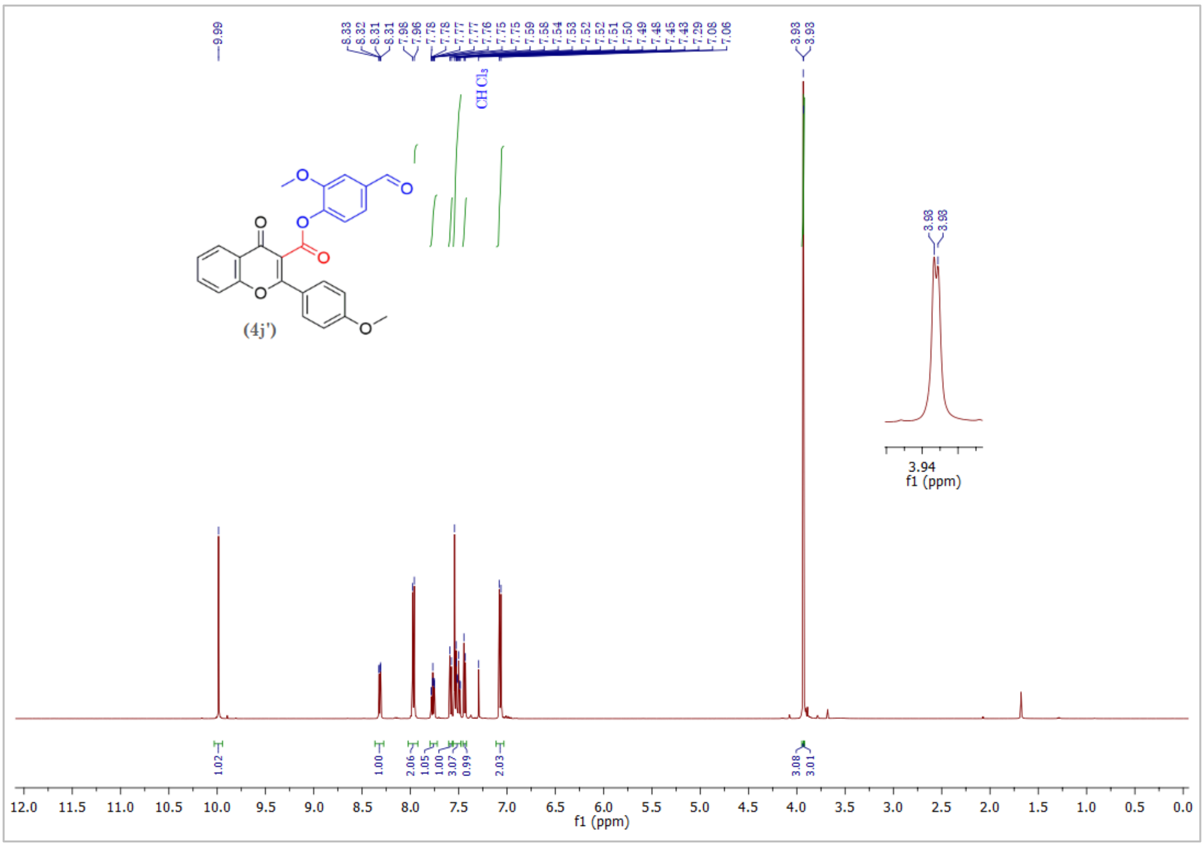

Figure S73. ^{1}H NMR spectrum of compound **4j'** (500 MHz, $CDCl_3$)

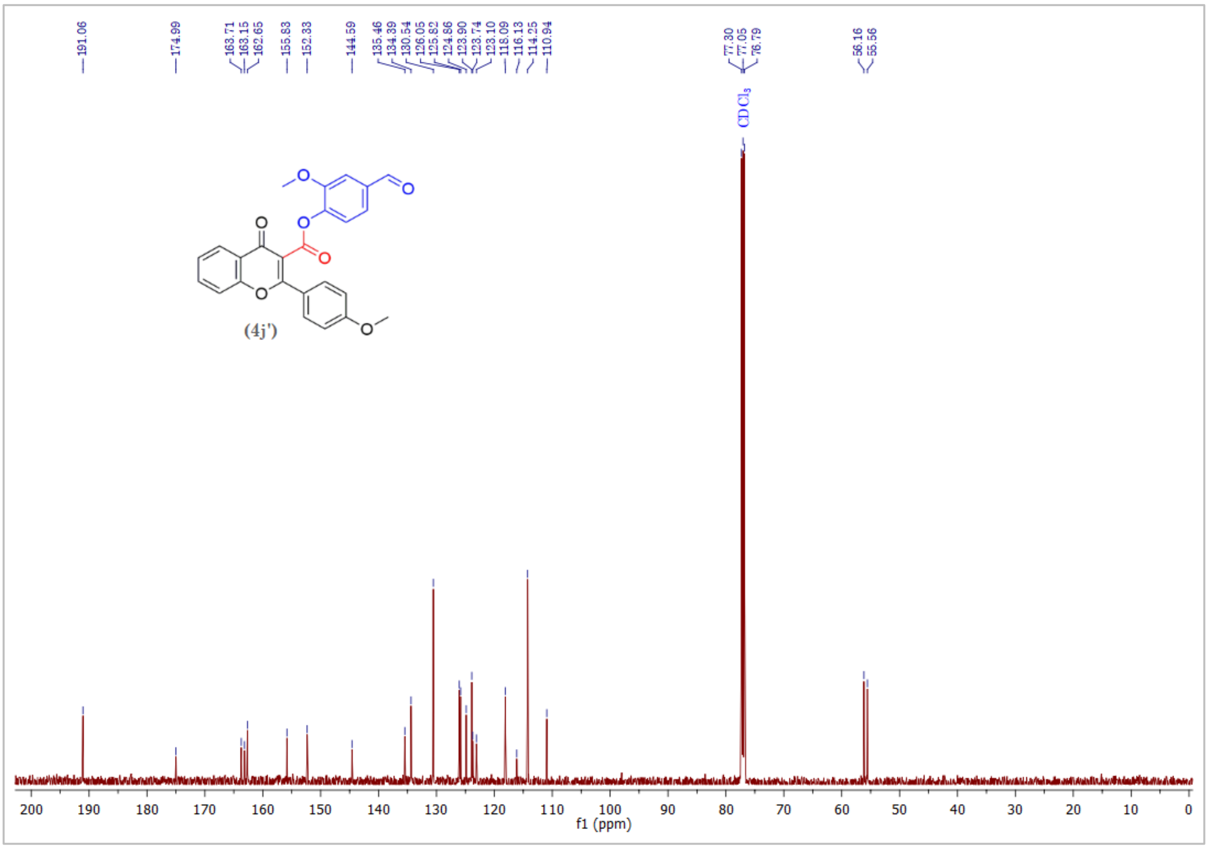

Figure S74. $^{13}C\{^{1}H\}$ NMR spectrum of compound **4j'** (125 MHz, $CDCl_3$)
